# Alkyl Thiocyanurates as Thioester Mimetics. Transthioesterification and Ligation Reactions with High Potential in Dynamic Covalent Chemistry

**DOI:** 10.1021/acs.joc.3c00200

**Published:** 2023-06-17

**Authors:** Grzegorz Wołczański, Wojciech Gil, Jakub Cichos, Marek Lisowski, Piotr Stefanowicz

**Affiliations:** Faculty of Chemsitry, University of Wrocław, F. Joliot-Curie 14, 50-383 Wrocław, Lower Silesia District, Poland

## Abstract

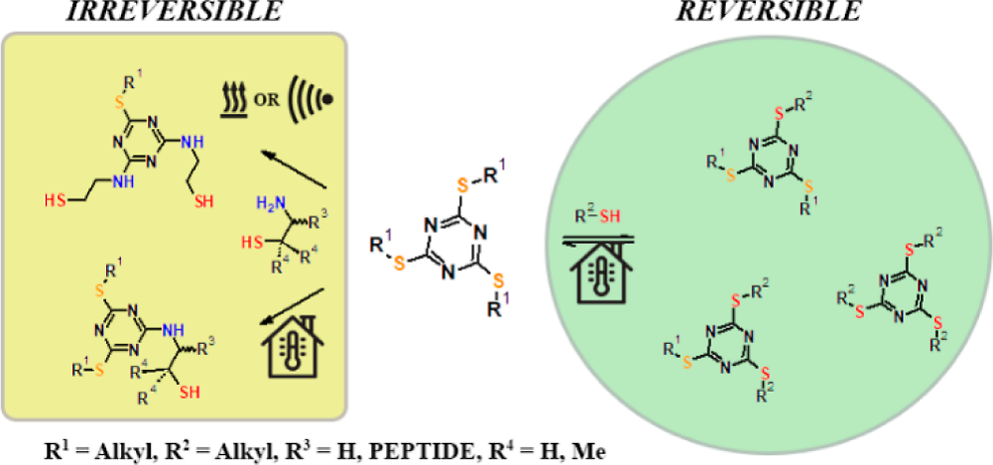

Alkyl thiocyanurates, the compounds formed in the SN reaction of thiocyanuric acid and alkyl halides, are susceptible to transthioesterification and ligation with molecules containing cysteamine, analogous to native chemical ligation of thioesters with peptides with an N-terminal cysteine moiety. The ligation is irreversible and results in the formation of mono- and disubstituted products dominantly. Transthioesterification, in contrast, is fully reversible and may be used in constructing dynamic systems. The application of this reactivity in dynamic covalent chemistry has been exemplified by the preparation of a library of mixed thiocyanurates of glutathione and thioglycolic acid with self-assembly abilities and metathesis between thiocyanurates of tris(carboxymethyl) and tris(carboxamidomethyl) catalyzed by MESNa (sodium 2-mercaptoethylsulphonate) or MPAA (4-mercaptophenylacetic acid). Differences in reactivity of thiocyanurates toward cysteamines and thiols has been explained based on conceptual DFT.

## Introduction

**Scheme 1:**
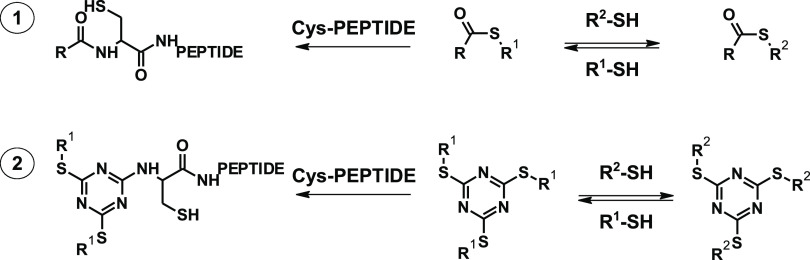
Similarities in the Reactivity of Carboxylic Acid Thioesters (1) and Expected Reactivity of Thiocyanuric Acid Esters (2)

In one of our previous works, we described the preparation of peptides conjugated with 1,3,5-trimercaptobenzene by one of its sulfhydryl groups, followed by oxidation, giving dynamic libraries of template-assembled synthetic proteins (TASP) molecules.^1^ In the same work, we described unsuccessful efforts to synthesize the triaza-analogues of those compounds. We found that the N-terminal [(4,6-bissulfanyl-1,3,5-triazin-2-yl)sulfanyl]acetyl group in modified peptides (abbreviated as TMT-Ac-peptides) is unstable during a prolonged standing at mild basic aqueous conditions at room temperature. Moreover, the thiocyanurate moiety was immediately converted into the mercaptoacetyl in the presence of free thiols (e.g., dithiothreitol, mercaptoethanol, ethylenedithiol) in the reaction mixture. These observations suggest that the sp2 carbon atom of thiocyanuric ester is prone to transthioesterification and possibly exhibits a reactivity similar to carboxylic thioesters, well known as substrates in syntheses of long peptides and small proteins by the native chemical ligation (NCL) (Scheme 1). There is only one literature report on the nucleophilic substitution of methanethiol in trimethyl thiocyanurate, but the use of sodium methoxide is required there.^2^ According to our observations, transthioesterification of alkyl thiocyanurate occurs in mild conditions (aqueous buffer and moderate pH). Thus, we found it important to explore this reaction in detail.

## Results and Discussion

### Exploring Reactivity of Thiocyanurates with Cysteamines

**Figure 1:**
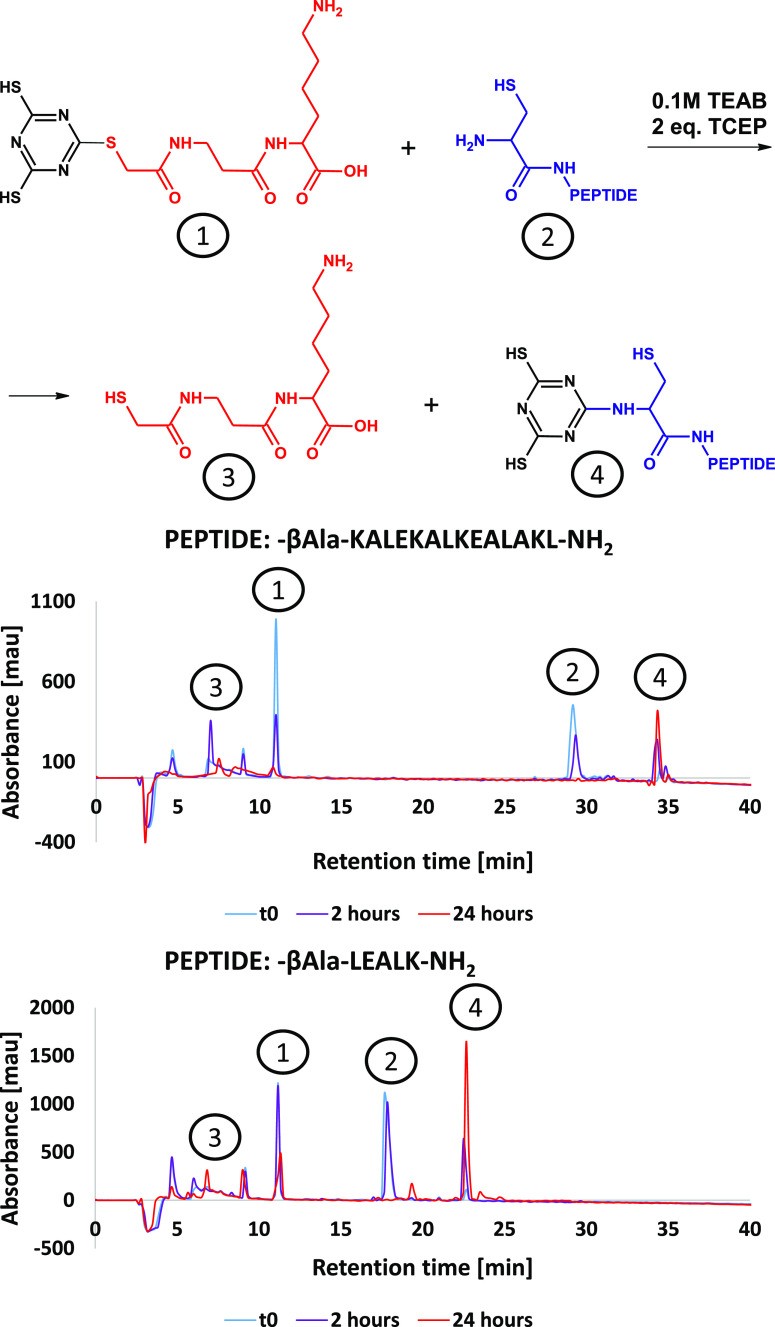
Ligation of Cys-peptides to monoesters of thiocyanuric acid.

If our predictions were correct, the thiocyanurate ester would react with peptides containing the N-terminal cysteine residue. It is in line with our interest in synthesizing new peptide conjugates and creating dynamic covalent libraries of TASP molecules. At first, we decided to test the reactivity of thiocyanuric acid monoesters. For this purpose, we prepared bromoacetyl-β-alanyl-lysine on a chlorotrityl solid support by the ultrasonic Fmoc protocol,^3^ followed by bromoacetylation using bromoacetic acid and *N*,*N*-diisopropylcarbodiimide in DMF. The BrAc-βAla-Lys-OH was cleaved from the resin using 5% TIS in 1% TFA in DCM. After evaporation under nitrogen, the crude product reacted with an excess of thiocyanuric acid in the presence of Hünig’s base. The resulting TMT-Ac-βAla-Lys-OH is stable enough to be partially purified by reversed-phase (RP)-HPLC. TMT-R means monoester of thiocyanuric acid, where R is the alkyl group and TMT is the 2,4,6-trissulfanyl-1,3,5-triazine (thiocyanuric acid; 2,4,5-trimercapto-1,2,3,-triazine) scaffold. However, HPLC analysis of collected fractions measured with time showed that the compound is slowly hydrolyzed in aqueous conditions. We also checked the stability of the compound after the lyophilization of collected fractions. The RP-HPLC-UV analysis did not show any evidence of a progressive decay of the thioester after such a treatment. For ligation experiments, two model peptides containing the N-terminal cysteine residue were prepared: short (heptapeptide) and long (hexadecapeptide), further abbreviated SM and LM, respectively. During ligation experiments, the 2 mM peptide solution in 0.5 M triethylammonium bicarbonate (TEAB) buffer containing 5 mM tris(2-carboxyethyl)phosphine (TCEP) and adjusted to pH 8.5 was mixed with 2 equivalents of TMT-Ac-βAla-Lys-OH and incubated at room temperature on a rotary shaker. The RP-HPLC-UV analysis and off-line characterization of collected fractions by high-resolution mass spectrometry showed full conversion of Cys-peptides into DMT-Cys-peptides after 24 h (Figure 1), where DMT is N-terminal 4,6-disulfanyl-1,3,5-triazin-2-yl group. Being interested in the synthesis of TASP molecules, we examined the air oxidation of the obtained compounds. Even after a long incubation time in TEAB buffer bubbled by air, also after the addition of iodine dissolved in methanol, we observed only selective disulfide bond formation between sulfhydryl groups of Cys residues. The chemical inertia of two free sulfhydryl groups of the dithiocyanuramide moiety toward mild oxidants could be explained by thione-thiol tautomerism and a high contribution of the thioketone tautomers to the resulting reactivity. This assumption is supported by results published by Stoyanova et al.^4^ They demonstrated that more polar thione tautomers of 2- or 4-mercaptopyridine and 2-mercaptopirimidine are preferentially stabilized in more polar solvents and with a proton-donating ability like water or ethanol. Due to the partial instability of the thiocyanuric acid monoesters, we did not investigate the transthioesterification reaction for this class of compounds as it would have no practical significance in dynamic covalent combinatorial chemistry.

Enriched by this knowledge, we turned our attention to exploring the reactivity of thiocyanuric acid tris(alkyl)esters. Representatives of this class of compounds can be readily synthesized by treating thiocyanuric acid with an excess of an alkyl halide in the presence of non-nucleophilic bases. The simplest compound examined was tris(carboxymethyl) thiocyanurate, abbreviated further as TMT(AcOH)_3_ [TMT(R^1^)_*X*_(R^2^)_*Y*_(R^3^)_*Z*_—tris(alkyl) tiocyanurate, where R^1^, R^2^, and R^3^ are alkyl substituents, *X* + *Y* + *Z* = 3], chosen for its very good water solubility in neutral or moderately alkaline environments. TMT(AcOH)_3_ is stable for at least 24 h at an aqueous basic condition up to pH 10 at 70 °C or even to pH 12 at 40 °C (see Supporting Information for details). The higher stability of the triester in comparison to the monoester of thiocyanuric acid is caused by the lack of thione-thiol tautomerism and the resulting increase in the aromaticity of the triazine ring. Any attempt to react TMT(AcOH)_3_ with a 3-fold or higher excess of cysteinyl peptides in an aqueous medium resulted in DMT(AcOH)_2_(peptide). DMT(R^1^)_*x*_(R^2^)_*Y*_(R^3^)_*Z*_ means *N*-[4,6-bis(alkylsylfanyl)-1,3,5-triazin-2-yl]amines, where R^1^ and R^2^ are alkyl substituents connected directly with sulfur atoms, R^3^ is the amine, and *X* + *Y* + *Z* = 3. This observation agrees with trends in reactivity in the aromatic nucleophilic substitution. Strong electron-donating properties of the amine group reduce the reactivity of other sp^2^ carbon atoms in the triazine ring toward the nucleophilic attack of mercaptans.

To better describe the properties of this connection, we synthesized simplified model compounds by reaction of cysteamine, cysteine, or penicillamine with TMT(AcOH)_3_ in 0.5 M TEAB buffer. A 4-fold excess of the sulfide group donor was used, and the reactions were carried out at 40° for 24 h. We observed a quantitative conversion of TMT(AcOH)_3_ into DMT(AcOH)_2_(cysteamine) or DMT(AcOH)_2_(Cys) and only a partial conversion into more sterically hindered DMT(AcOH)_2_(Pen), which was obtained in a 25% yield. All products were purified and fully characterized by mass spectrometry and NMR spectroscopy. NMR spectra were measured in DMSO-*d*_6_ at 300 K. All diastereotopic protons are visible as well-defined and separated multiplets. The most interesting is an additional small doublet accompanied by a much greater one above 8 ppm. Both signals correspond to the NH proton of Cys or Pen residues and have the same coupling constants. Also, multiplets of α-CH protons have their neighbors in these cases, and the signal area ratios for both groups of protons are equal. Such an additional set of signals does not occur in the ^1^H NMR spectrum of symmetrical DMT(AcOH)_2_(cystemine). In the 1H NMR spectrum of DMT(AcOH)_2_(d-Cys), there are also additional neighbors next to the signals of NH and α-CH groups, but their relative intensities are different than for DMT(AcOH)_2_(Cys). We believe that an additional carboxyl group in Cys and Pen is responsible for the differentiation of 1H NMR shifts of NH and α-CH protons due to the place of protonation of the triazine ring. The explanation seems to be very clear. The NH group of cysteamines loses its basic properties starting to behave like an amide group when bound to the triazine ring. Then the nitrogen atoms of the triazine ring remain the only hydrogen bond acceptors in the final molecule and stay protonated through intra or intermolecular interactions. Considering the 1H NMR spectra once again, we can conclude that the intramolecular hydrogen bond is mainly formed by one of the −AcOH substituents, while the carboxyl group of the third substituent is involved in the hydrogen bond formation in 38, 15, and 5 percent in the case of d-Cys, l-Cys, and l-Pen, respectively (NMR spectra were measured in DMSO-*d*_6_ at 300 K, the concentration of compounds was 10–20 mM). Differences in observed participation of substituents in hydrogen bond formation may result from different amounts of water remaining in the samples after the lyophilization of purified compounds.

**Figure 2:**
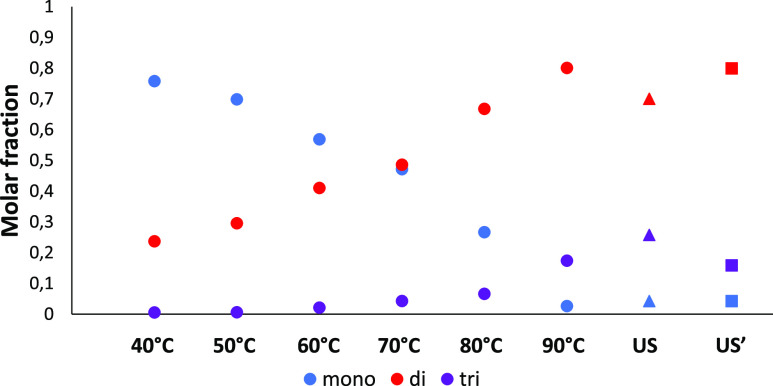
Molar fractions of products created by mono-, di-, and tri-substitution of glycolic acid residue in the reaction of TMT(AcOH)_3_ with cysteamine at temperatures between 40 and 90 °C (circles) or ultrasonic conditions (triangles), and the composition of DMT (blue square), MMT(red square), and TAT (violet square) obtained by ultrasonication.

We carried out the reaction at a higher temperature in TEAB buffer to see if it is possible to substitute more than one thioglycolic acid residue with 5 equivalents of cysteamine. Up to 50 °C, we observed the selective formation of DMT(AcOH)_2_(cysteamine) while starting from 60 °C the disubstituted MMT(AcOH) (cysteamine)_2_ started to rise, giving the monomercaptotriazine (MMT) as the main product at 90 °C. MMT(R^1^)_*X*_(R^2^)_*Y*_(R^3^)_*Z*_ means *N*-[4-(alkylamino)-6-(alkylsulfanyl)-1,3,5-triazin-2-yl]amines, where R^1^ is the alkyl substituent directly connected with the sulfur atom, R^2^ and R^3^ are amines, and *X* + *Y* + *Z* = 3. Additionally, at 90 °C, the main peak is accompanied by a smaller and partially coeluted peak, which corresponds with the trisubstituted TAT(cysteamine)_3_– *m*/*z* [M + H]^+^ = 307.0860 Da. TAT(R^1^)_*X*_(R^2^)_*Y*_(R^3^)_*Z*_ means 2,4,6-tris(alkylamino)-1,3,5-triazines, where R^1^, R^2^, and R^3^ are amines, and *X* + *Y* + *Z* = 3. If the reaction is performed in a glass vial dipped in an ultrasonic bath, then the disubstituted product is preferentially formed after 2 h of sonication (starting from room temperature, without temperature control). Additional signal with lower retention time is observed on the chromatogram registered with UV detection and corresponds to *m*/*z* [M + H]^+^ = 307.0855 Da, which was identified as MMT(cysteamine)_3_ (based on extracted UV absorption spectrum)—the trisubstituted product with one of the cysteamines connected to the triazine ring through the amine group. The TAT(cysteamine)_3_ is observed by LC–MS analysis, which shows that it coelutes with MMT(AcOH) (cysteamine)_2_. Based on extracted ion chromatograms, we analyzed molar fractions of mono-, di-, and trisubstituted products concerning the reaction conditions (Figure 2). In terms of the degree of substitution, the post-reaction mixture obtained in ultrasonic conditions is more advanced, but in terms of the type of molecules the composition of DMT, MMT, and TAT are almost identical to the mixture of products obtained by heating at 90 °C. An observation of MMT(cysteamine)_3_ in ultrasonic conditions means that at elevated temperatures the rate-limiting step is the intermolecular reaction between a triazine and cysteamine. The sonication accelerates the intermolecular reaction events by facilitating diffusion, while the intramolecular S-to-N migration is only slightly affected. The reactivity transthioesterification decreases in the order of TMT > DMT > MMT. The last substitution leading to a TAT molecule has limited importance, as it is obtained as a side-product only in harsh conditions.

The DMT(AcOH)_2_cysteamine and MMT(AcOH) (cysteamine)_2,_ have been isolated and characterized by MS and NMR. We do not observe an additional small signal accompanying the main signal of the amide proton on the 1H NMR spectra of those compounds. Thus, the SH group does not form a hydrogen bond with the triazine ring. This supports indirectly the hypothesis about a competitive formation of an intramolecular hydrogen bond between different carboxyl groups and the triazine ring in DMT(AcOH)_2_CysOH and its analogues. The NMR spectra of MMT(AcOH) (cysteamine)_2_ are more complicated. Inhibition of the *N*-triazine bonds rotation results in an occurrence of all three possible forms due to the mutual position of 2-mercaptoethyl groups, which is experimentally observed in splitting all signals on the 1H NMR and ^13^C NMR spectra. See Supporting Information for details.

**Scheme 2:**
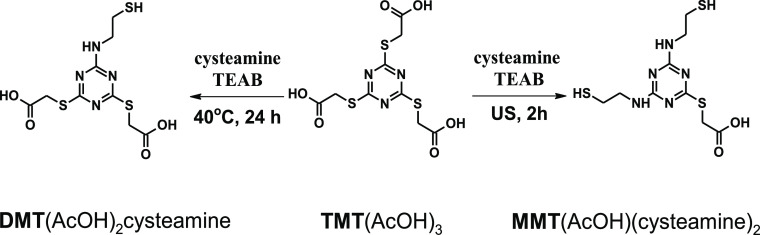
Types of Mercaptotriazines Observed in the Ligation Reaction of Cysteamine TMT = trimercaptotriazine, DMT = dimercaptotriazine, MMT = monomercaptotriazine.

Summarizing, the reactivity of thiocyanuric acid alkyl esters toward unprotected 2-mercaptoalkylamines under mild basic conditions is practically limited to mono- or occasionally disubstitution throughout an irreversible ligation reaction (Scheme 2), which is of little importance from the point of view of dynamic covalent chemistry. However, we believe that the described reactivity may be useful for the design of new selective reagents for the derivatization of cysteamines, similar to the recently published fluorescence probes based on ligation of cysteinyl-peptides to a *meso*-thioester-BODIPY.^5^

### Thiols Exchange

**Figure 3:**
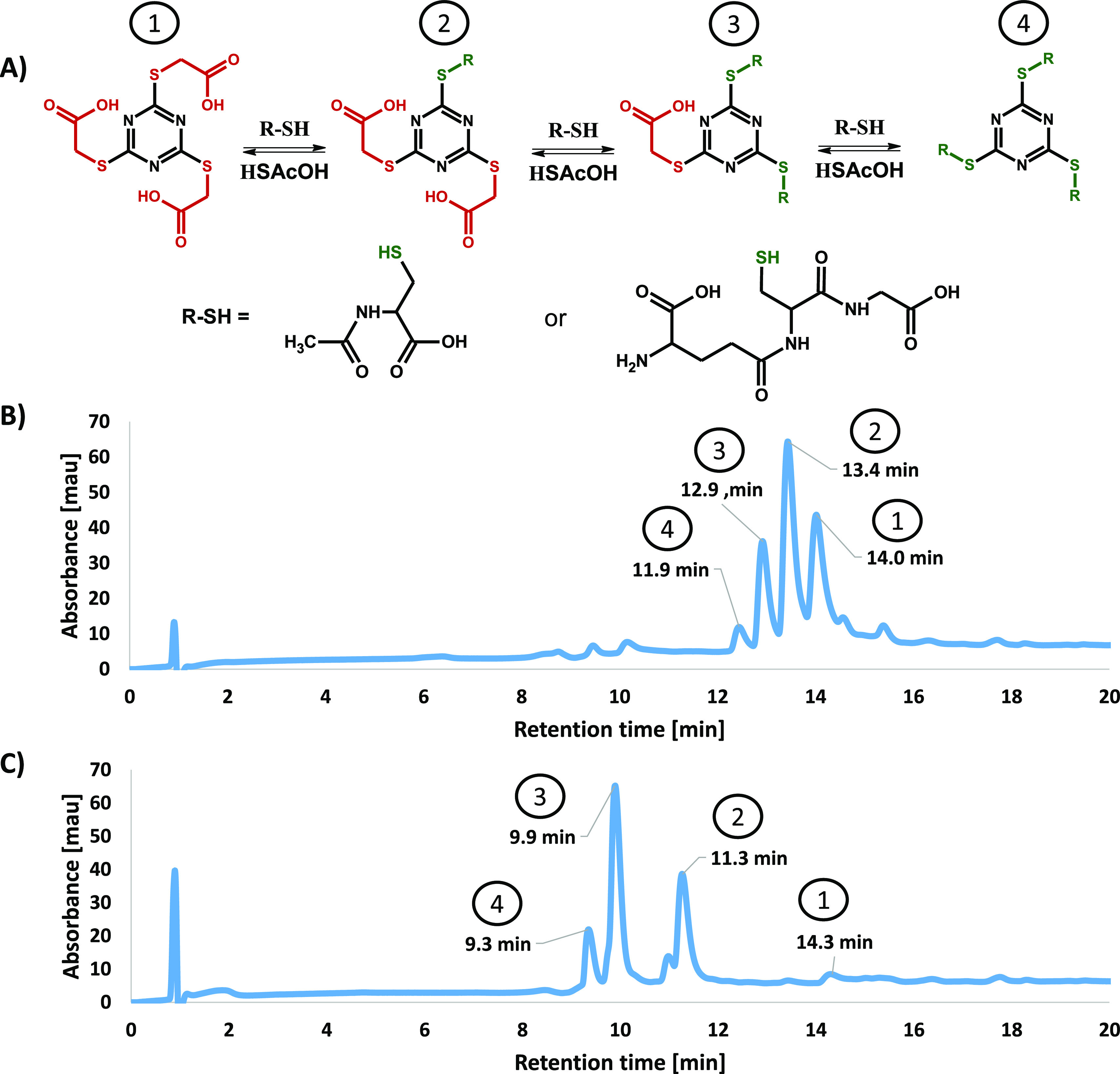
Transthioesterification of tris(carboxymethyl) thiocyanurate with simple peptides containing internal Cys residue: (A) scheme of the equilibria, (B) HPLC analysis of an equilibrated mixture of TMT(AcOH)3 with 4 equiv of AcCysOH, and (C) with 4 equiv of glutathione.

Due to the limitations of the above-discussed ligation reactions, we focused on the transthioesterification of thiocyanurates with compounds containing a free sulfhydryl group. We conducted two parallel experiments using AcCysOH and glutathione as thiols in model reactions (Figure 3). Further down in the text, glutathione in TMT(AcOH)_*X*_(glutathione)_*Y*_ and AcCysOH in TMT(AcOH)_*X*_(AcCysOH)_*Y*_ (where *X* + *Y* = 3) means that the sulfanyl radical form of this compound as substituent directly attached to the triazine ring. After 24–48 h of incubation, we did not observe further changes in the reaction mixture composition, which means that the system gained thermodynamic equilibrium. In contrast to unprotected 2-mercaptoalkylamines (cysteamines), the 4-fold excess of AcCysOH acting on TMT(AcOH)_3_ gave a mixture of all possible products—TMT(AcOH)_2_(AcCysOH), TMT(AcOH) (AcCysOH)_2_, and TMT(AcCysOH)_3_. The purified compounds were characterized by MS/MS^2^ and NMR, which fully supported these findings. A quasi-normal distribution of each triazine forms in the post-reaction mixture corresponds to a system where the exchange of thiol ligands in the triazine ring is kinetically controlled. As could be expected, the content of the triazine substrate is still higher than the fully exchanged product due to the steric hindrance of AcCysOH which results in a faster attack of thiolate obtained from thioglycolic acid than from AcCysOH.

As in the previous case, in the reaction of 4 equivalents of glutathione with TMT(AcOH)_3_, all possible products are observed in the reaction mixture. The disubstituted product is dominant, and the equilibrium is shifted toward the replacement of thioglycolic acid residues with glutathione more than in the case of AcCysOH. It appears that the distribution of triazine forms at equilibrium cannot be explained only by the exchange kinetics dependent on the structure of the nucleophile. It would seem that a larger, more complex molecule should be a worse nucleophile in the transthioesterification reaction. The result of the experiment can be explained by assuming that the exchange process in the case of glutathione is driven by a kind of pre-organization of two glutathione chains, favoring the attachment of the next glutathione instead of the reverse exchange. On the other hand, the distance of the negative charge from the reaction center may also affect the degree of ligand exchange. The carboxyl group of AcCysOH is relatively closer to the reacting SH group than is the case in glutathione. Thus, during the substitution of thioglycolic acid residues by glutathione, the electrostatic repulsion of negative charges of both ligands may play a less important role. Nonetheless, the oxidized glutathione can self-assemble itself, forming ordered fibrous structures with gel-like behavior in aqueous organic solutions,^6^ and can stimulate the amyloid formation of α-synuclein—probably by initial self-assembly.^7^ Thus, assembling effects of the glutathione substituent favoring further substitutions cannot be completely excluded.

Our glutathione derivatives appear to mimic the self-assembling systems described by Matsuura et al. They consist of trigonal glutathione conjugates with 1,3,5-tris(2-mercaptoethylaminocarbonyl)benzene or 2,4,6-tris[*N*-(mercaptoacetyl)aminamethyl]-1,3,5-trietylobenzene.^8^ These systems can self-assemble into viral capsid-like nanospheres. Having synthesized compounds and looking for possible explanations for differences in the composition of AcCysOH and glutathione-based libraries, we decided to investigate the possibility of similar behavior in our glutathione conjugates.

We analyzed the self-assembly properties of individual compounds to find potential differences in self-aggregation preferences. Purified glutathione conjugates were dissolved in water at a 10 mM concentration of the thiocyanurate, resulting in clear solutions without a visible precipitate. The obtained solutions were left for 5 days to stand at 4 °C. We did not observe changes in viscosity during the equilibration of the glutathione–triazine conjugates solutions. Even after a few days, the samples stayed transparent and clear. The obtained aged aqueous solutions were applied to the carbon-coated Cu-grid, and specimens were observed by transmission electron microscopy (TEM, the samples were stained by uranyl acetate). Simultaneously, the samples were analyzed by dynamic light scattering to verify the identity of structures observed in TEM with real objects in solutions.

**Figure 4:**
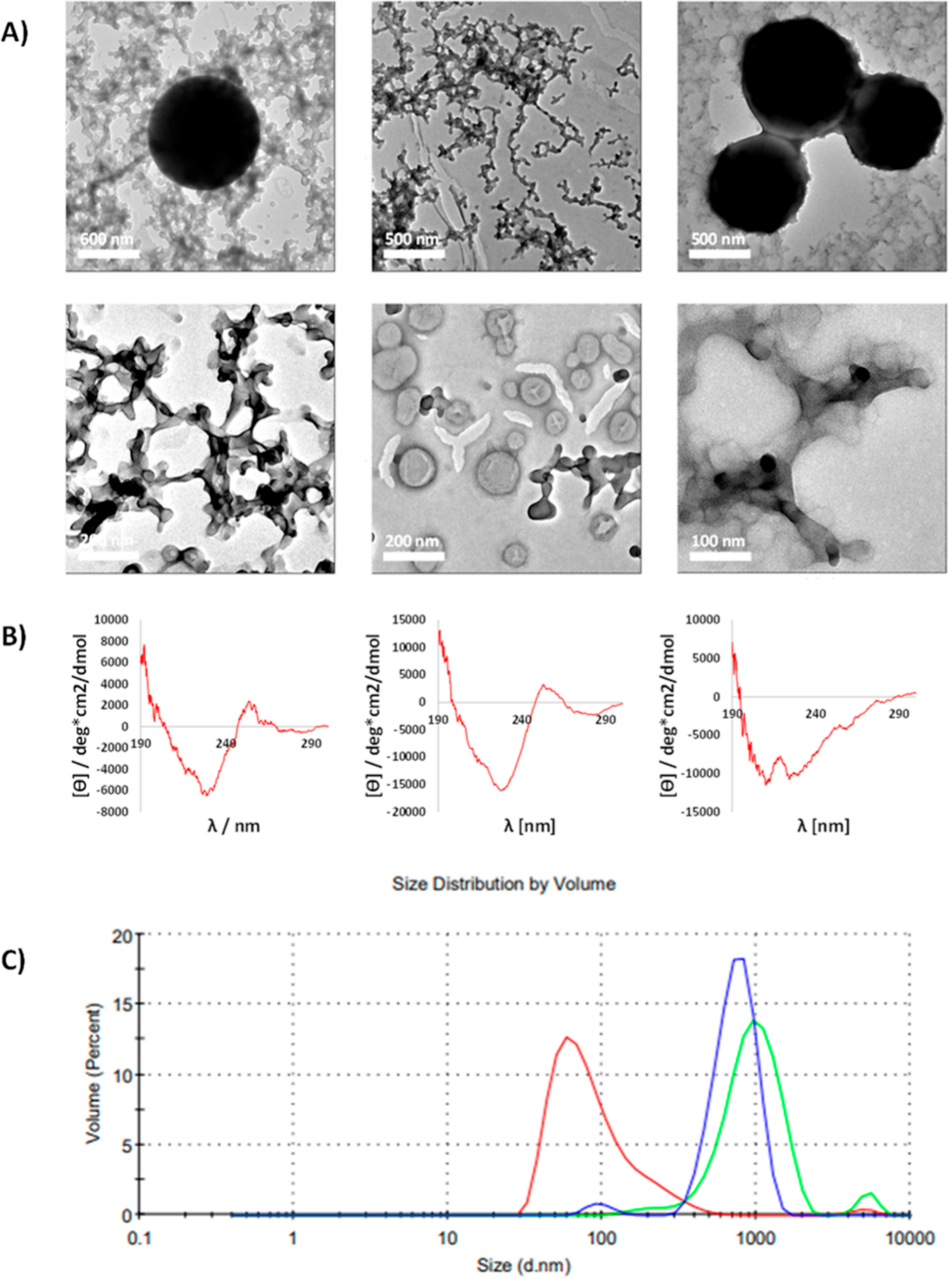
Investigation on self-assembly preferences of glutathione conjugates: (a) TEM imaging of self-assemblies formed by TMT(AcOH)_2_(glutathione) (left), TMT(AcOH) (glutathione)_2_ (center), and TMT(glutathione)_3_ (right) in water, (b) CD spectra of aged glutathione conjugate solutions corresponding to TEM images above, and (c) particle size distributions based on DLS measurements counted according to the volume of particles: TMT(AcOH)_2_(glutathione)—blue, TMT(AcOH) (glutathione)_2_—red, TMT(glutathione)_3_—green.

As shown in Figure 4, two types of regular structures were observed by TEM in all cases, spherical assemblies and cross-linked sponge-like structures, but some important differences should be noted. We observed different types of spherical assemblies for TMT(AcOH) (glutathione)_2_ than for others, respectively, small hollow and large filled spheres (assuming by observation of concave structures of wrinkly collapsed or dehydrated assemblies). The individual filled large particles were about 500–1000 nm in size. However, they could merge, giving larger structures. In the case of TMT(AcOH) (glutathione)_2_, we observed some fraction of small particles with a diameter of about 50 nm and larger hollow spheres. Interestingly, the hollow spheres took intermediate sizes 100–200 nm, and they were observed only for TMT(AcOH) (glutathione)_2_, which additionally did not form the large filled spheres observed for both counterparts. In all cases, we observed a sponge-like structure, which seemed to be built by assembling of spherical or tubular particles with a small diameter. The spongy structures were very similar in the two first analogues, though the structure of TMT(AcOH) (glutathione)_2_ was less cross-linked than for TMT(AcOH)_2_(glutathione). The sponge of TMT(glutathione)_3_ was less contrasted, which suggests a looser internal structure and greater distances between molecules. These observations may be explained based on CD spectra. In the spectra of TMT(AcOH)_2_(glutathione) and TMT(AcOH) (glutathione)_2_, a positive exciton doublet at about 250 nm was probably caused by a kind of stacking of 1,3,5-triazine rings. The influence of interactions between triazine rings decreased with increasing steric crowding around the rings, up to triple glutathione substitution, preventing this type of interaction. This also explains the greater cross-linking of sponges formed by the compound with one glutathione chain than the doubly substituted compound. The CD spectrum of TMT(glutathione)_3_ was still strongly influenced by the triazine chromophore, but the negative band at 210–225 nm might be rather connected with structures formed by interactions between glutathione moieties.

Dynamic light scattering measurements appear to support the existence of similar structures in a solution. In all cases, two populations of particles are visible (Figures 4C, S140–S142). The smaller particles of about 100 nm (depending on the compound) are probably responsible for the assembly into spongy structures during TEM sample preparation. Interestingly, these structures appear to be repetitively ordered when observed by TEM. Such assembling may be associated with specific interactions between particles and may indicate a kind of ordering of molecules in the small spheres. These small spheres in solutions are observed as numerous populations with average particle size increasing in series TMT(AcOH)_2_(glutathione) < TMT(AcOH) (glutathione)_2_ < TMT(glutathione)_3_, with the maximum of abundance at about 50, 90, and 110 nm, respectively. The second population of particles for TMT(AcOH) (glutathione)_2_ is observed as the tailing of the peak corresponding to the main population. These larger particles correspond probably to the hollow spheres observed by TEM. In the other cases, the populations of large particles, larger than 200 nm up to 2 μm, are visible by DLS. They correspond probably to the large, filled spheres found by TEM. The maxima of abundance for these structures are about 500 and 800 nm for TMT(AcOH)_2_(glutathione) and TMT(glutathione)_3_, respectively. Very large, higher than 1 μm particles, probably form by merging large spheres into larger structures, which are also sometimes visible on TEM (Figures S138 and S139). Differences in particle sizes determined by TEM and DLS may be caused by the participation of solvent molecules associating and filling the spheres. Nevertheless, there is a reasonable correlation of particle size trends for samples tested with these two methods.

The relationship between the structure of the obtained compounds and the assembled aggregates is worth a more detailed description. The most interesting is a preference for TMT(AcOH) (glutathione)_2_ to form assemblies similar to the hollow spheres described by Matsuura et al.^8^ They observed that a steric hindrance next to his aromatic scaffold in trigonal conjugates of glutathione caused the formation of filled regular assemblies with the size of 310 ± 50 nm.^8b^ While a conformationally non-disturbed analogue formed hollow spheres with sizes 100–250 nm.^8a^ In our compounds, the peptide chains are closer to the aromatic scaffold than in the systems developed by Matsuura. Thus, TMT(glutathione)_3_ has probably a rigid structure with glutathione main chains twisted to the plane of the triazine ring, which promotes the formation of large, filled spheres. The ability of TMT(AcOH)_2_(glutathione) to form the large spherical assemblies indistinguishable from those created by TMT(glutathione)_3_ may be explained by assuming an initial formation of a non-covalent trimer by stacking of triazine moieties, followed by self-assembly of those trimers mimicking TMT(glutathione)_3_. On the other hand, the more flexible TMT(AcOH) (glutathione)_2_ forms smaller hollow spheres, which probably may be assembled faster and more easily than the large ones.

The mixture of TMT(AcOH)_3_ and glutathione is the first and the simplest example of a DCL based on transthioesterification of trialkyl thiocyanurates, wherein thermodynamic stability and a rate of self-assembly probably could be responsible for the selection of the main products. We believe that this technique may be used in the search for new viral capsid-like covalent organic frameworks and other supramolecular systems for nanotechnology.

### Thiocyanurates Metathesis

The second type of thioester reactivity potentially useful in dynamic covalent chemistry is thioester metathesis, which is possible by using an addition of a catalytical thiol forming transitionally active thioester by the first transthioesterification step. Then in the next thioesterification steps, the thiol formed from the original ester may attack the parent molecule or other thioester present in the solution, leading to metathesis.

Nowadays, the most commonly used catalysts of NCL or widely transthioesterification reactions are MESNa (sodium 2-mercaptoethylsulphonate)^9^ and MPAA (4-mercaptophenylacetic acid),^9b^ which we tested in our model systems.

We prepared tris(carboxamidomethyl) thiocyanurate, TMT(AcNH_2_)_3_, as a second thioester next to TMT(AcOH)_3_. The amide counterpart is poorly soluble, both in DMF and in aqueous solutions. During its synthesis by the alkylation of thiocyanuric acid with iodoacetamide, the white precipitate falls out of the reaction mixture and is suitable for further work immediately after filtration, washing with water, and drying.

**Figure 5:**
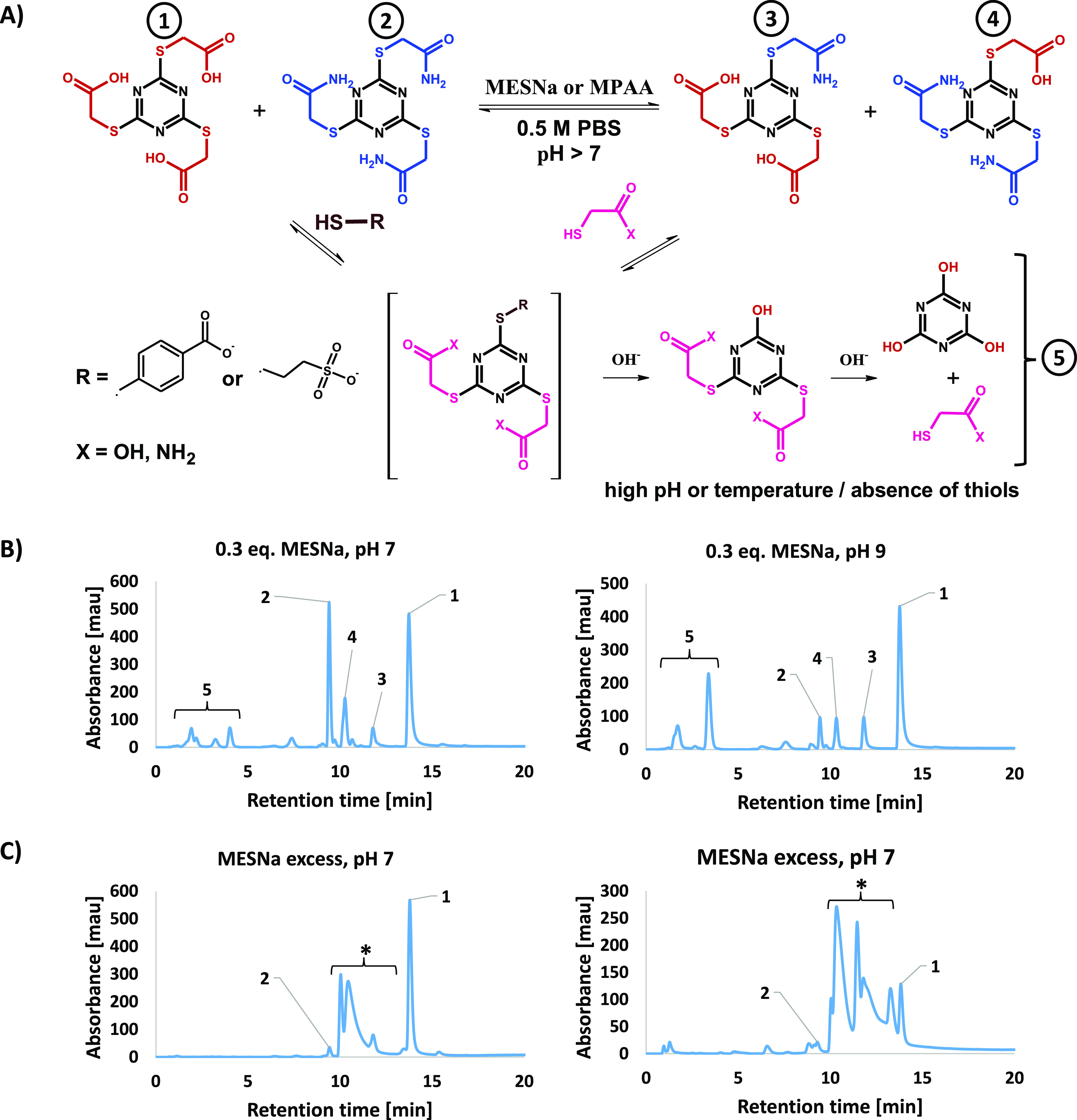
MESNa-catalyzed metathesis between tris(carboxamidomethyl) and tris(carboxymethyl) thiocyanurates. (A) Scheme of reaction pathways in the dynamic library of thiocyanurates, (B) RP-HPLC analysis at 254 nm after incubation of thiocyanurates with 0.3 equiv MESNa at 40 Celsius degrees, (C) RP-HPLC analysis of mixture obtained with a 10-fold excess of MESNa. All samples were analyzed after 24 h of incubation. * all possible thiocyanurates substituted by −AcOH, −AcNH_2_, −EtSO_3_H.

We prepared a mixture of TMT(AcOH)_3_ and TMT(AcNH_2_)_3_ by weighing the same amounts of both esters and dissolving in 10% AcOH obtaining about 5 mM concentration of both forms. The stock solution was divided into 1 mL aliquots in Eppendorf tubes, which were lyophilized and then suspended in 1 mL of 0.5 M phosphate buffer at pH 3, 7, and 9, containing 20 mM TCEP and 1.5 M MESNa or MPAA. After 24 h of vigorous mixing at room temperature or incubation at 40 Celsius degrees, all samples at pH 7 and 9 turned clear with a slightly yellow color, which was stronger in the case of pH 9. HPLC-PDA-MS analysis of the samples showed that at pH 7 and 9 all possible forms of thioesters are present (**1–4**, Figure 5A), while at pH 3 no progress of the reaction was observed. At higher pH, the metathesis is taken further, but significant progress in hydrolysis was also observed, in particular of more reactive TMT(AcNH_2_)_3_ (Figure 5B). Analyzing possible thiocyanurate forms, no significant differences were found in the composition of post-reaction mixtures obtained with the use of MESNa or MPAA as catalysts (detailed results available in the Supporting Information).

The amide TMT(AcNH_2_)_3_ is more reactive than the acid TMT(AcOH)_3_. Hydrolytic decay of thiocyanurates 1–4 depends clearly on the number of carboxylate groups close to the triazine ring, while TMT(AcOH)_3_ is the least susceptible molecule. It is well understood that proximity of the negative charge hampers a nucleophilic attack, but there is still one question remaining whether TMT(AcNH_2_)_3_ is hydrolyzing directly or after a substitution by MESNa or MPAA. To find the answer, we checked the stability of TMT(AcNH_2_)_3_ in the phosphate buffer at pH 9 and at room or elevated temperatures. In the absence of a catalyst, the triamide thiocyanurate remains stable even at elevated temperatures up to 70 Celsius degrees, similar to the acid counterpart. Our findings demonstrate that the active compound is formed by substituting at least one ligand by the catalyst, which may undergo hydrolysis resulting in reduced stability of the remaining thioester groups and the rapid decomposition of the entire molecule. We also performed additional experiments using a 10-fold excess of MESNa calculated for thiocyanurates collectively. Then during RP-HPLC analysis, we observed all possible forms including those containing ethyl-2-sulphonate substituents, and no traces of thiocyanurates hydrolysis were found (Figure 5C). We concluded that the reaction between thiocyanurates and mercaptans is chemoselective enough to prevent hydrolysis with a sufficient concentration of thiols.

A design of dynamic combinatorial libraries of thiocyanurates may have practical implications, but care should be taken to use an excess of thiols when conducting reactions in a mild alkaline aqueous medium. The simplest way to produce such libraries is mixing a symmetrical thiocyanurate with a 3-fold excess of each mercaptan included in the library in the presence of a reducing reagent like TCEP. When thinking about designing useful systems, the transthioesterification reaction rather than the metathesis itself should be considered. Nonetheless, all 2,4,6-tris(alkylsulfanyl)-1,3,5-triazines have similar absorption spectra with a characteristic maximum at 250–260 nm which makes libraries of such compounds easy to analyze by HPLC with UV detection.

### DFT Calculations of Thiocyanurates Reactivity Descriptors

To better understand the differences in reactivity of the types of triazines discussed in this article, we performed a series of DFT calculations at the B2-PLYP level of theory with the RI approximation for the perturbation step and RIJCOSX for the SCF step.^10^ A polarized triple-zeta def2-TZVP basis set was used. Geometry optimization was performed for neutral compounds using a conductor-like polymerizable continuum model of water implemented in ORCA 5.0.4.^11^ Geometries of N-electron states were used in single-point calculations of N-2e, N-1e, and N+1e electron densities. Output data were analyzed in Multiwfn 3.8^12^ using conceptual DFT tools^13^ (all calculated properties are available in the Supporting Information).

**Figure 6:**
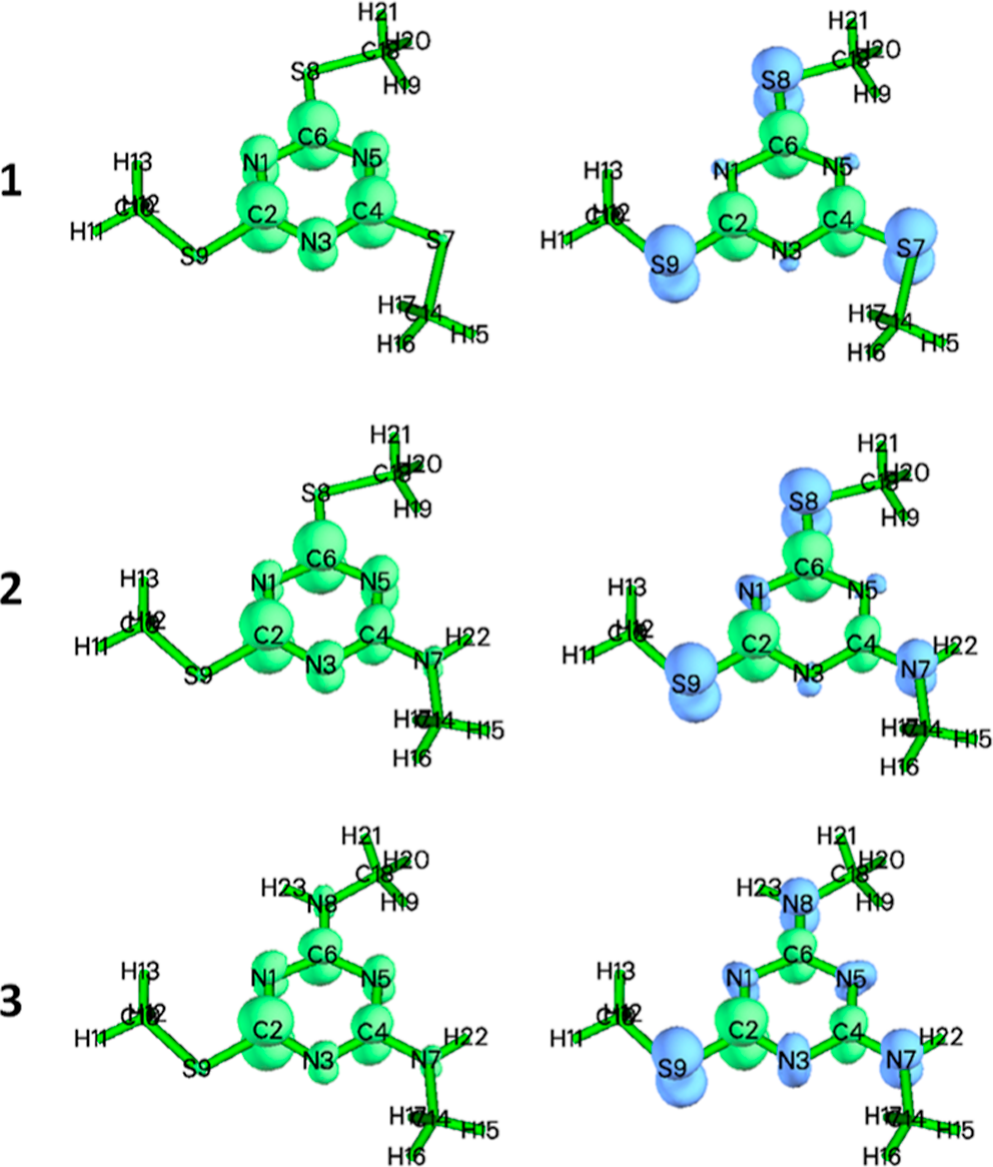
Isosurfaces of orbital weighted Fukui functions F^+^ (left) and dual descriptors (right) of (1) TMT(Me)_3_, (2) DMT(Me)_2_(methylamine), and (3) MMT(Me) (methylamine)_2_. Isovalue 0.005.

Orbital weighted Fukui functions and dual descriptors (Figure 6) were calculated due to a partial degeneration of HOMO and LUMO orbitals.

Through the analysis of Fukui functions, condensed Fukui indexes (Table 1), and dual descriptors, it is clear that the centers susceptible to the attack of the nucleophile are only carbon atoms linked by sulfur atoms. Simultaneously with the progressive replacement of sulfur atoms with NH groups, the reactivity of the molecule toward nucleophiles, expressed as the electrophilicity index, gradually decreases (electrophilicity index 1.6575, 1.4468, and 1333 eV for TMT, DMT, and MMT, respectively). However, there is also a decrease in the aromatic character of the triazine ring in the TMT > DMT > MMT series (HOMA aromatic index 0.9911 > 0.9810> 0.9785). While the changes in most properties are successive, the hardness and softness of TMT and DMT remain almost the same, and the change in these properties starts for MMT (the hardness of the molecule increases). It seems that the electrophilic centers of TMT and DMT are reactive toward a similar type of nucleophiles according to the hard and soft (Lewis) acids and bases (HSAB) theory, which could explain the experimentally observed reactivity.

**Table 1:**
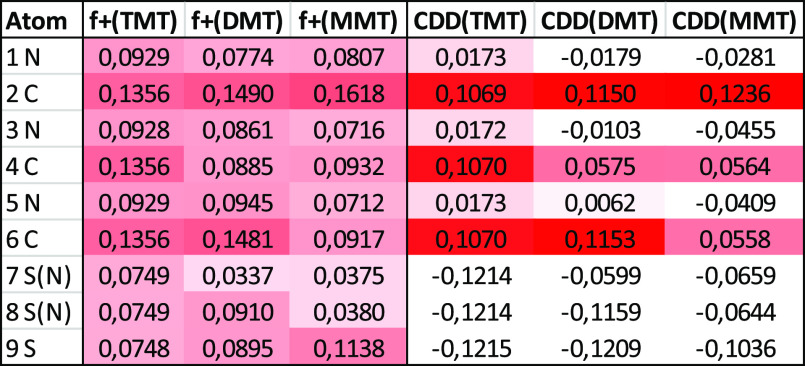
Condensed Orbital Weighted Fukui Indexes f^+^ and Dual Descriptors CDD for Thiocyanurate Units of TMT, DMT, and MMT Scaffold

## Conclusions

Investigating the synthesis of thiocyanuric acid–peptide conjugates, we found and have described new reactivity of alkyl thiocyanurates toward thiols at mild basic conditions in TEAB or PBS buffers. Alkyl esters of thiocyanuric acid show a reactivity very similar to that of carboxylic acid thioesters, making transthioesterification and NCL mode transformations possible within the aromatic 1,3,5-triazine ring.

Reactivity of trialkyl thiocyanurates toward 2-aminothiols via transthioesterification followed by the S to N 1,3,5-triazin-2-yl migration is generally limited to a monosubstitution at room temperature or a disubstitution at elevated temperatures in mild basic aqueous solutions. This limitation was explained based on the conceptual DFT analysis of hardness and softness of model trisubstituted 1,3,5-triazines, showing that tristhiocyanurates and monoamide dithiocyanurates should be reactive toward similar types of soft nucleophiles, while monothiocyanurate diamides are generally less reactive due to the increase in their hardness. Nonetheless, the ligation of cysteamines, including conjugations of cysteinyl-peptides with esters of thiocyanuric acid, may be of practical importance due to their high chemoselectivity and efficiency, e.g., in designing new derivatization agents.

On the other hand, transthioesterification of trialkyl thiocyanurates in the presence of an excess of thiols is very selective and as a reversible process is very promising from the point of view of applications in dynamic covalent chemistry. Two examples were used to demonstrate the possibility of generating dynamic libraries of compounds through substitution of the sulfanyl ligand in TMT(AcOH)_3_ with AcCysOH or glutathione. Since the reaction equilibria with glutathione seemed to be influenced by some intramolecular interactions, we decided to analyze the preferences for self-aggregation of the parent products. We found that TMT(AcOH) (glutathione)_2_ can form hollow nanospheres with a size of 100–200 nm in contrast to larger filled spheres with a size of 500–1000 nm formed by mono- and trisubstituted counterparts. Similar systems based on the tiocyanurate metathesis can potentially be useful in the selection of self-aggregating artificial capsid-like particles for nanotechnology.^8^

We also investigated the thiocyanurates metathesis reaction. Application of this approach using the same catalysts as for carboxylic acid thioesters is limited due to the susceptibility of transiently formed active thioesters to hydrolysis. However, the excess of thiols effectively protects against hydrolysis. Thus, the easiest way to create dynamic systems is the mixing of a symmetrical thiocyanurate with an appropriate excess of an equimolar mixture of compounds containing free sulfhydryl groups.

The thiocyanurate-based dynamic covalent chemistry has some advantages in comparison to other known trigonal systems based on thiol-disulfides exchange^8a,14^ or Schiff bases formation.^15^ Compared to systems based on disulfide chemistry, the thiocyanurate scaffold cannot react with itself, making such libraries predictable and easy in routine analysis. We can expect a tetrahedral number  of thiocyanurates (due to trigonal symmetry) accompanied by *N*^2^ dimeric disulfides, where *N* is the number of thiols used in the library. In addition, it is conceivable to remove unreacted thiols by using a solid support with attached reactive moieties as maleimidyl substituents that can selectively react with sulfhydryl groups. This additional step could be very effective in simplifying the analysis of final libraries. Systems based on Shiff bases could be even more complex and do not require additional purification. However, the transthioesterification of thiocyanurates has other and probably wider orthogonality toward many functionalities (e.g., it could be used in the presence of amine groups). Another advantage of the system based on tranthioesterification of thiocyanurates is that it could be simply quenched by acidification, giving us full control over the progress of the reaction, whereas freezing of the Shiff base equilibrium usually requires an additional reduction. We believe that our reaction could be used in a rational design of organic supramolecular architectures similar to those formed from tripodal Shiff bases or thiols (e.g., covalent organic frameworks, molecular cages).

## Data Availability

The data underlying this study are available in the published article and its Supporting Information.

## Supporting Information Available

Experimental procedures, characteristics of synthesized compounds, chromatograms, NMR and MS spectra, summarized DFT results, additional TEM images, and DLS results (PDF)
Compressed catalogue containing final geometries as name. XYZ files, summarized calculated properties as name_property.txt files, and electron densities as name.eldens.cube files (ZIP)
