# Supplementary material for: Alkyl Thiocyanurates as Thioester Mimetics. Transthioesterification and Ligation Reactions with High Potential in Dynamic Covalent Chemistry
Source: J Org Chem. 2023 Jun 17;88(13):8192–202. doi: 10.1021/acs.joc.3c00200 (PMC10337033; doi:10.1021/acs.joc.3c00200)
Supplement: Supplementary file 1 — jo3c00200_si_001.pdf [file jo3c00200_si_001.pdf]

Alkyl thiocyanurates as thioester mimetics. Transthioesterification and ligation reactions with high potential in dynamic covalent chemistry.

Grzegorz Wołczański*, Wojciech Gil, Jakub Cichos, Marek Lisowski, Piotr Stefanowicz

Faculty of Chemsitry, University of Wrocław, F. Joliot-Curie 14, 50-383 Wrocław, Lower Silesia District, Poland

*grzegorz.wolczanski2@uwr.edu.pl

Table of Contents

1. Materials and methods. General descriptions. S2

1.1. Chemicals S2

1.2. Solid phase peptide synthesis S2

1.3. ESI-MS and LC-MS analysis S2

1.4. HPLC analysis and purification S3

1.5. NMR spectroscopy S3

1.6. Circular dichroism and absorption spectra in near ultraviolet spectroscopy S3

1.7. TEM imaging S3

1.8. DLS measurements S3

1.9. Theoretical calculations S3

2. Synthesis of substrates for ligation and transthioesterification. S4

2.1. Esters of thiocyanuric acid S4

2.2. Solid-phase synthesis of cysteinyl peptides S11

3. Thermal and pH-dependent stability of thiocyanurates S15

4. Transthioestrification and ligation reactions S18

4.1. Reactions with cysteamines S18

4.1.1. Ligation of cysteinylpeptides to [(4,6-disulfanyl-1,3,5-triazin-2-yl)sulfanyl]acetyl-βAla-Lys-OH S18

4.1.2. Reaction of tris(carboxymethyl)thiocyanurate with the cysteamines (2-mercaptoethylamines) S25

4.1.3. Reaction of tris(carboxymethyl)thiocyanurate with cysteine S38

4.1.4. Reaction of tris(carboxymethyl)thiocyanurate with penicillamine S44

4.1.5. Reaction tris(carboxymethyl)thiocyanurate with *D*-cysteine S50

4.1. Reactions of tris(carboxymethyl)thiocyanurate with sulphides S52

4.1.1. Reaction with Ac-Cys-OH S52

4.1.2. Reaction with glutathione S62

5. Self-assembly of TMT(AcOH)$_X$(Glutathione)$_Y$ S78

5.1. TEM images S78

5.2. DLS measurements S89

5.3. CD spectra S90

6. The metathesis reaction of tris(carboxymethyl)thiocyanurate and tris(carboxamidomethyl)thiocyanurate S91
6.1. Influence of different pH and temperatures S92
6.2. Influence of the excess of a catalyst S101
7. DFT calculations S108
7.1. Conceptual DFT in Multiwfn S108

1. Materials and methods. General descriptions.

1.1. Chemicals

All commercially available reagents were used without further purification, and water was deionized by the reverse osmosis system (Hydrolab, Poland). Solvents for building block synthesis and peptide synthesis are as follows: chloroform (stabilized with amylene), $CDCl_3$; dimethylformamide, DMF; dichloromethane, DCM; methanol, MeOH; tetrahydrofuran, THF; diethyl ether, Et_2O; *N*,*N*-diisopropylethylamine, DIPEA; piperidine, PIP; HCOOH; trifluoroacetic acid, TFA; triisopropylsilane, TIS; and 1,2-ethanedithiol, EDT. Solvents in the analytical grade were obtained from Sigma-Aldrich, and Fmoc-amino acid derivatives for peptide synthesis were purchased from PeptideWeb. The coupling reagent PyBop, benzotriazole-1-yl-oxytripyrrolidinophosphonium hexafluorophosphate was purchased from Navoabiochem, *N*,*N′*-diisopropylcarbodiimide (DIC) was purchased from Fluka. The resin for SPPS - H-Rink amide ChemMatrix resin (0.40–0.60 mmol/g) was purchased from Sigma-Aldrich. Reagents for the synthesis of thiocyanurats bromoacetic acid, iodoacetamiande, and thiocyanuric acid were purchased from Sigma-Aldrich. Reagents for ligation and transthioesterification reactions, sodium 2-mercaptoethanesulfonate (MESNa), 4-mercaptophenylacetic acid (MPAA), tris(2-carboxyethyl)phosphine hydrochloride (TCEP x HCl), 1M triethylammonium bicarbonate buffer (TEAB, pH 8.5) were purchased from Sigma-Aldrich. Other chemicals, including hydrated disodium phosphate, and hydrated monosodium phosphate, were purchased from AppliChem, NaOH was purchased from Stanlab, and analytical grade HCl was purchased from Sigma-Aldrich. Solvents for LC-MS are as follows: MeCN and HCOOH in HPLC grade were purchased from Sigma-Aldrich, and MeOH and HPLC grade H_2O in HPLC were purchased from J. T. Baker. All deuterated solvents for NMR measurements were purchased from Sigma-Aldrich.

1.2. Solid phase peptide synthesis

Peptides synthesized on solid support were prepared according to the ultrasonic synthesis protocol recently reported by Wołczański *et all.* (DOI: 10.1016/j.tetlet.2019.05.069). The peptide cleavage was performed by incubation of the resin with 90/5/2.5/2.5 TFA/EDT/TIS/water mixture, followed by precipitation of the product in cold diethyl ether, centrifugation, and lyophilization from water.

1.3. ESI-MS and LC-MS analysis

The direct injection ESI-MS experiments were performed on a Fourier transform ion cyclotron resonance (FT-ICR) Apex-Qe Ultra 7T mass spectrometer (Brukerltonics, Bremen, Germany) equipped with a standard ESI source. The FT-ICR instrument was operated in the positive-ion mode and calibrated with the Tunemix™ mixture (Agilent Technologies, Palo Alto, CA, USA) following a quadratic method. The samples were prepared by dissolving 0.1 mg of the analytes in 1 mL of 0.1% formic acid in 50% aqueous acetonitrile. Analyte solutions (150 μL) were introduced at a flow rate of 3 μL/min. The instrument parameters were as follows: drying gas: nitrogen; flow rate: 1.5 L/min; temperature: 200°C; potential between the spray needle and the orifice: 4.5 kV.

The LC-MS experiments were performed on an LC-UV-IT-TOF instrument, which is a hybrid system consisting of a liquid chromatograph, aD detector, an ion trap, and a time-of-flight mass analyzer. In both instruments, CID fragmentation (with Argon) was used, and the potential between the spray needle and the orifice was set to 4.5 kV. The LC systems were operated with the following mobile phases: A = 0.1% HCOOH in H_2O and B = 0.1% HCOOH in MeCN in a gradient separation in a gradient separation mode at a 0.2 mL/min flow rate and a 2-10 μL injection. The IT-TOF instrument was calibrated using the IT-TOF adjustment standard sample (Shimadzu ®) containing trifluoroacetic salts in aqueous acetonitrile.

1.4. HPLC analysis and purification

Two analytical HPLC systems were used. The analyses of Cys-peptides and their DMT-analogues were performed on Thermo Separation with C18 column (Microsorb-MW 100-5 C-18 250x4.6 mm) and UV detector – samples analyzed at 220 nm. Most analyses were performed on the Nexera XR HPLC system (Shimadzu) with various C-18 columns (details in the description of analyticalta). The second HPLC system is equipped with the SPD-M20AD detector and connected to IT-TOF mass spectrometer (Shimadzu).

Purification of compounds was performed on the semi-preparative Varian ProStar HPLC system equipped with the TOSOH Bioscience TSKgel ODS 120T column (21.5 mm x 300 mm; 10µm), typically using the following solvent systems: 1%–70% B in A for 40 min, A 0.1% aqueous TFA, B 80% acetonitrile + 0.1% TFA, flow rate 7.0 ml/min, UV detection at 220 and 280 nm. The fractions obtained after separation were collected and lyophilized.

1.5. NMR spectroscopy

NMR measurements for compound characterization were performed at 300K on Bruker Avance III 500 MHz with a double resonance broadband probe (5 mm, $^{1}H/^{2}H/^{31}P-^{109}Ag$, Z-grad, ATMA). The measured compounds were at a concentration of 5-15 mM. Analysis of obtained spectra was performed using ACD/NMR Processor Academic Edition software. Structural assignments were made with additional information from gCOSY, gHSQC, and gHMBC experiments

1.6. Circular dichroism and absorption spectra in near ultraviolet spectroscopy

Self-assembly of glutathione conjugates was investigated using circular dichroism spectroscopy at the range of wavelength 190-350 nm. Electron circular dichroism spectra in near UV were recorded at 25°C on Jasco J-600 circular dichroism spectrometer with a temperature control accessory. Solvents used in sample preparation were HPLC gradient grade or spectroscopically pure. A path length of 1 mm was used. The concentrations of the solutions were in the range of 0.15–0.27 mM. Each spectrum represents the average of at least 20 scans. Theta are presented as molar ellipticity.

1.7. TEM imaging

Measurements were carried out using an FEI Tecnai G^2 20 X-TWIN transmission electron microscope equipped with an electron gun with a LaB_6 cathode, an FEI Eagle 2K CCD camera, and a HAADF detector for the STEM scanning and transmission technique. Samples in colloidal form were loaded onto EMS USA UL-50 mesh 400 copper grids with a carbon film 3 - 4 nm thick. It was then dried for about 30 min in the air at room temperature. After drying, the samples were contrasted for 50 s with a 2% $UO_2(OAc)_2$ solution. Imaging was performed at an accelerating voltage of 200 kV. EDS spectra were recorded with an EDAX detector. The parameters declared by the producer: TEM point resolution: 0.25 nm; TEM linear resolution: 0.102 nm; STEM resolution: 1.0 nm; a range of diffraction angles: ± 12°; minimum sharpness adjustment step: 1.8 nm; magnification range: 22 - 930 000 x; maximum sample slope: 30 °. Analysis of images was performed in HyperSpy software.

1.8. DLS measurements

The size of particles in solution was determined by dynamic light scattering (DLS) using a Zetasizer Nano ZS instrument (Malvern Instruments Ltd, Malvern, Worcestershire, UK). All measurements were performed at 25°C using disposable sizing cuvettes and distilled water as solvent.

1.9. Theoretical calculations

DFT calculations were performed in the ORCA 5.0.4 software. Inputta were prepared in the Avogadro software. Geometry optimization calculations of the N-electron states were performed on the P2BLYP level of theory with RI approximation for the perturbation step and RIJCOSX for the SCF step. Single point DFT calculations of N-1e and N+1e states were performed on the same level of theory using coordinates for N-electron states. Outputta were analyzed using Multiwfn software using conceptual DFT tools. All calculations were performed on DELL PC equipped with Intel® Xeon® E-2124G CPU @ (8GT/s; 4 cores, 3.40 GHz, turbo 4.50 GHz), and 32GB RAM.

2. Synthesis of substrates for ligation and transthioesterification.

2.1. Esters of thiocyanuric acid

Tris(carboxymetyl)thiocyanurate - TMT(AcOH)$_3$

Synthesis: A solution of 352 mg of thiocyanuric acid (2 mmol) in 10 mL of water was alkalized with a solution of 480 mg sodium hydroxide (12 mmol) in a minimal amount of water at room temperature. Then the solution was cooled in an ice bath, followed by the addition of 970 mg of bromoacetic acid (7 mmol). The reaction mixture was left on a magnetic stirrer at room temperature for 3h. Next, the solution was acidified to pH about 2 with HCl followed by immediate precipitation of white solids. The suspension was left in a fridge for full crystallization. After 24 hours, white precipitate was collected by filtration and dried in a vacuum over phosphorus pentoxide. Yield 0.54 g (77%).

HPLC: 13.8 min (column – Aeris 3.6 µm PEPTIDE XB-C18 50 x 2.1 mm; detection -D, 256 nm; gradient - 1%B for 5 min, 10-30%B 6-20 min, 30-100%B 21-25 min; flowrate 0.2 mL/min; eluent A – 0.1% formic acid in water, eluent B – 0.1% formic acid in acetonitrile)

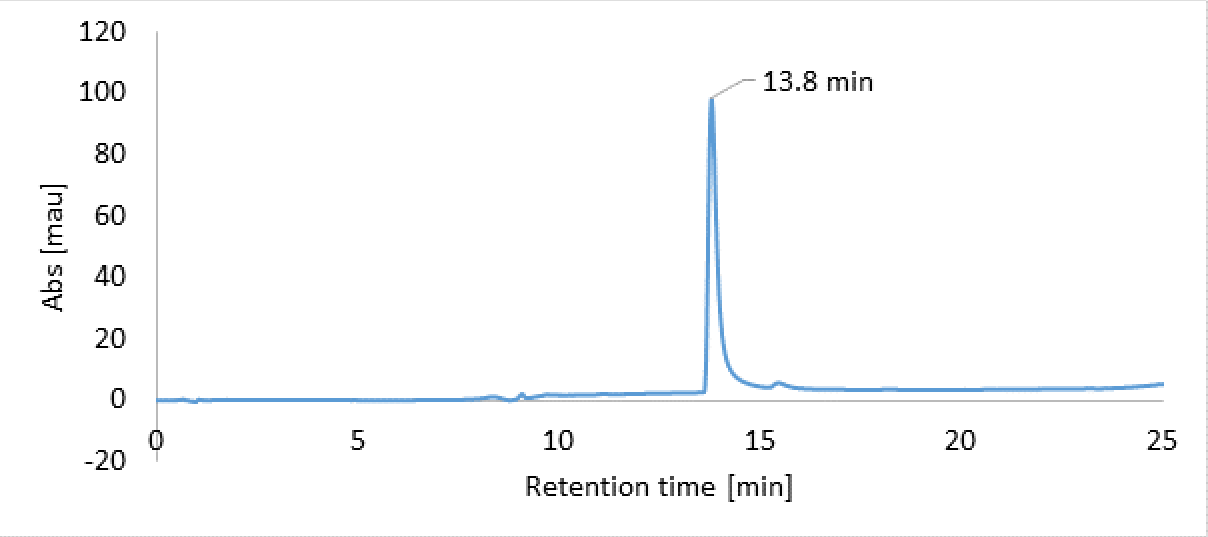

Figure S1. RP-HPLC chromatogram of TMT(AcOH)$_3$

HRMS (ESI-MS) m/z: $[M-H_2O+H]^+$ Calcd for $C_9H_9N_3O_5S_3$ 333.9626; Found 333.9580, $[M+H]^+$ Calcd for $C_9H_{10}N_3O_6S_3$ 351.9726; Found 351.9644, $[M+Na]^+$ Calcd for $C_9H_9N_3O_6S_3Na$ 373.9546; Found 373.9543, $[M+K]^+$ Calcd for $C_9H_9N_3O_6S_3K$ 389.9285; Found 389.9297, $[2M+Na]^+$ Calcd for $C_{18}H_{18}N_6O_{12}S_6Na$ 724.9199; Found 724.9194.

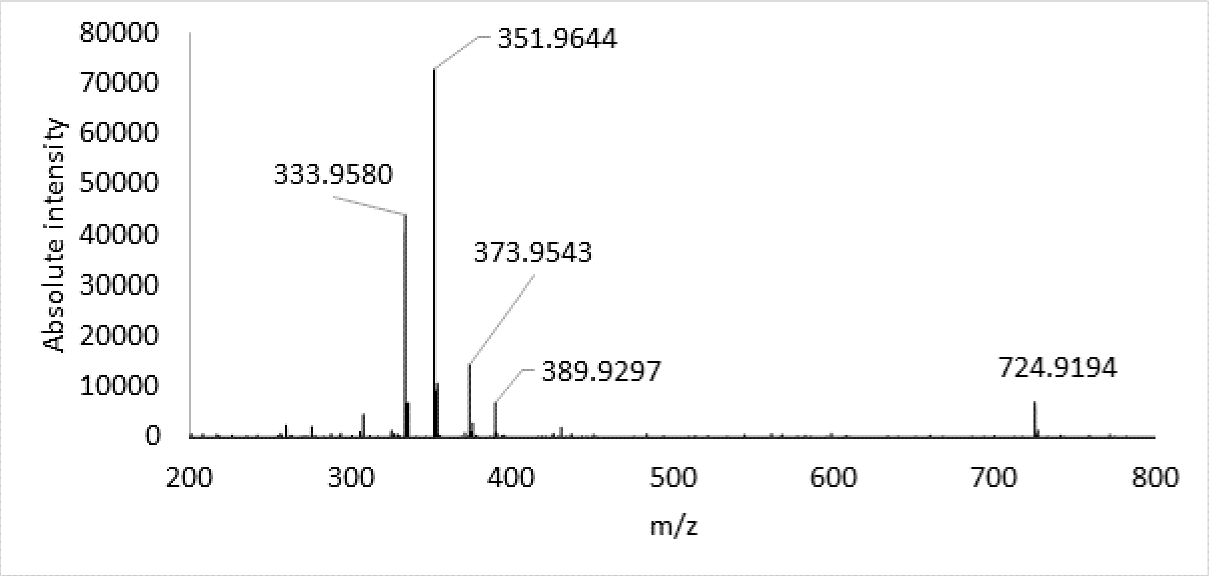

Figure S2. ESI-MS spectrum of TMT(AcOH)$_3$ in positive ion mode.

1H NMR (DMSO-d6, 500 MHz, 25°C) δ (ppm) = 3.95 (s, 6H, CH_2 SAcOH)

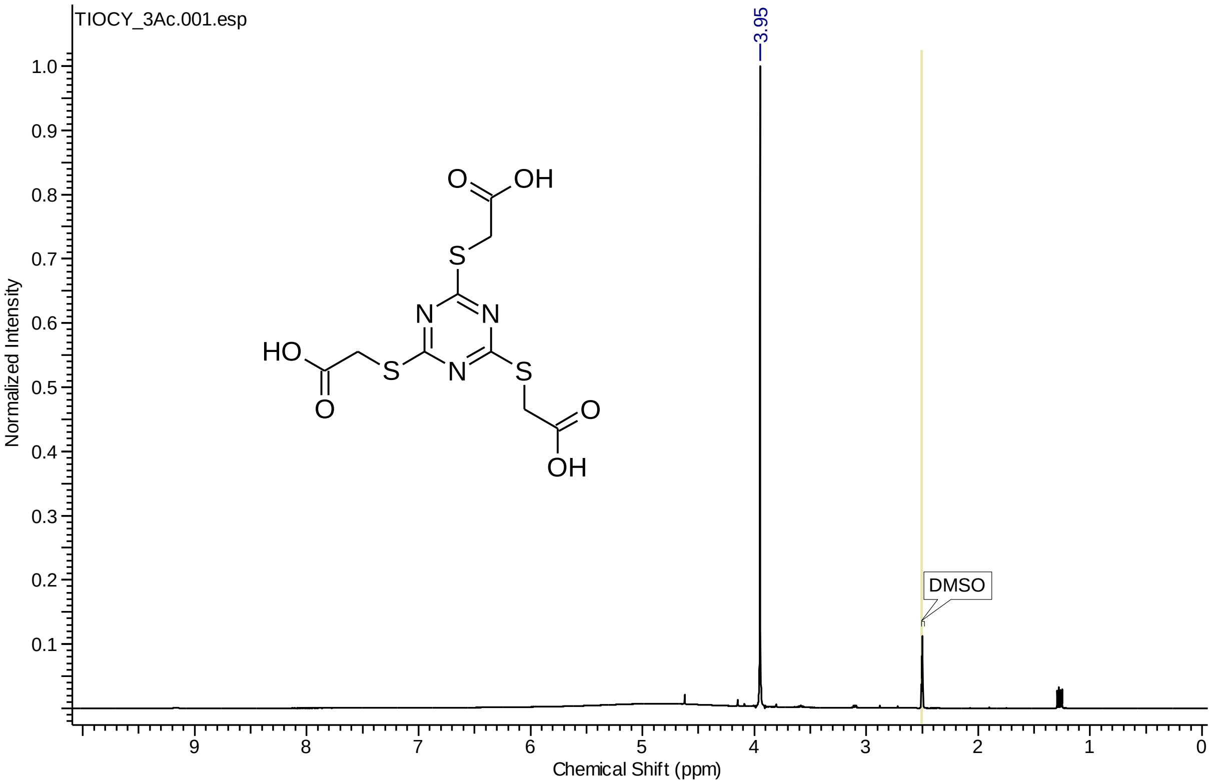

Figure S3. 1H NMR spectrum of TMT(AcOH)$_3$ in DMSO-d$_6$

13C{H} NMR (DMSO-d6, 125 MHz, 25°C) δ (ppm) = 178.5 (triazine), 169.4 (C(O), SAcOH), 32.7 (CH_2, SAcOH)

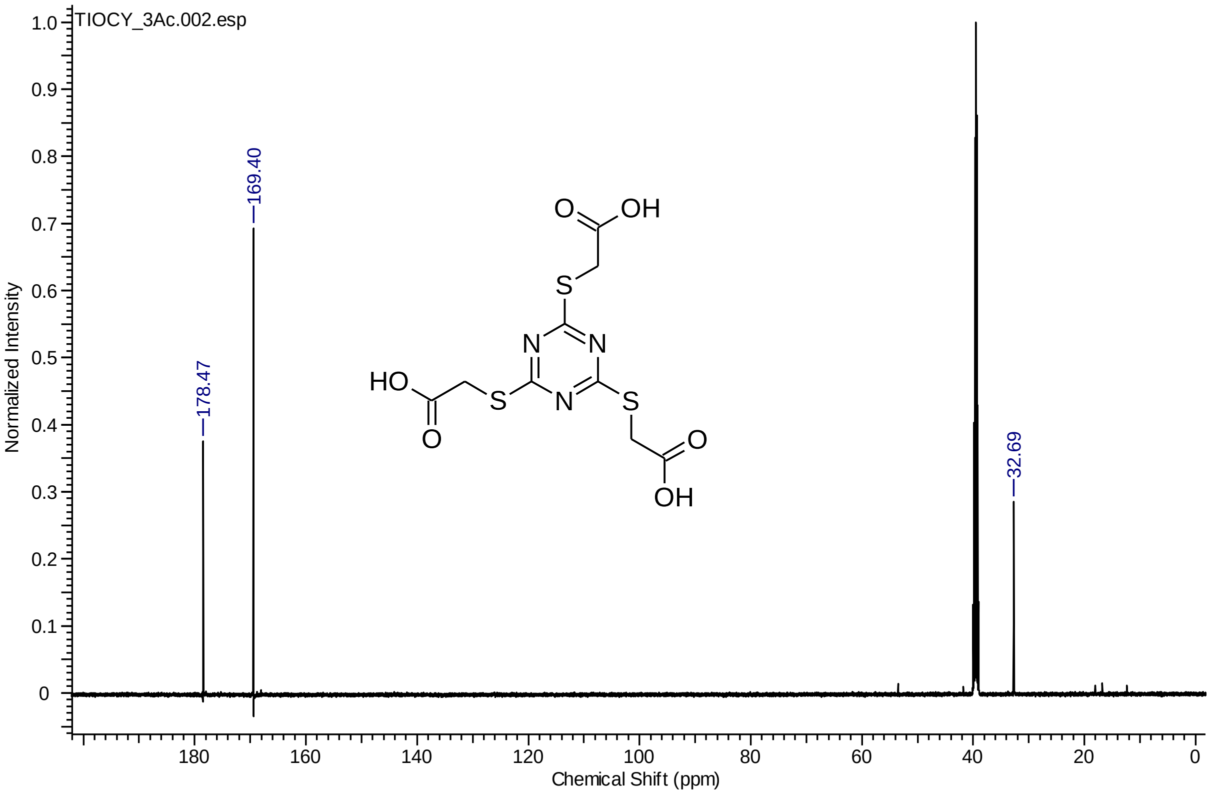

Figure S4. 13C{H} NMR spectrum of TMT(AcOH)$_3$ in DMSO-d$_6$

Tris(caboxamidomethyl)thiocyanurate - TMT(AcNH$_2$)$_3$

Synthesis: 1.7 mL of triethylamine (12 mmol) was added to a solution of 352 mg of thiocyanuric acid (2 mmol) in 5 mL of DMF. Resulted solution was filtered through a syringe filter with 0.2µm PES membrane, followed by the addition of 1.29 g of iodoacetamide (7 mmol). The reaction mixture was left on a magnetic stirrer for 2 hours in arkness. A white amorphous solid precipitated in the reaction mixture. The reaction mixture was diluted with water to 50 mL of the volume. The precipitated product was centrifuged in a falcon tube, washed two times with water, and lyophilized. Yield 0.65 g (93%).

HPLC: 9.1 min (column – Aeris 3.6 µm PEPTIDE XB-C18 50 x 2.1 mm; detection -D, 256 nm; gradient - 1%B for 5 min, 10-30%B 6-20 min, 30-100%B 21-25 min; flowrate 0.2 mL/min; eluent A – 0.1% formic acid in water, eluent B – 0.1% formic acid in acetonitrile)

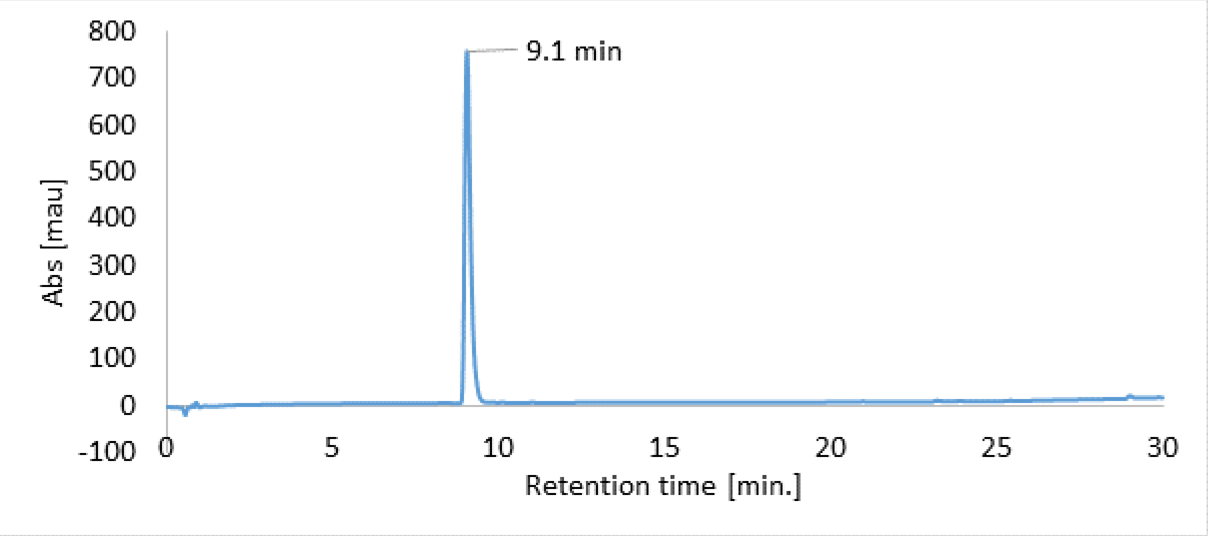

Figure S5. RP-HPLC chromatogram of TMT(AcNH$_2$)$_3$

HRMS (ESI-MS) m/z: [M-NH$_3$+H]$^+$ Calcd for $C_9H_{11}N_5O_3S_3$ 331.9946; Found 331.9996, [M+H]$^+$ Calcd for $C_9H_{13}N_6O_3S_3$ 349.0206; Found 349.0117, [M+Na]$^+$ Calcd for $C_9H_{12}N_6O_3S_3Na$ 371.0025; Found 371.0004, [M+K]$^+$ Calcd for $C_9H_{12}N_6O_3S_3K$ 386.9765; Found 386.9683, [2M+Na]$^+$ Calcd for $C_{18}H_{24}N_{12}O_6S_6Na$ 719.0158; Found 719.0012.

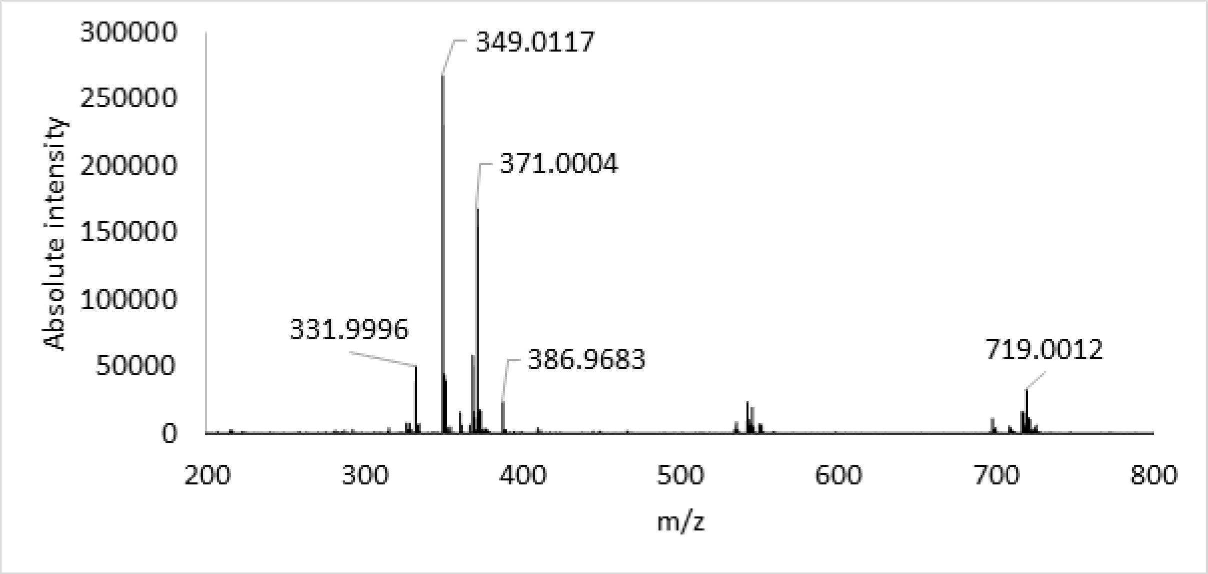

Figure S6. ESI-MS spectrum of TMT(AcNH$_2$)$_3$ in positive ion mode.

1H NMR (DMSO-d6, 500 MHz, 25°C) δ (ppm) = 7.24 (s, 3H, *cis*-NH), 7.61 (s, 3H, *trans*-NH), 3.95 (s, 6H, HA#, SAcNH$_2$)

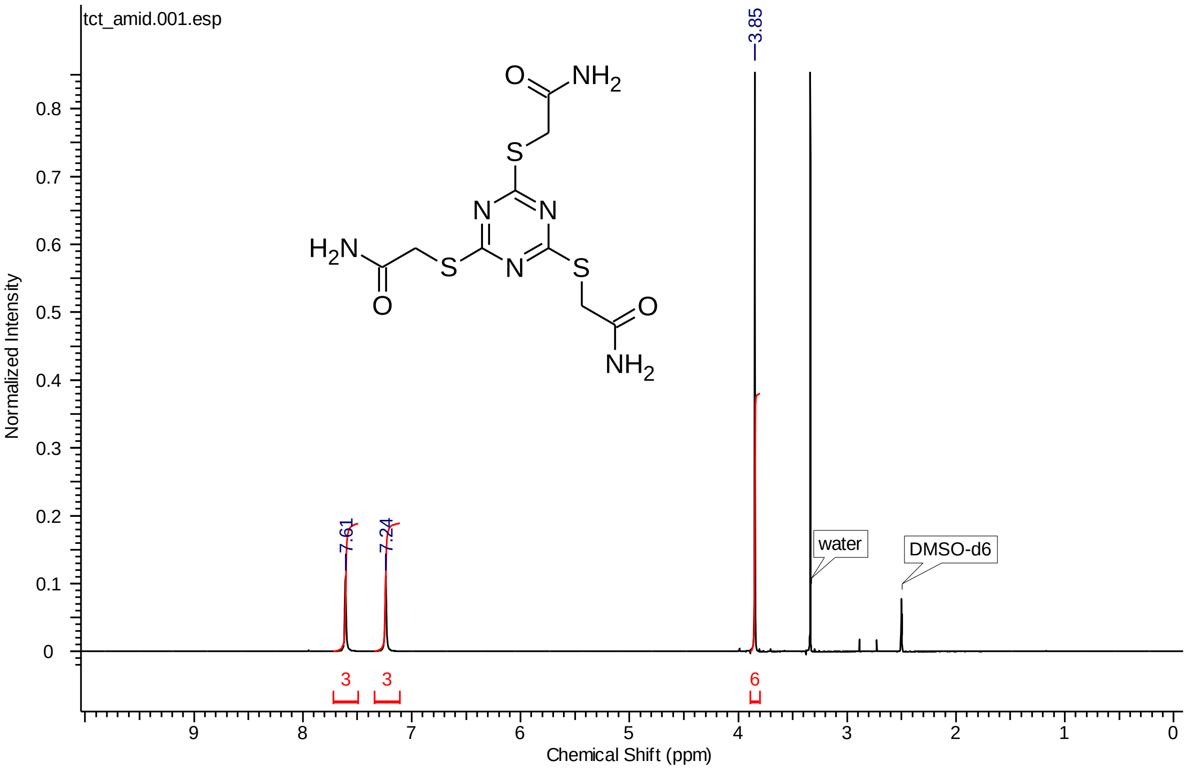

Figure S7. 1H NMR spectrum of TMT(AcNH$_2$)$_3$ in DMSO-d$_6$

13C{H} NMR (DMSO-d6, 125 MHz, 25°C) δ (ppm) = 178.5 (triazine), 168.6 (C(O), SAcNH$_2$), 34.0 (CH$_2$, SAcNH$_2$)

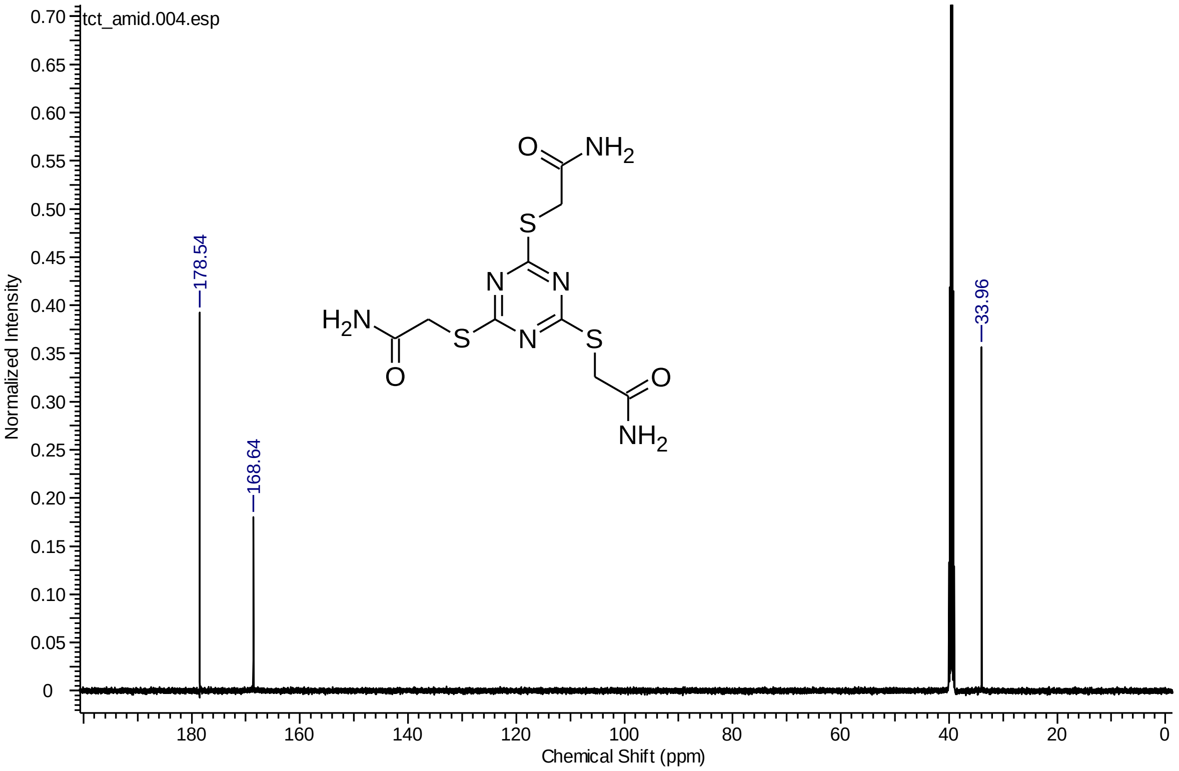

Figure S8. 13C{H} NMR spectrum of TMT(AcNH$_2$)$_3$ in DMSO-d$_6$

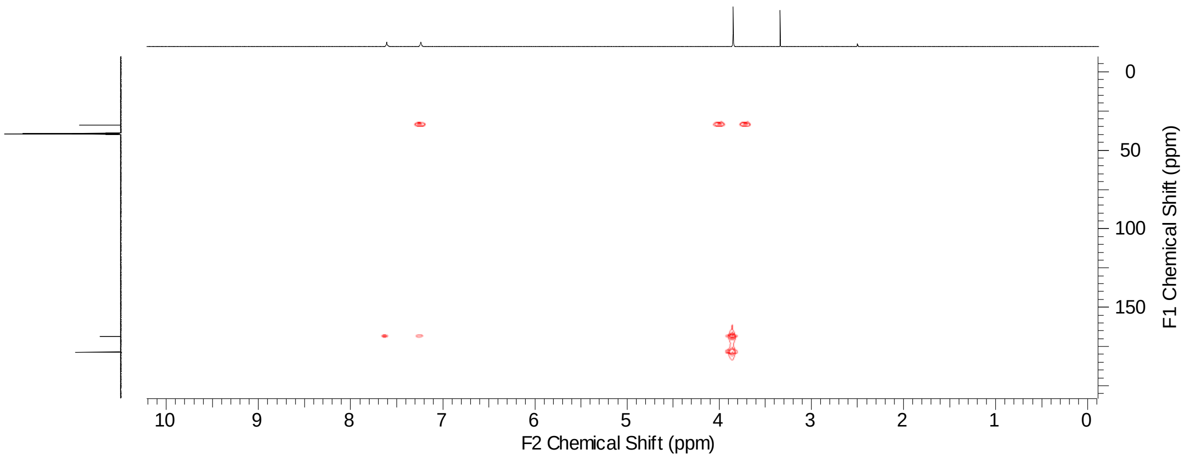

Figure S9. HMBC spectrum of TMT(AcNH$_2$)$_3$. The correlation of amide protons with carbonyl carbon atoms supports the assignments above

N-[*S*-(4,6-disulfanyl-1,3,5-triazin-2-yl)sulfanyl]acetyl-βAla-Lys-OH (abbreviated TMT-Ac-βAla-Lys-OH)

Synthesis: The solid phase step of the peptide synthesis was performed on 200 mg of preloaded Fmoc-Lys(Boc)-Wang resin with declared loading 0.6 mmol/g (0.12 mmol). The H-βAla-Lys-O-Wang peptidyl resin was synthesized according to the general method (p.1.2). After that the bromoacetyl moiety has been introduced using a mixture of 85 mg bromoacetic acid (5 equiv., 0.6 mmol) and 94µL of *N,N'*-diisopropylcarbodiimide (5 equiv., 0.6 mmol) in 1mL DMF for 1h. Then the resin has been washed with DMF, DCM, diethyl ether, and again DCM, and after that treated without drying with 95/5 TFA/TIS mixture to cleave the bromoacetylated product from the resin. The eluate was evaporated under nitrogen to dryness. The crude bromoacetylpeptide was dissolved in DMF and mixed with 64 mg of thiocyanuric acid (3 equiv., 0.36 mmol) and 9 eq. of 192 mg of *N,N*-diisopropylethylamine (9 equiv., 1.1 mmol). Next, the solvent was evaporated again under a nitrogen stream overnight. The crude product has been purified on the RP-HPLC semi-preparative Varian ProStar system, using the TOSOH Bioscience C18 column.

HPLC: 11.3 min (sample preparation: 1 mg of the peptide dissolved in 1 mL of eluent A; column – Microsorb-MW 100-5 C-18, 250x4.6 mm; detection – UV, 220 nm; gradient – 0-80%B in 40 min; flowrate 1 mL/min; eluent A – 0.1% TFA in water, eluent B – 0.1% TFA in 80% acetonitrile)

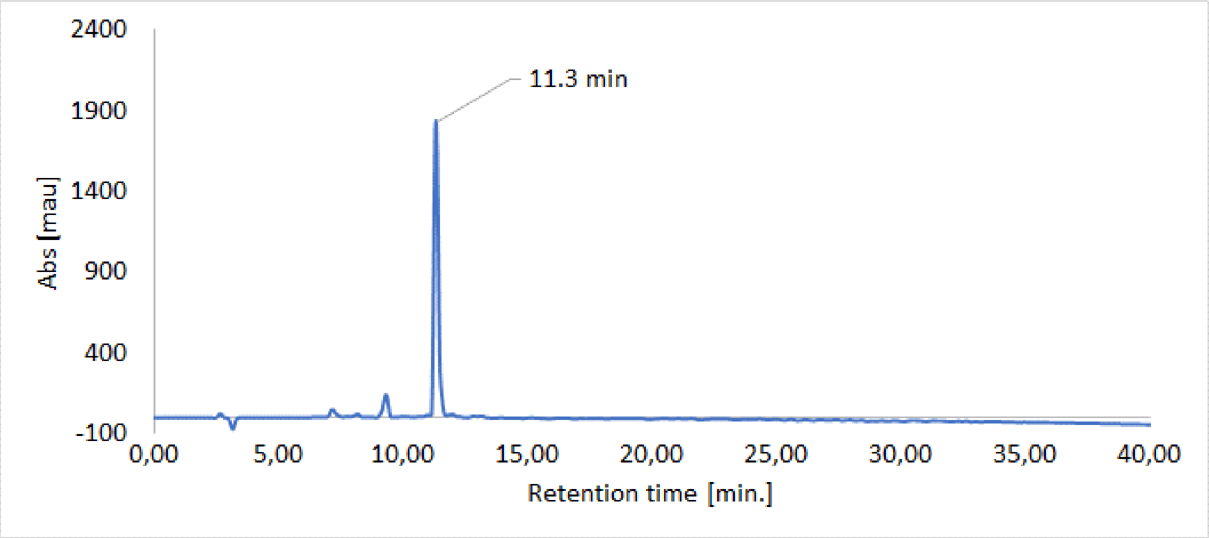

Figure S10. RP-HPLC chromatogram of TMT-AcβAlaLys.

HRMS (ESI-MS) m/z: $[M+H]^+$ Calcd for $C_{14}H_{23}N_6O_4S_3$ 435.0937; Found 435.0925, $[2M+H]^+$ Calcd for $C_{28}H_{45}N_{12}O_8S_6$ 869.1802; Found 869.1724, $[3M+H]^+$ Calcd for $C_{42}H_{67}N_{18}O_{12}S_9$ 1303.2667; Found 1303.2456

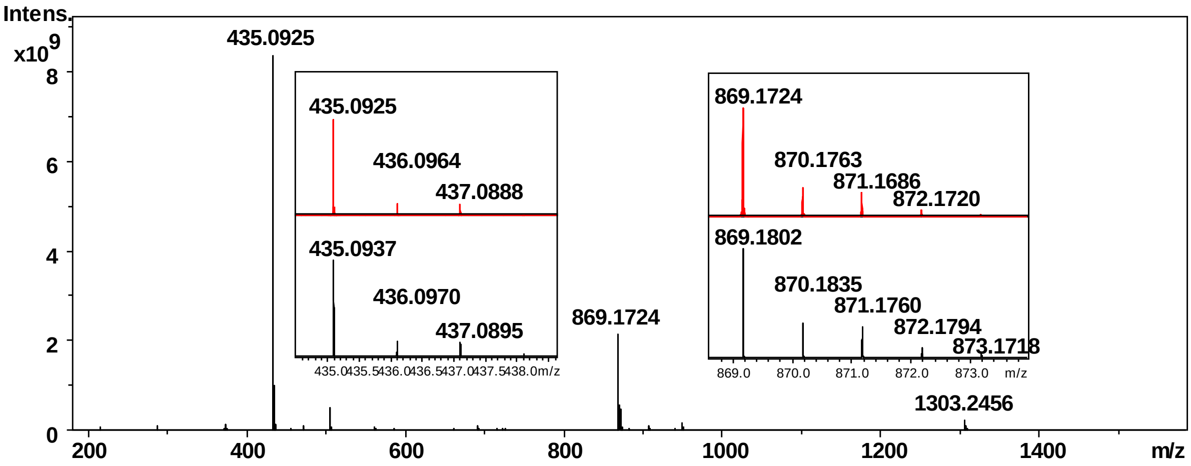

Figure S11. ESI-MS spectrum of TMT-Ac-βAla-Lys-OH in positive ion mode.

This compound slowly decomposes under aqueous conditions, including HPLC purification. The monothiocyanurate was further used without any additional purification and an analytic (p. 4.1.1.).

2.2. Solid-phase synthesis of cysteinyl peptides

Peptides were synthesized according to the general method (p.1.2). Purified peptides were characterized by ESI-MS spectrometry, including tandem method with CID fragmentation. Products were purified using Varian ProStar HPLC system equipped with the TOSOH Bioscience TSKgel ODS 120T column (21.5 mm x 300 mm; 10μm), typically using the following solvent systems: 1%–70% B in A for 40 min, A 0.1% aqueous TFA, B 80% acetonitrile + 0.1% TFA, flow rate 7.0 ml/min, UV detection at 220 and 340 nm. Collected fractions were characterized by direct ESI-MS, and lyophilized. The purity of the products was confirmed by HPLC with UV detection at 220 nm.

H-Cys-βAla-Leu-Glu-Ala-Leu-Lys-NH_2 (H-Cys-βAla-LEALK-NH_2)

HPLC: 13.2 min (sample preparation: 1 mg of the peptide dissolved in 1 mL of eluent A; column – Microsorb-MW 100-5 C-18, 250x4.6 mm; detection – UV, 220 nm; gradient – 0-80%B in 40 min; flowrate 1 mL/min; eluent A – 0.1% TFA in water, eluent B – 0.1% TFA in 80% acetonitrile)

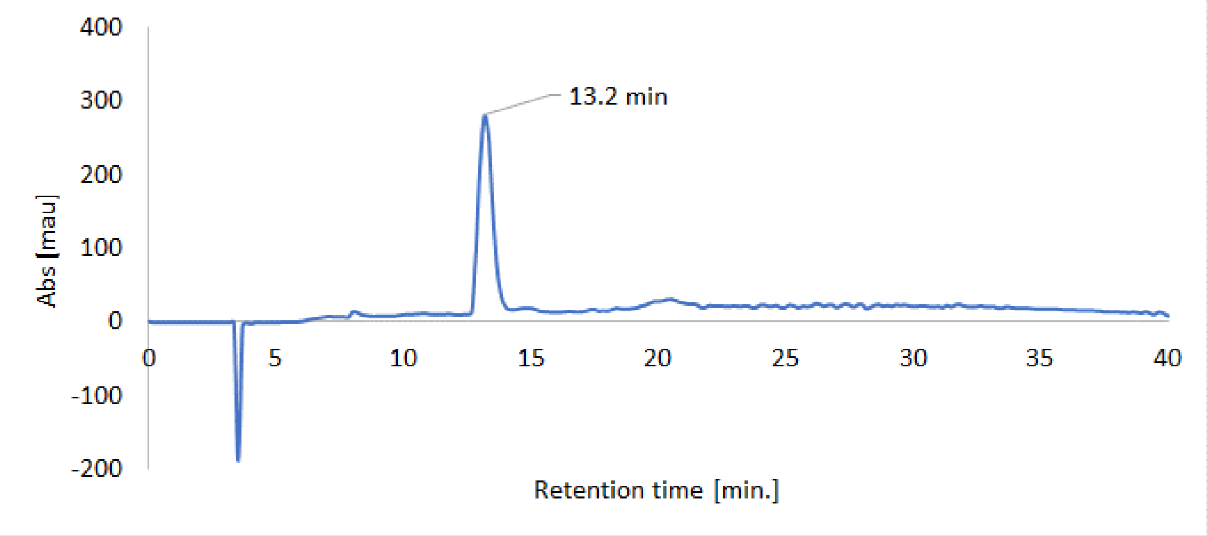

Figure S12. RP-HPLC chromatogram of H-Cys-βAla-LEALK-NH_2

HRMS (ESI-MS) m/z: $[M+H]^+$ Calcd for $C_{32}H_{60}N_9O_9S$ 746.4229; Found 746.4221, $[2M+H]^+$ Calcd for $C_{64}H_{119}N_{18}O_{18}S_2$ 1491.8386; Found 1491.8360

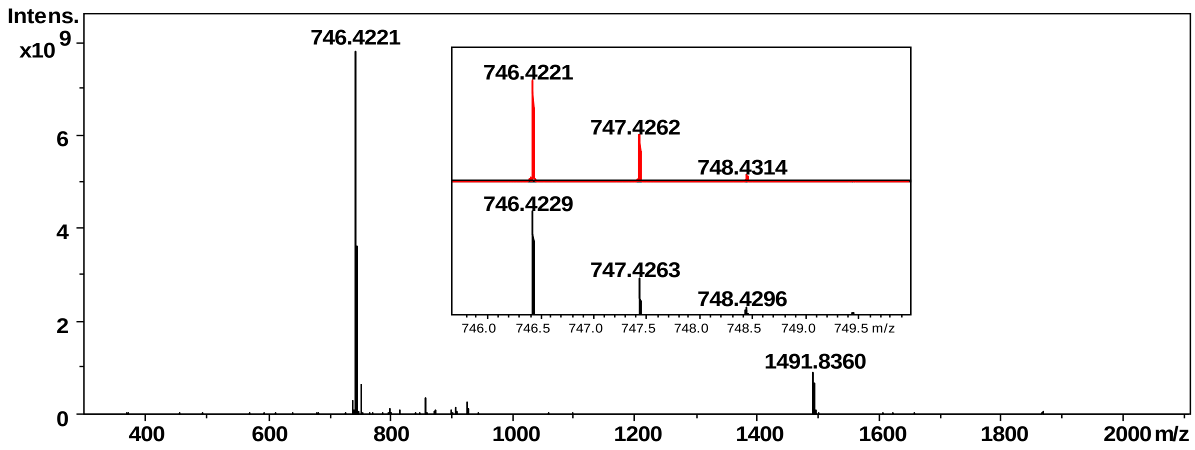

Figure S13. ESI-MS spectrum of H-Cys-βAla-LEALK-NH_2 in positive ion mode

ESI-MS(+)/MS, m/z: 729.3985 (b_7, Calcd 729.3964), 601.3032 (b_6, Calcd 601.3014), 583.2926 ($[b_6-H_2O]^+$, Calcd 583.2908), 573.3084 (a_6, Calcd 573.3065), 556.2799 ($a_6(z_7)$, Calcd 556.2799), 538.2717 ($[a_6(z_7)-H_2O]^+$, Calcd 538.2694), 470.2085 ($[b_5-H_2O]^+$, Calcd 470.2068), 460.2245 (a_5, Calcd 460.2224), 442.2137 ($[a_5-H_2O]^+$, Calcd 442.2119Da), 417.1821 (b_4, Calcd 417.1802), 399.1714 ($[b_4-H_2O]^+$, Calcd 399.1697), 385.2098 ($b_4(y_6)$, Calcd 385.2082), 330.2514 (y_3, Calcd 330.2500)

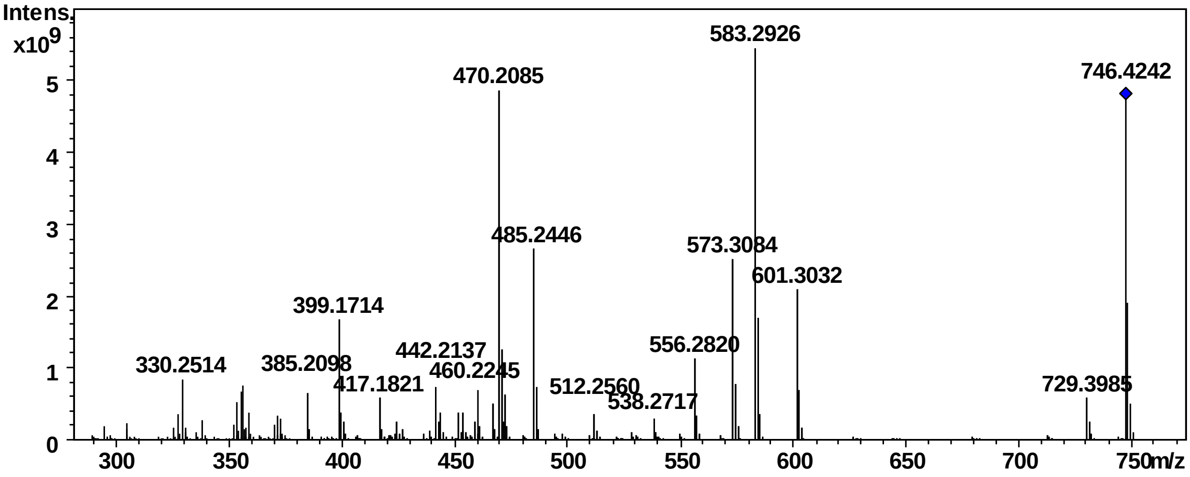

Figure S14. CID fragmentation MS[2] spectrum of H-Cys-βAla-LEALK-NH₂, [M+H]⁺ parent ion

H-Cys-βAla-Lys-Ala-Leu-Glu-Lys-Ala-Leu-Lys-Glu-Ala-Leu-Ala-Lys-Leu-NH_2
(H-Cys-βAla-KALEKALKEALAKL-NH_2)

HPLC: 28.2 min (sample preparation: 1 mg of the peptide dissolved in 1 mL of eluent A; column – Microsorb-MW 100-5 C-18, 250x4.6 mm; detection – UV, 220 nm; gradient – 0-80%B in 40 min; flowrate 1 mL/min; eluent A – 0.1% TFA in water, eluent B – 0.1% TFA in 80% acetonitrile)

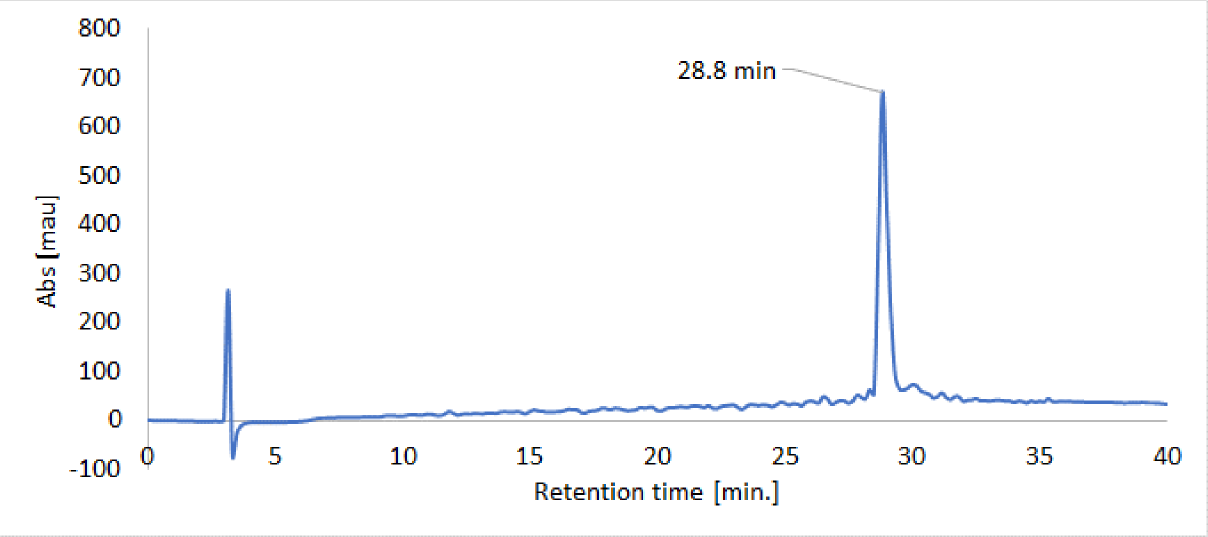

Figure S15. RP-HPLC chromatogram of H-Cys-βAla-KALEKALKEALAKL-NH_2

HRMS (ESI-MS) m/z: $[M+3H]^{3+}$ Calcd $C_{76}H_{142}N_{21}O_{20}S$ for 567.0148; Found 567.0163, $[M+2H]^{2+}$ Calcd for $C_{76}H_{141}N_{21}O_{20}S$ 850.0186; Found 850.0152, $[M+H]^{+}$ Calcd for $C_{76}H_{140}N_{21}O_{20}S$ 1699.0299; Found 1699.0220

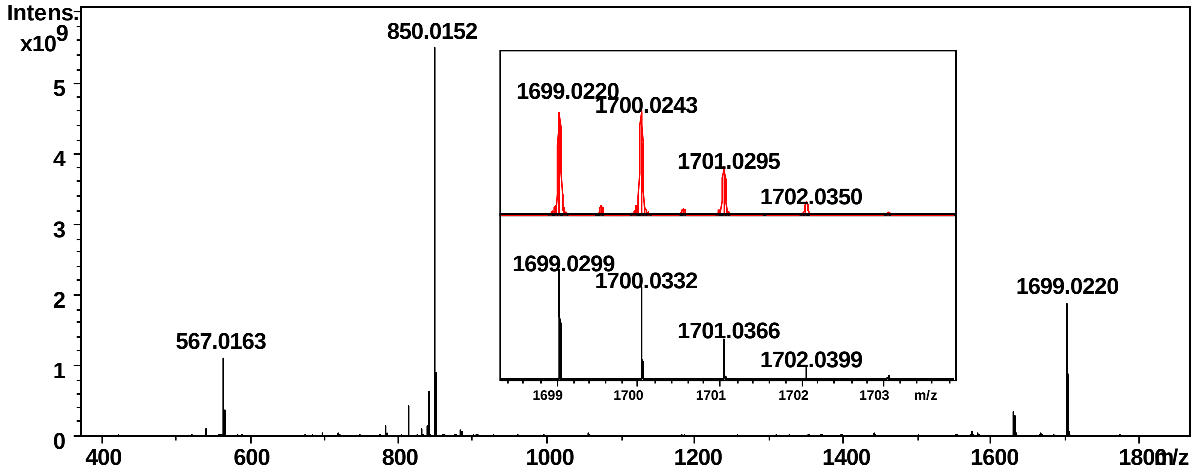

Figure S16. ESI-MS spectrum of H-Cys-βAla-KALEKALKEALAKL-NH_2 in positive ion mode

ESI-MS(+)/MS, m/z: 1681.9704 ([M-H_2O+H]$^+$, Calcd 1681.0199), 1663.9627 ([b_{16}-H_2O]$^+$, 1663.9933), 1568.8897 (b_{15}, Calcd 1568.9198), 1440.8007 (b_{14}, Calcd 1440.8243), 1422.7937 ([b_{14}-H_2O]$^+$, Calcd 1422.8137), 1369.7678 (b_{13}, Calcd 1369.7872), 1256.6858 (b_{12}, Calcd 1256.7031), 1185.6508 (b_{11}, Calcd 1185.6660), 1167.6424 ([b_{11}-H_2O]$^+$, Calcd 1167.6554), 1066.6885 (y_{10}(b_{16}), Calcd 1066.6983), 1056.6138 (b_{10}, Calcd 1056.6234), 938.5959 (y_9(b_{16}), Calcd 938.6033), 928.5210 (b_9, Calcd 928.5285), 910.5113 ([b_9-H_2O]$^+$, Calcd 910.5179), 825.5145 (b_8(y_9), Calcd 825.5193), 815.4398 (b_8, Calcd 815.4444), 797.4293 ([b_8-H_2O]$^+$, Calcd 797.4338), 754.4786 (y_7(b_{16}), Calcd 754.4822), 744.4038 (b_7, Calcd 744.4073), 697.4216 (b_6(y_7), Calcd 641.3981), 626.3853 (y_6(b_{16}), Calcd 626.3872), 616.3107 (b_6, Calcd 616.3123), 608.3749 ([y_6(b_{16})-H_2O]$^+$, Calcd 608.3766), 598.3004 ([b_6-H_2O]$^+$, Calcd 598.3017), 513.3026 (b_5(y_6), Calcd 513.3031), 495.2919 ([b_5(y_6)-H_2O]$^+$, Calcd 495.2926), 487.2692 (b_5, Calcd 487.2697), 442.2658 (b_4(y_7), Calcd 442.2660), 424.2555 ([b_4-H_2O]$^+$,Calcd 424.2554)

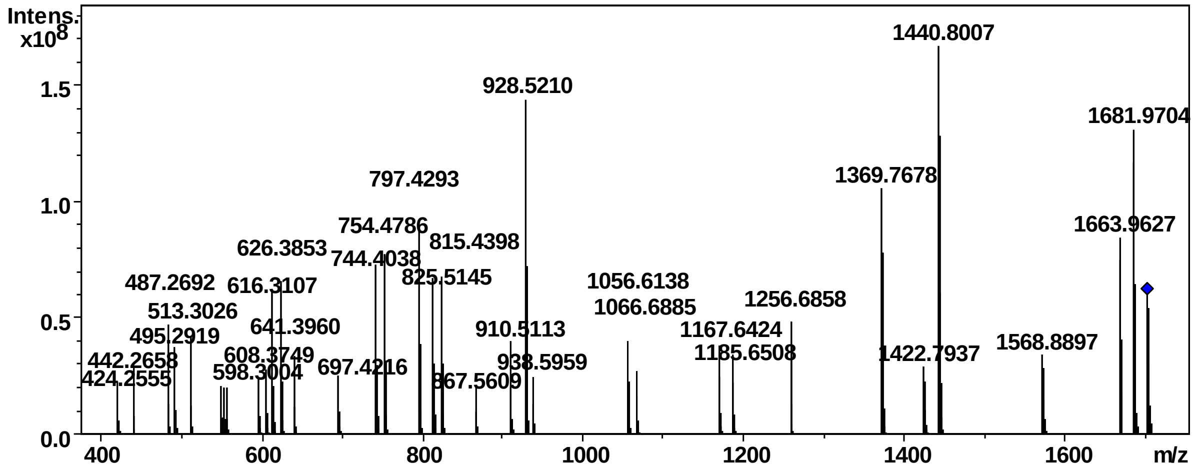

Figure S17. CID fragmentation MS^2 spectrum of H-Cys-βAla-KALEKALKEALAKL-NH_2, [M+H]$^+$ parent ion.

3. Thermal and pH-dependent stability of thiocyanurates

The stock solution of TMT(AcOH)$_3$ was prepared by dissolving 10 mg of the thiocyanurate in 2 mL water 1mL of water. 50 uL aliquots of the stock solution were diluted to 1mL by adding 950 uL of phosphate buffer solution adjusted to pH 5, 6, 8, 10, or 11. Obtained samples were divided into two portions placed in HPLC vials, which were incubated at 40 or 70 Celsius degrees in an oven for 24 hours. Samples weranalyzeded by HPLC with UV detection at 255 nm – before and after incubations.

HPLC: column – ReproSil-XR 120 C18-MS 3 µm, 100 x 2 mm; detection -D, 256 nm and ESI-MS (IT-TOF Shimadzu); gradient – 1-70%B in 15 min; eluent A – 0.1% formic acid in the water, flowrate 0.2 mL/min; eluent B – 0.1% formic acid in acetonitrile

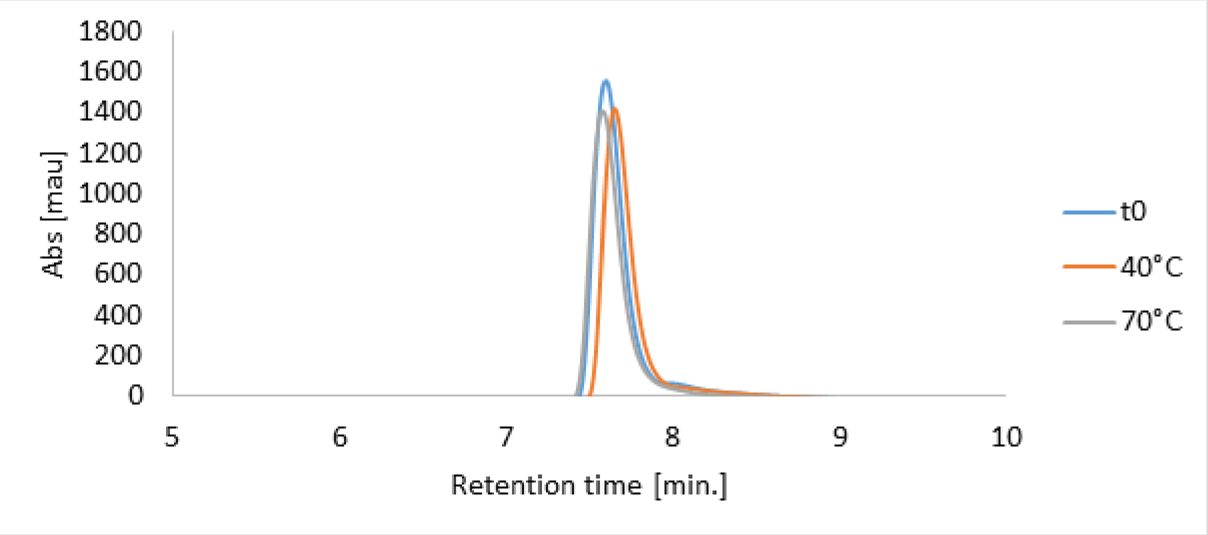

Figure S18. Thermal stability of TMT(AcOH)$_3$ at pH 5

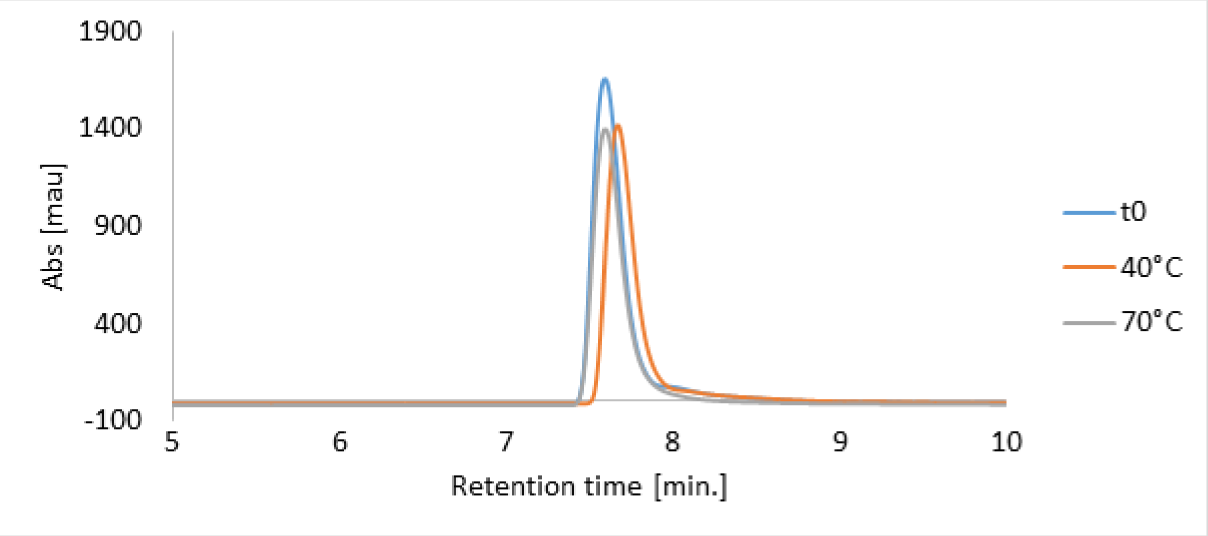

Figure S19. Thermal stability of TMT(AcOH)$_3$ at pH 6

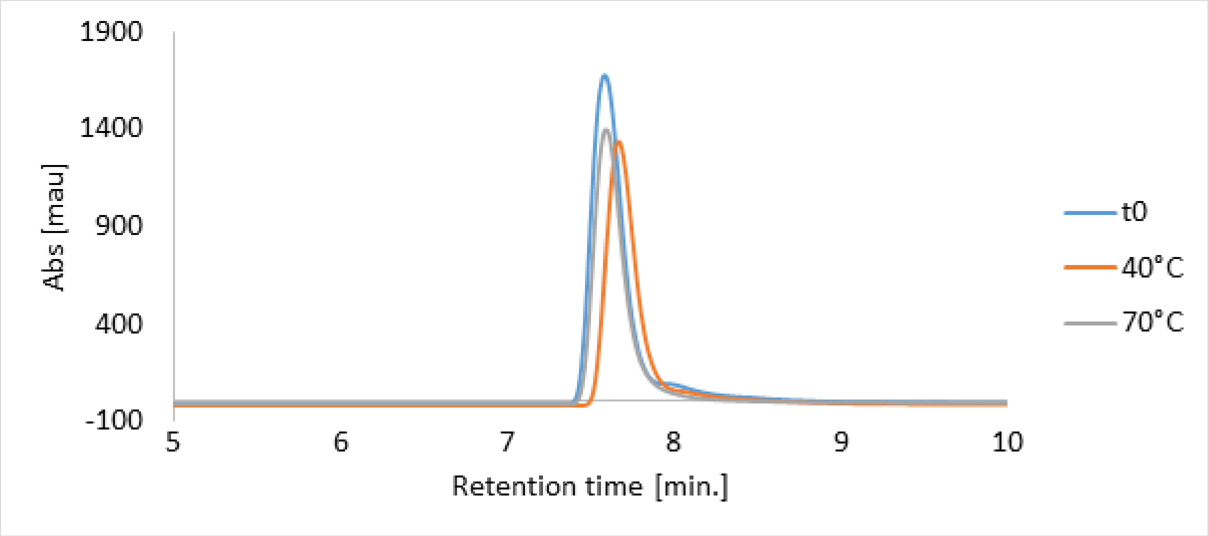

Figure S20. Thermal stability of TMT(AcOH)$_3$ at pH 8

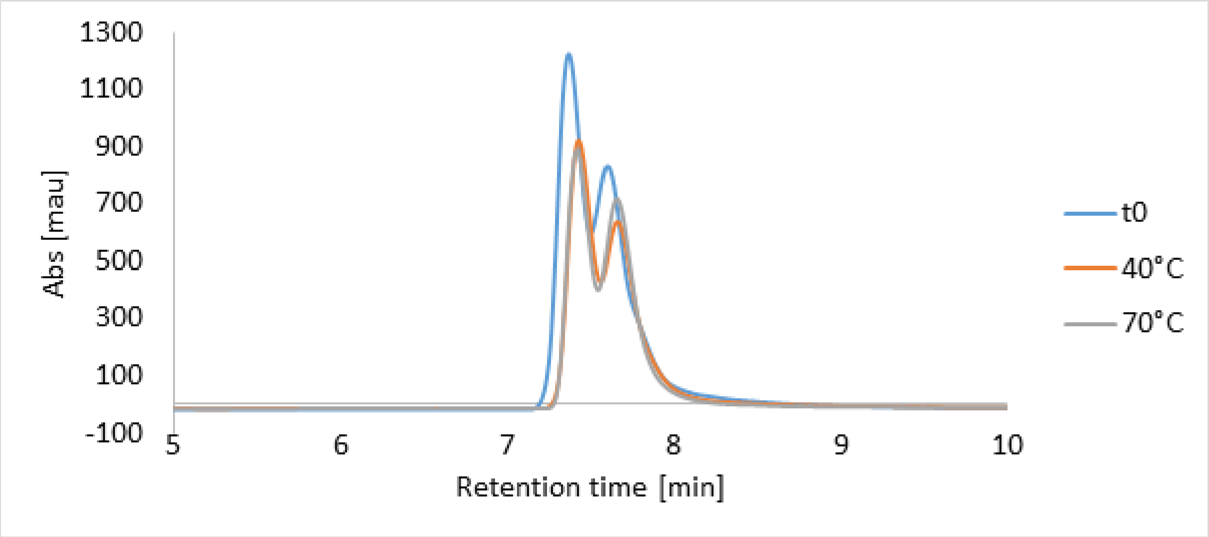

Figure S21. Thermal stability of TMT(AcOH)3 at pH 10

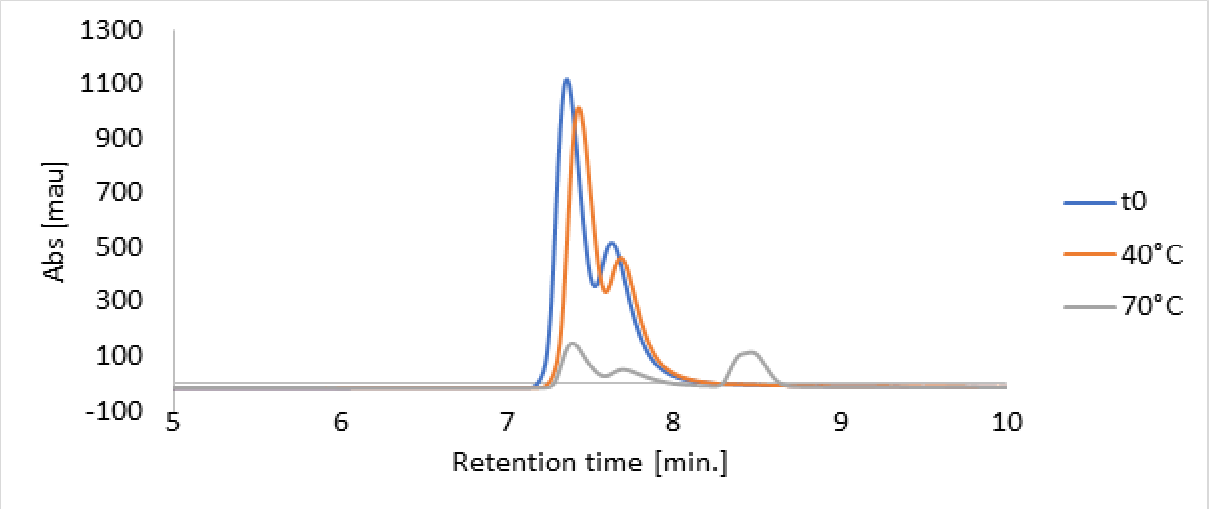

Figure S22. Thermal instability of TMT(AcOH)3 at pH 11

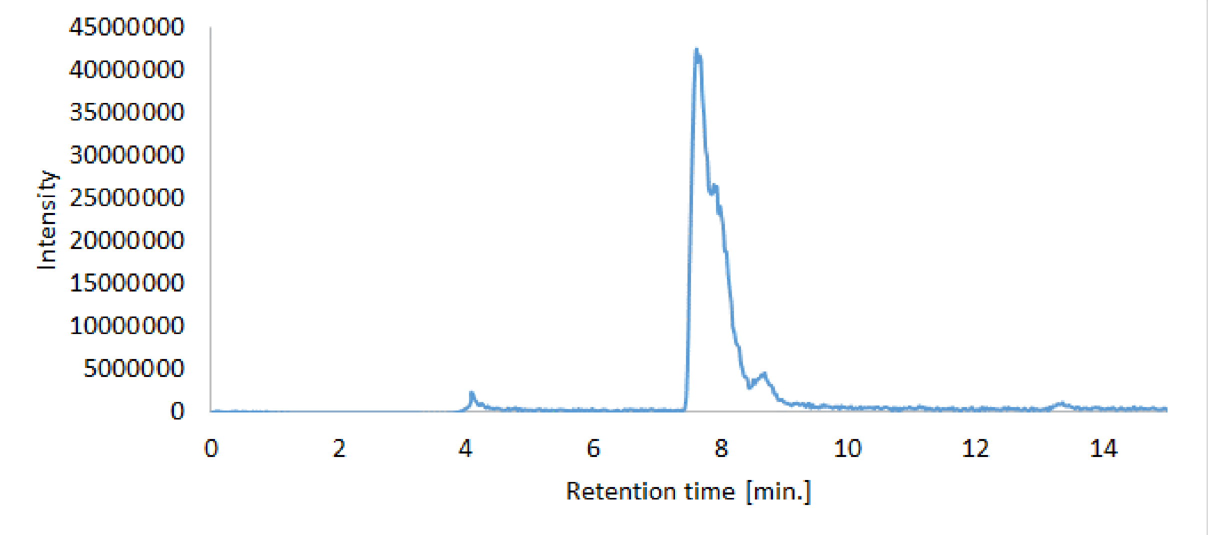

Figure S23. LC-MS for TMT(AcOH)$_3$ at pH 11 and 40°C (total ion chromatogram for m/z 200-500)

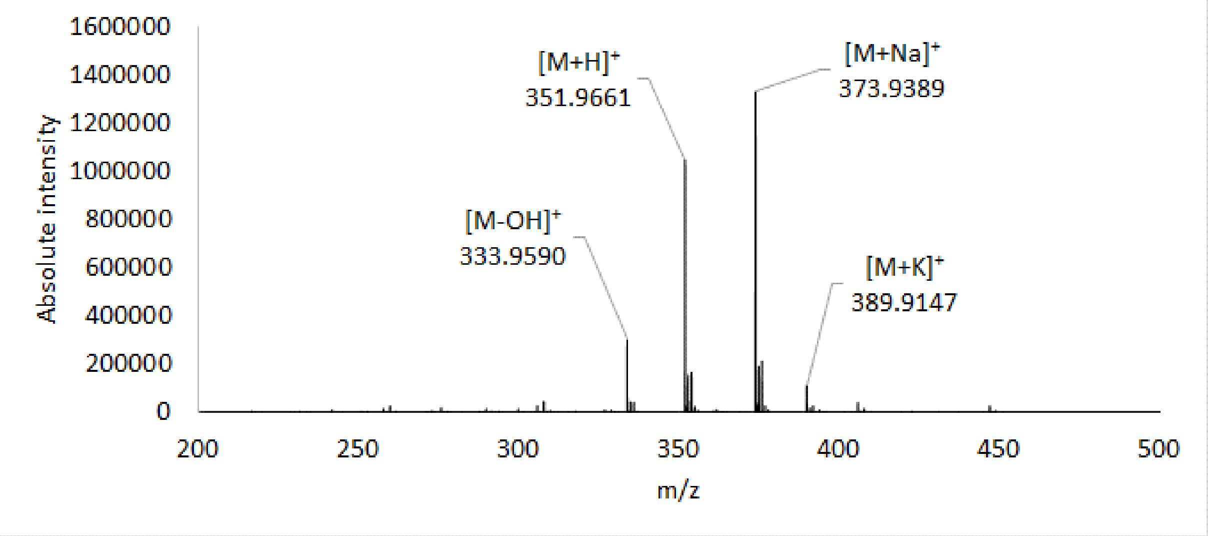

Figure S24. Averaged MS spectrum from 7 to 9 minutes derived from LC-MS of TMT(AcOH)$_3$ at pH 11 at 40°C. Absence of signals indicating degradation of TMT(AcOH)$_3$.

4. Transthioestrification and ligation reactions

4.1. Reactions with cysteamines

4.1.1. Ligation of cysteinylpeptides to [(4,6-disulfanyl-1,3,5-triazin-2-yl)sulfanyl]acetyl-βAla-Lys-OH

General procedure: The ligation was performed by mixing a Cys-peptide (about 2 mM) in 0.5 M TEAB buffer containing 20 mM TCEP and 1.5 equiv. of thiocyanuric acid ester. The reaction was carried at room temperature for 24 hours on a platform shaker. The progress of the reaction of cysteinyl-peptide was monitored by HPLC and offline MS characterization of the collected fractions. Samples for HPLC analysis were prepared by 10-fold dilution with water. Final solution was acidified with TFA to pH 2, and evaporated under nitrogen stream overnight. Products were purified using Varian ProStar HPLC system equipped with the TOSOH Bioscience TSKgel ODS 120T column (21.5 mm x 300 mm; 10µm), typically using the following solvent systems: 1%–70% B in A for 40 min, A 0.1% aqueous TFA, B 80% acetonitrile + 0.1% TFA, flow rate 7.0 ml/min, UV detection at 220 and 280 nm. Collected fractions were characterized by direct ESI-MS, and lyophilized. The purity of the products was confirmed by HPLC with UV detection at 220 nm.

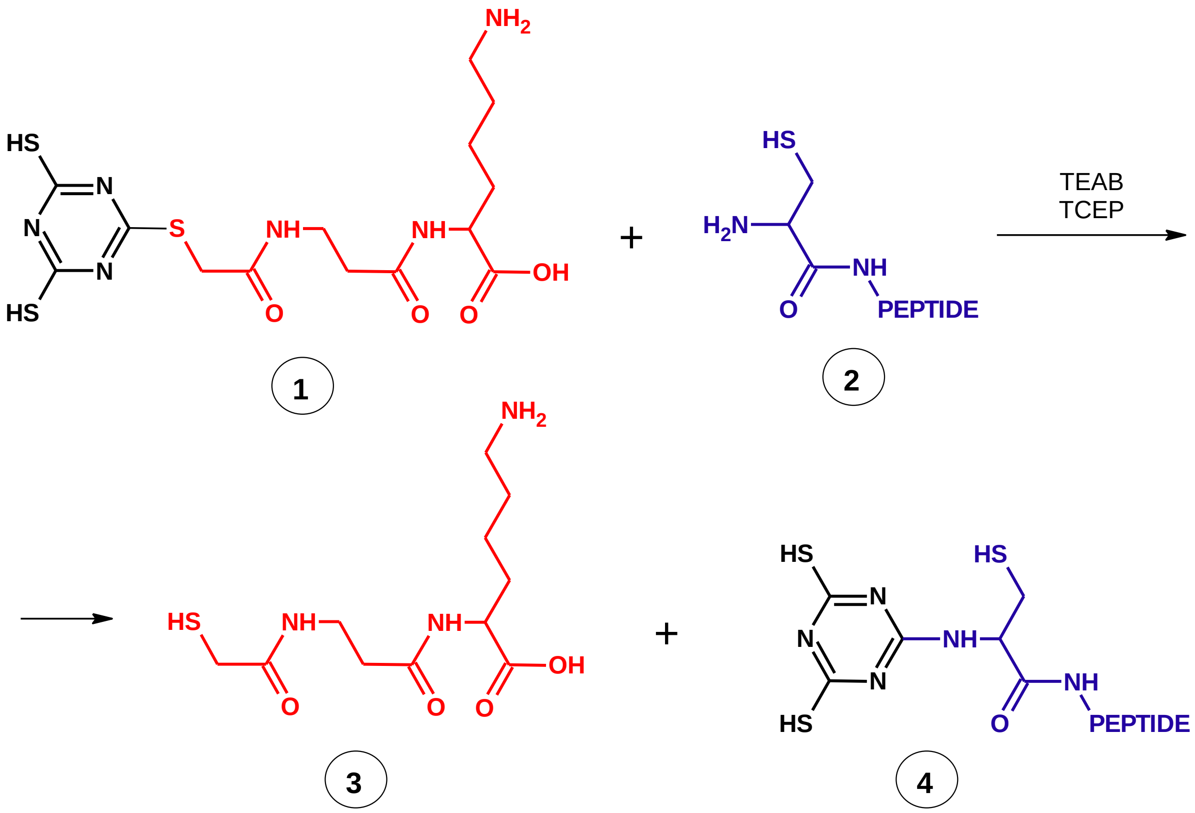

Scheme S1. Ligation of cysteinyl peptide to monoalkyl thiocyanurates represented by [(4,6-disulfanyl-1,3,5-triazin-2-yl)sulfanyl]acetyl-βAla-Lys-OH.

4.1.1.1. *Ligation of H-Cys-βAla-LEALK-NH₂*

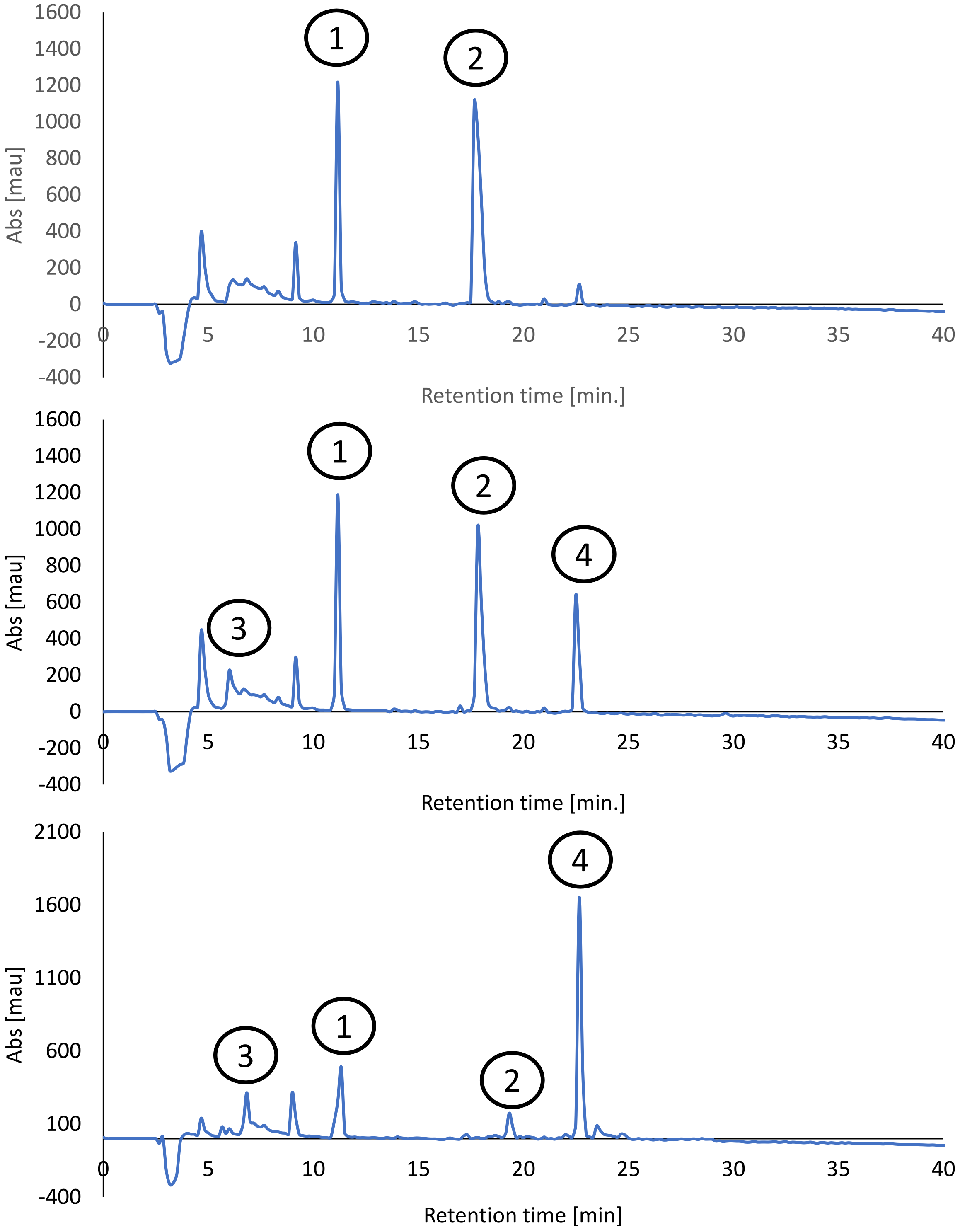

Figure S25. RP-HPLC analysis (column – Microsorb-MW 100-5 C-18, 250x4.6 mm; detection – UV, 220 nm; gradient – 0-80%B in 40 min; flowrate – 1 mL/min; eluent A – 0.1% TFA in water, eluent B – 0.1% TFA in 80% acetonitrile) of reaction mixture between H-Cys-βAla-LEALK-NH$_2$ and TMT-Ac-βAla-Lys-OH in the presence of 20 mM TCEP in 0.5M TEAB, from top to bottom: immediately after mixing reagents, after 1 hour of incubation, and after 24 hours of incubation at room temperature. Peaks signed as 1-4 correspond to appropriate compounds on the Scheme S1.

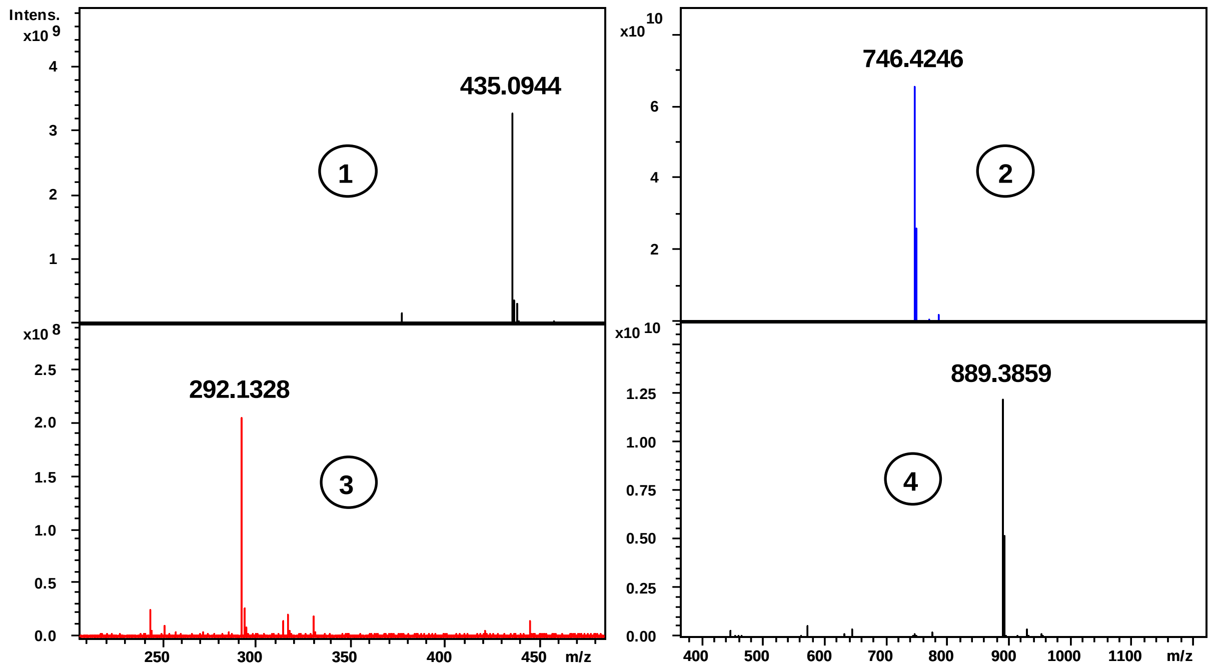

Figure S26. ESI-MS in positive ion mode, corresponding to peaks 1-4 from the Figure S25.

The product's characterization – DMT-Cys-βAla-LEALK-NH_2

HPLC: 22.5 min (sample preparation: 1 mg of the peptide dissolved in 1 mL of eluent A; column – Microsorb-MW 100-5 C-18, 250x4.6 mm; detection – UV, 220 nm; gradient – 0-80%B in 40 min; flowrate - 1 mL/min; eluent A – 0.1% TFA in water, eluent B – 0.1% TFA in 80% acetonitrile)

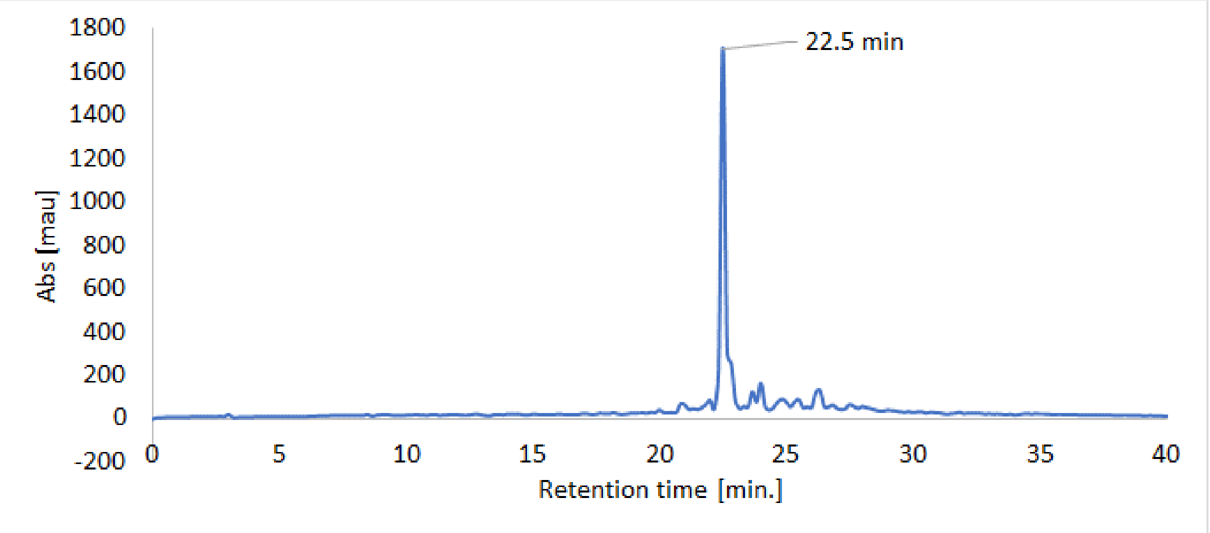

Figure S27. RP-HPLC chromatogram of purified DMT-Cys-βAla-LEALK-NH2

HRMS (ESI-MS) m/z: $[M+H]^+$ Calcd for $C_{35}H_{61}N_{12}O_9S_3$ 889.3841; found 889.3903

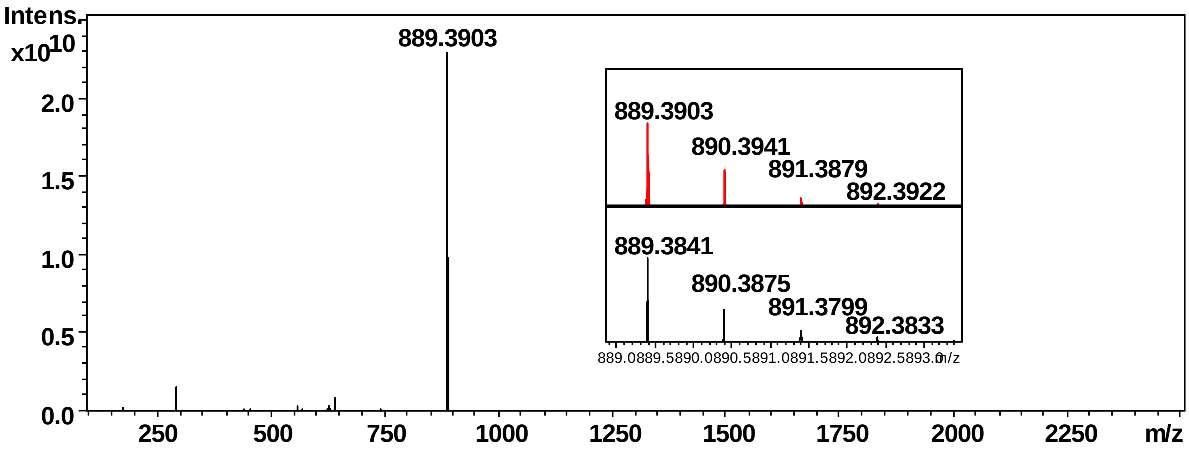

Figure S28. ESI-MS in positive ion mode of purified DMT-Cys-βAla-LEALK-NH$_2$ and comparison of measured (red) and simulated (black) isotopic pattern of $[M+H]^+$ ion.

ESI-MS(+)/MS, m/z: 771.4251 ($[M-C_2H_2N_2S_2+H]^+$, Calcd 771.4182), 754.3976 ($[[b_7-C_2H_2N_2S_2+H]^+$, Calcd 754.3922), 737.4371 ($[M-C_2H_2N_2S_2-H_2S+H]^+$, Calcd 737.4304), 728.4186 ($y_7-H_2S-H_2O$, Calcd 728.4124), 643.4190 (y6, Calcd 643,4137), 631.1830 (b_5, Calcd 631.1785), 626.3019 ($b_6-C_2H_2N_2S_2+H$, Calcd 626.2972), 560.1449 (b_4, Calcd 560.1414), 513.2161 ($b_5-C_2H_2N_2S_2+H$, Calcd 513.2131), 498.2955 ($b_5(y_6)$, Calcd 498.2922), 470.3001 ($a_5(y_6)$, Calcd 470.2973), 442.1782 (a5(y7)-H_2S-H_2O, Calcd 442.2119), 431.1013 (b_3, Calcd 431.0988), 385.2102 ($b_4(y_6)$, Calcd 385.2082)

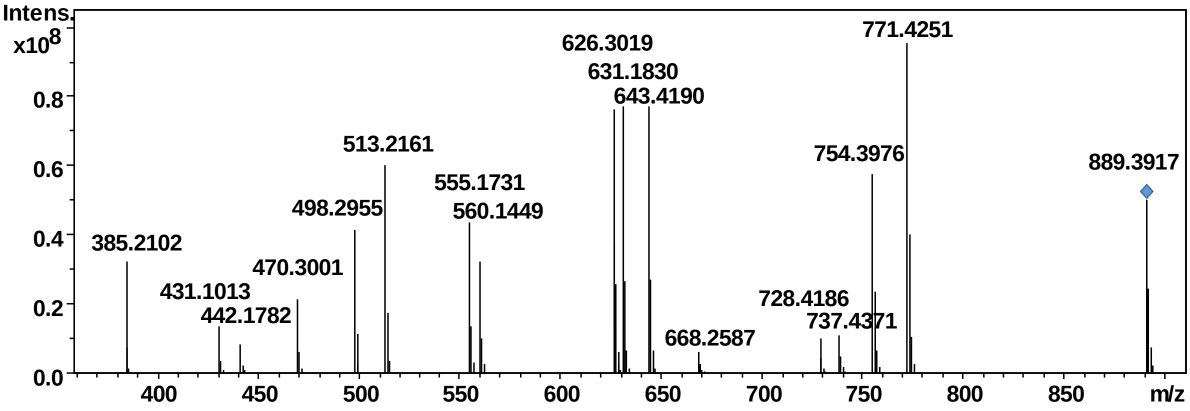

Figure S29. CID fragmentation MS^2 spectrum of DMT-Cys-βAla-LEALK-NH$_2$

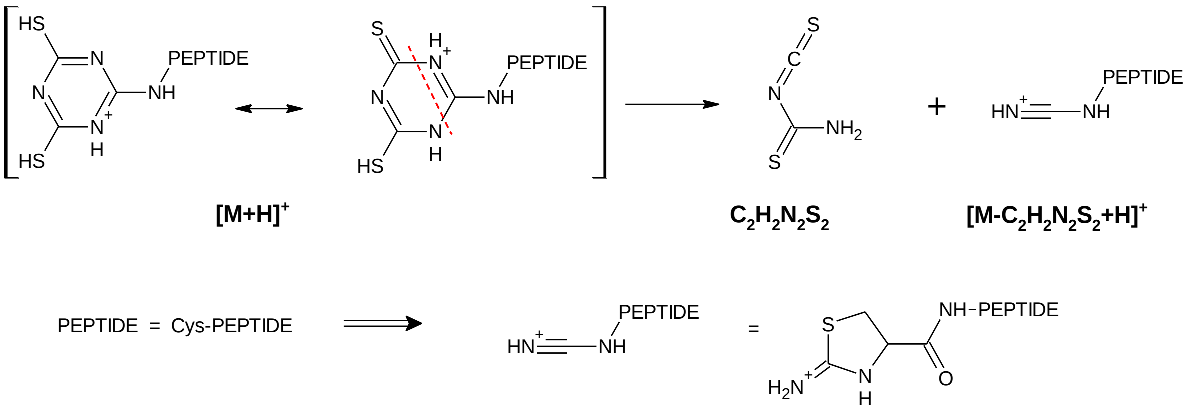

Figure S30. Fragmentation pathway involving disintegration of DMT moiety of DMT-Cys-peptides

4.1.1.2. Ligation of H-Cys-βAla-KALEKALKEALAKL-NH$_2$

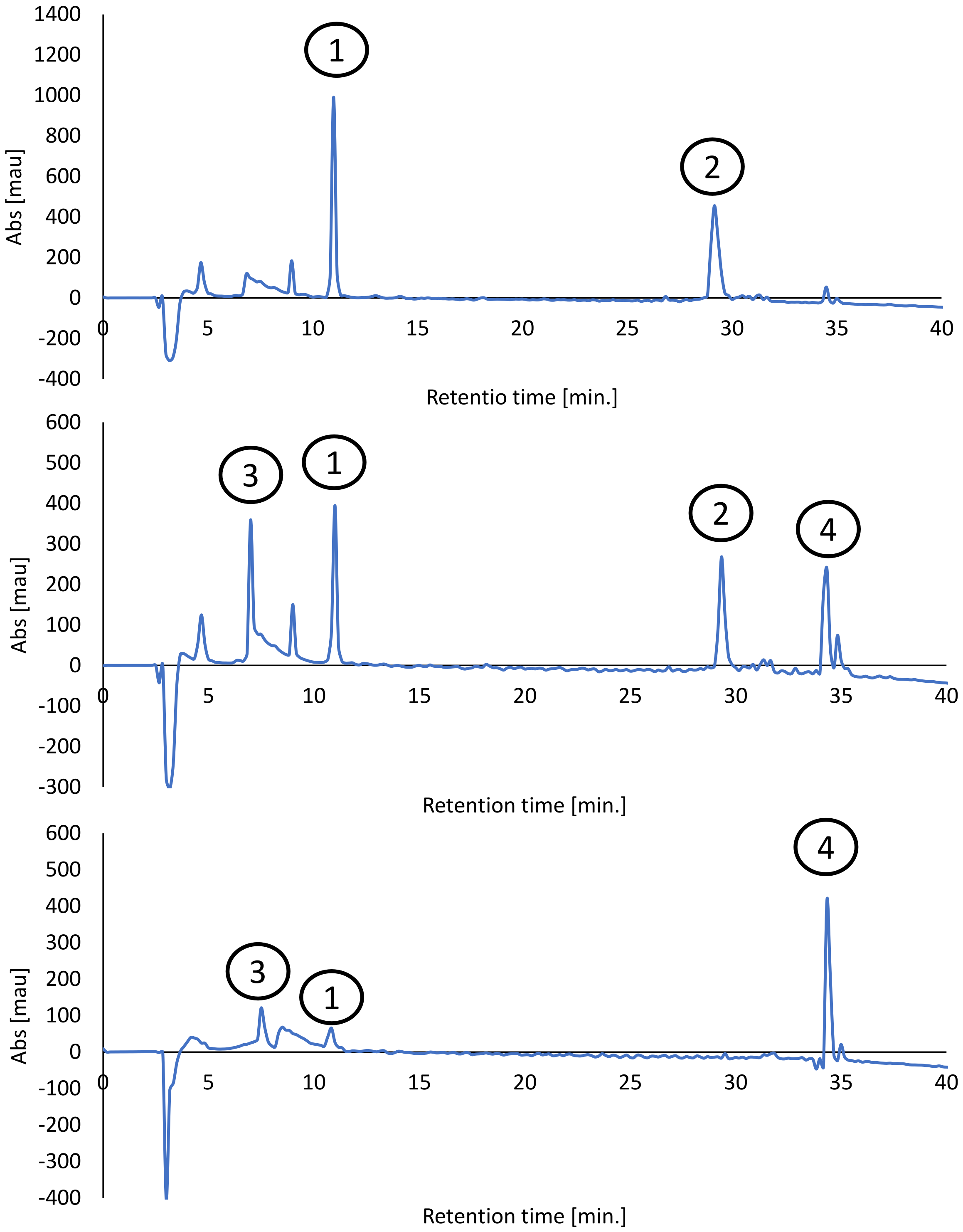

Figure S31.RP HPLC analysis (column – Microsorb-MW 100-5 C-18, 250x4.6 mm; detection – UV, 220 nm; gradient – 0 80%B in 40 min; flowrate – 1 mL/min; eluent A – 0.1% TFA in water, eluent B – 0.1% TFA in 80% acetonitrile) of reaction mixture between H-Cys-βAla-KALEKALKEALAKL-NH2 and TMT-Ac-βAla-Lys-OH in the presence of 20 mM TCEP in 0.5M TEAB, from top to bottom: immediately after mixing reagents, after 1 hour of incubation, and after 24 hours of incubation at room temperature. Peaks signed as 1-4 correspond to appropriate compounds on the Scheme S1.

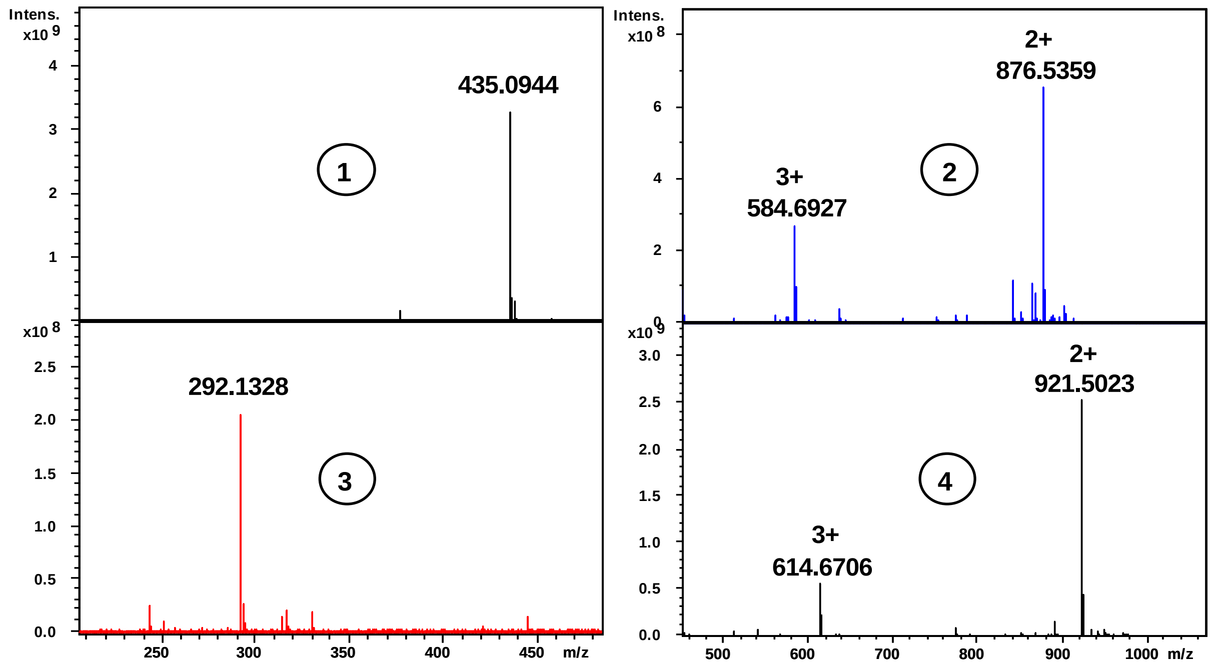

Figure S32.Figure S26. ESI-MS in positive ion mode, corresponding to peaks 1-4 from the Figure S31.

The product's characterization – DMT-Cys-βAla-KALEKALKEALAKL-NH_2

HPLC: 34.3 min (sample preparation: 1 mg of the peptide dissolved in 1 mL of eluent A; column – Microsorb-MW 100-5 C-18, 250x4.6 mm; detection – UV, 220 nm; gradient – 0-80%B in 40 min; flowrate – 1mL/min; eluent A – 0.1% TFA in water, eluent B – 0.1% TFA in 80% acetonitrile)

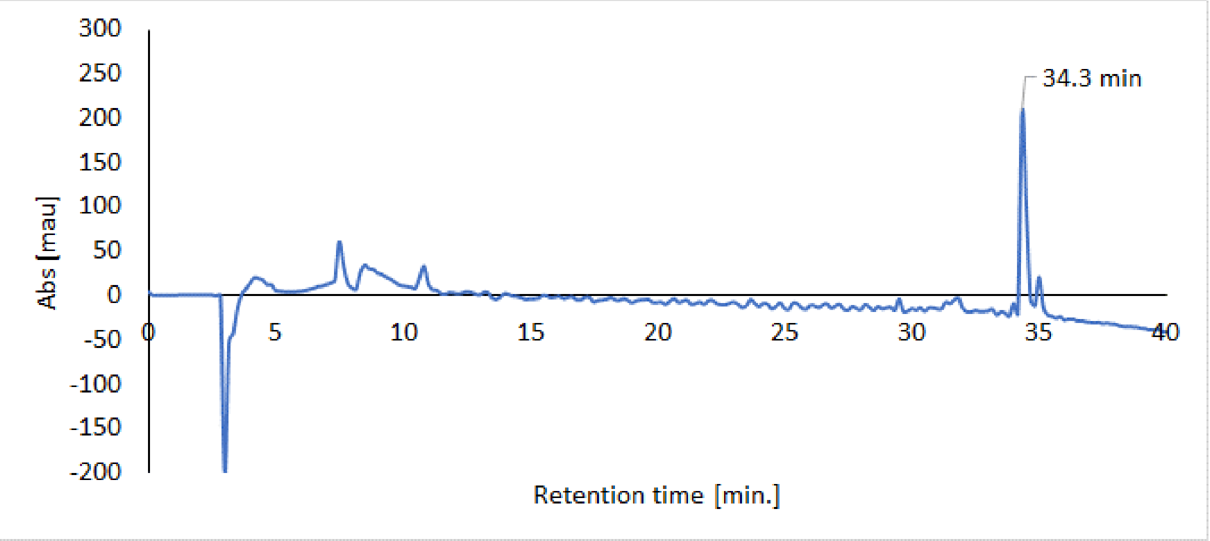

Figure S33. RP-HPLC chromatogram of purified DMT-Cys-βAla-KALEKALKEALAKL-NH_2

HRMS (ESI-MS) m/z: $[M+3H]^{3+}$ Calcd for $C_{79}H_{143}N_{24}O_{20}S_3$ 614.6685; Found 614.6706, $[M+2H]^{2+}$ Calcd for $C_{79}H_{142}N_{24}O_{20}S_3$; Found 921.5023

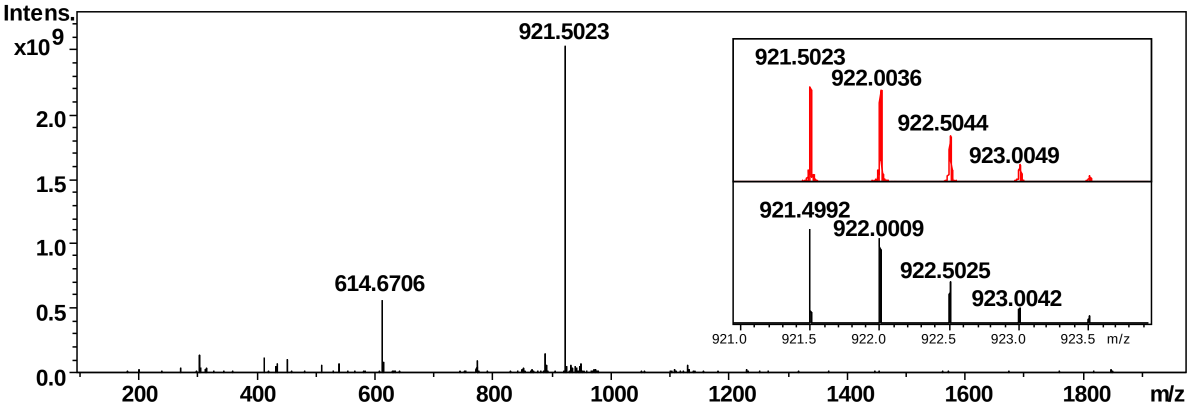

Figure S34.ESI-MS spectrum in positive ion mode of purified DMT-Cys-βAla-KALEKALKEALAKL-NH$_2$ and comparison of measured (red) and simulated (black) isotopic pattern of $[M+2H]^{2+}$ ion.

ESI-MS(+)/MS, m/z: 1465.8261 (b_{14}-$C_2H_2N_2S_2$, Calcd 1465.8195), 1394.7869 (b_{13}-$C_2H_2N_2S_2$, Calcd 1394.7824), 1379.8660 (z_{13}, Calcd 1379.8621), 1361.8578 (z_{13}-H_2O, Calcd 1361.8515), 1337.8202 ($b_{13}(y_{15})$, Calcd 1337,8151), 1308,8301 (z_{12}, Calcd 1308,8250), 1290,8213 (z_{12}-H_2O, Calcd 1290.8144), 1266.7812 ($b_{12}(y_{13})$, Calcd 1266.7780), 1195.7458 (z_{11}, Calcd 1195.7409), 1177.7343 (z_{11}-H_2O, Calcd 1177.7303), 1138.6871 ($b_{11}(y_{13})$, Calcd 1138.6830), 1067.6500 ($b_{10}(y_{12})$, Calcd 1067.6459), 996.6127 ($b_9(y_{12})$, Calcd 996.6088), 953.52797 (b_9, Calcd 953.5237), 938.6079 (z_9, Calcd 938.6033), 920.5952 (z_9-H_2O, Calcd 920.5928), 883.5281 ($b_8(y_{11})$, Calcd 883.5247), 867.5700 (z_8, Calcd 867.5662), 849.5589 (z_8-H_2O, Calcd 849.5557), 825.5226 ($b_8(y_9)$, Calcd 825.5193), 807.5128 ($b_8(y_9)$-H_2O, Calcd 807.5087), 769.4057 (b_7-$C_2H_2N_2S_2$, Calcd 769.4025), 754.4849 (z_7, Calcd 754.4822), 736.4743 (z_7-H_2O, Calcd 736.4716), 733.4161 ($a_8(y_{16})$-$3H_2O$, Calcd 733.4178), 697.4269 ($b_7(y_9)$, Calcd 697.4243), 641.3095 (b_6-$C_2H_2N_2S_2$, Calcd 641.3076), 626.3896 (z_6, Calcd 626.3872), 608.3788 (z_6-H_2O, Calcd 608.3766), 555.3519 ($b_5(y_8)$, Calcd 555.3501), 513.3048 ($b_5(y_6)$, Calcd 513.3031), 495.2943 ($b_5(y_6)$-H_2O, Calcd495.2926), 442.2672 ($b_4(y_7)$, Calcd442.2660), 399.1817 (b_4-$C_2H_2N_2S_2$, 399.1809), 381.1712 (b_4-H_2O-$C_2H_2N_2S_2$, Calcd 381.1703)

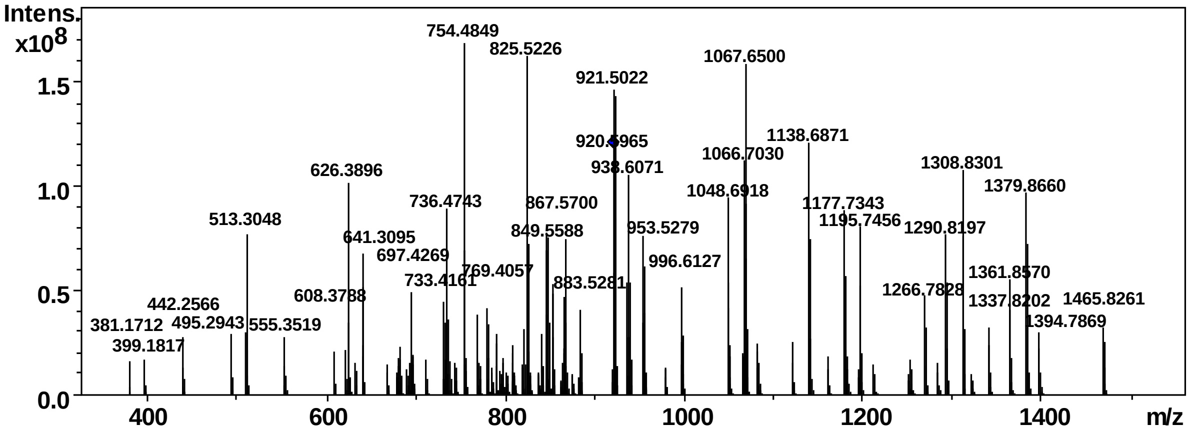

Figure S35. CID fragmentation MS^2 spectrum of DMT-Cys-βAla-KALEKALKEALAKL-NH$_2$, $[M+2H]^{2+}$ parent ion

4.1.2. Reaction of tris(carboxymethyl)thiocyanurate with the cysteamines (2-mercaptoethylamines)

Synthesis optimization: 350 mg of TMT(AcOH)$_3$ (1 mmol) and 390 mg of cysteamine (5 mmol) were dissolved in 10 mL of 0.5 M TEAB with 20 mM TCEP. The solution was divided into 1mL aliquots in glass vials. Obtained samples were freeze-dried and dissolved in 1mL of 0.5M TEAB immediately before an incubation. The reaction was carried out in a laboratory oven at 40-90°C for 24 hours, and in an ultrasonic bath (Bandelin RK 100H 80/160 W 35 kHz) for 2 hours. Samples for HPLC and LC-MS were prepared by 100-fold dilution with water. Nexera XR HPLC System withD detector coupled to IT-TOF mass spectrometer (Shimadzu) has been used to perform analyses. We analyzed HPLC chromatograms for 240 nm and extracted ion chromatograms of monosubstituted ($[M+H]^+$ m/z = 337), disubstituted ($[M+H]^+$ m/z = 322), and trisubstituted ($[M+H]^+$ m/z = 307) products.

HPLC: column – Aeris 3.6 μm PEPTIDE XB-C18 50 x 2.1 mm; detection -D, 240 nm; gradient – 1-70%B in 15 min; flowrate – 0.2 mL/min; eluent A – 0.1% formic acid in the water, eluent B – 0.1% formic acid in acetonitrile)

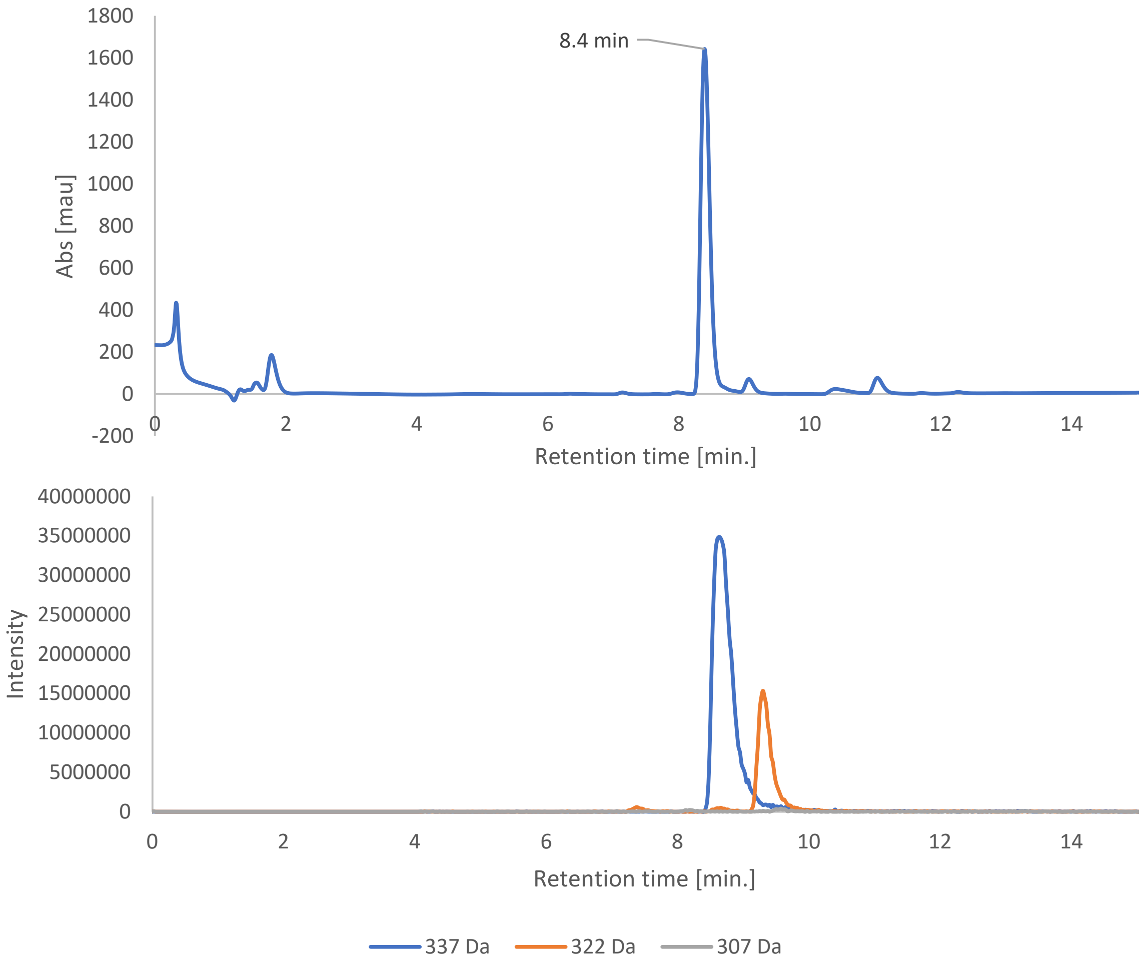

Figure S36. RP-HPLC profile (detection at 240 nm,D) of the reaction of cysteamine excess with TMT(AcOH)$_3$ at 40°C (top) and extracted ion chromatogram for hypothetical products (bottom): DMT(AcOH)$_2$(cysteamine) (blue), MMT(AcOH)(cysteamine)$_2$ (orange), and TAT(cysteamine)$_3$ (gray)

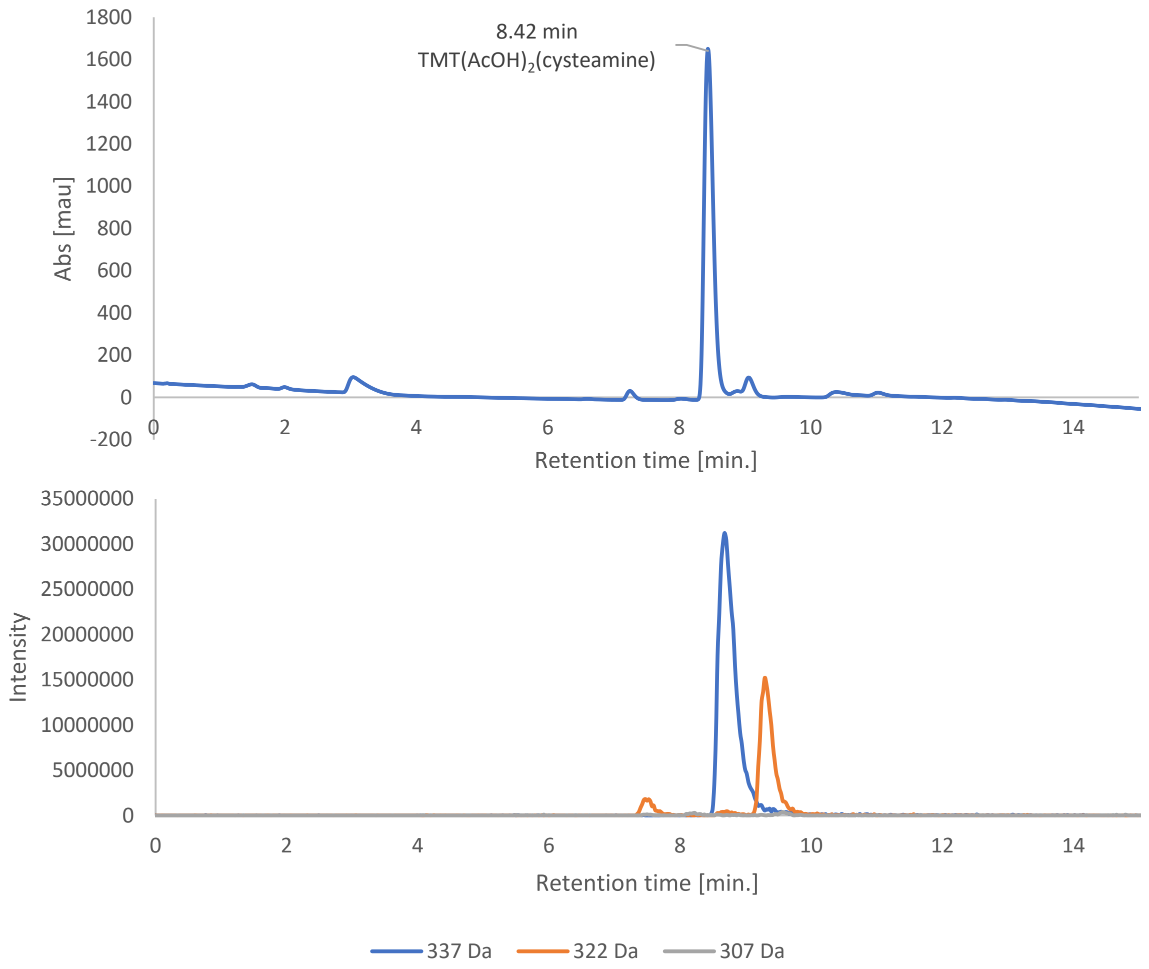

Figure S37. RP-HPLC profile (detection at 240 nm,D) of the reaction of cysteamine excess with $TMT(AcOH)_3$ at 50°C (top) and extracted ion chromatogram for hypothetical products (bottom): $DMT(AcOH)_2$(cysteamine) (blue), MMT(AcOH)(cysteamine)$_2$ (orange), and TAT(cysteamine)$_3$ (gray)

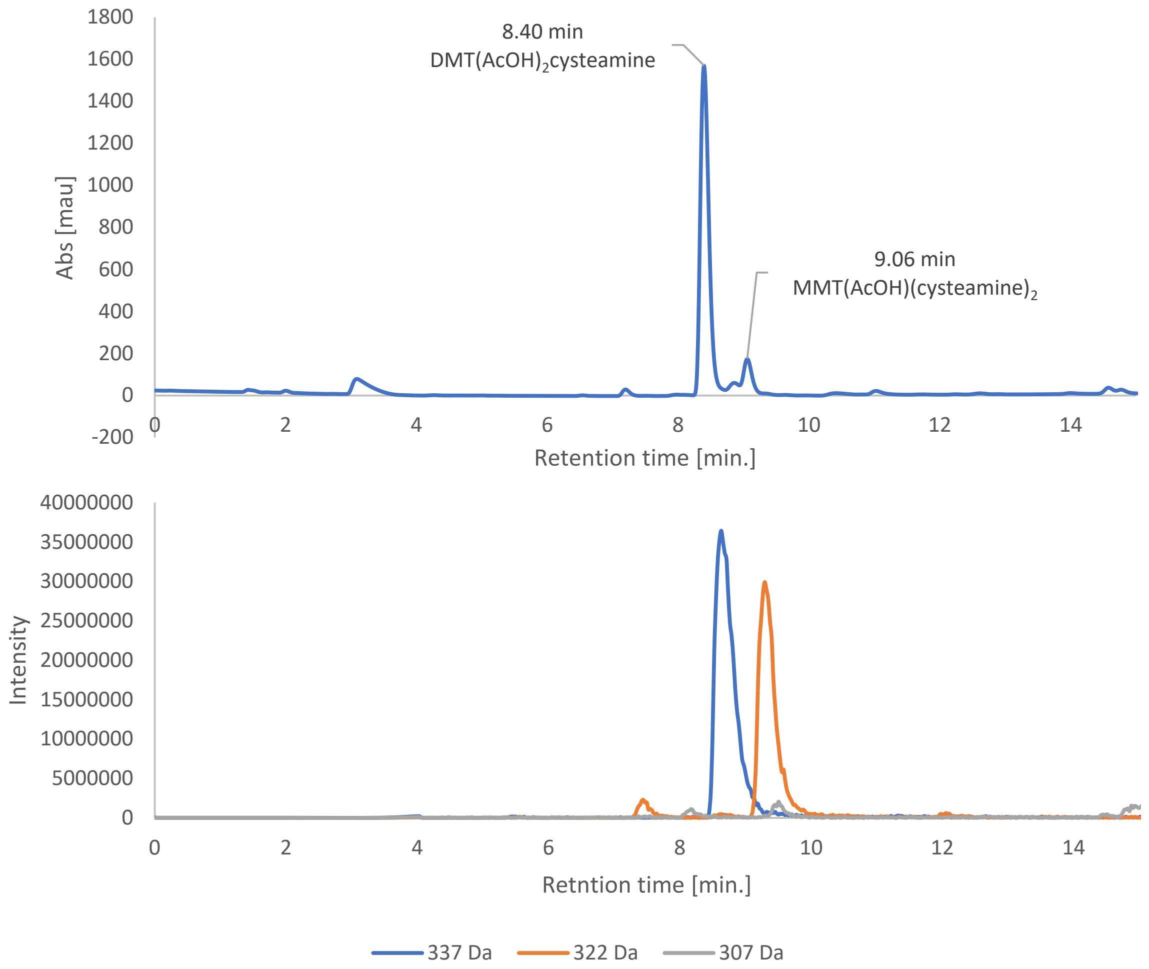

Figure S38.RP-HPLC profile (detection at 240 nm,D) of the reaction of cysteamine excess with TMT(AcOH)$_3$ at 60°C (top) and extracted ion chromatogram for hypothetical products (bottom): DMT(AcOH)$_2$(cysteamine) (blue), MMT(AcOH)(cysteamine)$_2$ (orange), and TAT(cysteamine)$_3$ (gray)

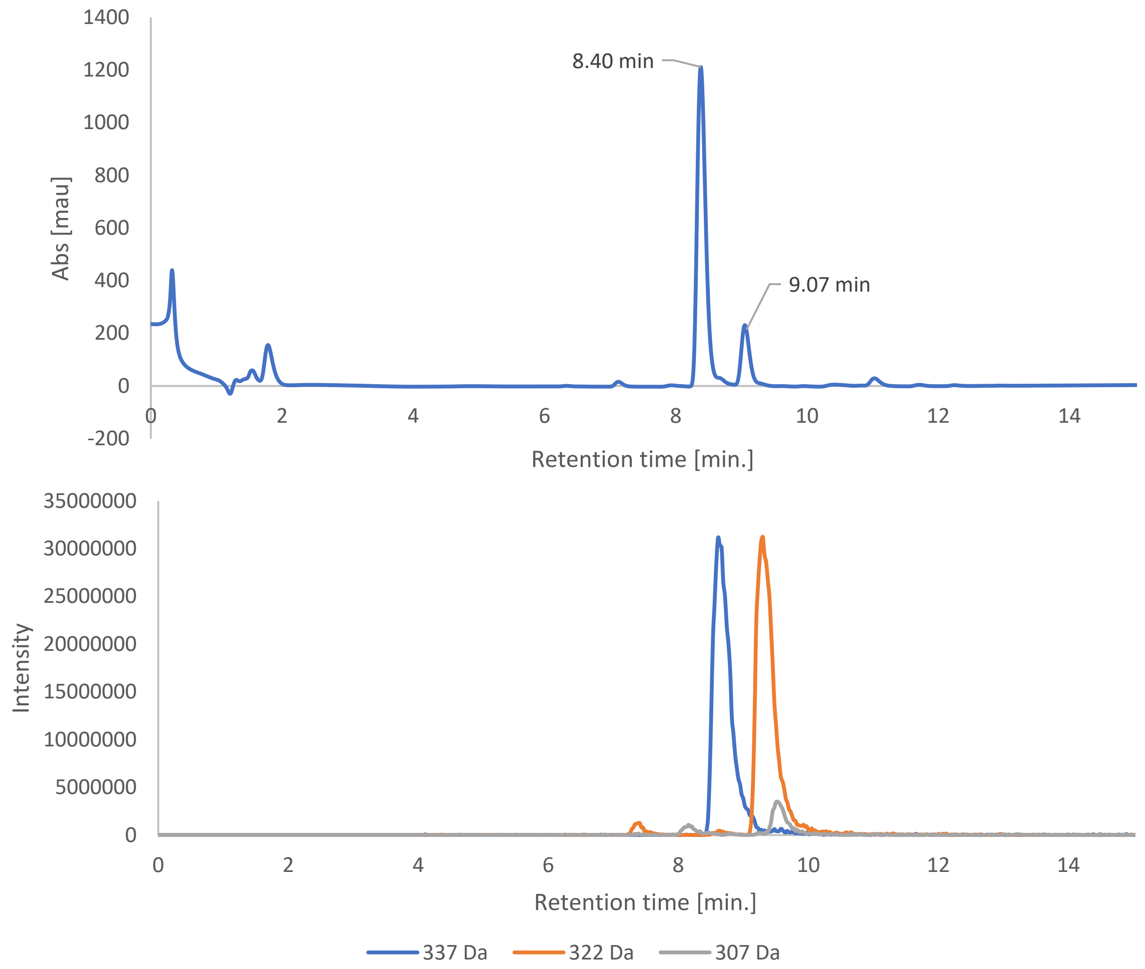

Figure S39. RP-HPLC profile (detection at 240 nm,D) of the reaction of cysteamine excess with TMT(AcOH)$_3$ at 70°C (top) and extracted ion chromatogram for hypothetical products (bottom): DMT(AcOH)$_2$(cysteamine) (blue), MMT(AcOH)(cysteamine)$_2$ (orange), and TAT(cysteamine)$_3$ (gray)

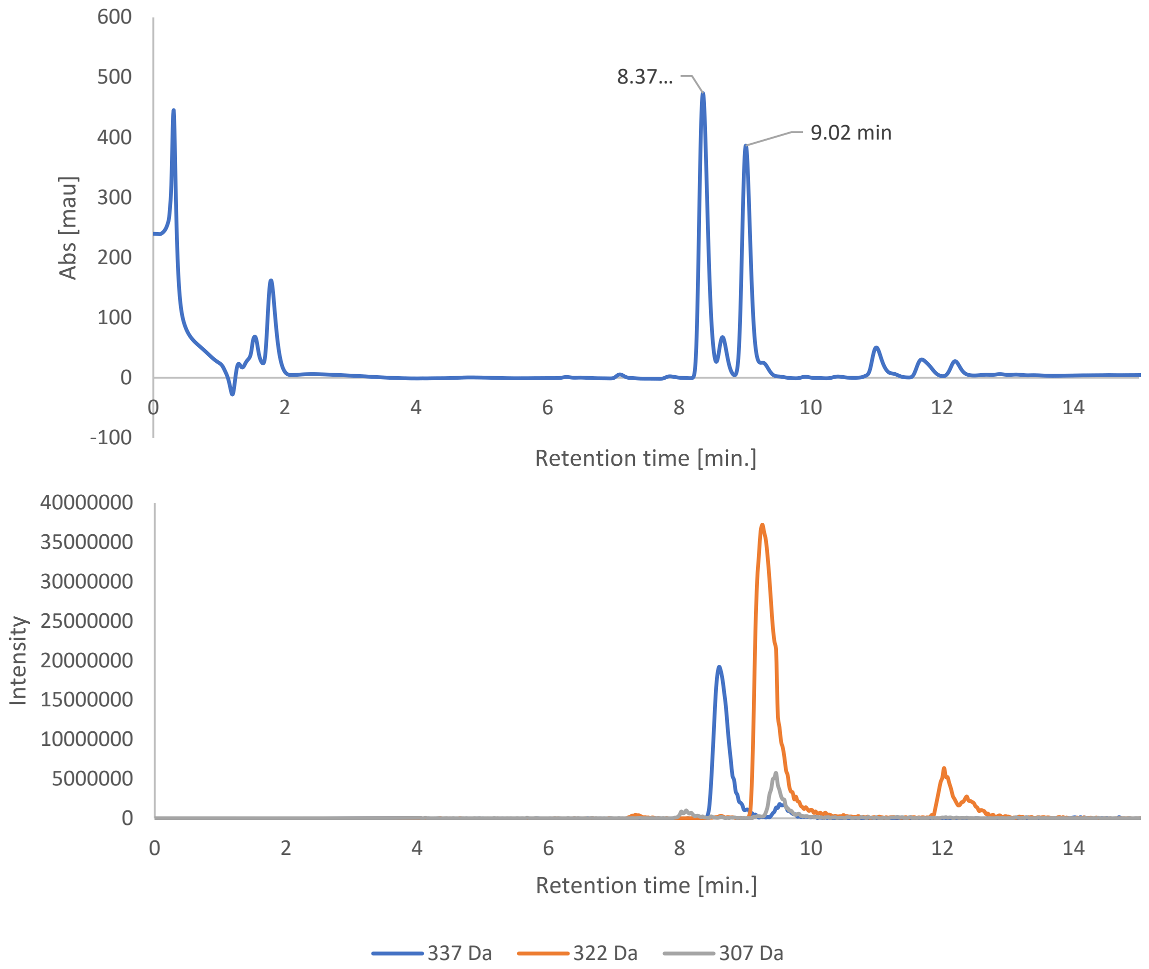

Figure S40. RP-HPLC profile (detection at 240 nm,D) of the reaction of cysteamine excess with TMT(AcOH)$_3$ at 80°C (top) and extracted ion chromatogram for hypothetical products (bottom): DMT(AcOH)$_2$(cysteamine) (blue), MMT(AcOH)(cysteamine)$_2$ (orange), and TAT(cysteamine)$_3$ (gray)

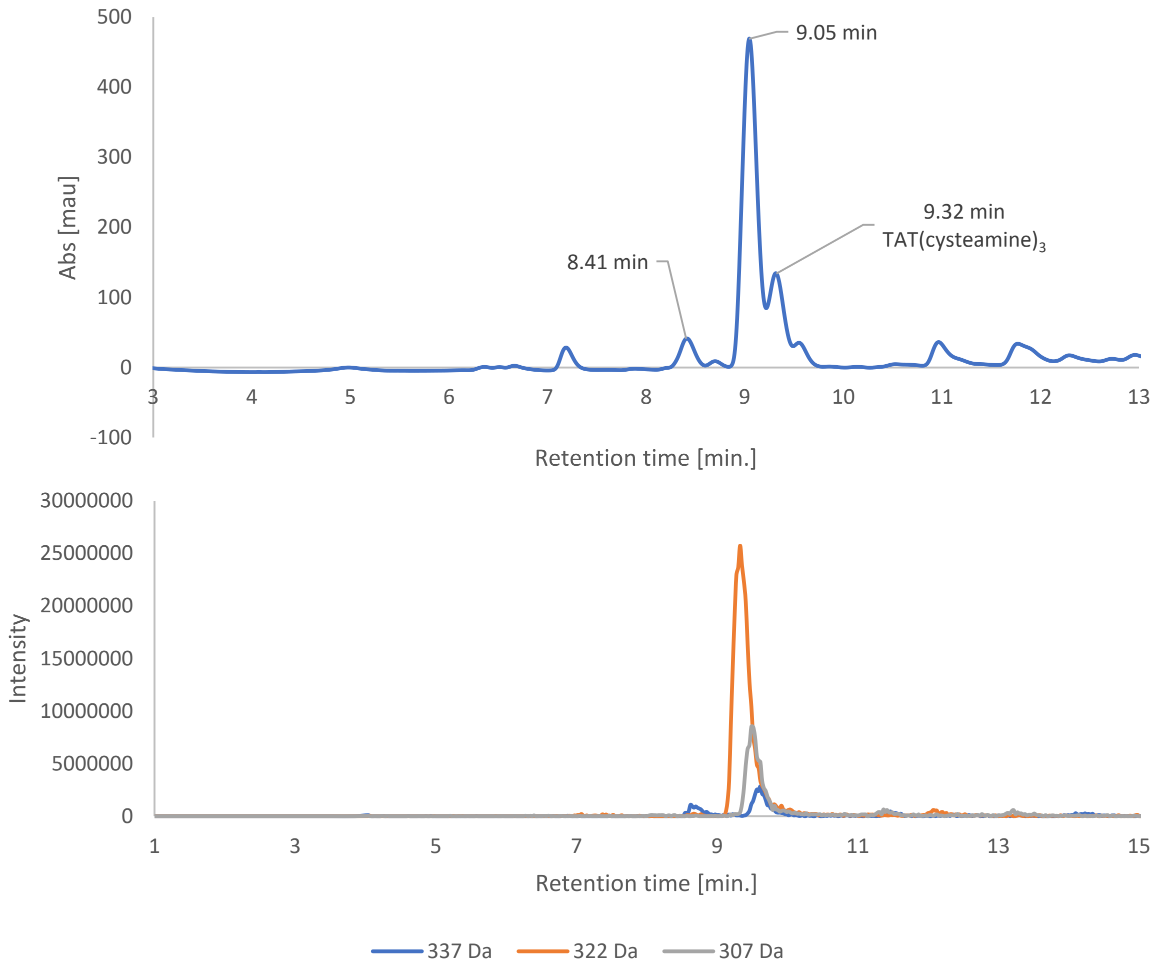

Figure S41. RP-HPLC profile (detection at 240 nm,D) of the reaction of cysteamine excess with TMT(AcOH)$_3$ at 90°C (top) and extracted ion chromatogram for hypothetical products (bottom): DMT(AcOH)$_2$(cysteamine) (blue), MMT(AcOH)(cysteamine)$_2$ (orange), and TAT(cysteamine)$_3$ (gray)

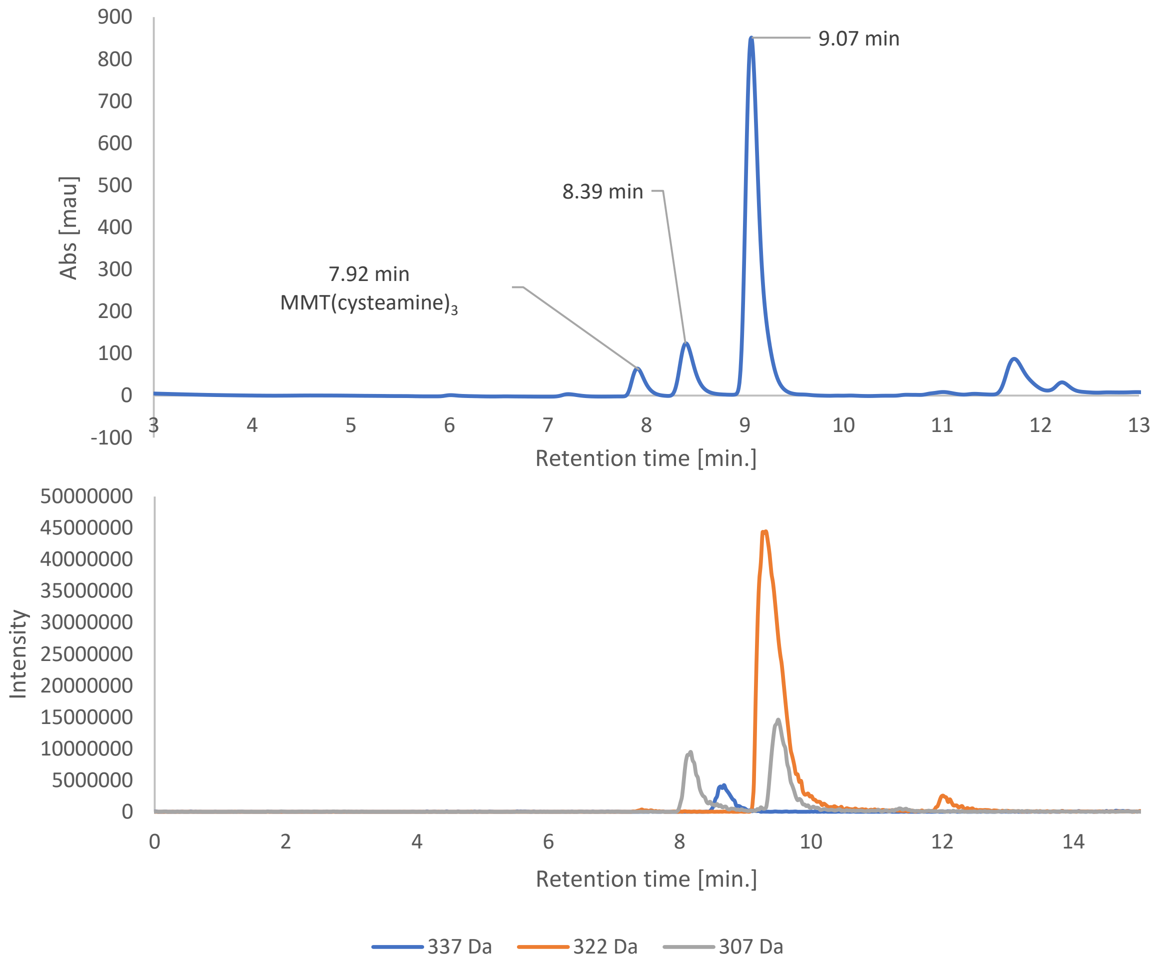

Figure S42. RP-HPLC profile (detection at 240 nm,D) of the reaction of cysteamine excess with TMT(AcOH)$_3$ by ultrasonication for 2 hours (top) and extracted ion chromatogram for hypothetical products (bottom): DMT(AcOH)$_2$(cysteamine) (blue), MMT(AcOH)(cysteamine)$_2$ (orange), and TAT(cysteamine)$_3$ (gray)

DMT(AcOH)$_2$(cysteamine) – *N*-[4,6-bis(carboxymethylsulfanyl)-1,3,5-triazin-2-yl]cysteamine

Synthesis: 100 mg TMT(AcOH)$_3$ (0.3 mmol) and 78 mg cysteamine (1 mmol) were dissolved in 5 mL of 0.5 M TEAB containing 20 mM TCEP. The reaction mixture was stirred on a rotary shaker for 24 hours at room temperature. Progress of the reaction was checked by RP-HPLC analysis with detection at 240 nm. Samples for the analysis were prepared by 100-fold dilution of the reaction mixture with water. Final mixture was acidified to pH 2 by TFA, and lyophilized. Crude product was purified using Varian ProStar HPLC system equipped with the TOSOH Bioscience TSKgel ODS 120T column (21.5 mm x 300 mm; 10µm), typically using the following solvent systems: 1% for 5 min, 1–40% B in A for 40 min, A 0.1% aqueous TFA, B 80% acetonitrile + 0.1% TFA, flow rate 7.0 ml/min. Collected fractions were characterized by direct ESI-MS, and lyophilized. Purity of the product was confirmed by analytical RP-HPLC.

HPLC: 8.3 min (column – Aeris 3.6 µm PEPTIDE XB-C18 50 x 2.1 mm; detection -D, 240 nm; gradient – 1-70%B in 15 min; flowrate 0.2 mL/min; eluent A – 0.1% formic acid in water, eluent B – 0.1% formic acid in acetonitrile)

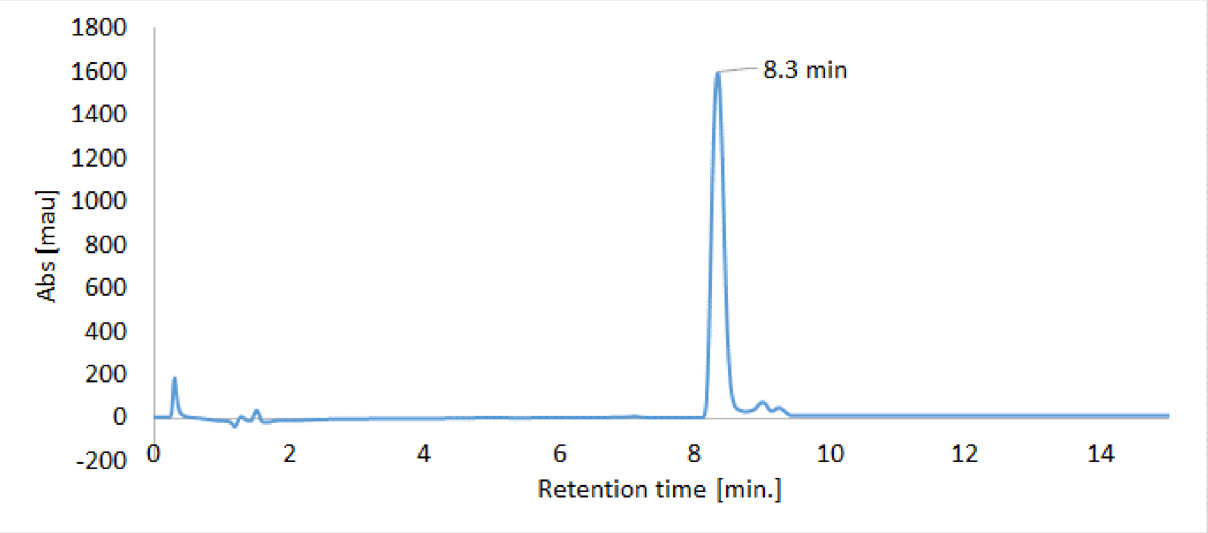

Figure S43. RP-HPLC chromatogram of purified DMT(AcOH)$_2$(cysteamine)

HRMS (ESI-MS) m/z: [M-H$_2$O+H]$^+$ Calcd for $C_9H_{11}N_4O_3S_3$ 318.9993; Found 318.9946, [M+H]$^+$ Calcd for $C_9H_{13}N_4O_4S_3$ 337.0093; Found 337.0101, [M+Na]$^+$ Calcd for $C_9H_{12}N_4O_4S_3Na$ 358.9913; Found 358.9953.

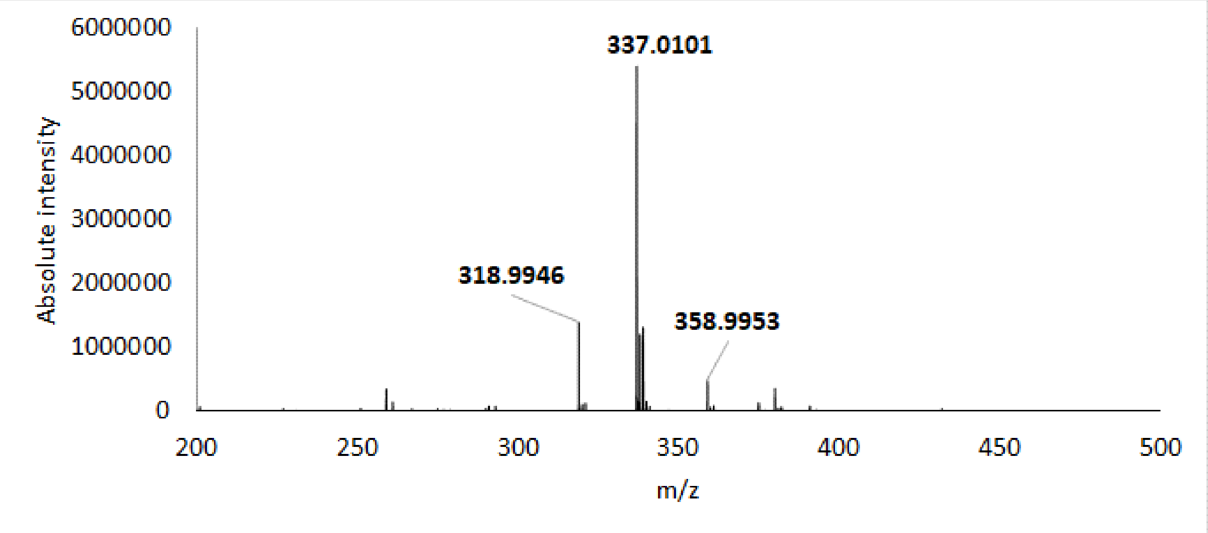

Figure S44. ESI-MS spectrum in positive ion mode of DMT(AcOH)$_2$(cysteamine)

1H NMR (DMSO-d6, 500 MHz, 25°C) δ (ppm) = 8.21 (t, 1H, NH, cysteamine), 3.87 (s, 2H, CH_2, AcOH), 3.85 (s, 2H, CH_2, AcOH), 3.40 (q, 2H, CH_2, cysteamine), 2.60 (q, 2H, CH_2, cysteamine), 2.31 (t, 1H, SH, cysteamine)

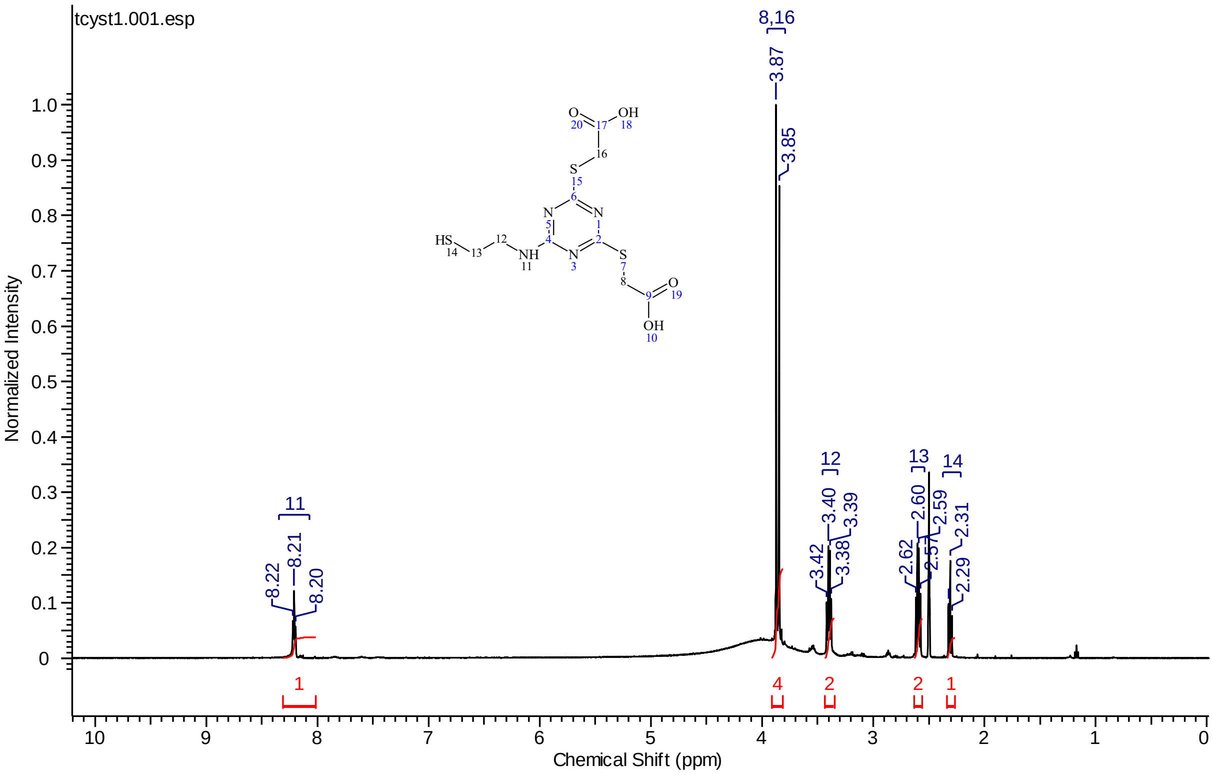

Figure S45. 1H-NMR of DMT(AcOH)$_2$(cysteamine) in DMSO-d$_6$

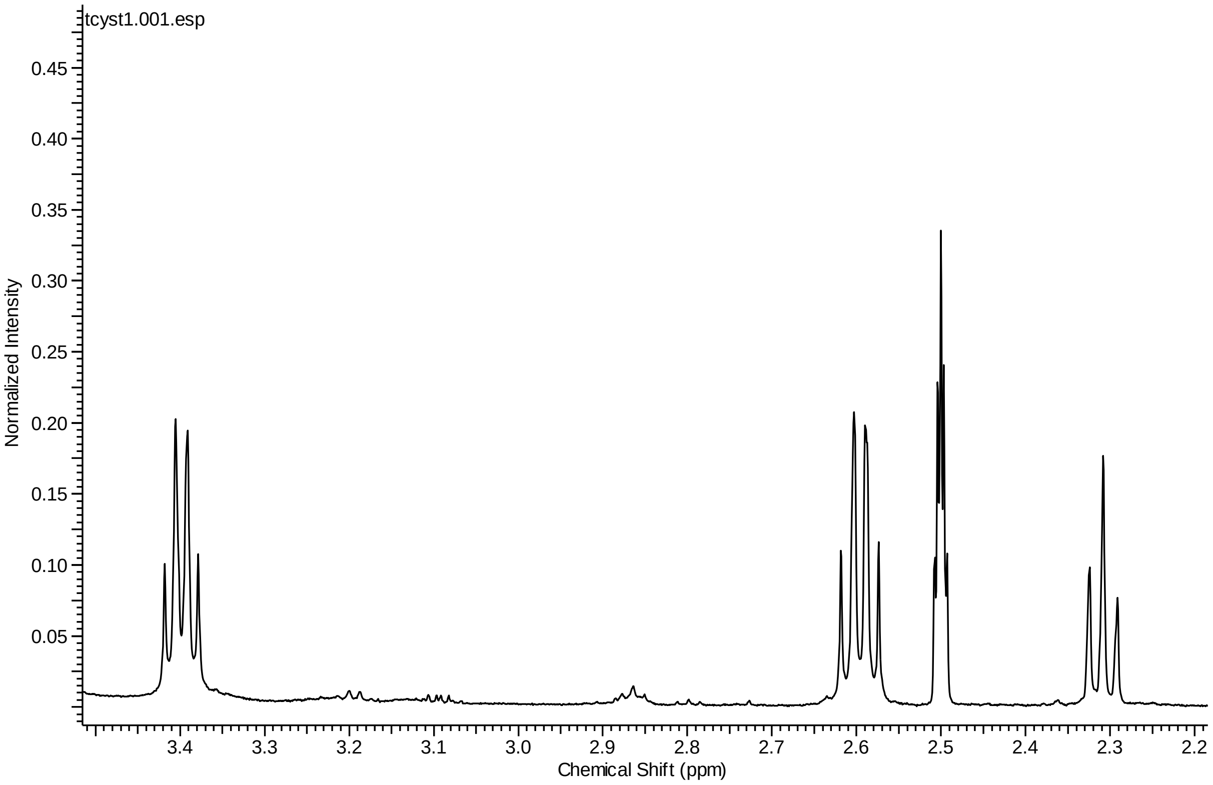

Figure S46. The region of alkyl 1H NMR shifts corresponding to cysteamine and thioglycolic acid residues in DMT(AcOH)$_2$(cysteamine). Broadening of the analyte signals relative to the residual solvent signal.

13C{H} NMR (DMSO-d6, 125 MHz, 25°C) δ (ppm) = 178.3 & 177.4 (triazine, C-S), 170.0 & 169.8 (AcOH, COOH), 162.1 (triazine, C-NH), 43.5 (cysteamine, C-NH), 32.4 & 32.0 (AcOH, CH_2) 23.1 (cysteamine, C-S)

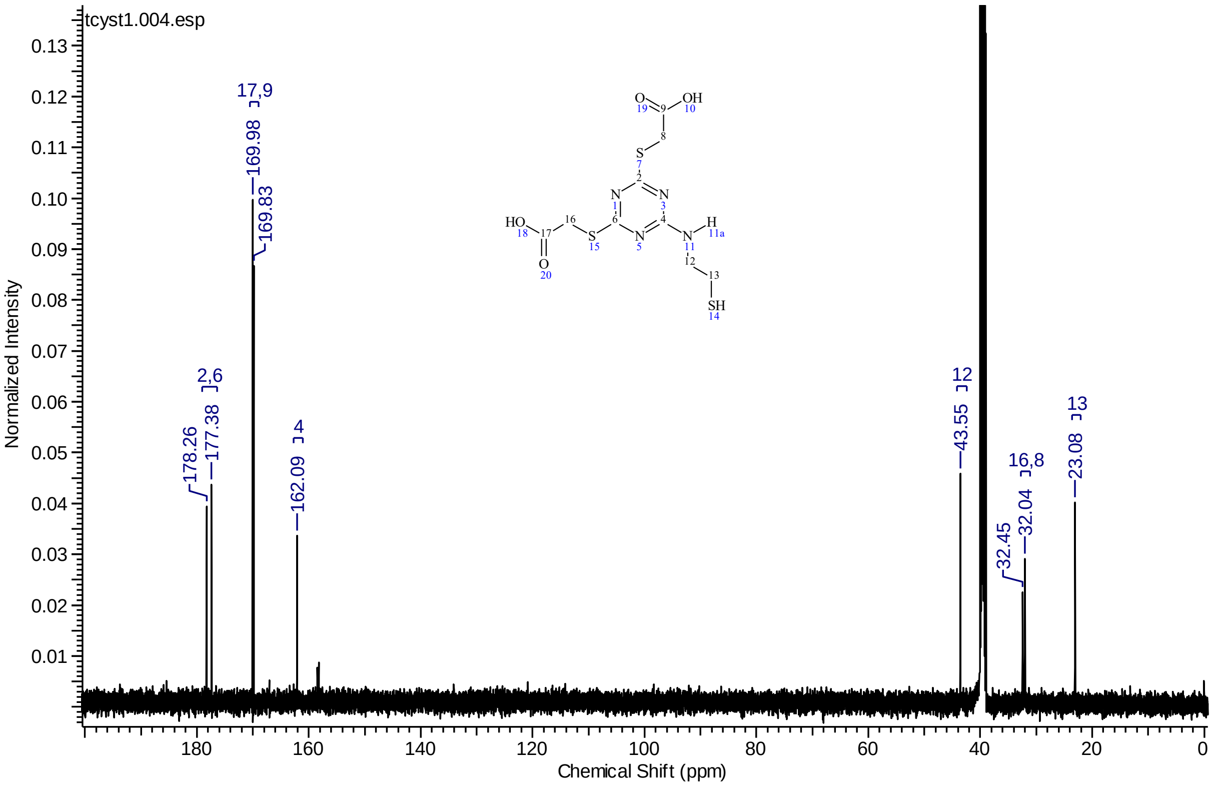

Figure S47. 13C{H} NMR spectrum of DMT(AcOH)2(cysteamine) in DMSO d6. Duplication of signals corresponding to thioglycolic acid residues proves the coplanarity of the 1,3,5-triazin-2-yl cysteamine bond differentiating other substituents.

MMT(AcOH)(cysteamine)$_2$ – *N*-[4-(carboxymethylsulfanyl)-6-(sulfanylethylamino)]cysteamine

Synthesis: 100 mg TMT(AcOH)$_3$ (0.3 mmol) was dissolve in a solution of 117 mg cysteamine (1.5 mmol) in 5 mL of 0.5 M TEAB containing 20 mM TCEP, followed by immediate sonication in a glass vial equipped with septum, and immersed in an ultrasonic bath. The reaction mixture was sonicated 4 times 30 minutes. Progress of the reaction was checked by RP-HPLC analysis with detection at 240 nm. Samples for the analysis were prepared by 100-fold dilution of the reaction mixture with water. Final mixture was acidified to pH 2 by TFA, and lyophilized. Crude product was purified using Varian ProStar HPLC system equipped with the TOSOH Bioscience TSKgel ODS 120T column (21.5 mm x 300 mm; 10µm), typically using the following solvent systems: 1% for 5 min, 1–40% B in A for 40 min, A 0.1% aqueous TFA, B 80% acetonitrile + 0.1% TFA, flow rate 7.0 ml/min. Collected fractions were characterized by direct ESI-MS, and lyophilized. Purity of the product was confirmed by analytical RP-HPLC.

HPLC: 9.0 min (column – Aeris 3.6 µm PEPTIDE XB-C18 50 x 2.1 mm; detection -D, 240 nm; gradient – 1-70%B in 15 min; flowrate 0.2 mL/min; eluent A – 0.1% formic acid in water, eluent B – 0.1% formic acid in acetonitrile)

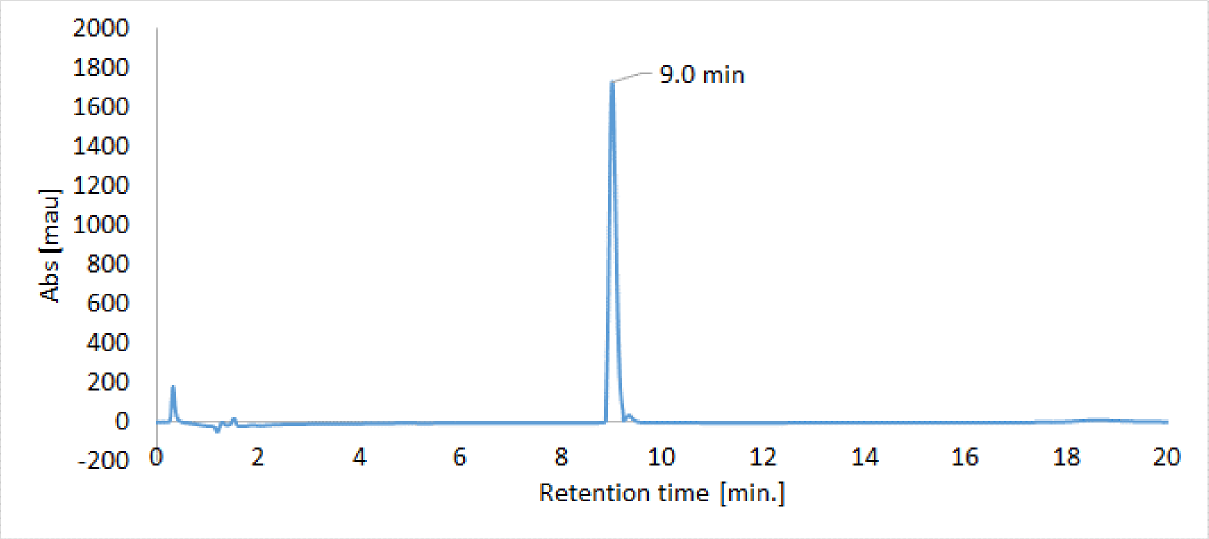

Figure S48. RP-HPLC chromatogram of purified DMT(AcOH)(cysteamine)$_2$

HRMS (ESI-MS) m/z: $[M+H]^+$ Calcd for $C_9H_{16}N_5O_2S_3$ 322.0461; Found 322.0521.

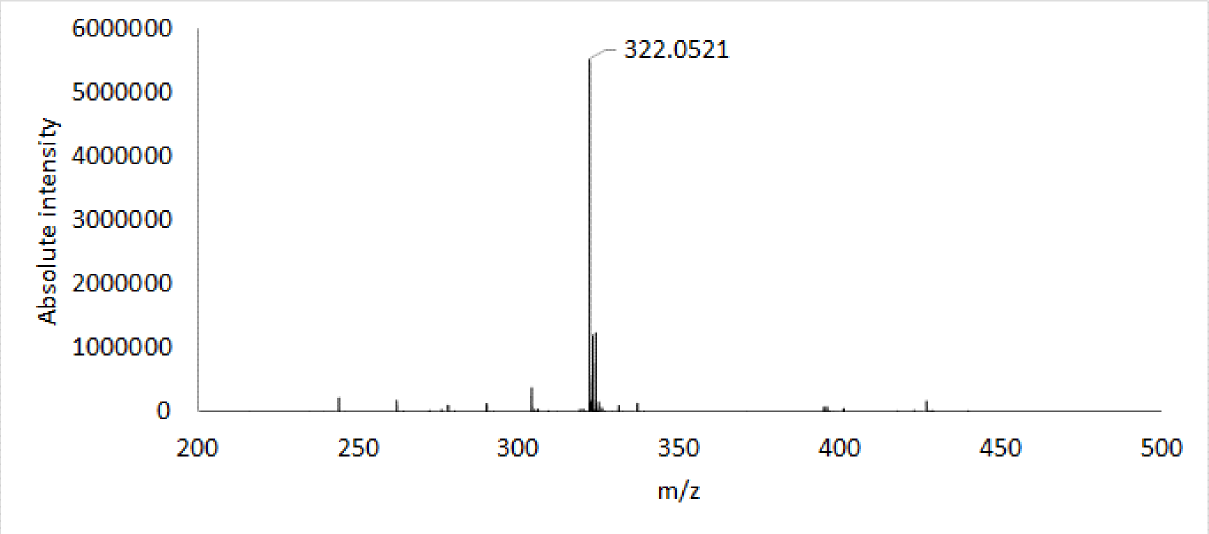

Figure S49. ESI-MS spectrum in positive ion mode of MMT(AcOH)(cysteamine)$_2$

1H NMR (DMSO-d6, 500 MHz, 25°C) δ (ppm) = 7.51 – 7.64 (2H, NH, cysteamine); 3.81-3.87 (2H, CH_2, AcOH); 3.32-3.41 (4H, $CH_2^{12,16}$, cysteamine); 2.56-2.65 (4H, $CH_2^{13,17}$, cysteamine); 2.26-2.37 (2H, SH,cysteamine)

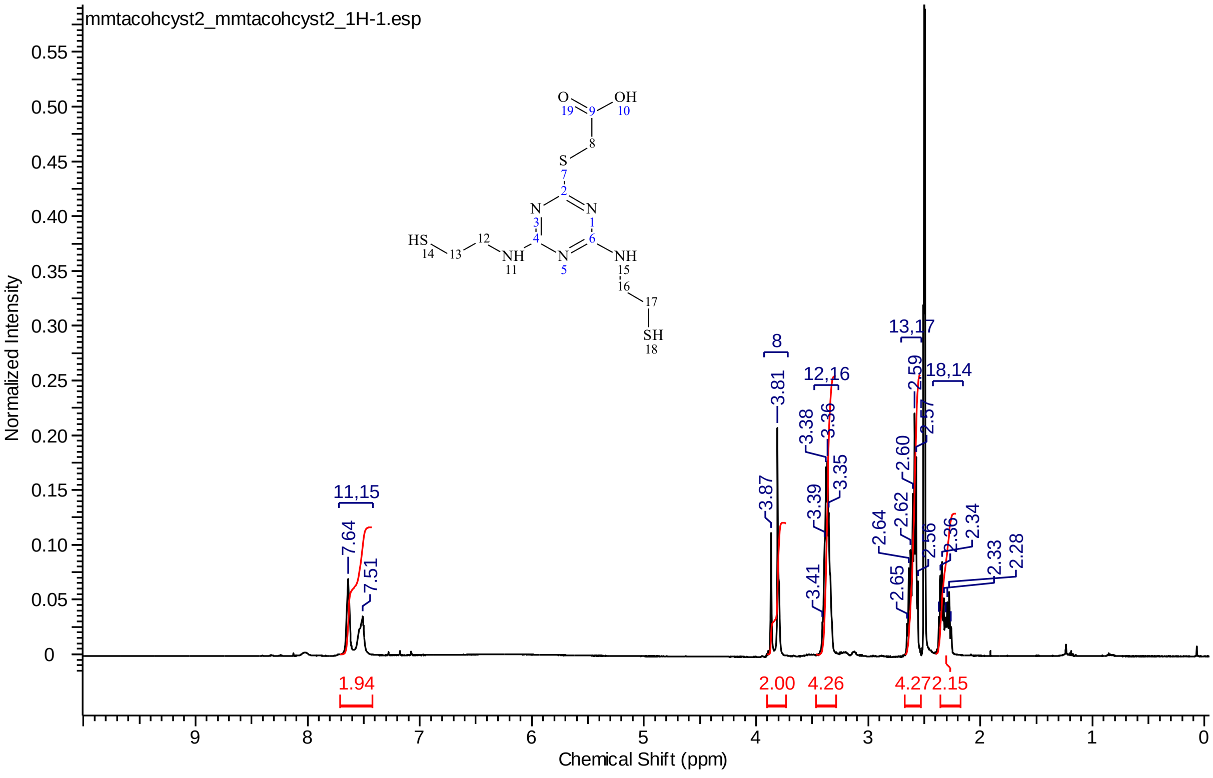

Figure S50. 1H NMR spectrum of MMT(AcOH)(cysteamine)₂ in DMSO-d₆

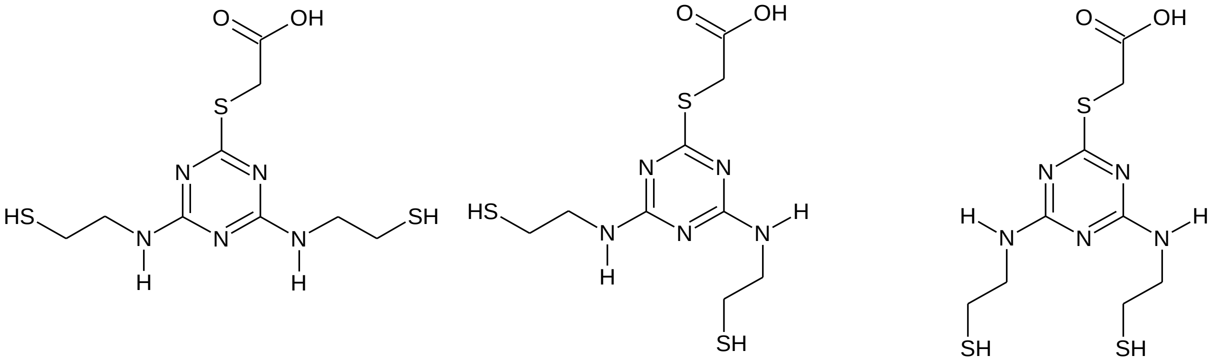

Figure S51. Possible isomers of MMT(AcOH)(cysteamine)₂

13C{H} NMR (DMSO-d6, 125 MHz, 25°C) δ (ppm) = 178.9 & 177.4 & 176.2 (triazine, C-S); 170.7 & 170.6 (AcOH, COOH); 163.8 & 163.6 & 163.5 & 162.8 (triazine, C-NH); 44.1 & 43.9 (cysteamine, C-NH); 32.9 & 32.5 & 32.0 (AcOH, CH_2); 23.9 & 23.6 & 23.5 (cysteamine, C-S)

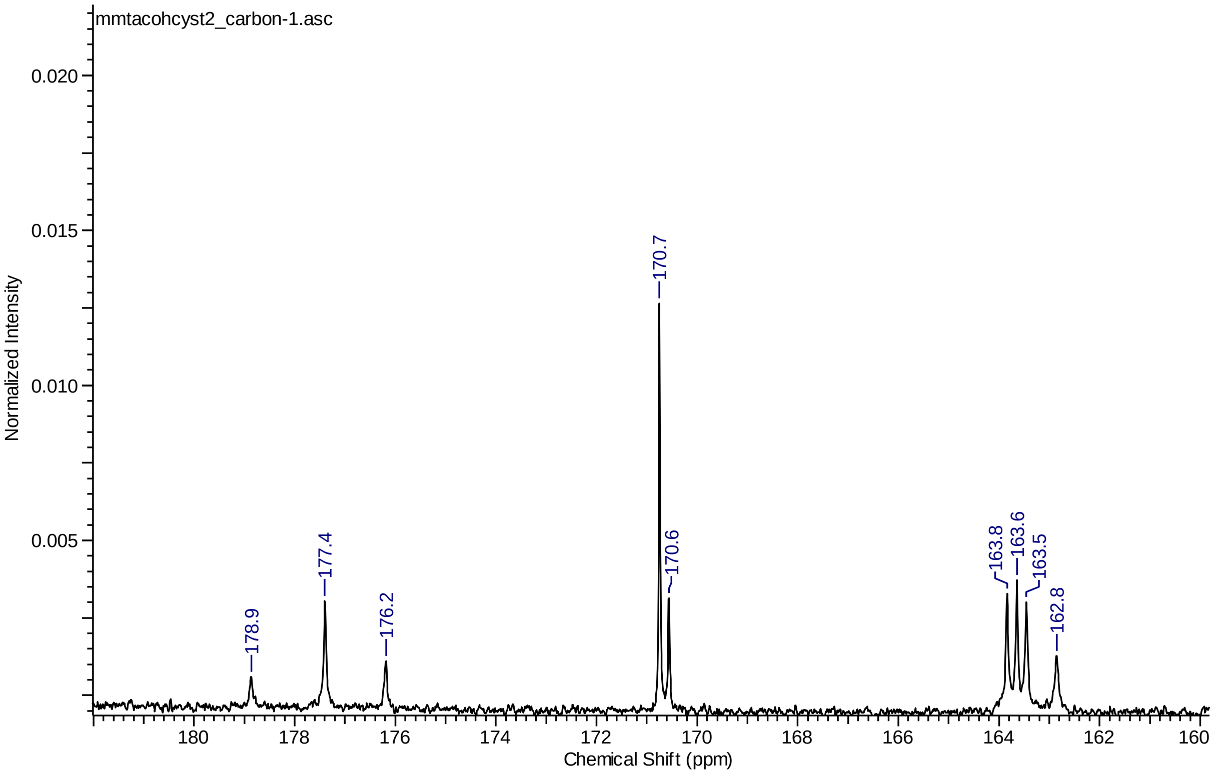

Figure S52. 13C{H} NMR region of carbonyl and 1,3,5-triazine carbon atoms of MMT(AcOH)(cysteamine)$_2$ in DMSO-d_6

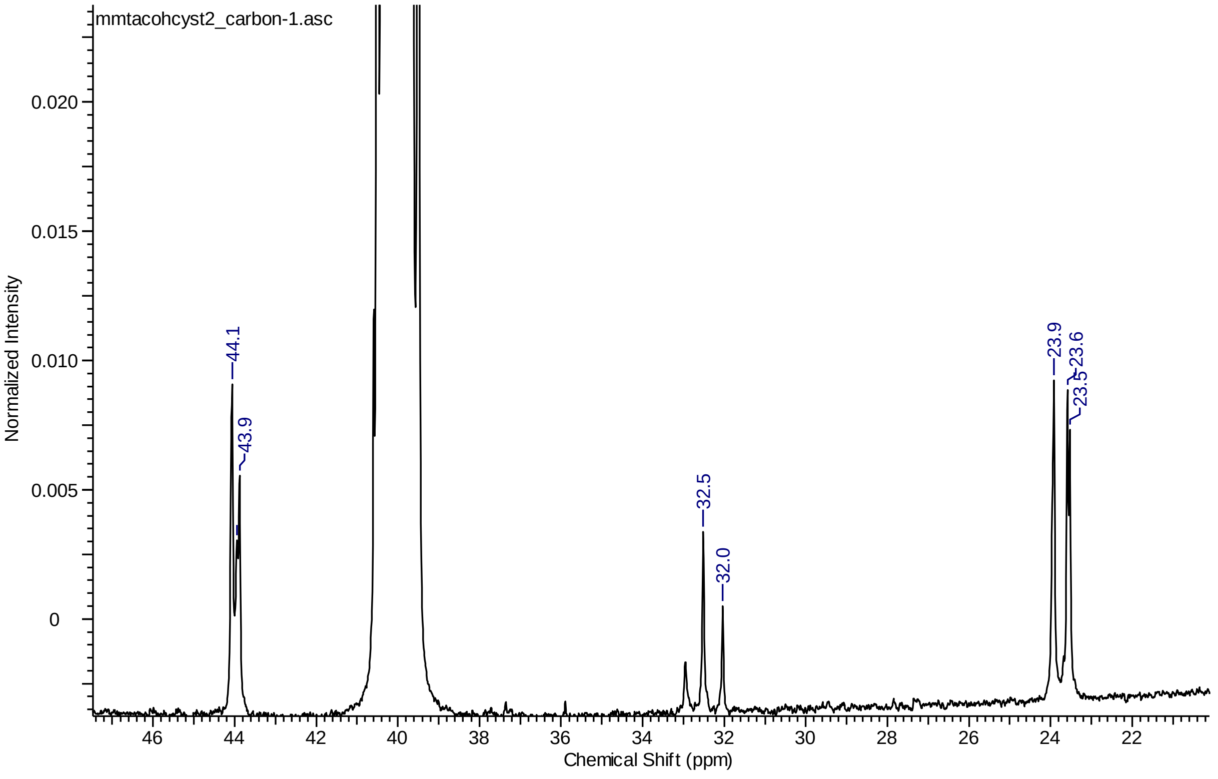

Figure S53. 13C{H} NMR region of aliphatic carbon atoms of MMT(AcOH)(cysteamine)$_2$ in DMSO-d_6

4.1.3. Reaction of tris(carboxymethyl)thiocyanurate with cysteine

Synthesis: 100 mg TMT(AcOH)$_3$ (0.3 mmol) and 121 mg cysteine (1 mmol) were dissolved in 5 mL of 0.5 M TEAB containing 20 mM TCEP. The reaction mixture was stirred on a rotary shaker for 24 hours at room temperature. Progress of the reaction was checked by RP-HPLC analysis with detection at 240 nm. Samples for the analysis were prepared by 100-fold dilution of the reaction mixture with water. Final mixture was acidified to pH 2 by TFA, and lyophilized. Crude product was purified using Varian ProStar HPLC system equipped with the TOSOH Bioscience TSKgel ODS 120T column (21.5 mm x 300 mm; 10µm), typically using the following solvent systems: 1%–70% B in A for 40 min, A 0.1% aqueous TFA, B 80% acetonitrile + 0.1% TFA, flow rate 7.0 ml/min. Collected fractions were characterized by direct ESI-MS, and lyophilized. Purity of the product was confirmed by analytical RP-HPLC.

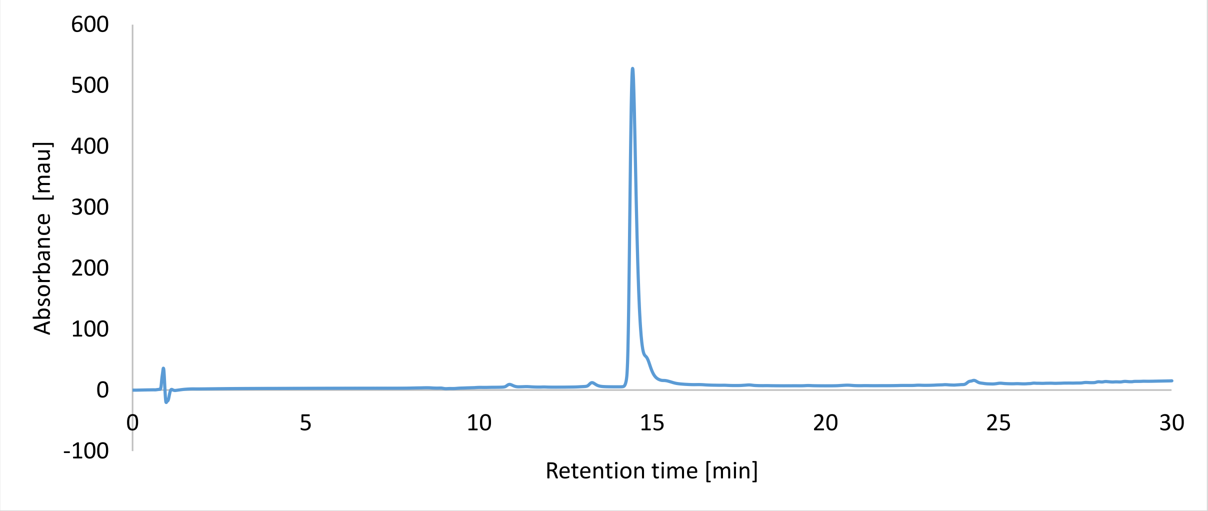

Figure S54. RP-HPLC profile of the mixture after reaction of an excess of cysteine with TMT(AcOH)$_3$.

DMT(AcOH)$_2$(Cys) - *N*-[4,6-bis(carboxymethylsulfanyl)-1,3,5-triazin-2-yl]-*L*-cysteine

HPLC: 14.3 min (column – Aeris 3.6 µm PEPTIDE XB-C18 50 x 2.1 mm; detection -D, 240 nm; gradient - 1%B for 5 min, 10-30%B 6-20 min, 30-100%B 21-25 min; flowrate 0.2 mL/min; eluent A – 0.1% formic acid in water, eluent B – 0.1% formic acid in acetonitrile)

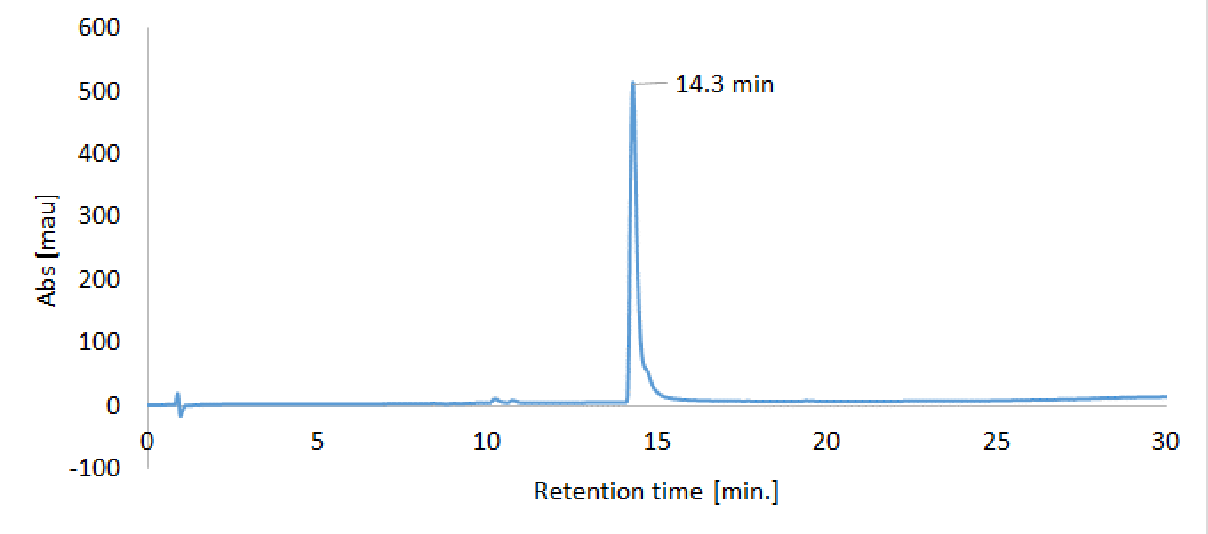

Figure S55. RP-HPLC chromatogram of purified DMT(AcOH)$_2$(Cys)

HRMS (ESI-MS) m/z: $[M-H_2O+H]^+$ Calcd for $C_{10}H_{11}N_4O_5S_3$ 362.9892; Found 362.9828, $[M+H]^+$ Calcd for $C_{10}H_{13}N_4O_6S_3$ 380.9992; Found 380.9980, $[M+Na]^+$ Calcd for $C_{10}H_{12}N_4O_6S_3Na$ 402.9811; Found 402.9822

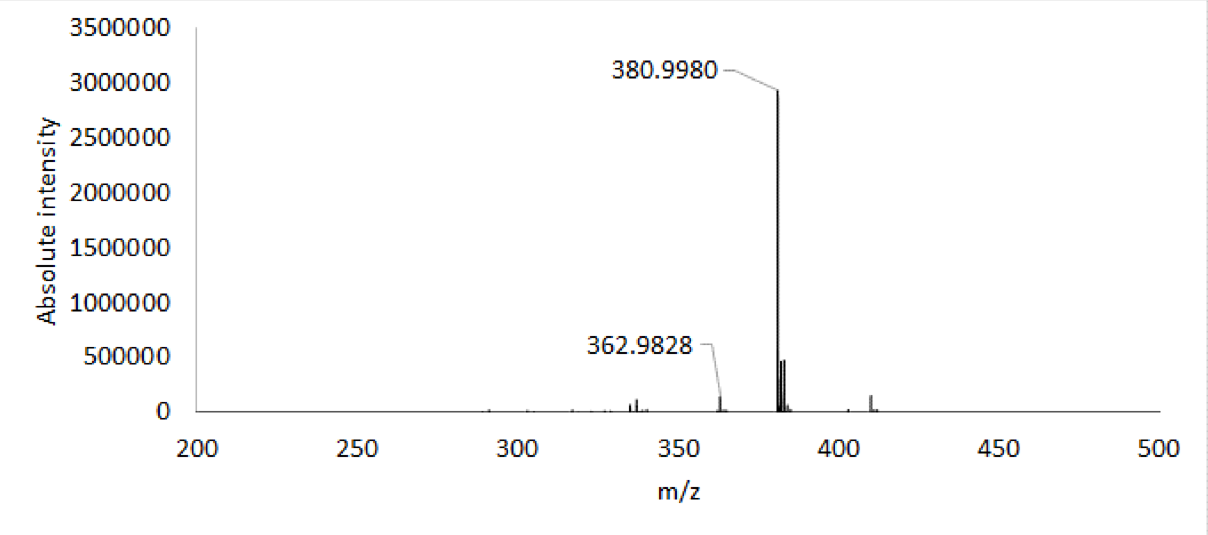

Figure S56. ESI-MS spectrum in positive ion mode of DMT(AcOH)$_2$(Cys)

ESI-MS(+)/MS, m/z: 362.9828 ($[M-OH]^+$, Calcd 362.9892); 334.9910 ($[M-COOH]^+$, Calcd 334.9942); 316.9813 ($[M-H_2O-COOH]^+$, Calcd 316.9837); 304.9636 ($[M-OH-CH_2CO_2]^+$, Calcd 304.9837); 288.9851 ($[M-SH-CH_2CO_2]^+$, Calcd 289.0060); 270.9836 ([$[M-H_2S-OH-CH_2CO_2]^+$, Calcd 270.9960), 258.9987 ($[M-C_3H_4O_2S-OH]^+$, Calcd 258.9960)

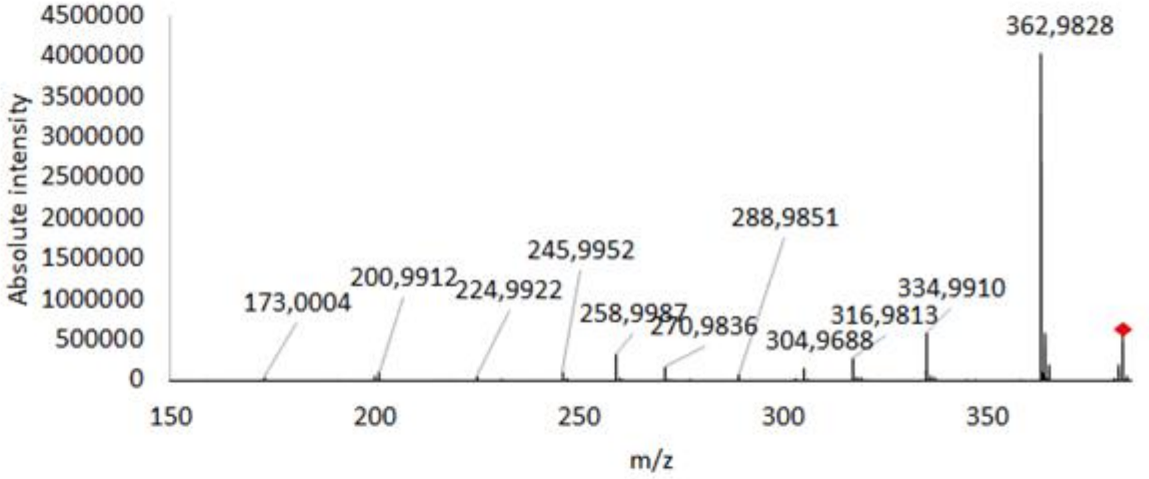

Figure S57. CID fragmentation MS^2 spectrum of DMT(AcOH)(Cys), parent ion $[M+H]^+$

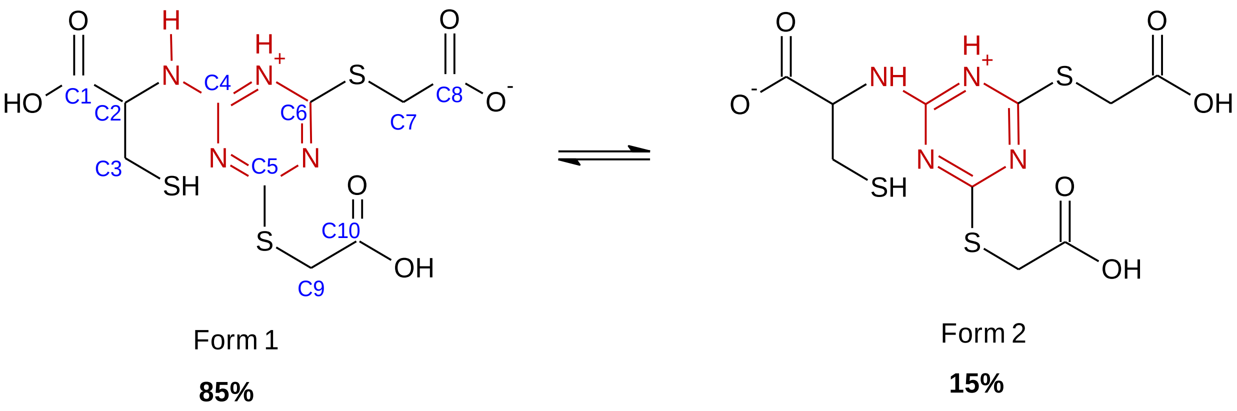

Scheme S2. Possible ionic forms of DMT(AcOH)$_2$(Cys) responsible for differentiating of 1H and 13C chemical shifts

1H NMR (DMSO-d6, 500 MHz, 25°C) δ (ppm) = 8.42** (d, NH, Cys, form 2) , 8.28 (d, 1H, NH, Cys, form 1), 4.69** (m, HA, Cys, form 2), 4.56 (m, 1H, HA, Cys, form 1), 3.91 (d, 2H, H.A#, SAc), 3.85 (d, 2H, HA#, SAc), 3.23** (dd, HB1, Cys, form 2), 3.04** (dd, HB2, Cys, form 2), 2.98 (m, 1H, HB1, Cys, form 1), 2.82 (m, 1H, HB2, Cys, form 1), 2.55 (t, 1H, SH, Cys)

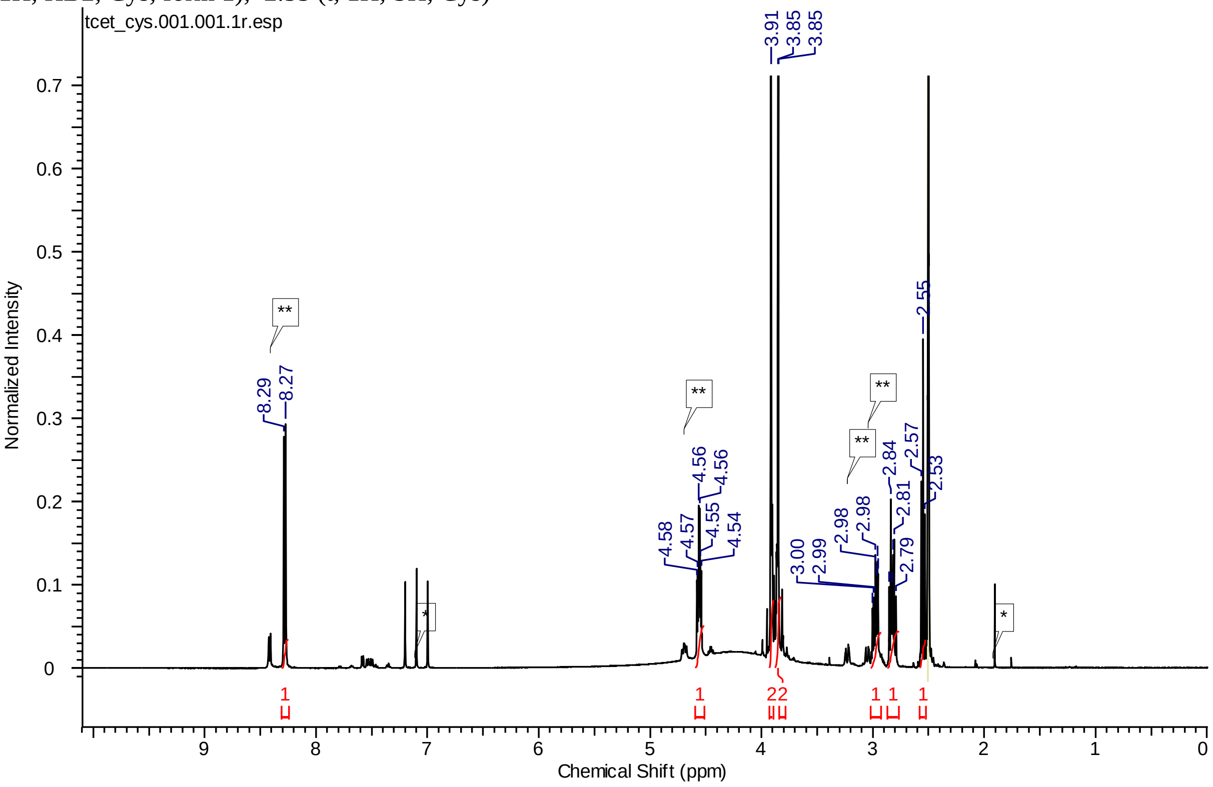

Figure S58. 1H NMR spectrum of DMT(AcOH)$_2$(Cys) in DMSO-d$_6$

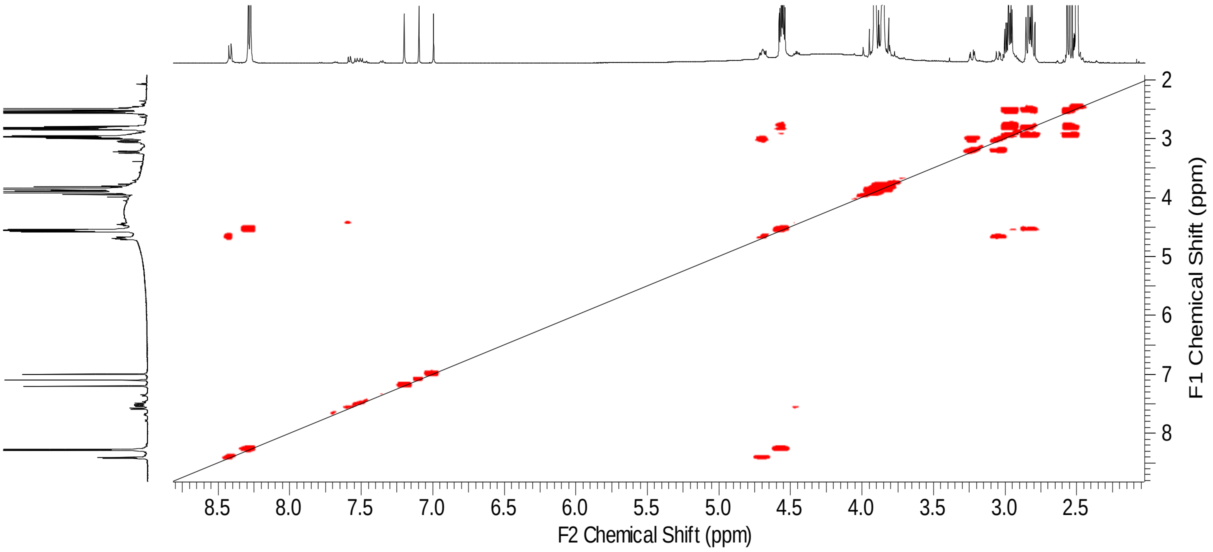

Figure S59. COSY spectrum of DMT(AcOH)$_2$(Cys) in DMSO-d$_6$

13C{H} NMR (DMSO-d6, 125 MHz, 25°C) δ (ppm) = 178.2 (C5, form 1), 178.2 (C5, form 2), 177.8 (C6, form 2), 177.8 (C6, form 1), 171.7 (C1, form 2), 171.3 (C1, form 1), 169.9 (C10/C8, form 2), 169.8 (C10/C8, form 1), 162.2 (C4), 56.16 (C2, form 1), 52.5 (C2, form 2), 38.1 (C3, form 2), 32.4 (C7, form 1), 32.3 (C7, form 2), 32.1 (C9, form 2), 32.1 (C9, form 1), 25.2 (C3, form 1)

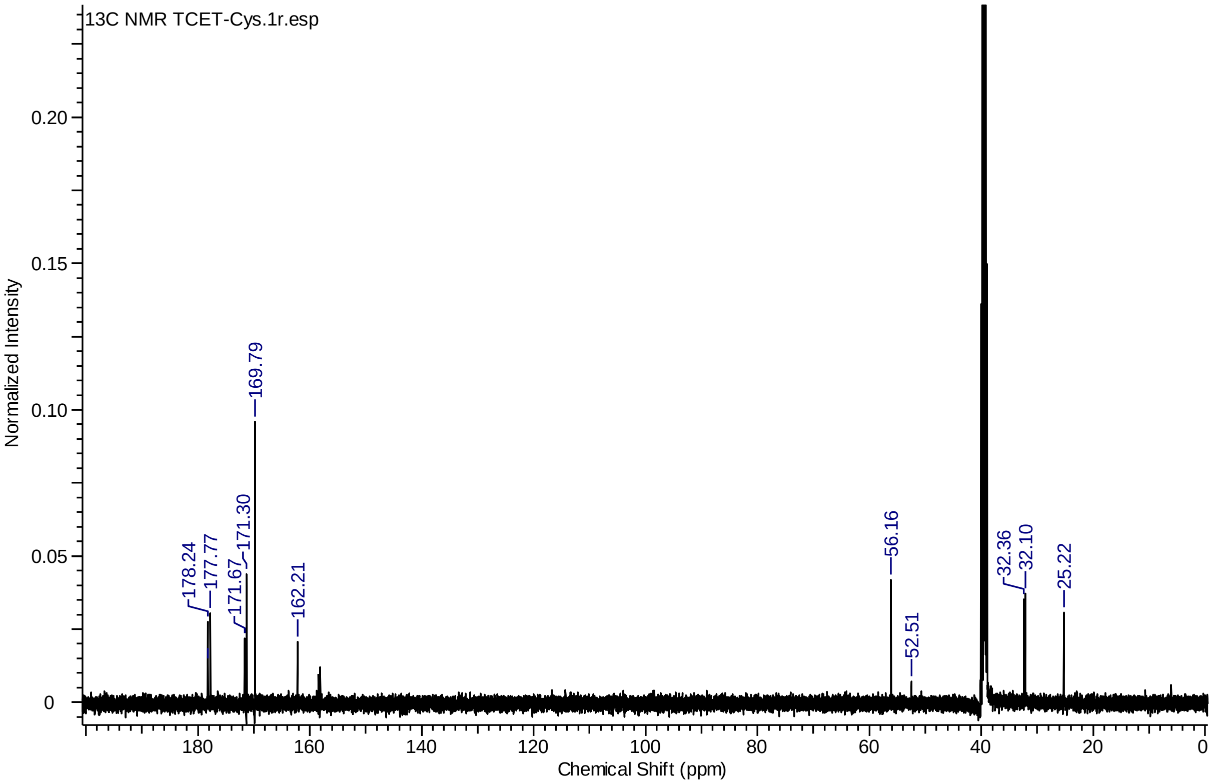

Figure S60. 13C{H} NMR spectrum of DTM(AcOH)$_2$(Cys) in DMSO-d$_6$

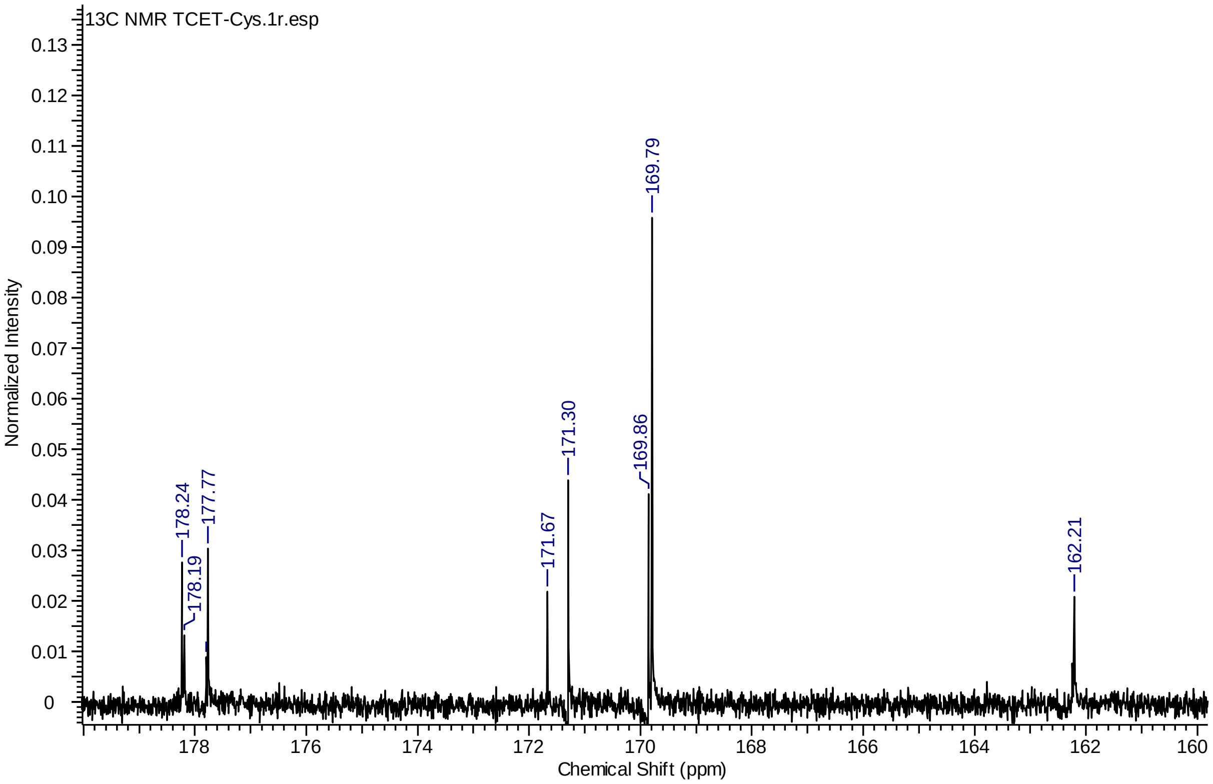

Figure S61. The region of carbonyl and aromatic carbon chemical shifts on the spectrum of DMT(AcOH)$_2$(Cys) in DMSO-d$_6$

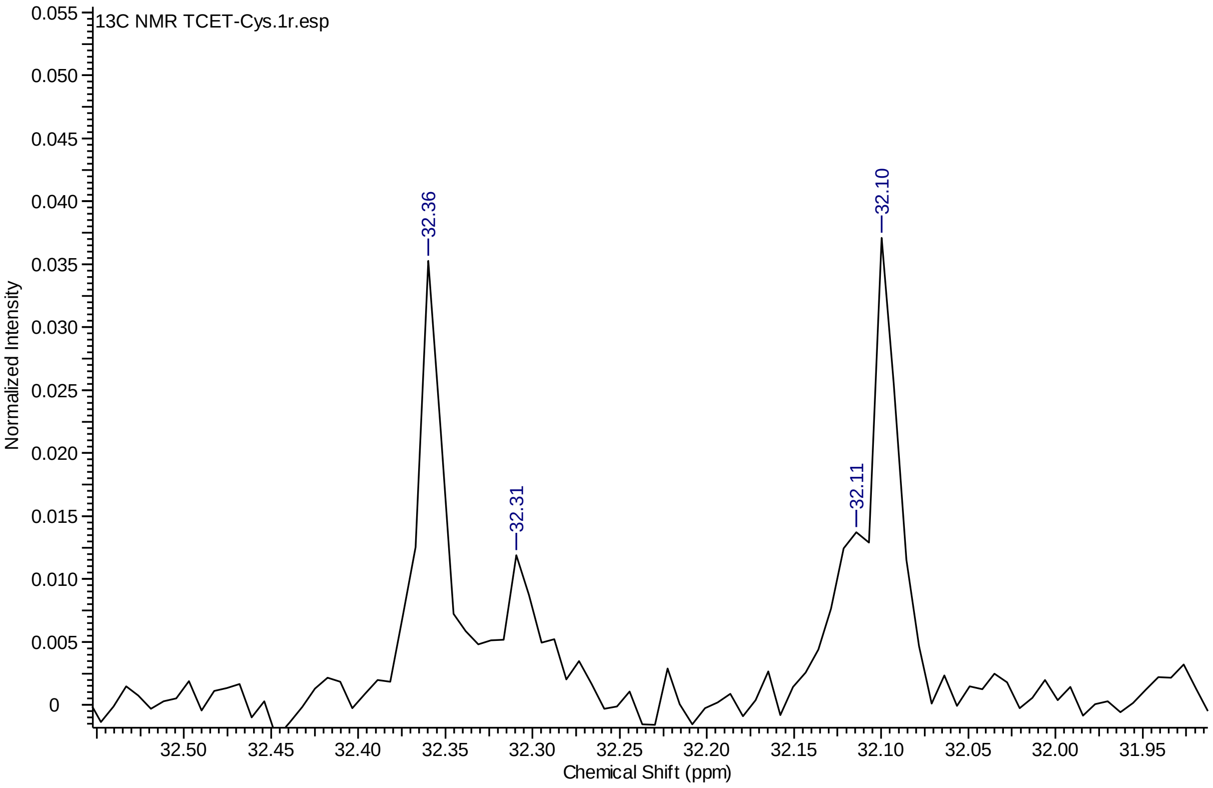

Figure S62. Signals of methylene group carbon atoms of thioglycolic acid residues of DMT(AcOH)(Cys) on 13C{H} NMR spectrum measured for sample dissolved in DMSO-d$_6$

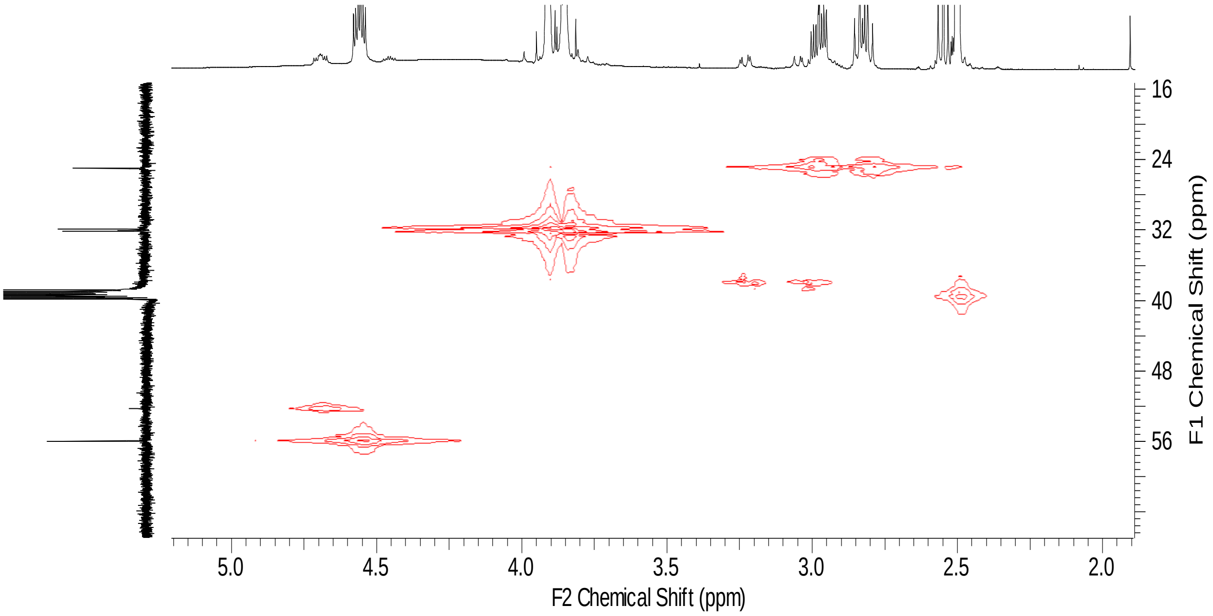

Figure S63. HSQC spectrum of DMT(AcOH)(Cys) in DMSO-d_6

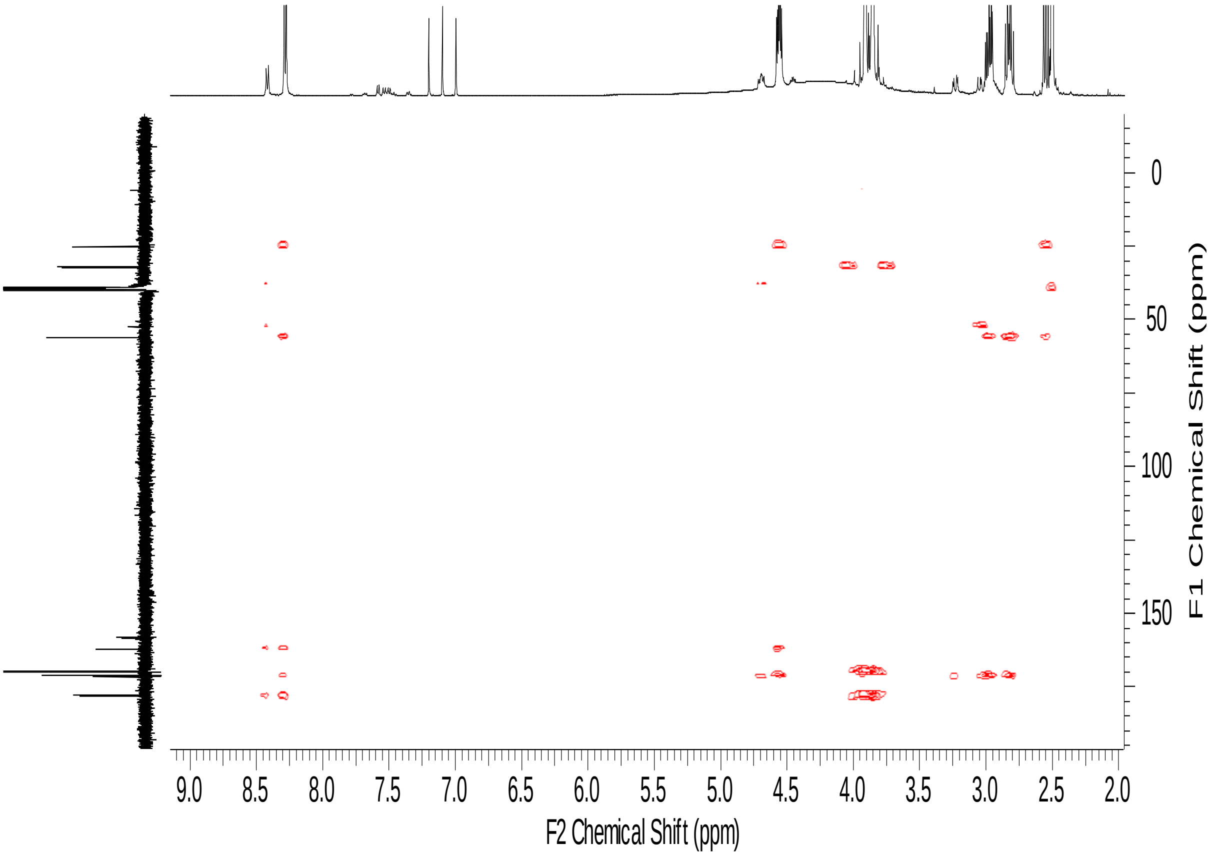

Figure S64. HMBC spectrum of DMT(AcOH)$_2$(Cys) in DMSO-d_6

4.1.4. Reaction of tris(carboxymethyl)thiocyanurate with penicillamine

Synthesis: 100 mg TMT(AcOH)$_3$ (0.3 mmol) and 150 mg penicillamine (1 mmol) were dissolved in 5 mL of 0.5 M TEAB containing 20 mM TCEP. The reaction mixture was stirred on a rotary shaker for 24 hours at room temperature. Progress of the reaction was checked by RP-HPLC analysis with detection at 240 nm. Samples for the analysis were prepared by 100-fold dilution of the reaction mixture with water. Purification as for DMT(AcOH)$_2$(Cys).

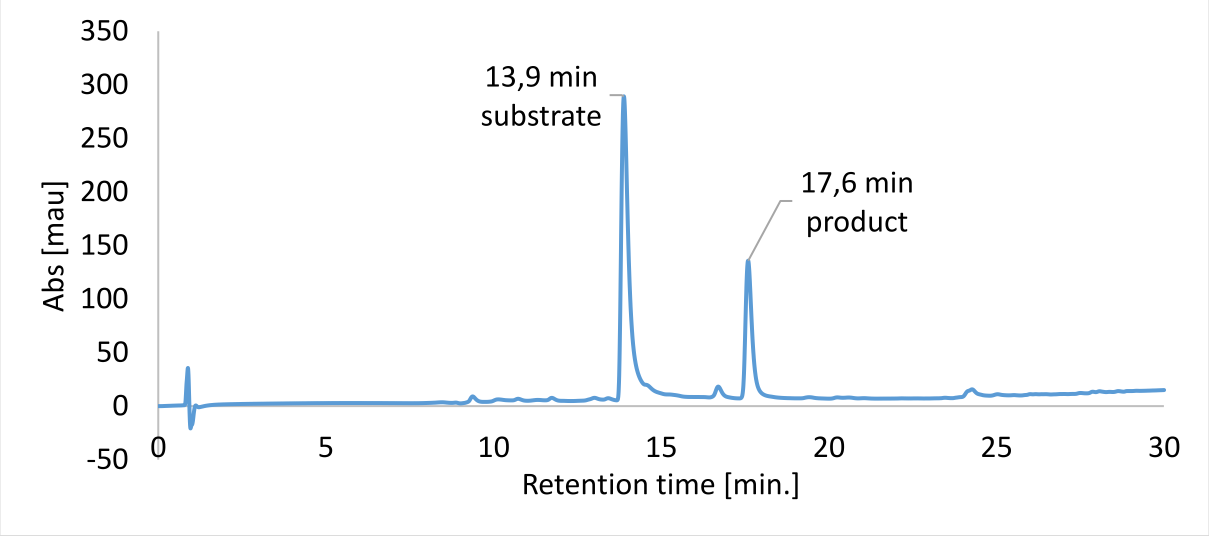

Figure S65. RP-HPLC profile of crude products mixture after reaction between an excess of L-penicilamine and TMT(AcOH)$_3$

DMT(AcOH)$_2$(Pen) - *N*-[4,6-bis(carboxymethylsulfanyl)-1,3,5-triazin-2-yl]-*L*-penicilamine

HPLC: 17.6 min (column – Aeris 3.6 µm PEPTIDE XB-C18 50 x 2.1 mm; detection -D, 240 nm; gradient - 1%B for 5 min, 10-30%B 6-20 min, 30-100%B 21-25 min; flowrate 0.2 mL/min; eluent A – 0.1% formic acid in water, eluent B – 0.1% formic acid in acetonitrile)

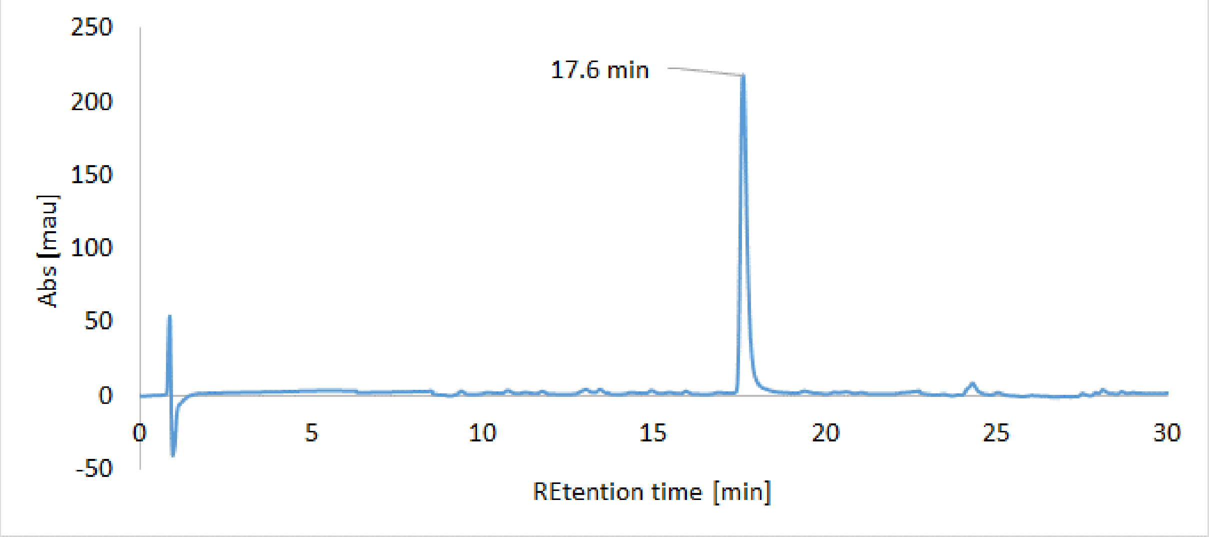

Figure S66. RP-HPLC chromatogram of purified DMT(AcOH)$_2$(Pen) in DMSO-d$_6$

HRMS (ESI-MS) m/z: $[M+H]^+$ Calcd for $C_{12}H_{17}N_4O_6S_3$ 409.0305; Found 409.0368, $[M+Na]^+$ Calcd for $C_{12}H_{16}N_4O_6S_3Na$ 431.0124; Found 431.0098.

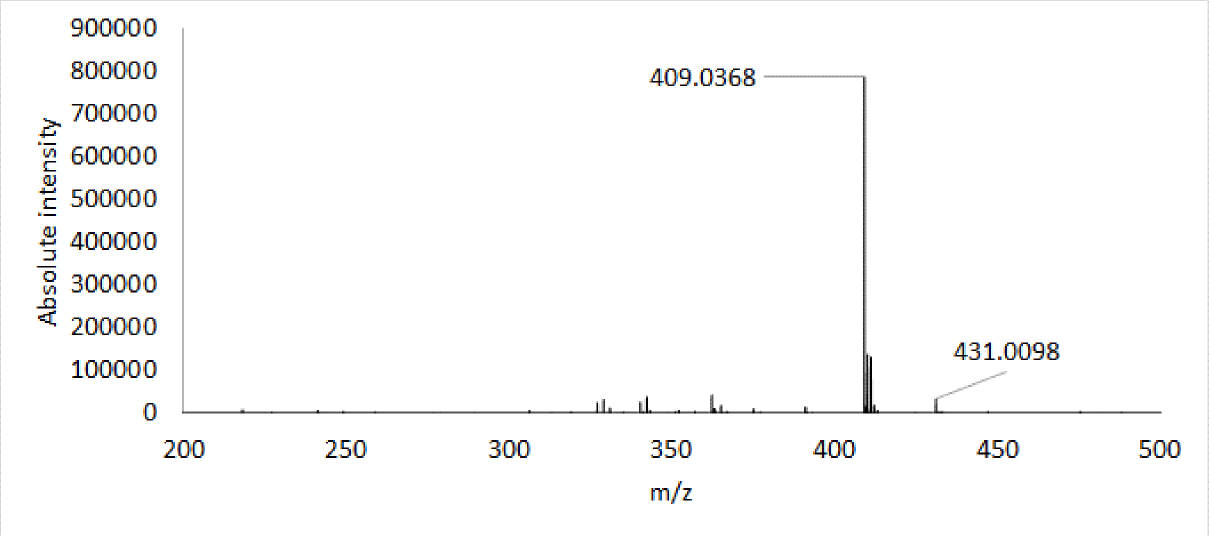

Figure S67. ESI-MS spectrum in positive ion mode of DMT(AcOH)$_2$(Pen)

ESI-MS(+)/MS: 391.0267 ([M-OH]$^+$, Calcd 391.0205); 375.0468 ([M-SH]$^+$, Calcd 375.0433); 363.0184 ([M-COOH]$^+$, Calcd 363.0255); 357.0336 ([M-H_2O-SH]$^+$, Calcd 357.0327); 329.0397 ([M-H_2S-COOH]$^+$, Calcd 329.0378); 258.9987 ([M-$C_5H_8O_2S$-OH]$^+$, Calcd 258.9960);

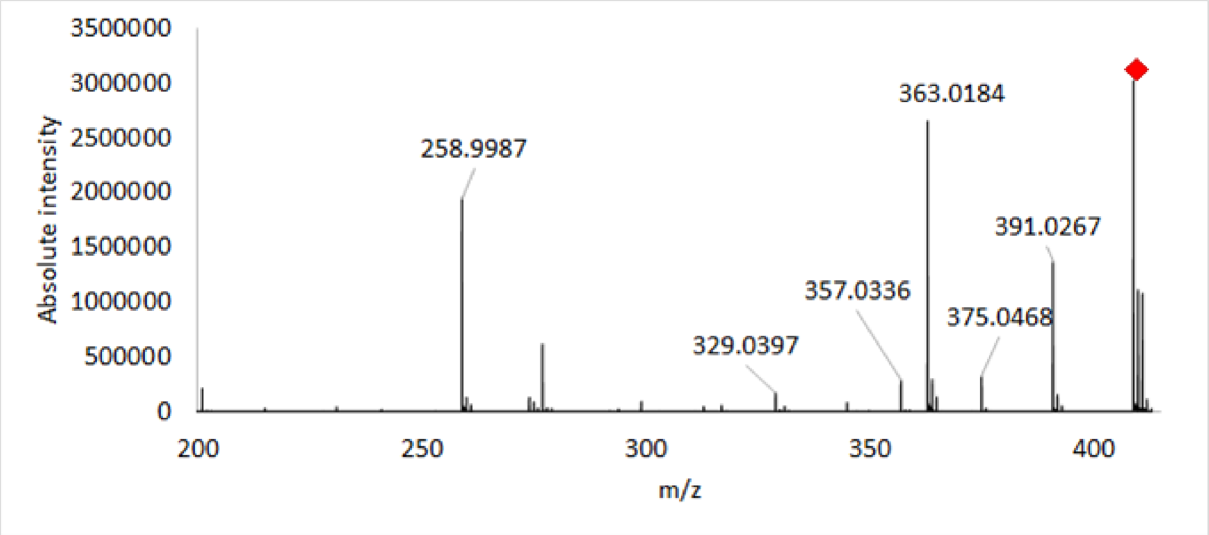

Figure S68. CID fragmentation MS^2 spectrum of DMT(AcOH)$_2$(Pen), [M+H]$^+$ parent ion.

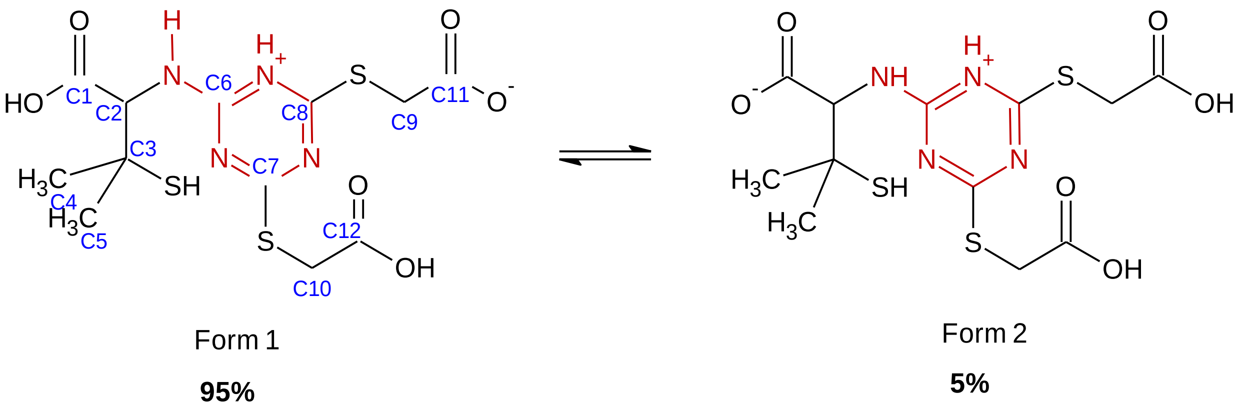

Scheme S3. Possible ionic forms of DMT(AcOH)₂(Pen) responsible for differentiating of 1H and 13C chemical shifts

1H NMR (DMSO-d6, 500 MHz, 25°C) δ (ppm) = 8.24 (d, NH, Pen, form 2), 8.17 (d, 1H, NH, Pen, form 1), 4.65 (d, 1H, HA, Pen, form 1), 4.59** (d, HA, Pen, form 2), 3.94 (dd, 2H, HB#, C9), 3.86 (dd, 2H, HB#, C10), 3.13 (s, 1H, SH, Pen), 1.46 (s, 3H, HB1, Pen), 1.43 (s, 3H, CB2, Pen),

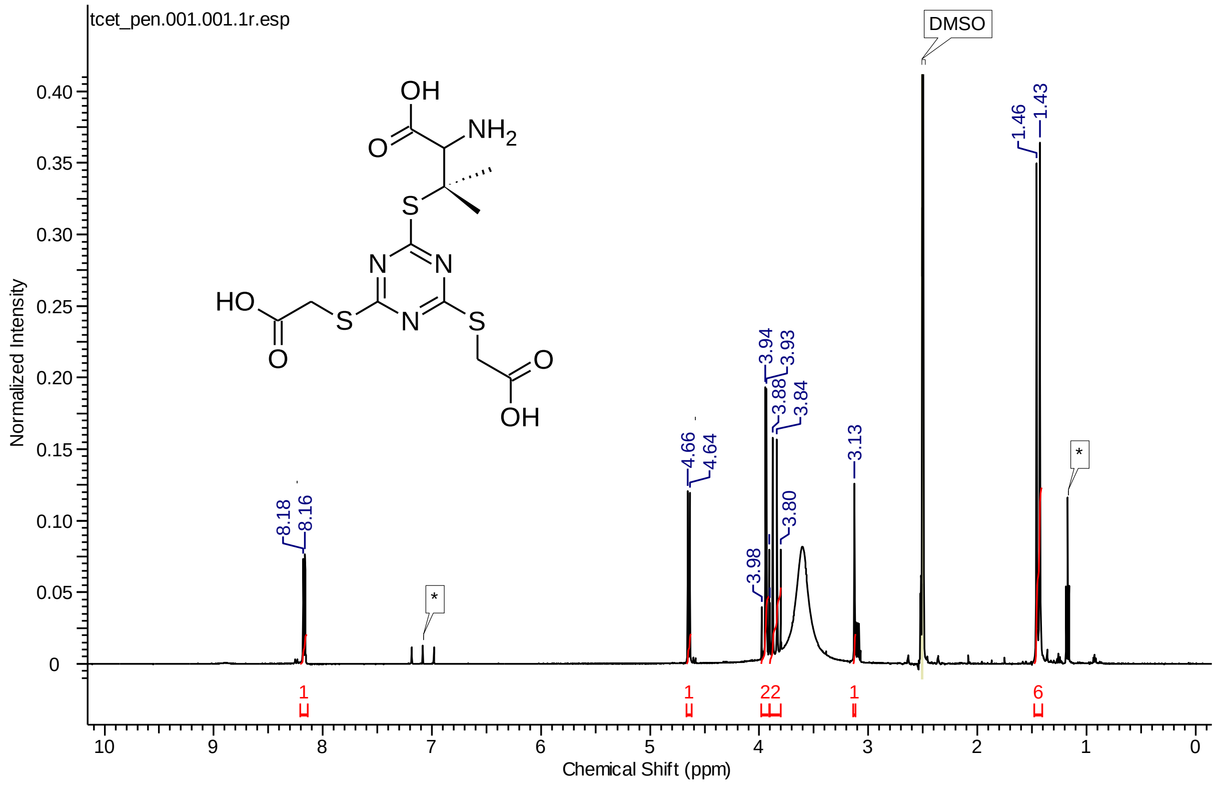

*Figure S69. 1H NMR spectrum of purified DMT(AcOH)$_2$(Pen) in DMSO-d$_6$. *solvent impurities*

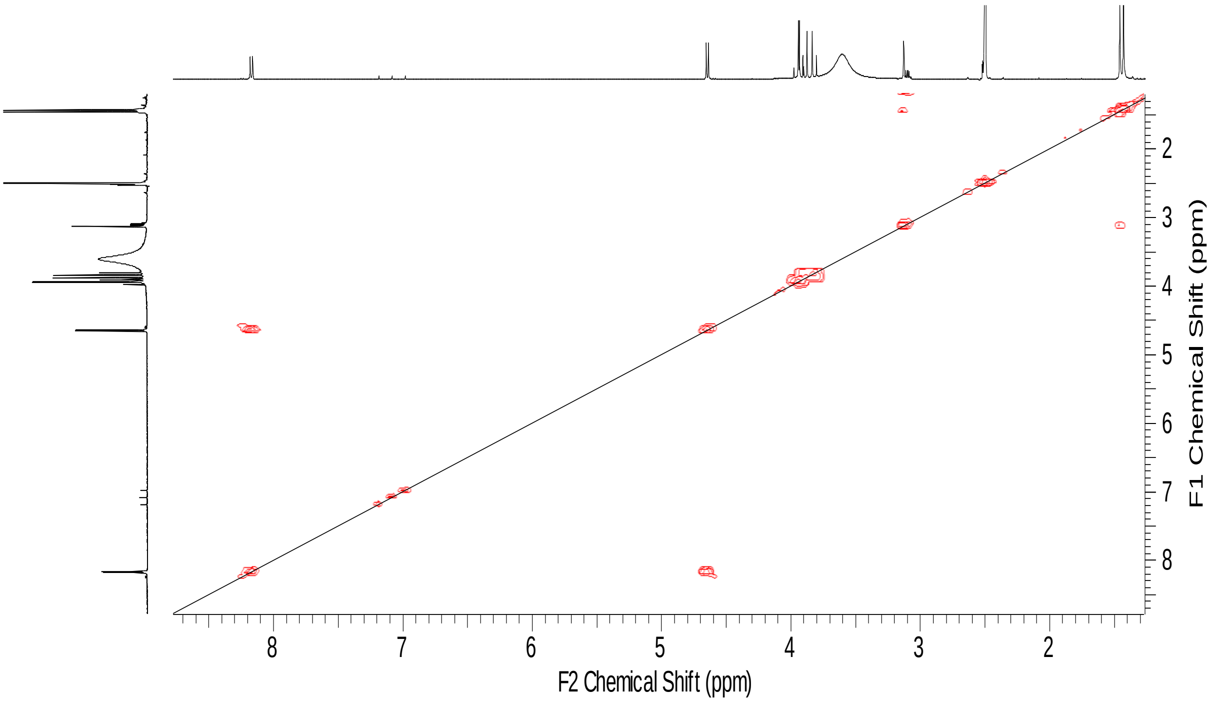

Figure S70. COSY spectrum of DMT(AcOH)$_2$(Pen) in DMSO-d$_6$

13C{H} NMR (DMSO-d6, 125 MHz, 25°C) δ (ppm) = 178.3 (C7), 177.8 (C8), 170.8 (C1), 169.8 (C11/C12), 169.8 (C11/C12), 162.3 (C6), 63.2 (C2), 45.2 (C3), 32.4 (C10), 32.1 (C9), 30.6 (C4/5), 29.1 (C4/5)

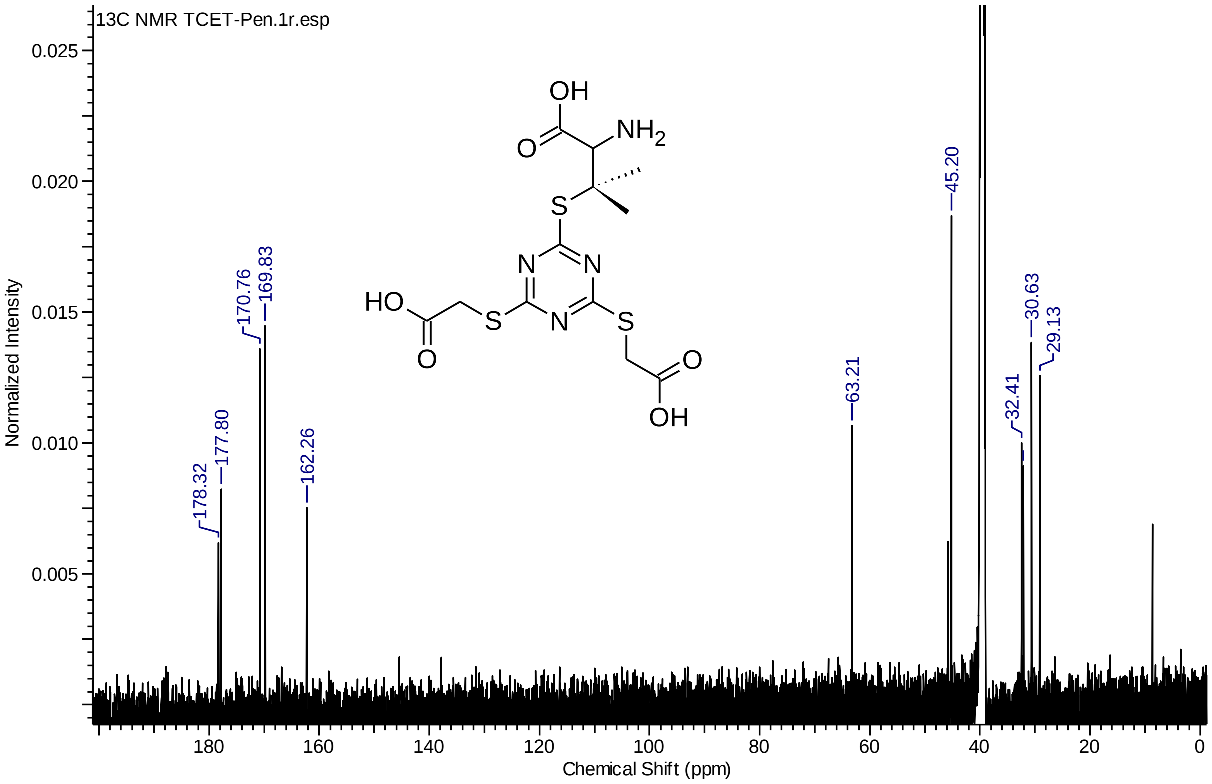

Figure S71. 13C NMR spectrum of DMT(AcOH)(Pen) in DMSO-d_6

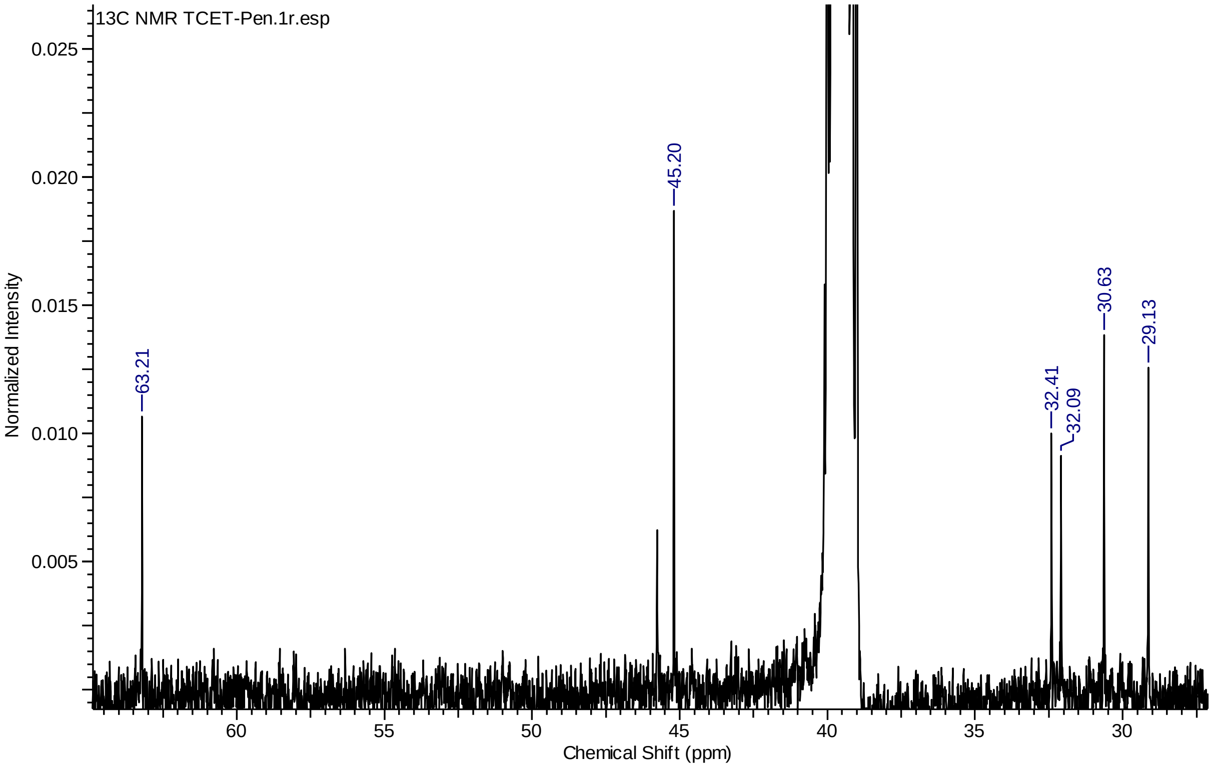

Figure S72. The aliphatic carbon shift region on 13C NMR spectrum of DMT(AcOH)$_2$(Pen) in DMOS-d_6

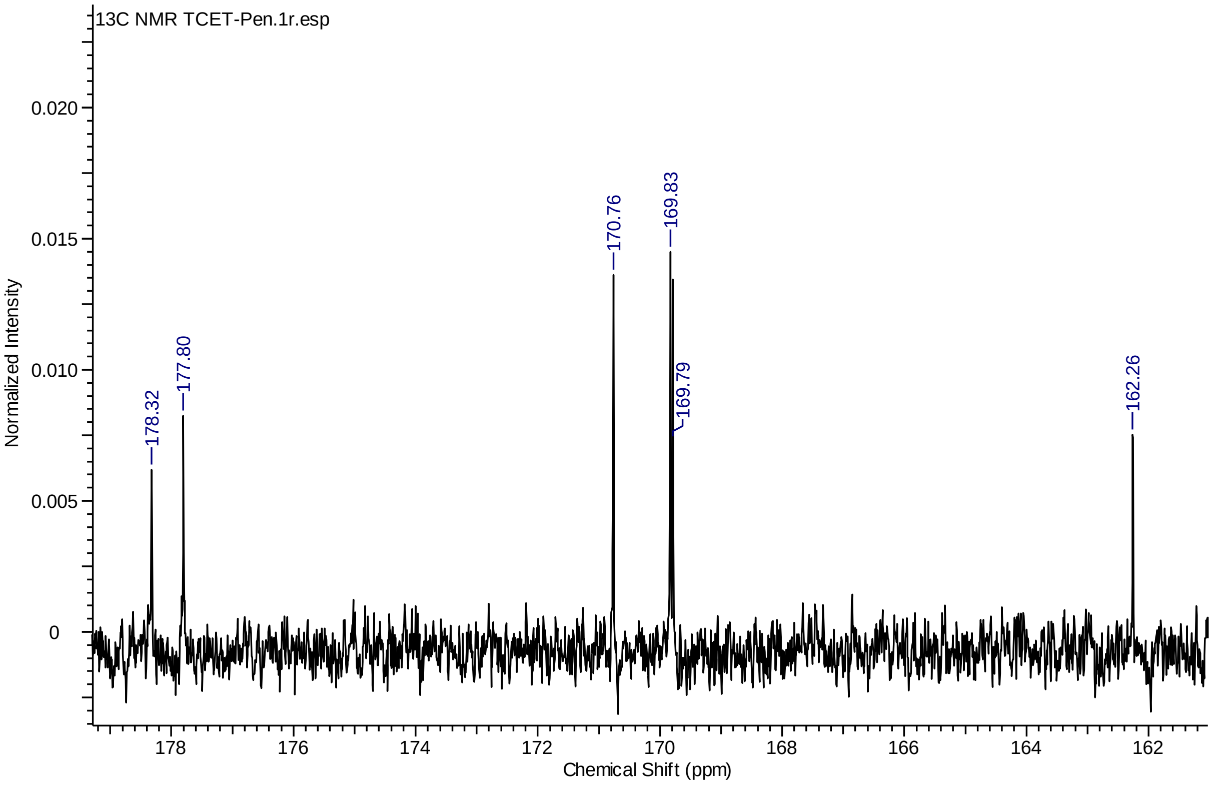

Figure S73. The aromatic carbon shift region on 13C NMR spectrum of DMT(AcOH)$_2$(Pen) in DMOS-d$_6$

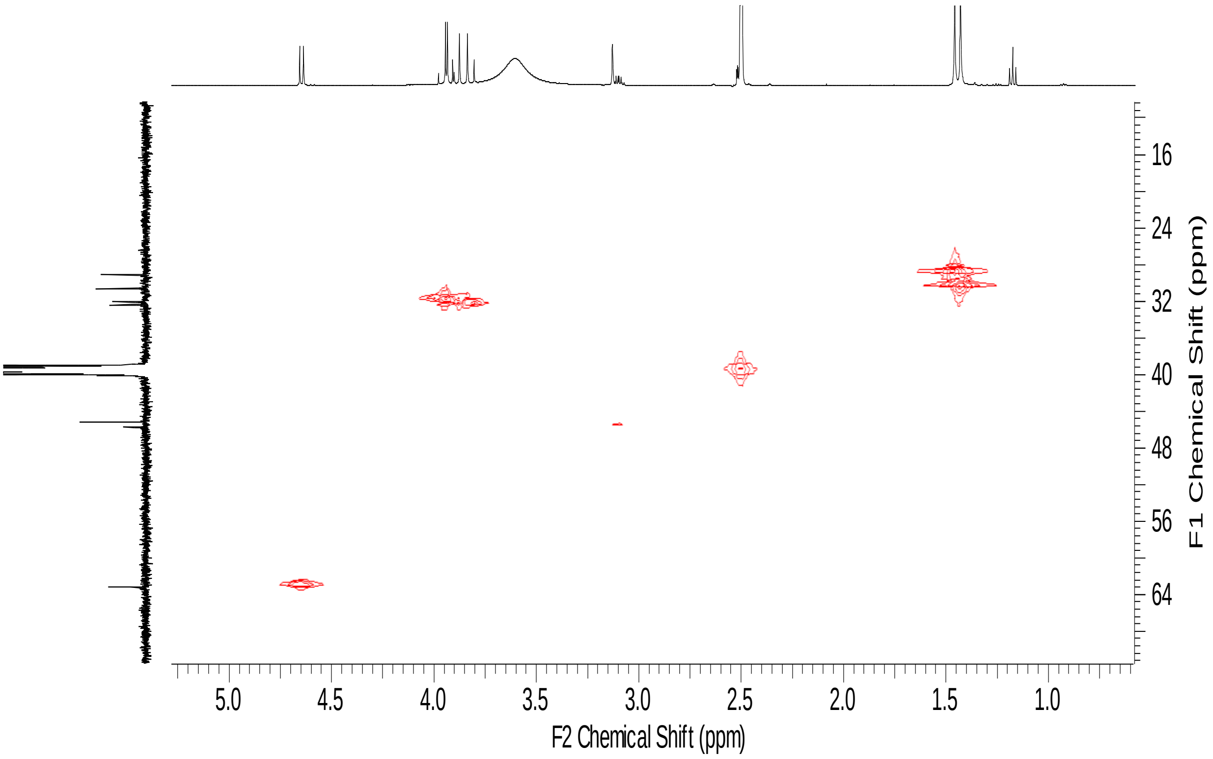

Figure S74. HSQC of DMT(AcOH)$_2$(Pen) in DMSO-d$_6$

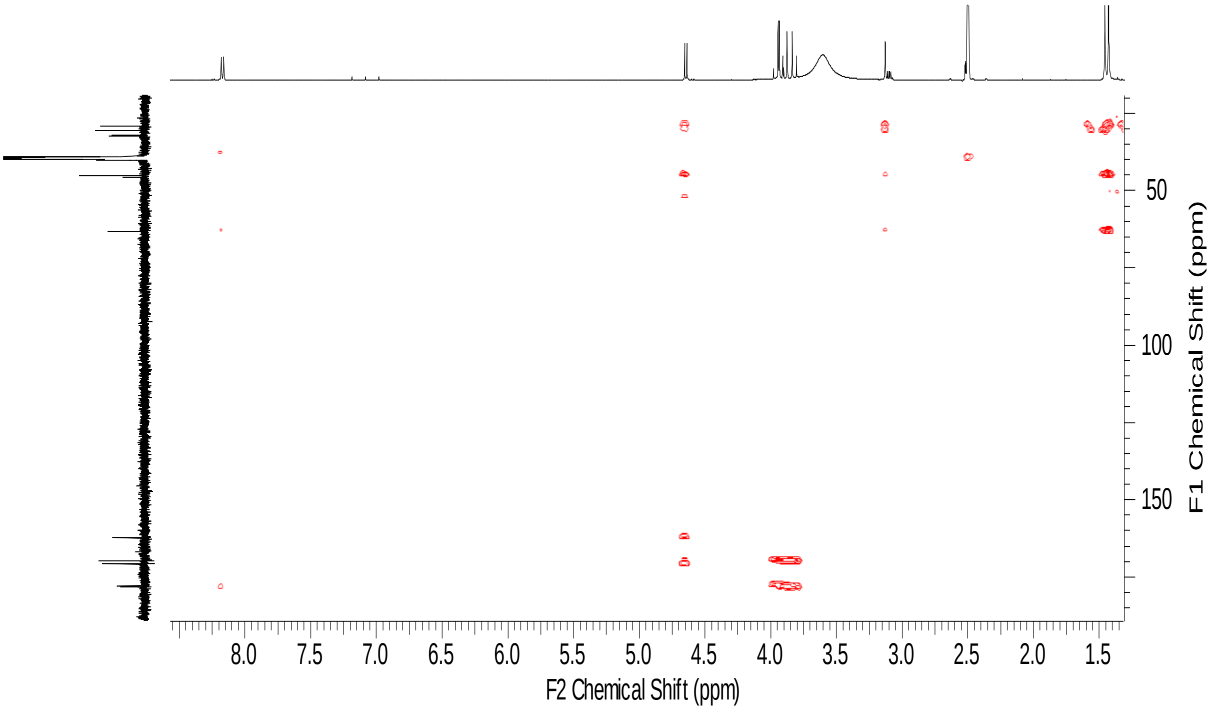

Figure S75. HMBC spectrum of DMT(AcOH)$_2$(Pen) in DMSO-d$_6$

4.1.5. Reaction tris(carboxymethyl)thiocyanurate with *D*-cysteine

DMT(AcOH)$_2$(*D*-Cys) - *N*-[4,6-bis(carboxymethylsulfanyl)-1,3,5-triazin-2-yl]-*D*-cysteine

Synthesis: 100 mg TMT(AcOH)$_3$ (0.3 mmol) and 121 mg *D*-cysteine (1 mmol) were dissolved in 10 mL of 0.5 M TEAB containing 20 mM TCEP. The reaction mixture was stirred on a rotary shaker for 24 hours at room temperature. Progress of the reaction was checked by RP-HPLC analysis with detection at 240 nm. Samples for the analysis were prepared by 100-fold dilution of the reaction mixture with water. Purification as for DMT(AcOH)$_2$(Cys).

HPLC: 8.8 min (column - ReproSil-XR 120 C18-MS 3 µm, 100 x 2 mm; detection -D, 240 nm; gradient – 1-70%B in 15 min; eluent A – 0.1% formic acid in the water, flowrate 0.2 mL/min; eluent B – 0.1% formic acid in acetonitrile)

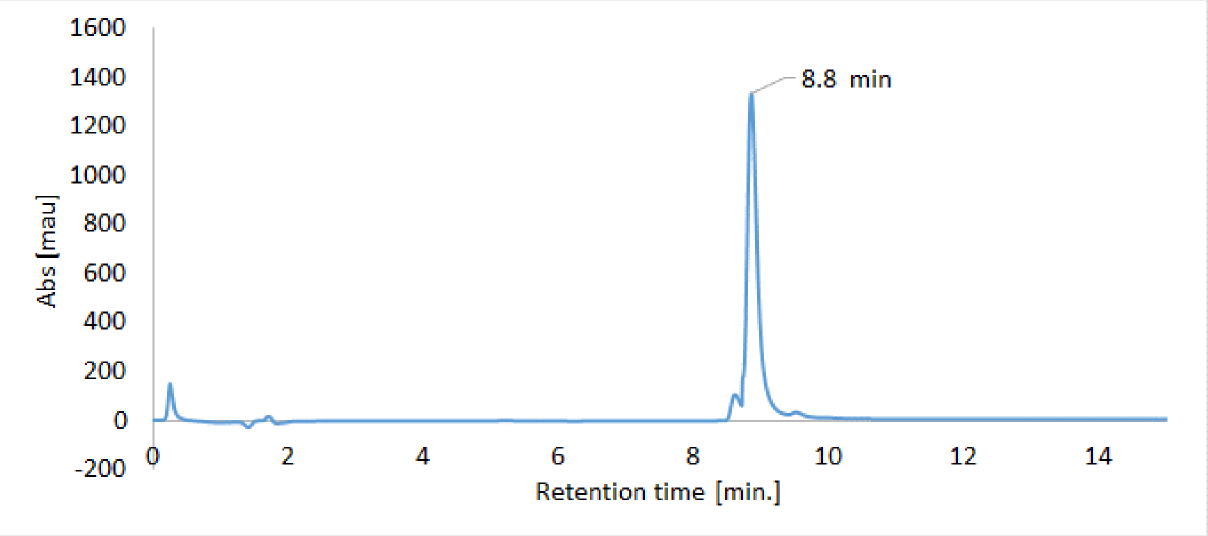

Figure S76. RP-HPLC chromatogram of purified DMT(AcOH)$_2$(D-Cys)

HRMS (ESI-MS) m/z: [M-H_2O+H]$^+$ Calcd for $C_{10}H_{11}N_4O_5S_3$ 362.9892; Found 362.9809, [M+H]$^+$ Calcd for $C_{10}H_{13}N_4O_6S_3$ 380.9992; Found 380.9968.

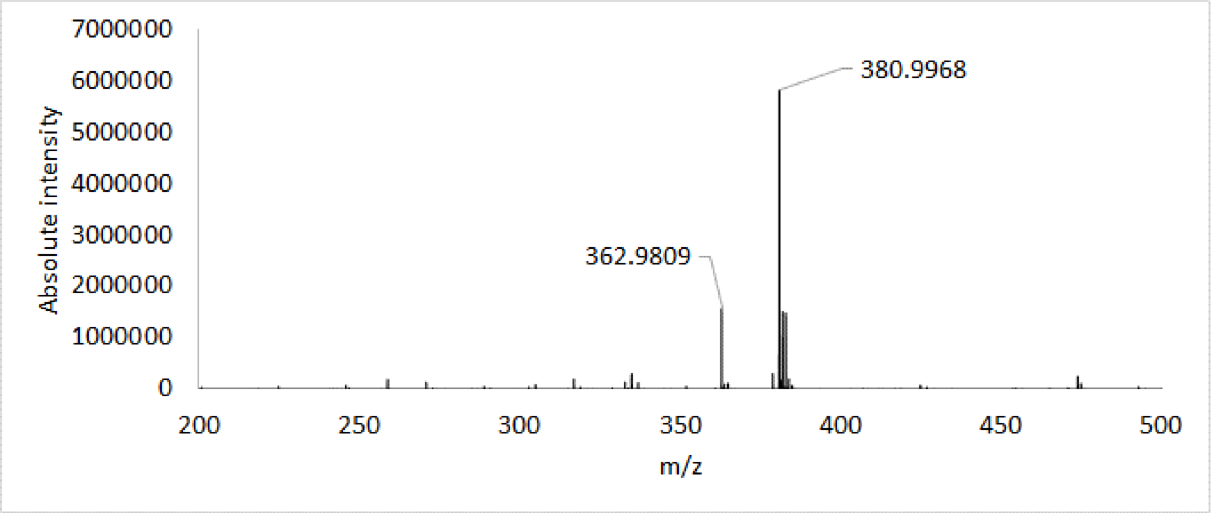

Figure S77. ESI-MS spectrum in positive ion mode of DMT(AcOH)$_2$(D-Cys)

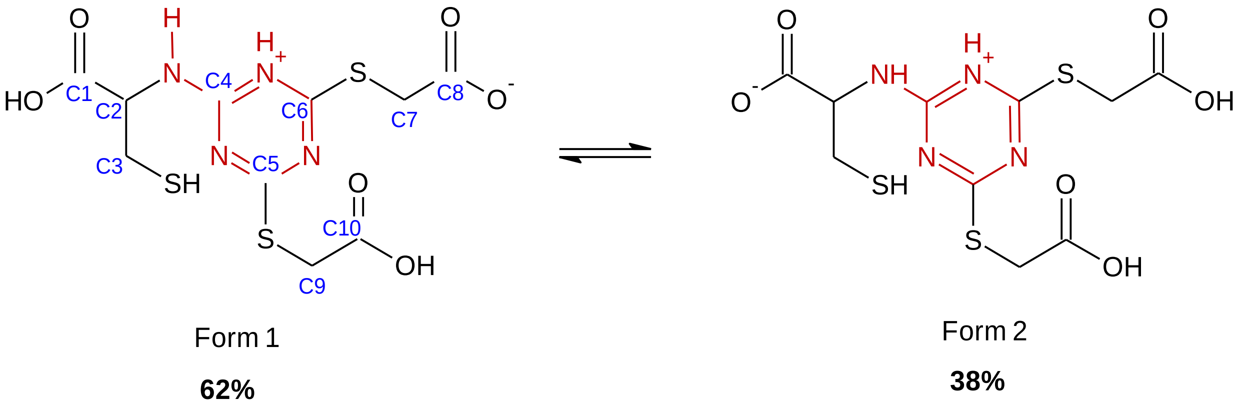

Scheme S4. Possible ionic forms of DMT(AcOH)2(D-Cys) responsible for differentiating of the 1H and 13C NMR shifts

1H NMR (DMSO-d6, 500 MHz, 25°C) δ (ppm) = 8.42** (d, NH, Cys, form 2), 8.28 (d, 1H, NH, Cys, form 1), 4.55 (m, 1H, HA, Cys, form 1), 4.68** (m, HA, Cys, form 2), 3.85-3.96 (d, 2H, HA#, SAc), 2.80-3.10 (m, 2H, HB#, Cys), 2.55 (t, 1H, SH, Cys)

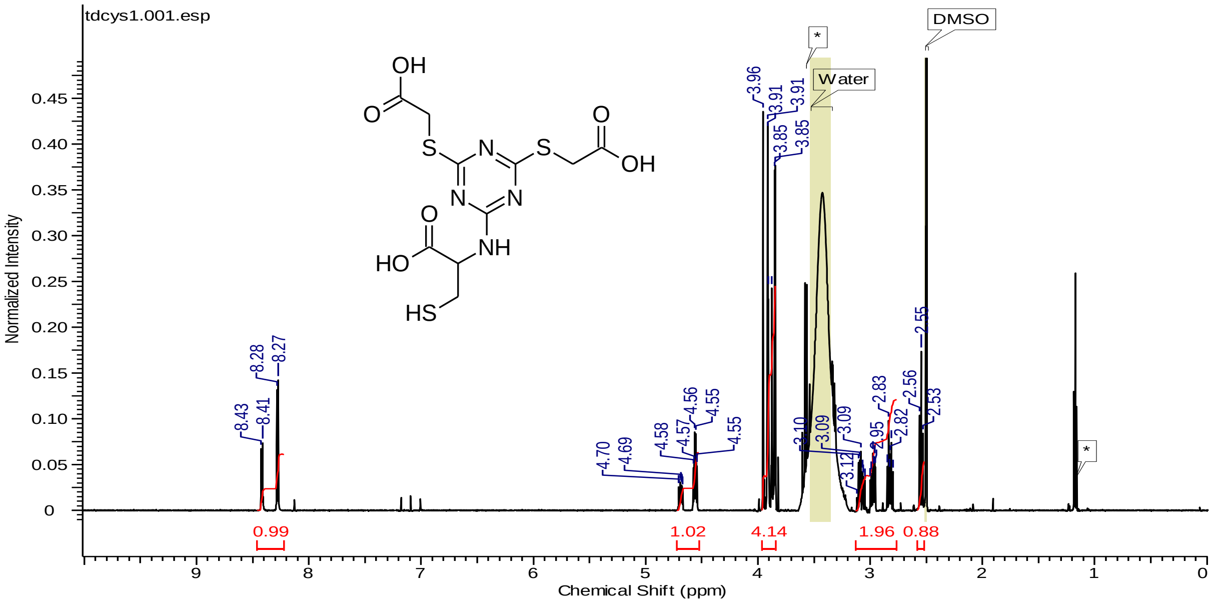

Figure S78. 1H NMR spectrum of DMT(AcOH)$_2$(D-Cys)

4.1. Reactions of tris(carboxymethyl)thiocyanurate with sulphides

General procedure: 105 mg tris(carboxymethyl)thiocyanurate (0.3 mmol) and 4 equiv. of an organic sulfide (1.2 mmol; 196mg pf AcCysOH or 369 mg of glutathione) were dissolved in 10 mL of 0.5M TEAB buffer containing 20 mM TCEP. The mixture was incubated at room temperature on a platform shaker. The progress of the reaction was monitored by HPLC using 254 nm as the wavelength of detection. After equilibrated of products, the final mixture was acidified TFA to pH 2, diluted with water, and lyophilized. Crude product was purified using Varian ProStar HPLC system equipped with the TOSOH Bioscience TSKgel ODS 120T column (21.5 mm x 300 mm; 10µm), typically using the following solvent systems: 1% for 10 min, 1–40% B in A for 60 min, A 0.1% aqueous TFA, B 80% acetonitrile + 0.1% TFA, flow rate 7.0 ml/min. Collected fractions were characterized by direct ESI-MS, and lyophilized. Purity of the product was confirmed by analytical RP-HPLC.

4.1.1. Reaction with Ac-Cys-OH

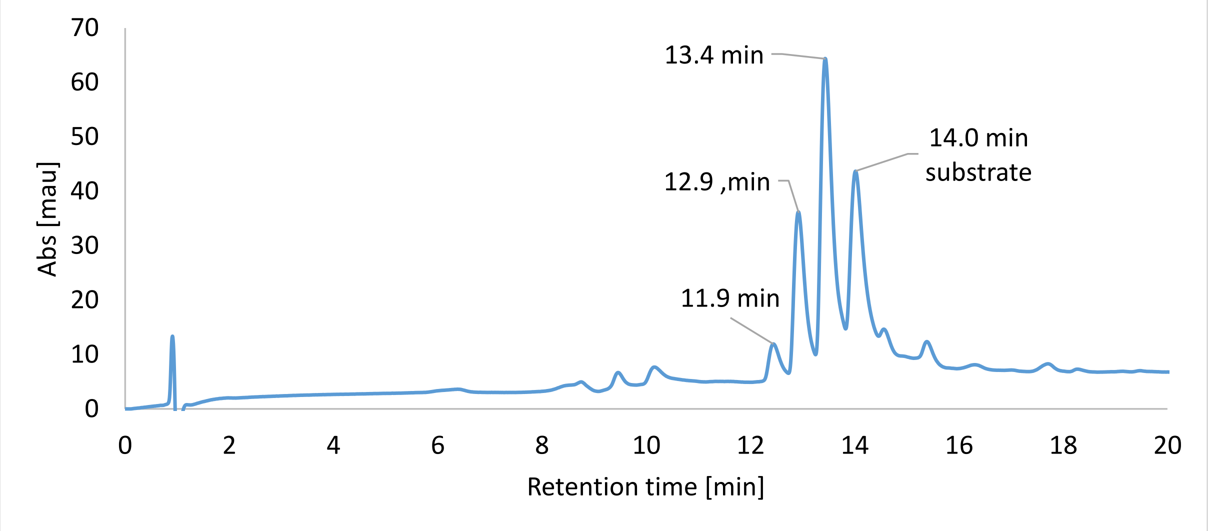

Figure S79. RP-HPLC frofile (detection at 254 nm) of the final mixture after reaction of an excess of AcCysOH with TMT(AcOH)$_3$: 14.01 min – TMT(AcOH)3; 13.43 min – TMT(AcOH)$_2$(AcCysOH); 12.91 min – TMT(AcOH)(AcCysOH)$_2$; 12.44 min – TMT(AcCysOH)$_3$.

TMT(AcOH)$_2$(AcCysOH) – 2-(acetylamino)-3-({4,6-bis[(carboxymethyl)sulfanyl]-1,3,5-triazin-2-yl)propanoic acid

HPLC: 13.1 min (column – Aeris 3.6 µm PEPTIDE XB-C18 50 x 2.1 mm; detection -D, 240 nm; gradient - 1%B for 5 min, 10-30%B 6-20 min, 30-100%B 21-25 min; flowrate 0.2 mL/min; eluent A – 0.1% formic acid in water, eluent B – 0.1% formic acid in acetonitrile)

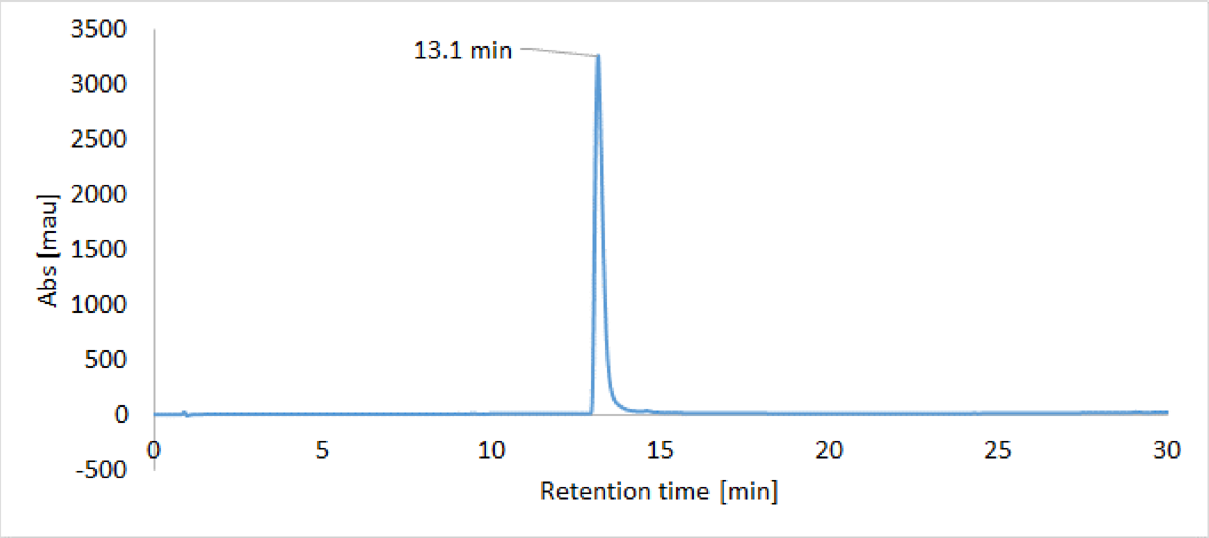

Figure S80. RP-HPLC chromatogram of purified TMT(AcOH)$_2$(AcCysOH)

HRMS (ESI-MS) m/z: $[M+H]^+$ Calcd for $C_{12}H_{15}N_4O_7S_3$ 423.0097; Found 423.0121, $[2M+H]^+$ Cacld for $C_{24}H_{29}N_8O_{14}S_6$ 845.0122; Found 845.0160

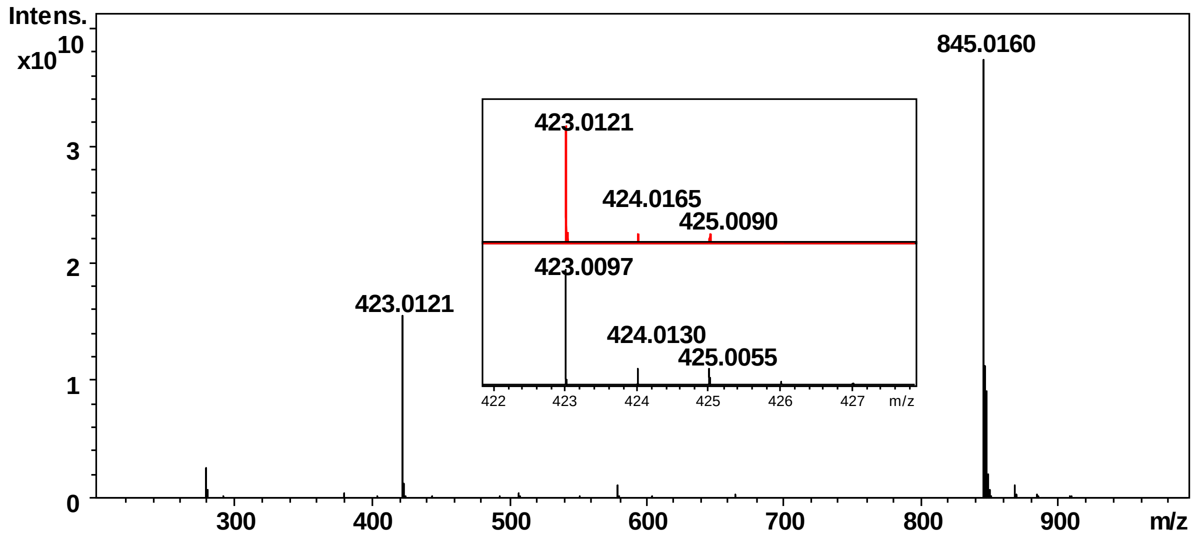

Figure S81. ESI-MS in positive ion mode of purified TMT(AcOH)$_2$(AcCysOH) and comparison of measured (red) and simulated (black) isotopic pattern of $[M+H]^+$ ion.

ESI-MS(+)/MS, m/z: 404.9892 ($[M-OH]^+$, Calcd 404.9997); 380.9978 ($[M-Ac+2H]^+$, Calcd 380.9991); 362.9826 ($[M-AcOH+2H]^+$, Calcd 362.9891); 293.9710 ($[M-Ac\Delta AlaOH+H]^+$, Calcd 293.9671); 275,9517 ($[M-Ac\Delta AlaOH-OH]^+$, Calcd 275.9571); 257,9324 ($[M-Ac\Delta AlaOH-H_2O-OH]^+$, Calcd 257.9466);

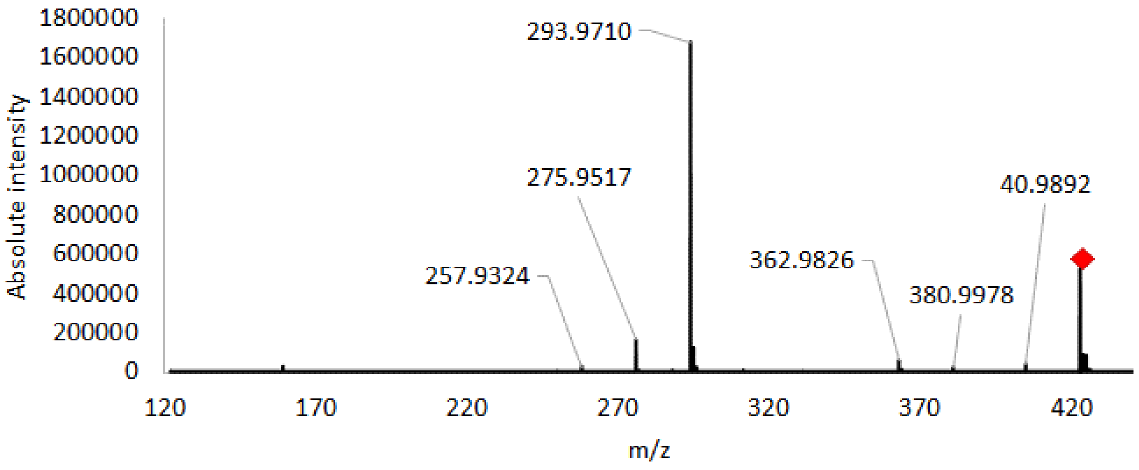

Figure S82. CID fragmentation MS^2 spectrum of TMT(AcOH)2(AcCysOH), [M+H]$^+$ parent ion

1H NMR (DMSO-d6, 500 MHz, 25°C) δ (ppm) = 8.36 (d, 1H, HN, Cys), 4.54 (m, 1H, HA, Cys), 3.97 (s, 4H, HA, SAc), 3.67 (dd, 1H, HB1, Cys), 3.27 (dd, 1H, HB2, Cys), 1.83 (s, 3H, Ac)

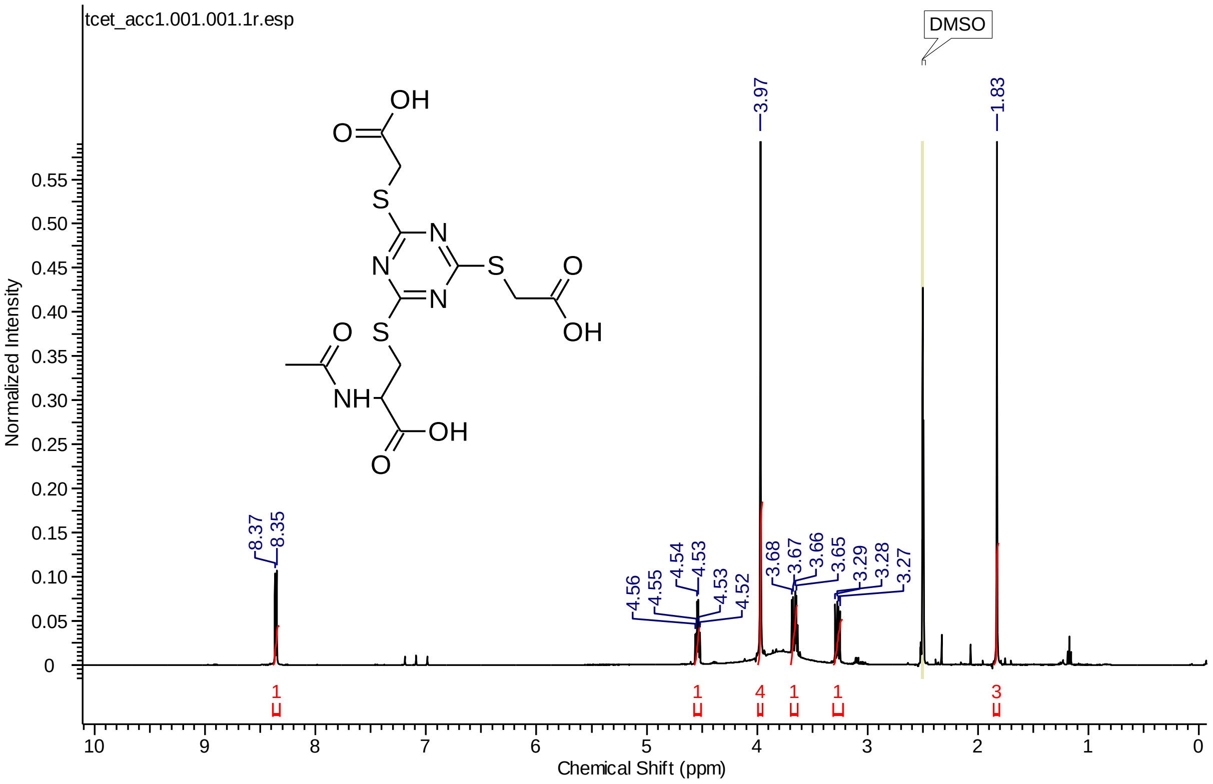

Figure S83. 1H NMR spectrum of TMT(AcOH)$_2$(AcCysOH) inDMSO-d$_6$

13C{H} NMR (DMSO-d6, 125 MHz, 25°C) δ (ppm) = 178.6 (C3), 178.3 (C1,C2), 171.6 (C8), 169.4 (C11), 169.4 (C4,C6), 51.1 (C9), 32.5 (C5,C7), 30.9 (C10), 22.3 (C12)

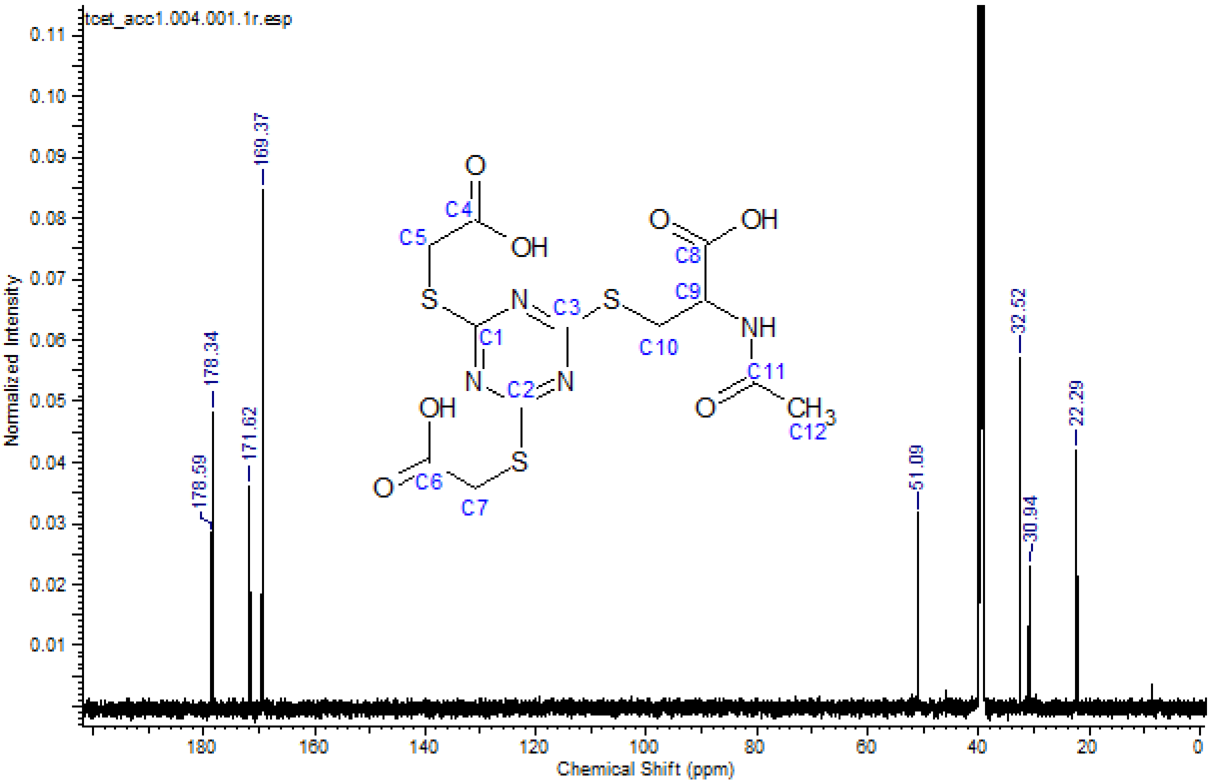

Figure S84. 13C{H} NMR spectrum of TMT(AcOH)$_2$(AcCysOH) in DMSO-d$_6$

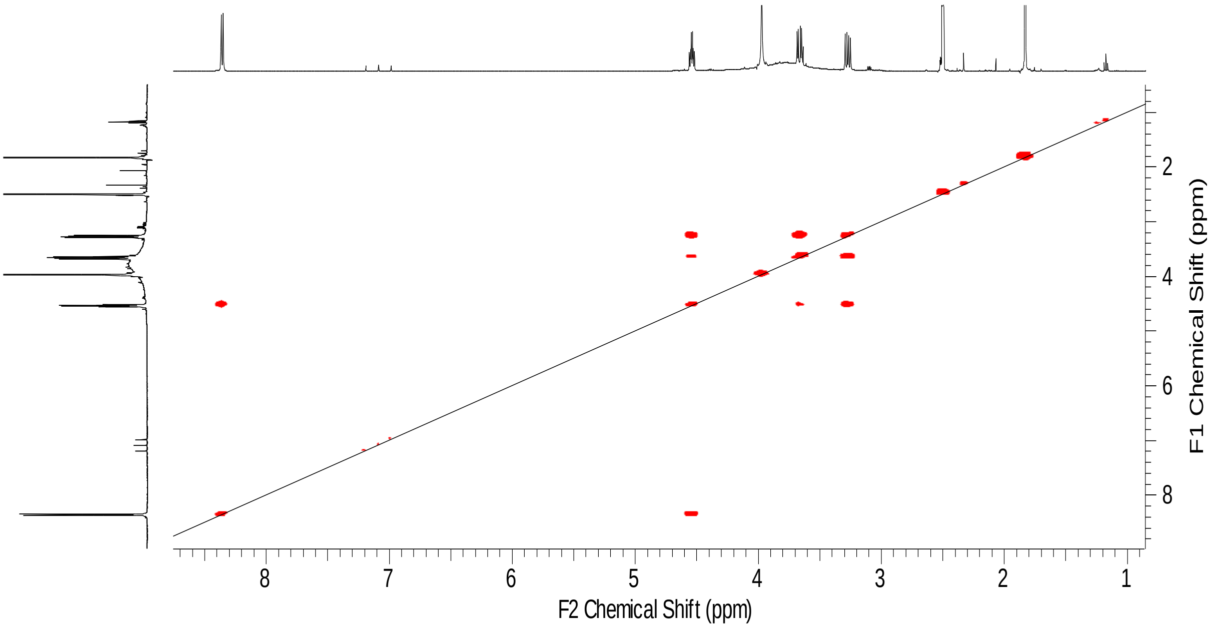

Figure S85. COSY spectrum of TMT(AcOH)$_2$(AcCysOH)

TMT(AcOH)(AcCysOH)$_2$ – 3,3’-({6-[(carboxymethyl)sulfanyl]-1,3,5-triazine-2,4-diyl}disulfanyl)bis[2-(acetylamino)propanoic acid]

HPLC: 12.7 min (column – Aeris 3.6 µm PEPTIDE XB-C18 50 x 2.1 mm; detection -D, 240 nm; gradient - 1%B for 5 min, 10-30%B 6-20 min, 30-100%B 21-25 min; flowrate 0.2 mL/min; eluent A – 0.1% formic acid in water, eluent B – 0.1% formic acid in acetonitrile)

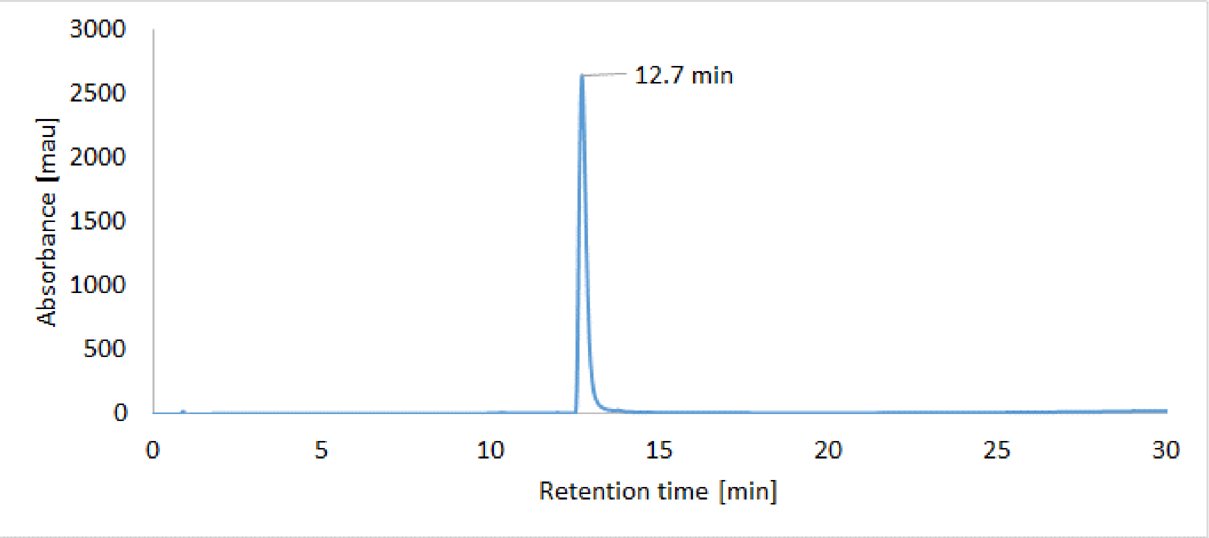

Figure S86. RP-HPLC chromatogram of purified TMT(AcOH)(AcCysOH)$_2$

HRMS (ESI-MS) m/z: $[M+H]^+$ Calcd for $C_{15}H_{20}N_5O_8S_3$ 494.0468; Found 494.0491, $[2M+H]^+$ Calcd for $C_{30}H_{39}N_{10}O_{16}S_6$ 987.0864; Found 987.0929

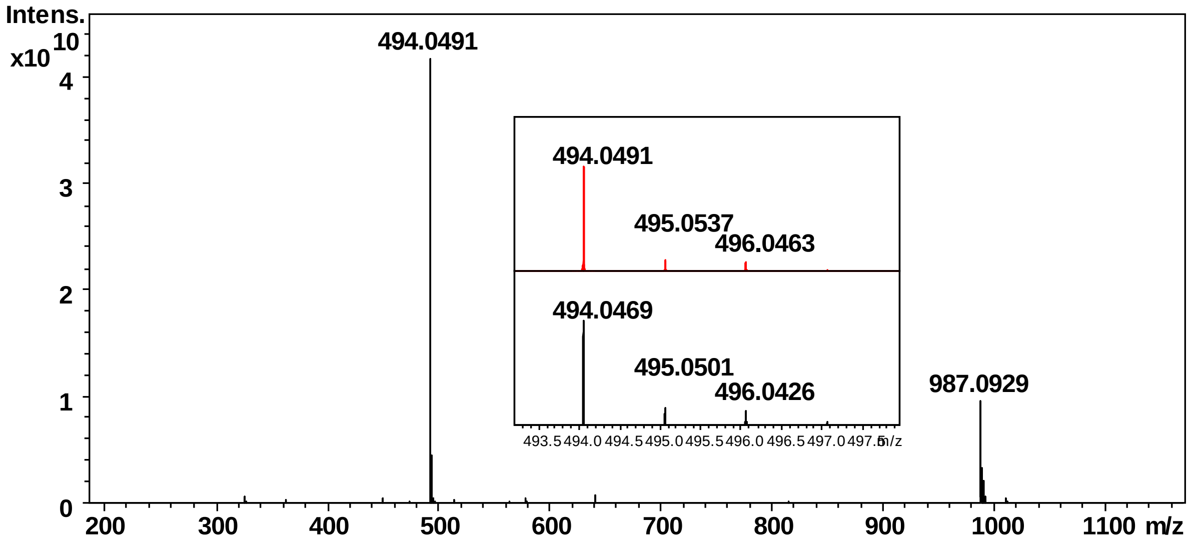

Figure S87. ESI-MS spectrum in positive ion mode of TMT(AcOH)(AcCysOH)$_2$ and comparison of measured (red) and simulated (black) isotopic pattern of [M+H]+ ion.

ESI-MS(+)/MS, m/z: 476.0294 ($[M\text{-}OH]^+$, Calcd 476.0368); 452.0292 ($[M\text{-}Ac+2H]^+$, Calcd 452.0363); 434.0183 ($[M\text{-}AcOH+2H]^+$, Calcd 434.0263); 406.0227 ($[M\text{-}AcOH\text{-}COOH]^+$, Calcd 406.0308); 364.9944 ($[M\text{-}Ac\Delta AlaOH+H]^+$, Calcd 365.0043); 346.9836 ($[M\text{-}Ac\Delta AlaOH\text{-}OH+H]^+$, Calcd 346.9942); 322.9902 ($[M\text{-}Ac\Delta AlaOH\text{-}Ac+2H]^+$, Calcd 322.9937); 305.9793 ($[M\text{-}Ac\Delta AlaOH\text{-}AcNH2+H]^+$, Calcd 305.9677); 276.9913 ($[M\text{-}Ac\Delta AlaOH\text{-}Ac\text{-}COOH+H]^+$, Calcd 276.9888); 235.9556 ($[C_5H_6N_3O_2S_3]^+$, Calcd 234.9544); 217.9501 ($[C_5H_4N_3OS_3]^+$, Calcd 217.9516)

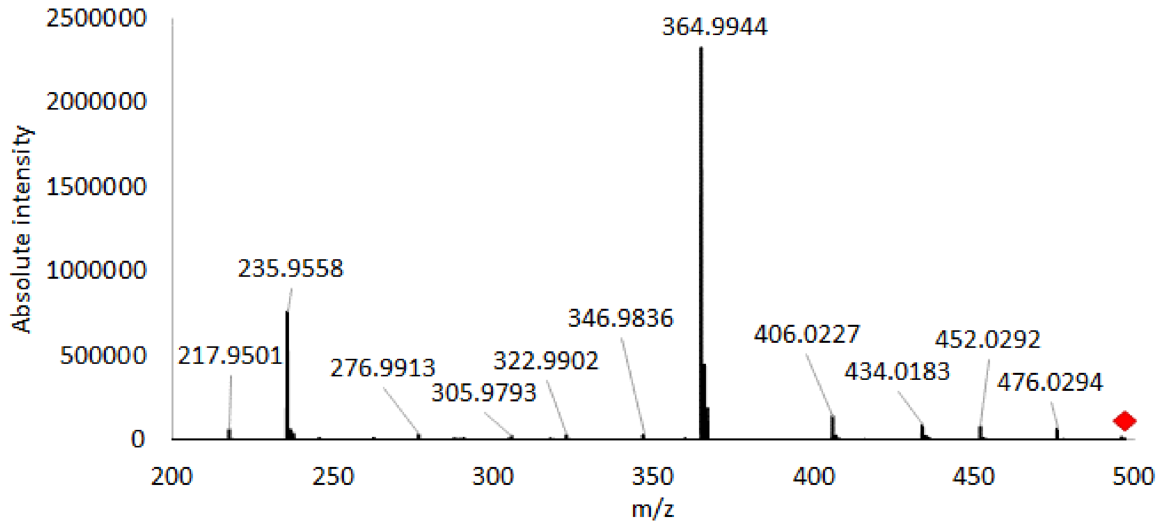

Figure S88. CID fragmentation MS[2] spectrum of TMT(AcOH)(AcCysOH)$_2$, [M+H]$^+$ parent ion.

1H NMR (DMSO-d6, 500 MHz, 25°C) δ (ppm) = 8.35 (d, 2H, HN, Cys), 4.53 (m, 2H, HA, Cys), 3.99 (s, 2H, HA, SAc), 3.69 (dd, 2H, HB1, Cys), 3.27 (dd, 2H, HB2, Cys), 1.83 (s, 6H, Ac)

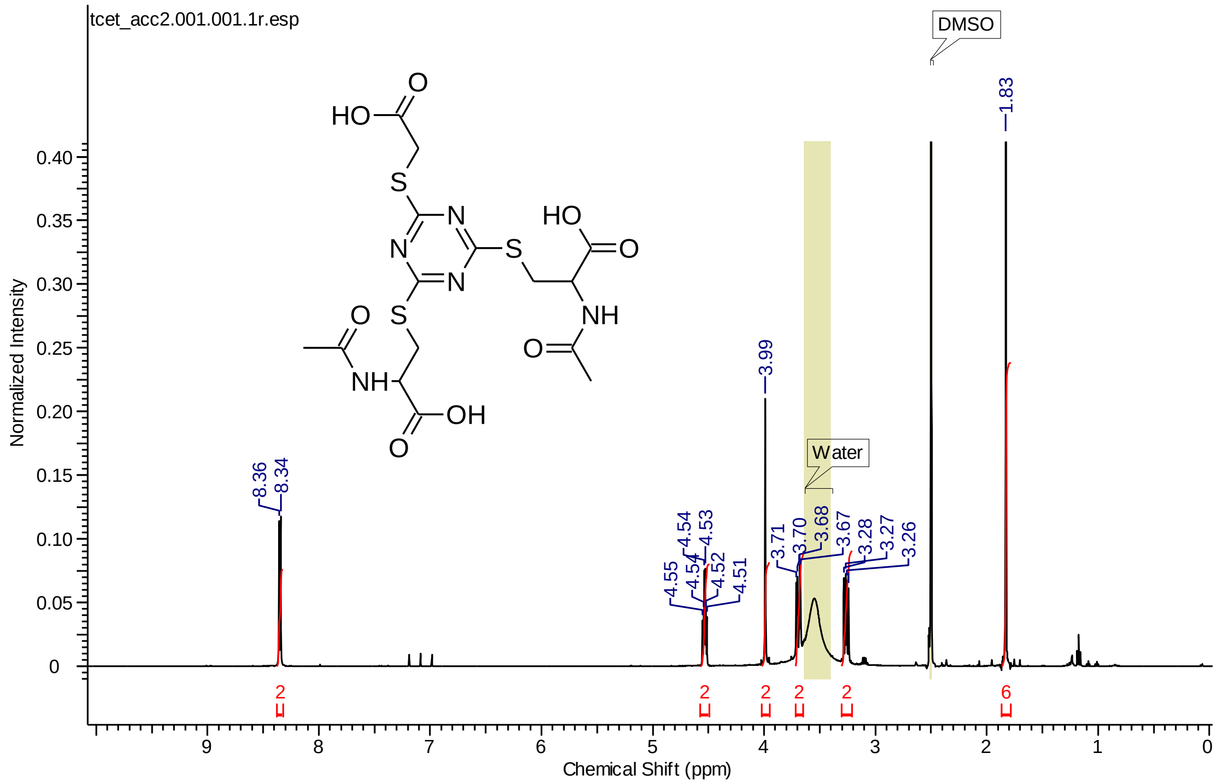

Figure S89. 1H NMR spectrum of TMT(AcOH)(AcCysOH)$_2$ in DMSO-d_6

13C{H} NMR (DMSO-d6, 125 MHz, 25°C) δ (ppm) = 178.5 (C2,C3), 178.3 (C1), 171.6 (C6, C11), 169.4 (C9, C14), 169.4 (C4), 51.1 (C7,C12), 32.4 (C5), 30.9 (C8,C13), 22.3 (C10,C15)

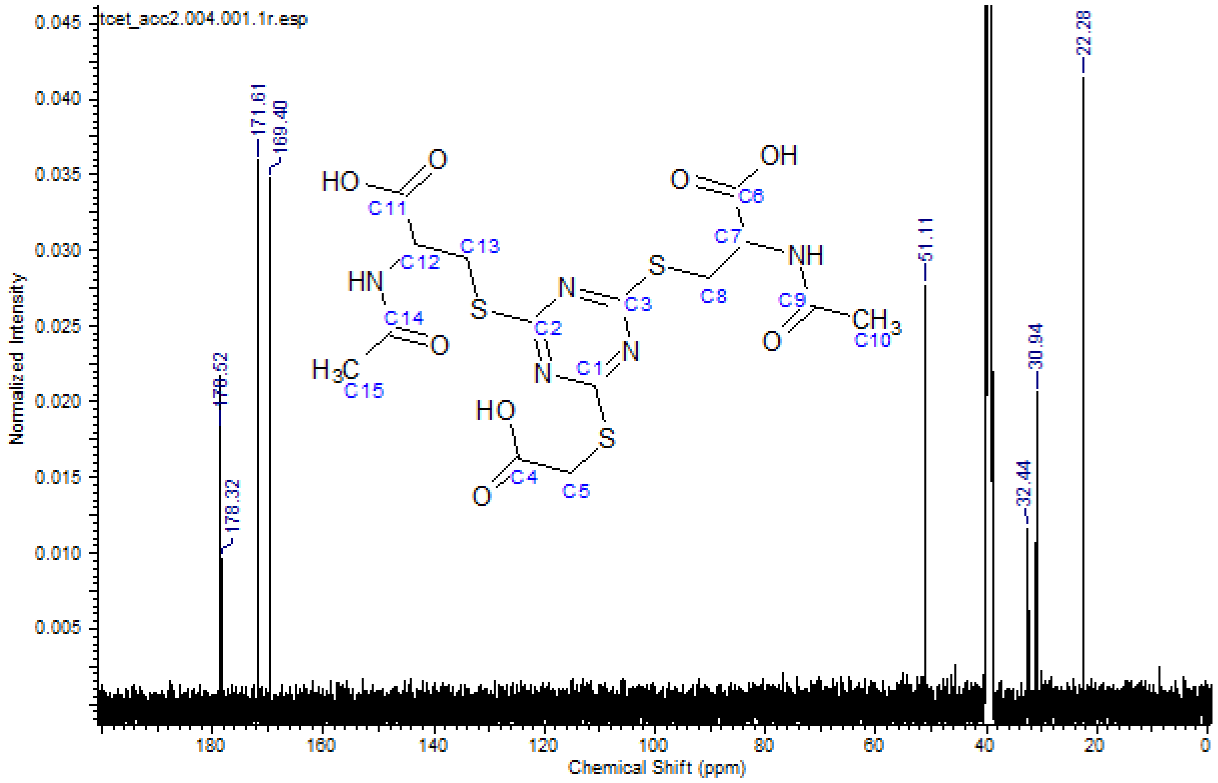

Figure S90. 13C{H} NMR spectrum of TMT(AcOH)(AcCysOH)$_2$ in DMSO-d$_6$

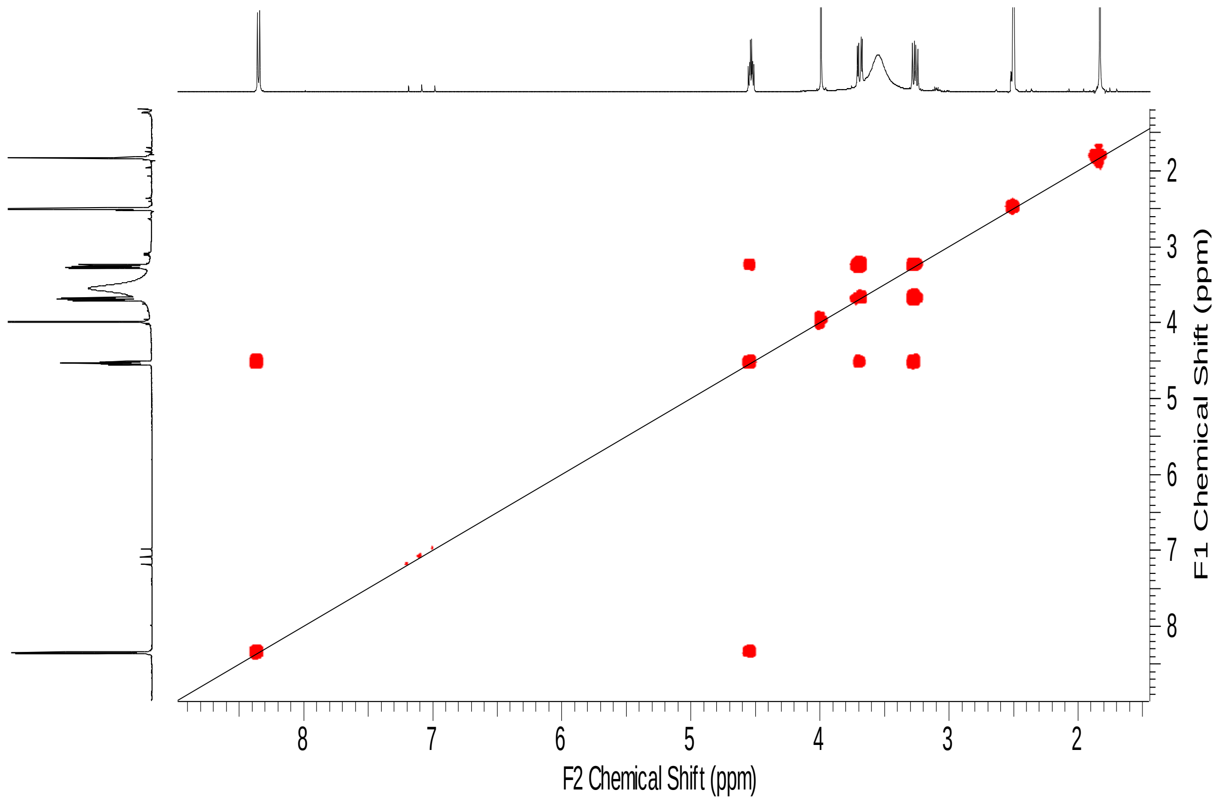

Figure S91. COSY spectrum of TMT(AcOH)(AcCysOH)$_2$

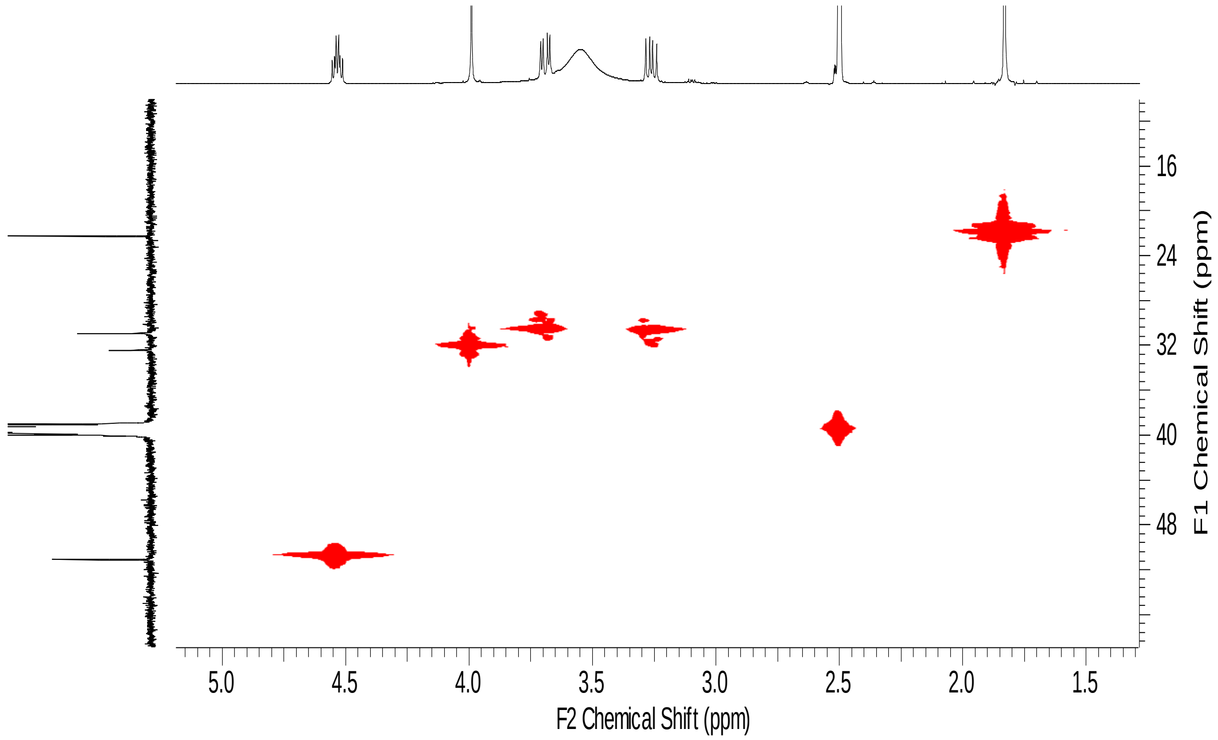

Figure S92. HSQC spectrum of TMT(AcOH)(AcCysOH)$_2$

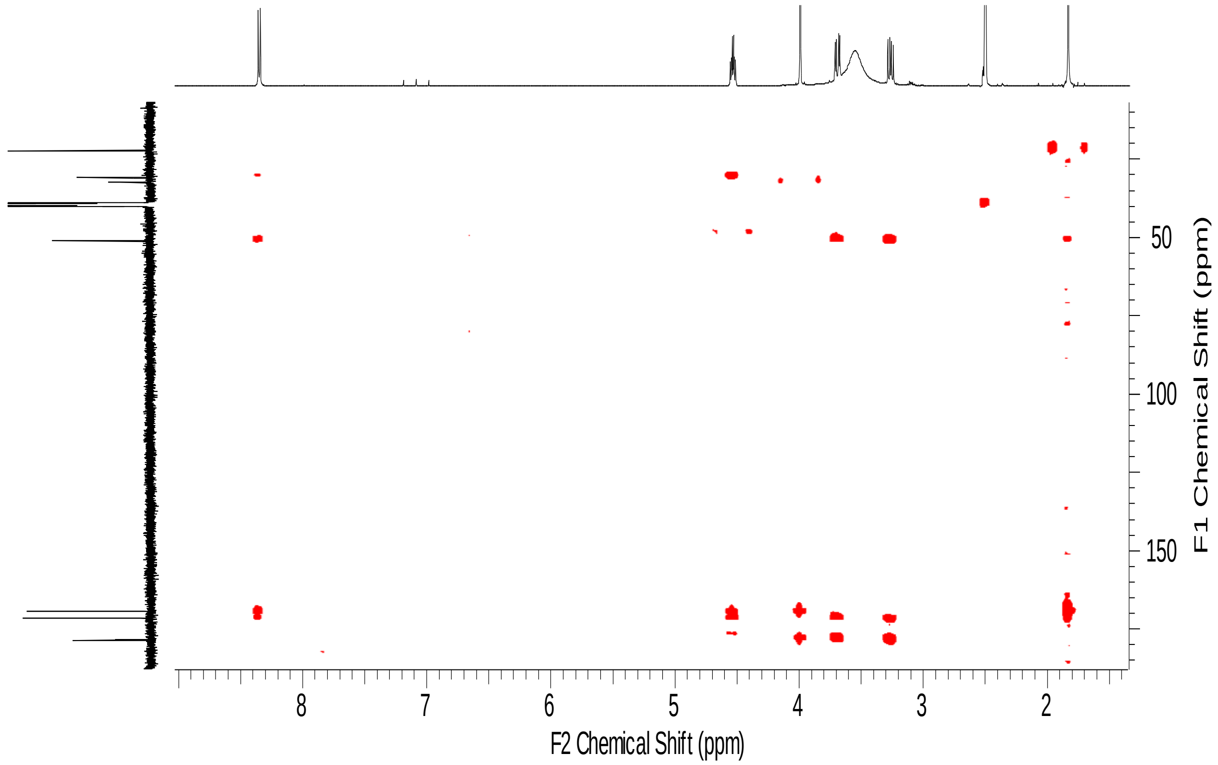

Figure S93. HMBC spectrum of TMT(AcOH)(AcCysOH)$_2$

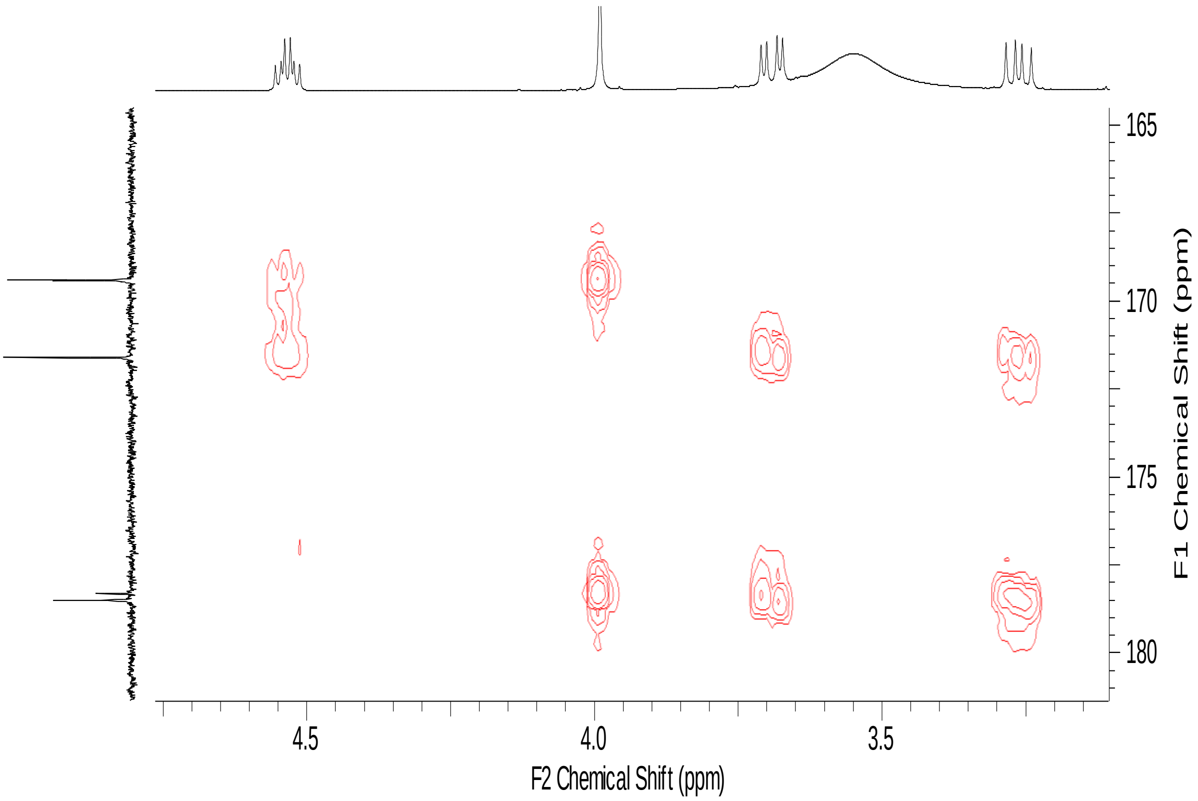

Figure S94. Correlation between aliphatic protons and carbonyl carbon atoms on HMBC spectrum of TMT(AcOH)(AcCysOH)$_2$

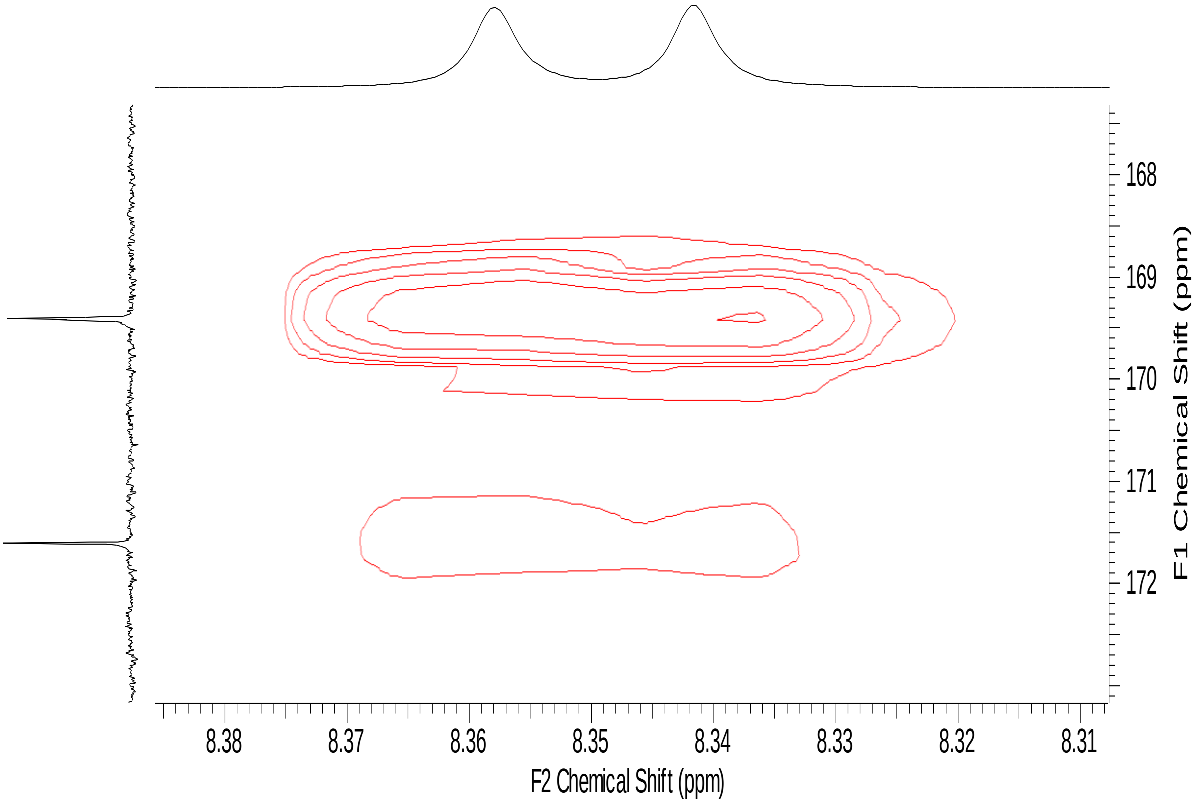

Figure S95. Correlation between amid protons and carbonyl carbon atoms on HMBC spectrum of TMT(AcOH)(AcCysOH)$_2$

TMT(AcCysOH)$_3$ – 3,3',3''-(1,3,5-triazine-2,4,6-triyl}trisulfanyl)tris[2-(acetylamino)propanoic acid]

Not isolated. The retention time and mass spectra have been described based on RP-HPLC/LC-MS analysis of the post-reaction mixture.

HPLC: 12.4 min (column – Aeris 3.6 µm PEPTIDE XB-C18 50 x 2.1 mm; detection -D, 240 nm; gradient - 1%B for 5 min, 10-30%B 6-20 min, 30-100%B 21-25 min; flowrate 0.2 mL/min; eluent A – 0.1% formic acid in water, eluent B – 0.1% formic acid in acetonitrile)

HRMS (ESI-MS) m/z: $[M+H]^+$ Calcd for $C_{18}H_{25}N_6O_9S_3$ 565.0839; Found 565.0734, $[M+Na]^+$ Calcd for $C_{18}H_{24}N_6O_9S_3Na$ 587.0660; Found 587.0671, $[M+K]^+$ Calcd for $C_{18}H_{24}N_6O_9S_3K$ 603.0398; Found 603.0242

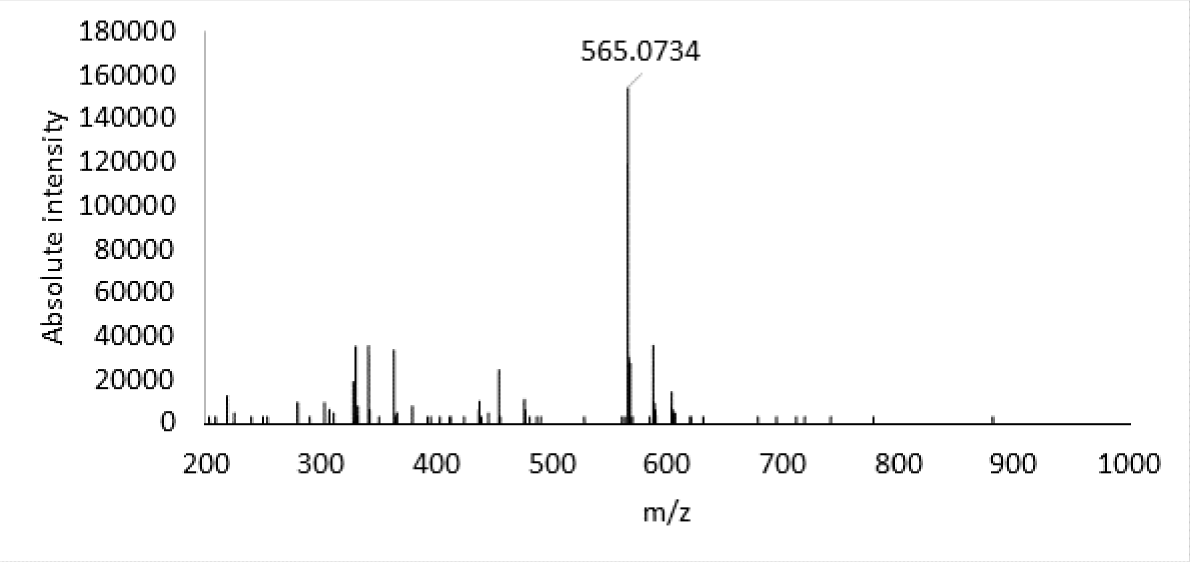

Figure S96. ESI-MS spectrum in positive ion mode of TMT(AcCysOH)$_3$

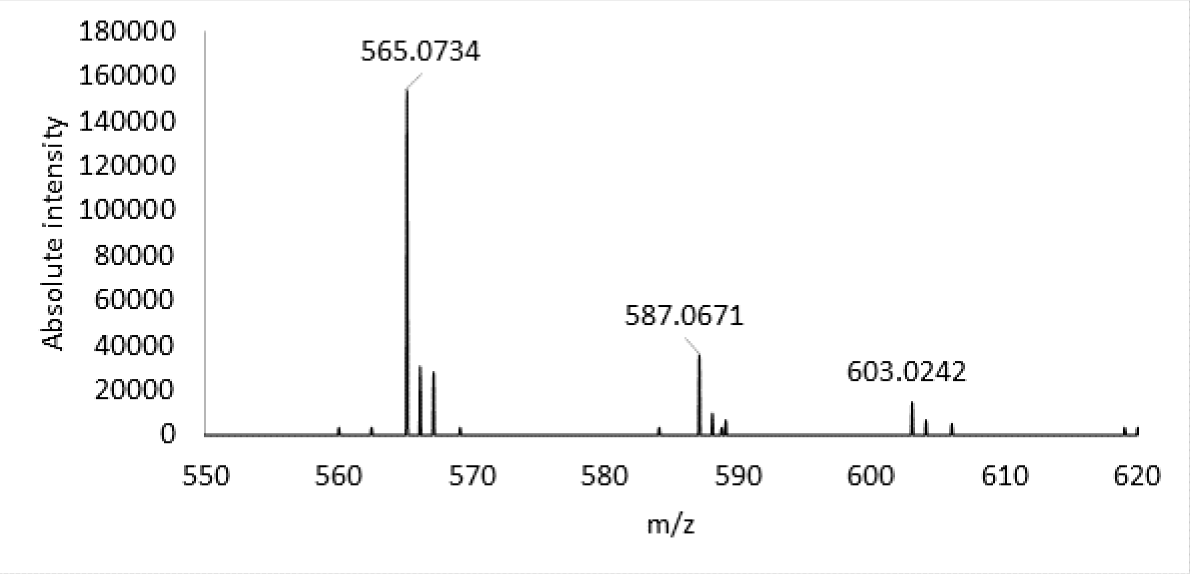

Figure S97. Fragment of ESI-MS spectrum of TMT(AcCysOH)$_3$ corresponding to $[M+H]^+$, $[M+Na]^+$, and $[M+K]^+$ ions.

ESI-MS(+)/MS (automatic CID fragmentation during LC-MS analysis), m/z: 547.0732 ($[M-OH]^+$, Calcd 547.0739); 523.0708 ($[M-Ac+2H]^+$, Calcd 523.0734); 506.0309 ($[M-AcNH2+H]^+$, Calcd 506.0468); 477.0683 ($[M-Ac-COOH+H]^+$, Calcd 477.0679); 436.0426 ($[M-Ac\Delta AlaOH+H]^+$, Calcd 436.0414); 394.0218 ($[M-Ac\Delta AlaOH-Ac+2H]^+$, Calcd 394.0308); 360.0372 ($[M-2Ac+2H-AcNH_2-OH]^+$, Calcd 360.0259); 306.9922 ($[M-2Ac\Delta AlaOH+H]^+$, Calcd 306.9988)

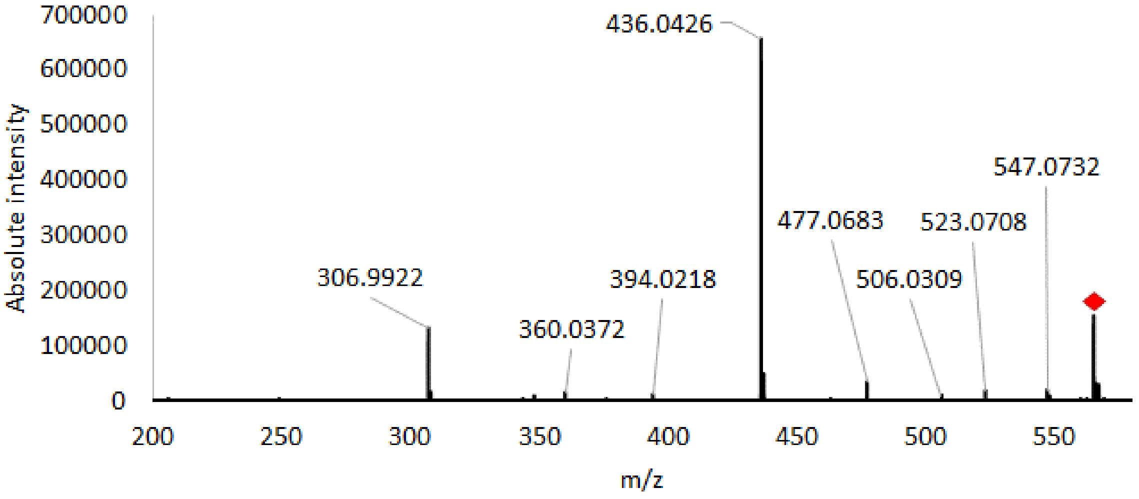

Figure S98. CID fragmentation MS² spectrum of TMT(AcCysOH)₃, [M+H]⁺ parent ion.

4.1.2. Reaction with glutathione

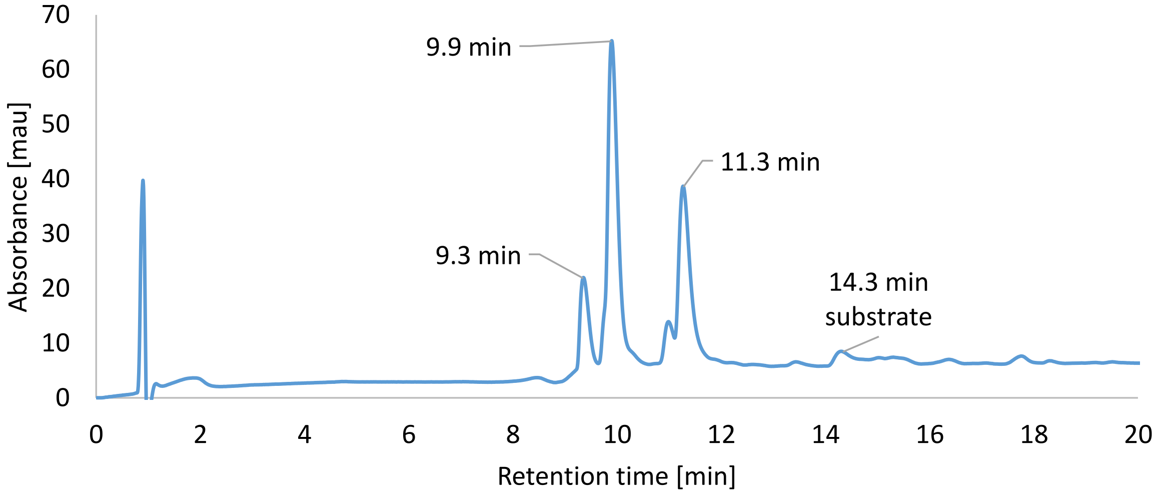

Figure S99. RP-HPLC profile (detection at 240 nm) of the final mixture after reaction of glutathione with $TMT(AcOH)_3$: 14.3 min – $TMT(AcOH)_3$; 11.3 min – $TMT(Ac)_2$(glutathione); 9.9 min – TMT(Ac)$(glutathione)_2$; 9.3 min – $TMT(glutathione)_3$

TMT(AcOH)$_2$(glutathione) – *S*-({4,6-bis[(carboxymethyl)sulfanyl]-1,3,5-triazin-2-yl)glutathione

HPLC: 12.0 min (column – Aeris 3.6 µm PEPTIDE XB-C18 50 x 2.1 mm; detection -D, 240 nm; gradient - 1%B for 5 min, 10-30%B 6-20 min, 30-100%B 21-25 min; flowrate 0.2 mL/min; eluent A – 0.1% formic acid in water, eluent B – 0.1% formic acid in acetonitrile)

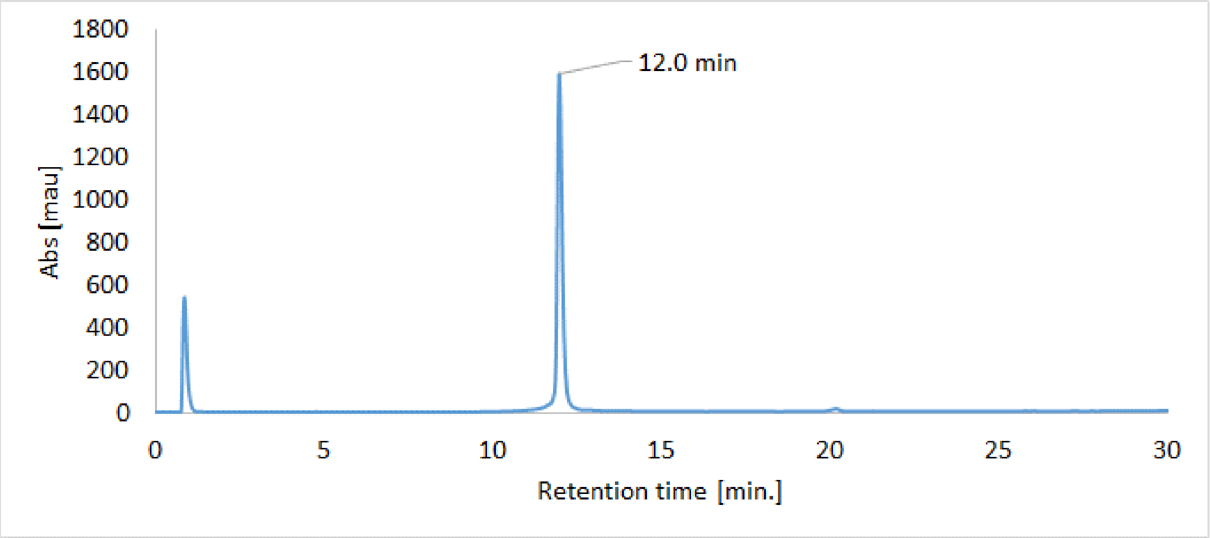

Figure S100.RP-HPLC chromatogram of purified TMT(AcOH$_2$(glutathione)

HRMS (ESI-MS) m/z: [M+H]$^+$ Calcd for $C_{17}H_{23}N_6O_{10}S_3$ 567.0632; Found 567.0641, [2M+H]$^+$ Calcd for $C_{34}H_{45}N_{12}O_{20}S_6$ 1133.1192; Found 1133.1336

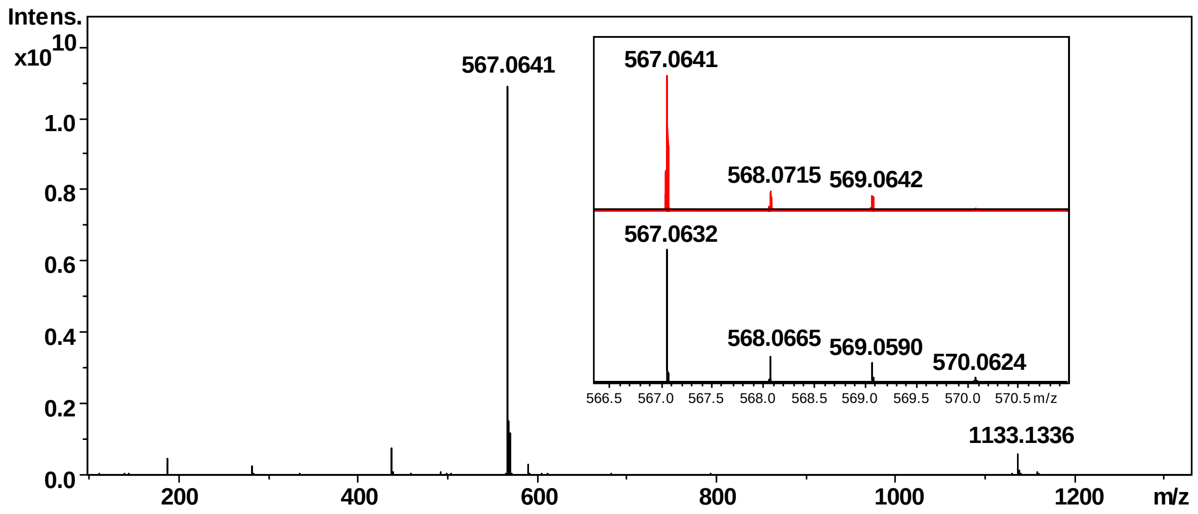

Figure S101. ESI-MS spectrum in positive ion mode of TMT(AcOH)$_2$(glutathione)

ESI-MS(+)/MS, mz: 549.0578 ($[C_{17}H_{21}N_6O_9S_3]^+$ = b_3, Calcd 549.0532); 492.0355 ($[C_{15}H_{18}N_5O_8S_3]^+$ = b_2, Calcd 492.0317]); 438.0240 ($[C_{12}H_{16}N_5O_7S_3]^+$ = y_2, Calcd 438.0206); 420.9977 ($[C_{12}H_{14}N_5O_6S_3]^+$ = $b_2(y_2)$, Calcd 420.0106); 378.0030 ($[C_{10}H_{12}N_5O_5S_3]^+$ = $y_1(c_2)$, Calcd 377.9995); 362.9912 ($[C_{10}H_{11}N_4O_5S_3]^+$ = $b_1(y_2)$, 362.9891); 346.0303 ($[C_{10}H_8N_3O_5S_3]^+$ = $b_1(z_2)$, Calcd 345.9621); 334.9961 ($[C_9H_{11}N_4O_4S_3]^+$ = $a_1(y_2)$, Calcd 334.9937);

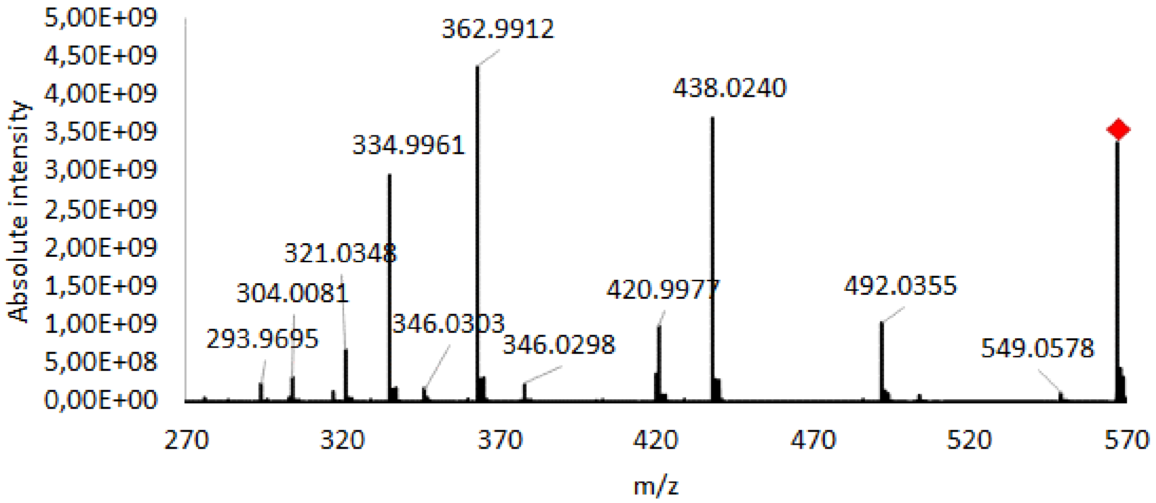

Figure S102. CID fragmentation MS² spectrum of TMT(AcOH)₂(glutathione), [M+H]⁺ parent ion

1H NMR (DMSO-d6, 500 MHz, 25°C) δ (ppm) = 8.43 (t, 1H, NH, Gly), 7.13 (bm, 3H, NH_3^+, Glu), 8.38 (d, 1H, NH, Cys), 4.65 (m, 1H, HA, Cys), 3.96 (dd, 4H, HA##, SAc), 3.77 (m, 1H, HA, Glu), 3.75 (dd, 2H, HA#, Gly), 3.53 (dd, 1H, HB1, Cys), 3.29 (dd, 1H, HB2, Cys), 2.33 (dm, 2H, HG#, Glu), 1.98 (dm, 2H, HB#, Glu)

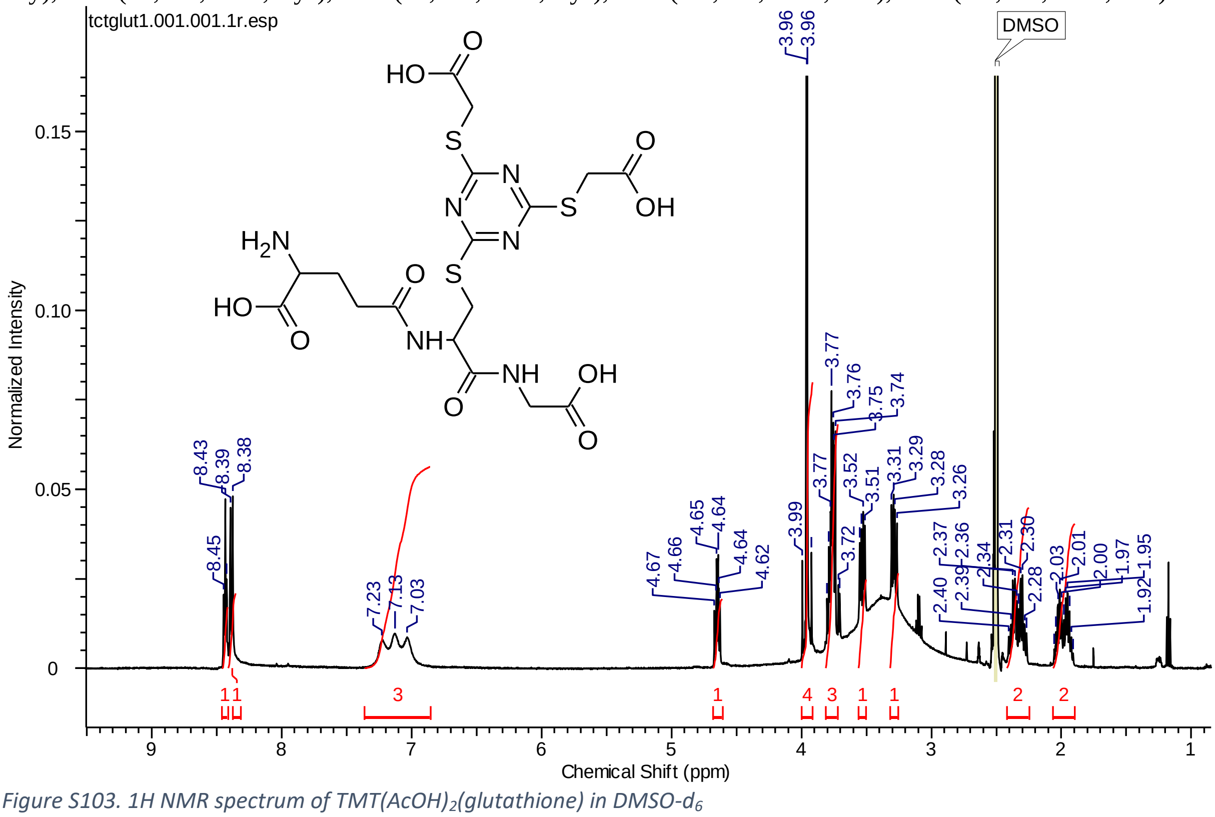

Figure S103. 1H NMR spectrum of TMT(AcOH)₂(glutathione) in DMSO-d₆

13C{H} NMR (DMSO-d6, 125 MHz, 25°C) δ (ppm) = 178.6 (C11), 178.3 (C12/C13), 171.2 (C5), 170.8 (C1/C9), 169.9 (C7), 169.4 (C17/C17), 52.0 (C2), 51.2 (C6), 40.8 (C8), 32.7 (C14/C16), 31.5 (C10), 30.8 (C4), 25.9 (C3)

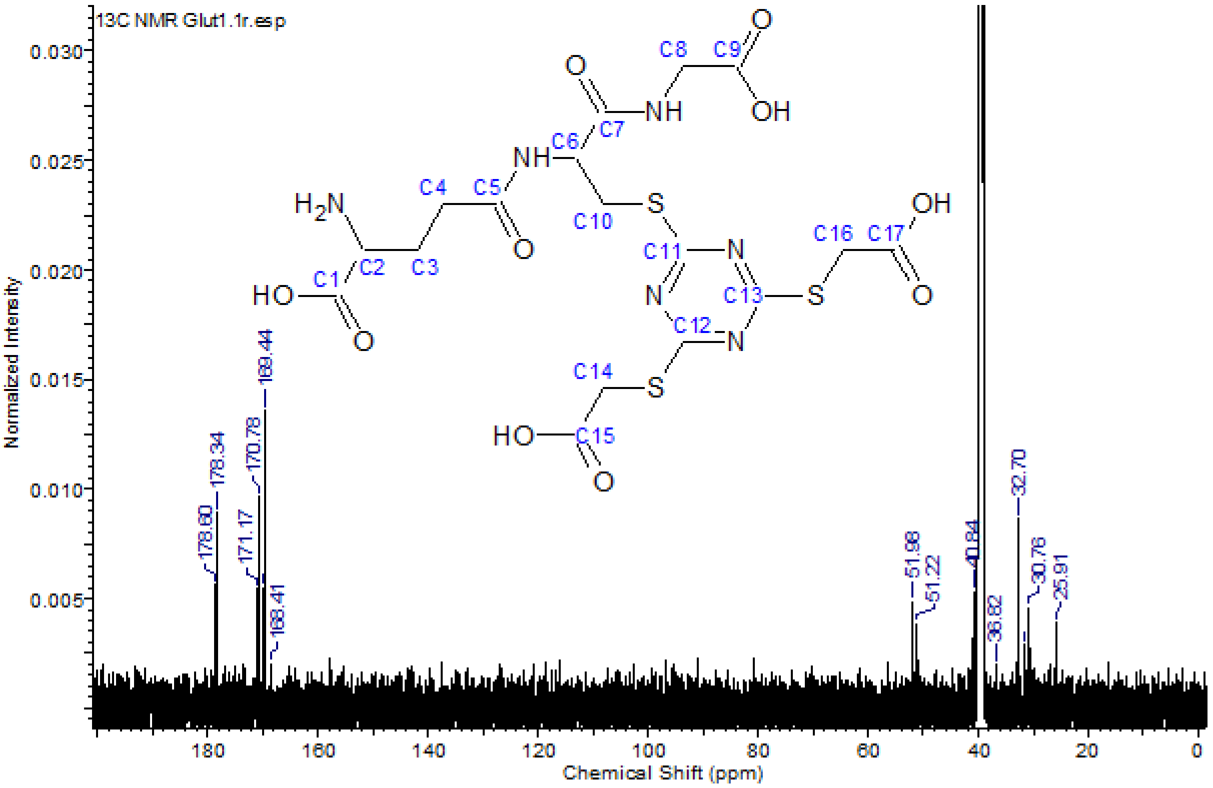

Figure S104. 13C{H} NMR spectrum of TMT(AcOH)$_2$(glutathione) in DMSO-d$_6$

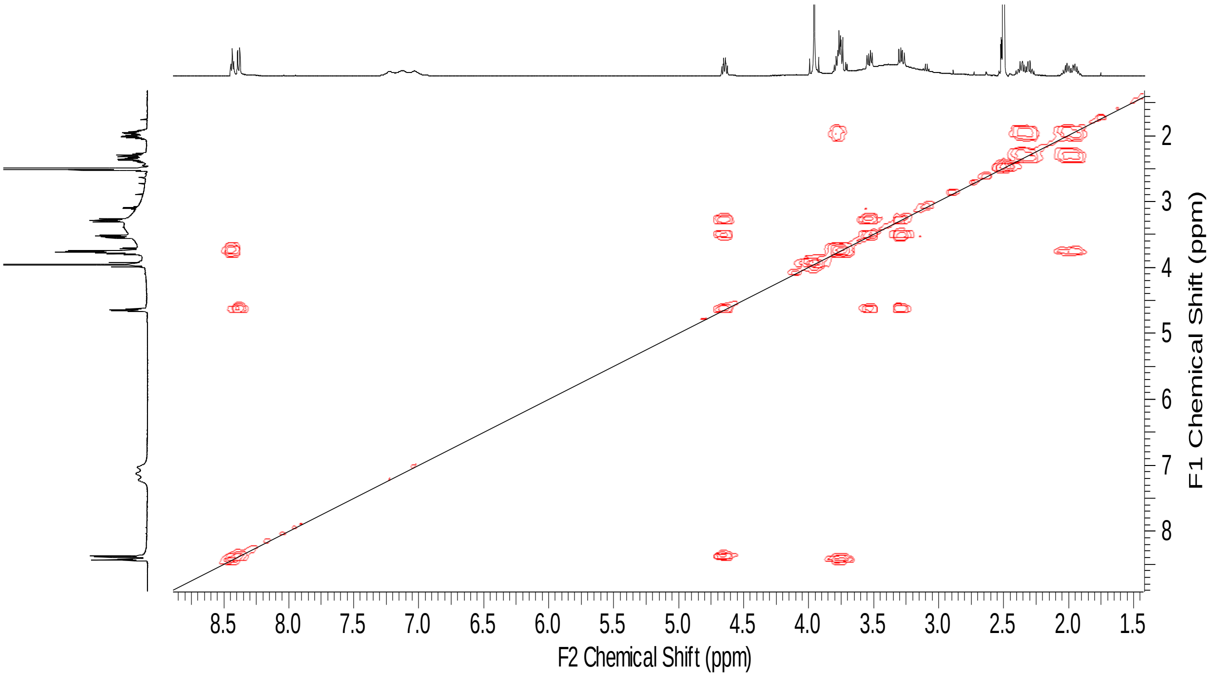

Figure S105. COSY spectrum of TMT(AcOH)$_2$(glutathione) in DMSO-d$_6$

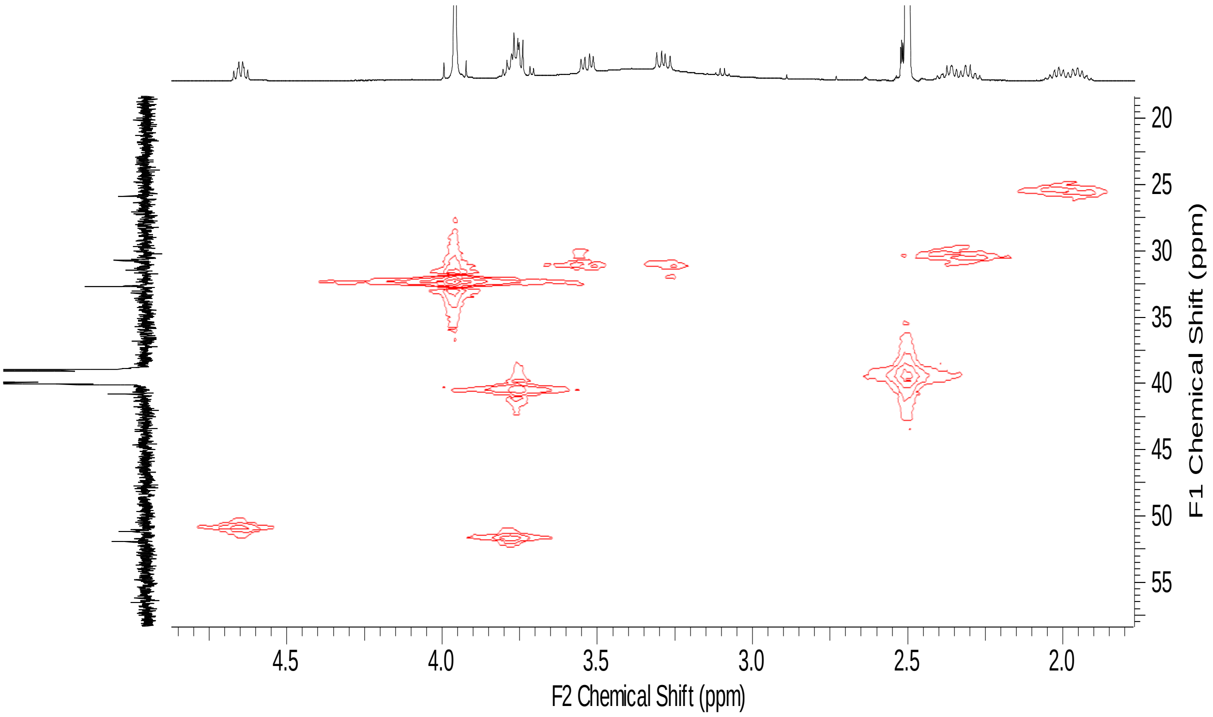

Figure S106. HSQC spectrum of TMT(AcOH)$_2$(glutathione) in DMSO-d_6

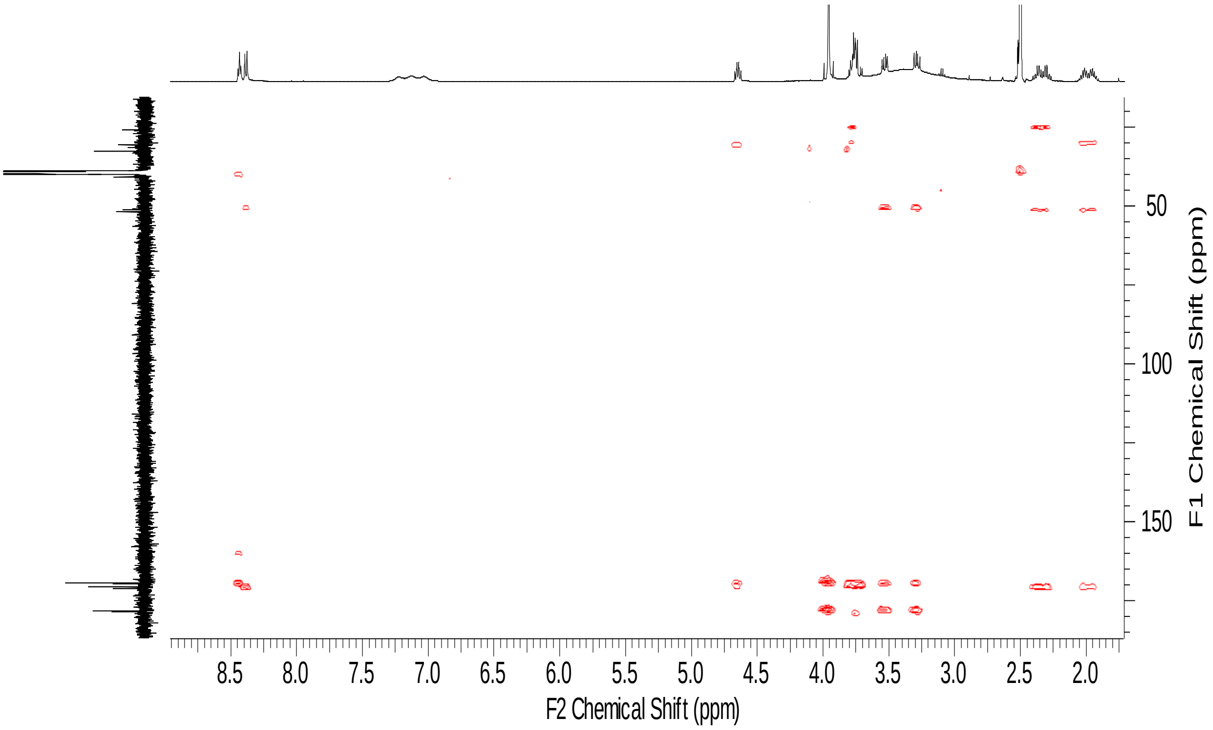

Figure S107. HMBC spectrum of TMT(AcOH)$_2$(glutathione) in DMSO-d_6

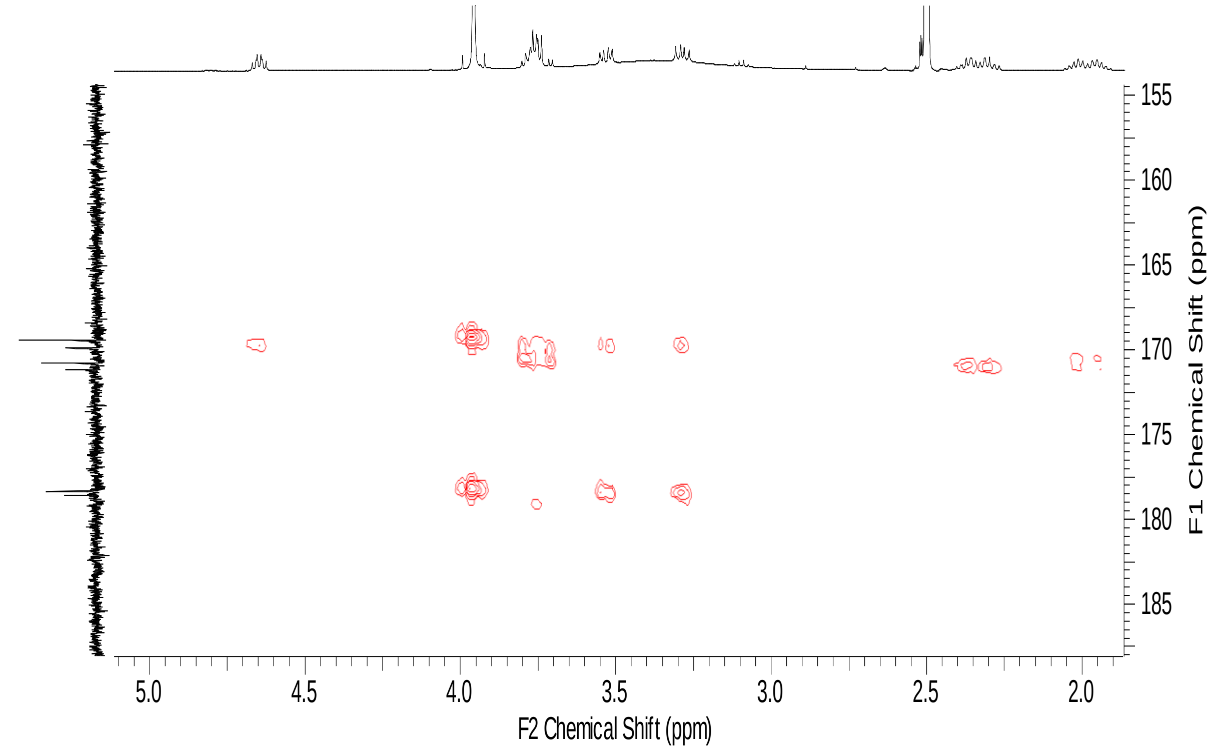

Figure S108. Correlations of aliphatic protons and aromatic or carbonyl carbon atoms on HMBC spectrum of TMT(AcOH)$_2$(glutathione)

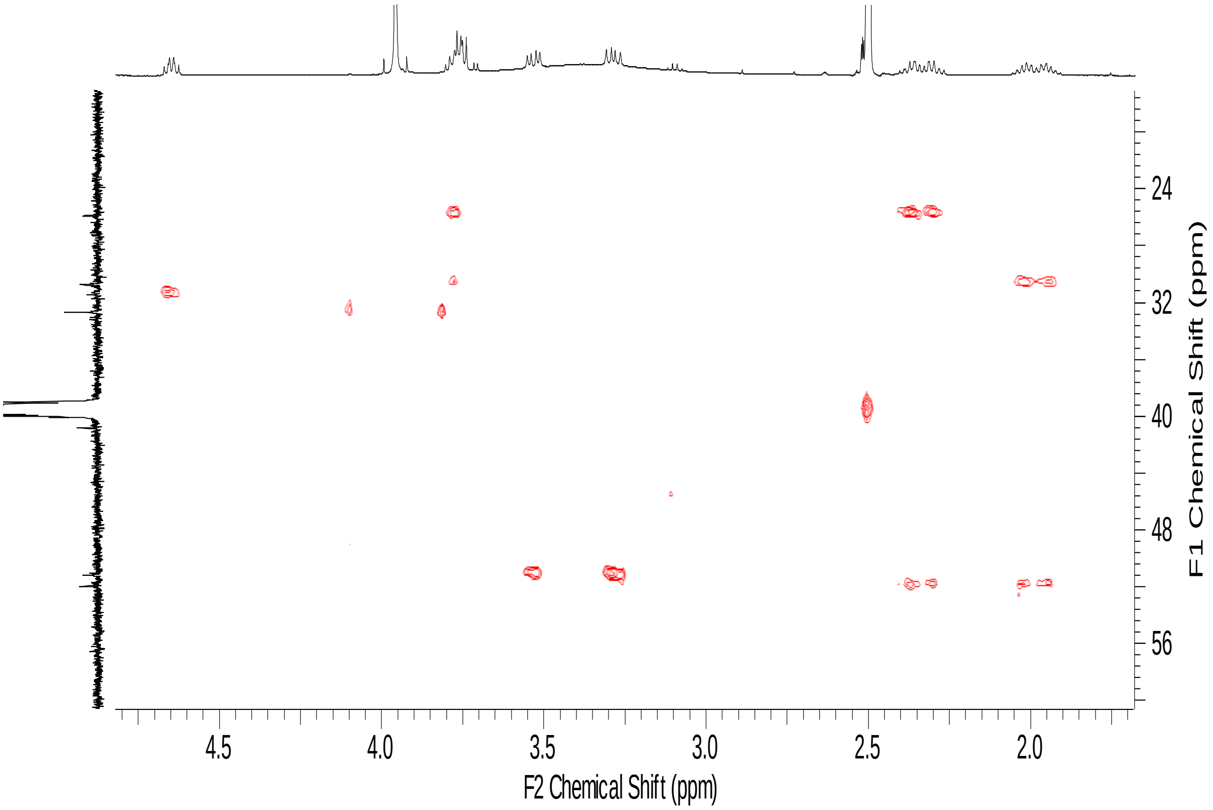

Figure S109. Correlations between aliphatic protons andaliphatic carbon atoms on HMBC spectrum of TMT(AcOH)$_2$(glutathione)

TMT(AcOH)(glutathione)$_2$ –
S,S'-({6-[(carboxymethyl)sulfanyl]-1,3,5-triazine-2,4-diyl}disulfanyl)bis(glutathione)

HPLC: 9.8 min (column – Aeris 3.6 µm PEPTIDE XB-C18 50 x 2.1 mm; detection -D, 240 nm; gradient - 1%B for 5 min, 10-30%B 6-20 min, 30-100%B 21-25 min; flowrate 0.2 mL/min; eluent A – 0.1% formic acid in water, eluent B – 0.1% formic acid in acetonitrile)

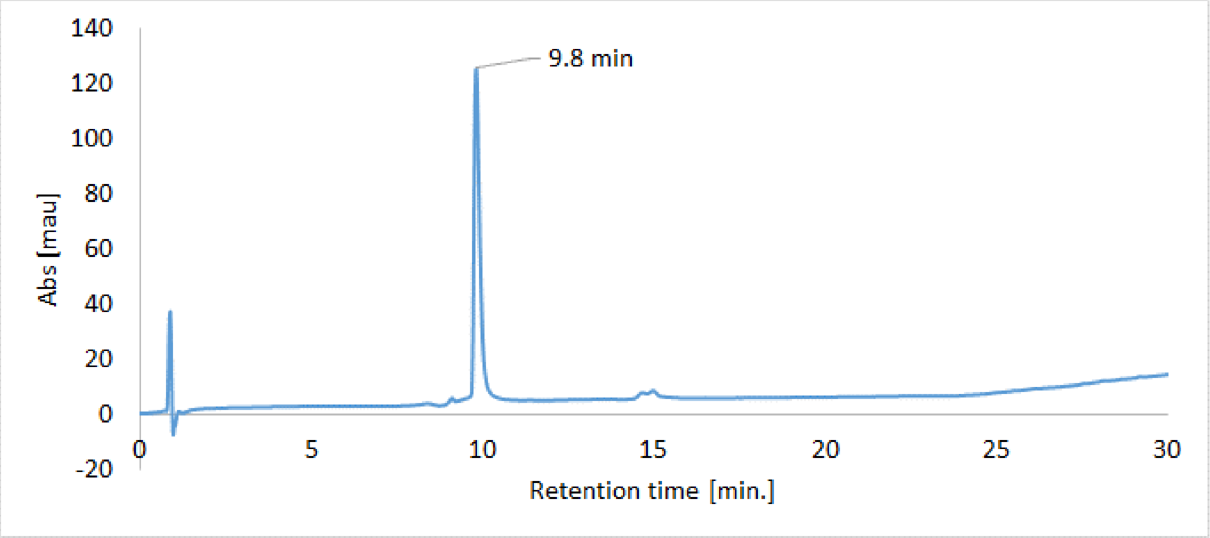

Figure S110. RP-HPLC chromatogram of purified TMT(AcOH)(glutathione)$_2$

HRMS (ESI-MS) m/z: $[M+H]^+$ Calcd for $C_{25}H_{35}N_9O_{14}S3$ 782.1538; Found 782.1601

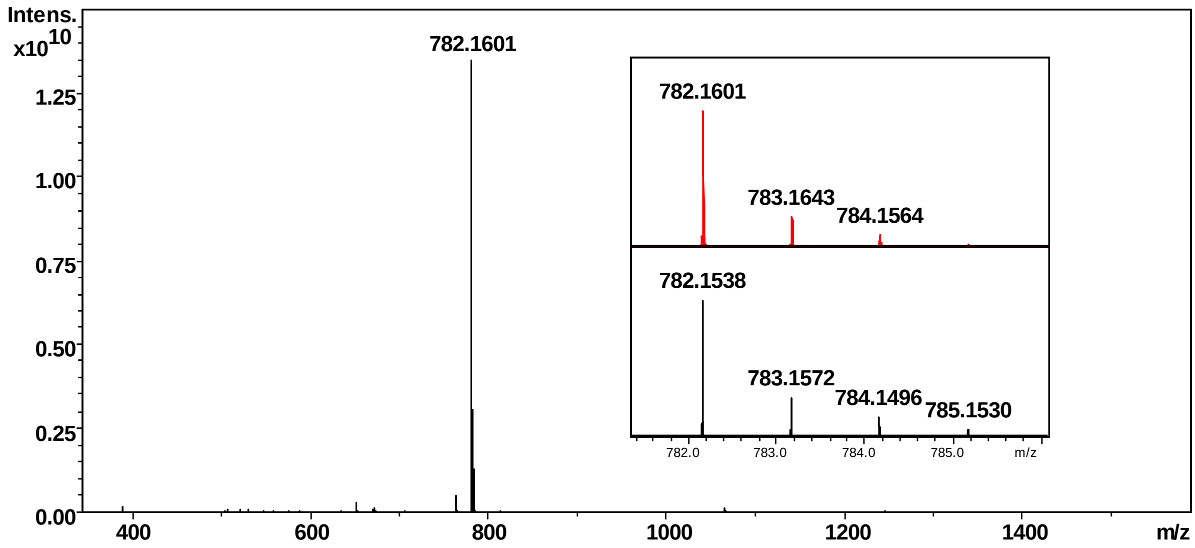

Figure S111. ESI-MS in positive ion mode of TMT(AcOH)(glutathione)$_2$ and comparison of measured (red) and simulated (black) isotopic pattern of $[M+H]^+$ ion.

ESI-MS(+)/MS, m/z: 764.1505 ([M-H_2O+H]$^+$, Calcd 764.1438); 746.1396 ([M-2H_2O+H]$^+$, Calcd 746.1333); 653.1173 (M/y_2, Calcd 653.1112); 635.1061 ([M/y_2-H_2O]$^+$,Calcd 635.1012); 617.0964 ([M/y_2-2H_2O]$^+$, Calcd 617.0964); 578.0847 (b_2/y_2 or M/b_2(y_2), Calcd 578.0792); 561.1232 ([b_2/y_2-NH_3]$^+$, Calcd 544.0267); 544.0966 ([b_2/y_2-H_2S]$^+$, Calcd 544.0915); 524.0729 (y_2/y_2, Calcd 524.0686); 507.0467 (y_2/y_2-NH_3, Calcd 507.0426); 509.0624 ([M-ΔGSH+H]$^+$, Calcd 509.0577); 491.0514 ([M-ΔGSH-H_2O+H]$^+$, Calcd 491.0514); 449.0407 (b_1(y_2)/y_2, Calcd 449.0372); 432.0791 ([b_1(y_2)/y_2-NH_3]$^+$, Calcd 432.0101Da); 390.0571 (unknown); 380.0184 (y_2/M-ΔGSH, Calcd 380.0151); 346.0301 (y_2-H_2S/M-ΔGSH, Calcd 346.0274); 304.9855 (b1(y2)/M-ΔGSH, Calcd 304.9837)

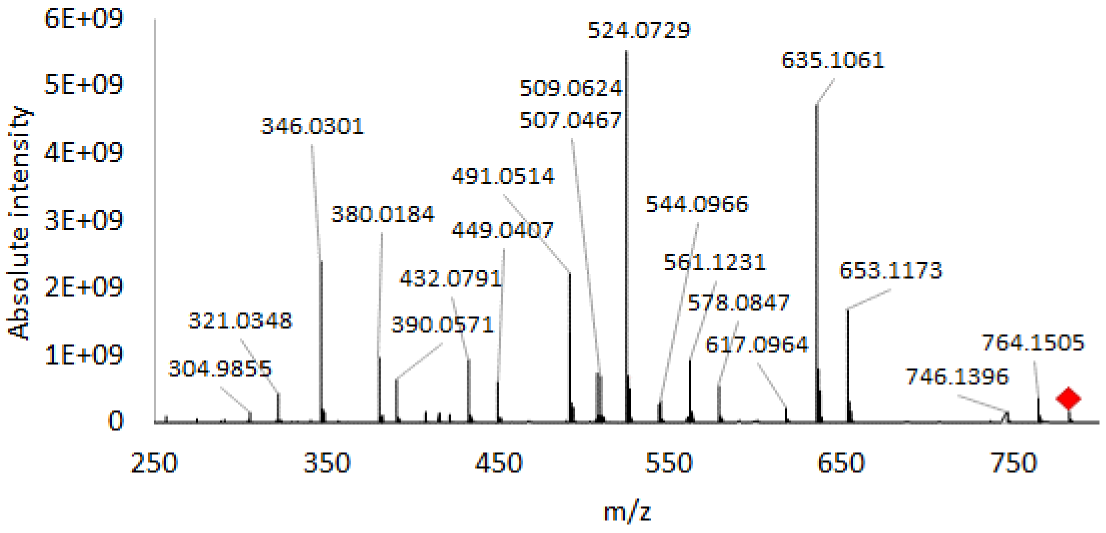

Figure S112. CID fragmentation MS^2 spectrum of TMT(AcOH)(glutathione)$_2$, [M+H]$^+$ paretn ion.

Legend for the assignment:

M/b_2 – one of the glutathione chains has been fragmented giving b_2 ion

b_3/b_2 – both glutathionyl chains have been fragmented b_2 and b_3 structures respectively

ΔGSH – neutral loss of H-γ-Glu-ΔAla-Gly-OH

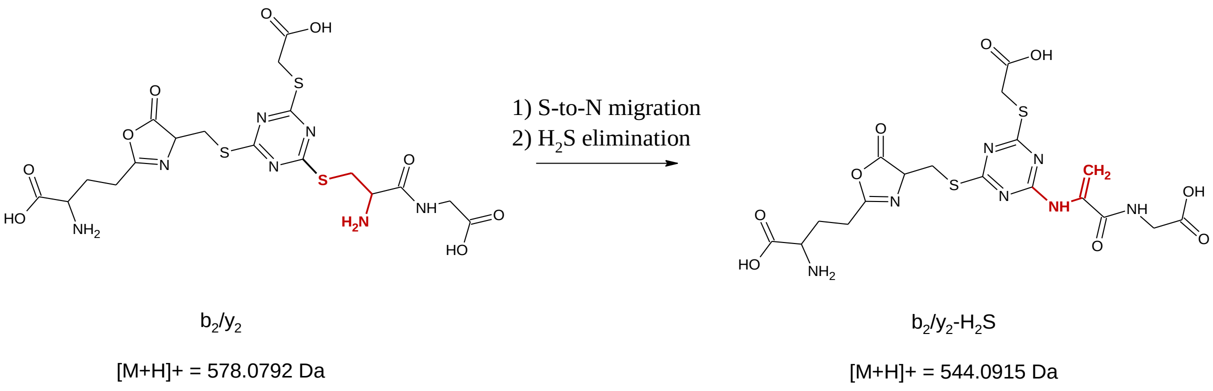

Scheme S5. The pathway proposition for S-to-N rearrangement of b_2/y_2 ion and H_2S elimination

1H NMR (DMSO-d6, 500 MHz, 25°C) δ (ppm) = 8.42 (d, 2H, NH, Cys)**,** 8.37 (t, 2H, NH, Gly), 4.65 (m, 2H, HA, Cys), 3.98 (dd, 2H, HA#, SAc), 3.87 (t, 2H, HA, Glu), 3.76 (ddd, 4H, HA#, Gly), 3.58 (dd, 2H, HB1, Cys), 3.26 (dd, 2H, HB2, Cys), 2.34 (m, 4H, HG#, Glu), 2.00 (m, 4H, HB#, Glu),

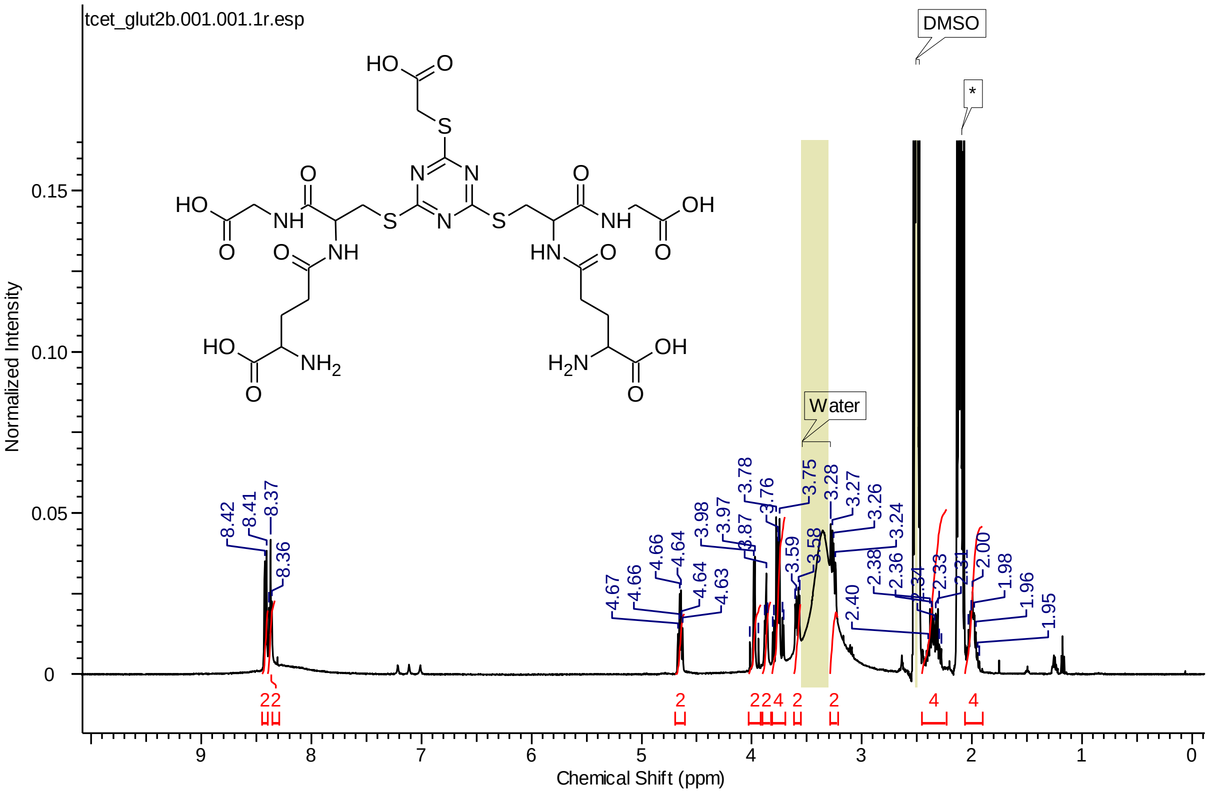

*Figure S113. 1H NMR spectrum of TMT(AcOH)(glutathione)$_2$ inDMSO-d$_6$. *solvent impurity.*

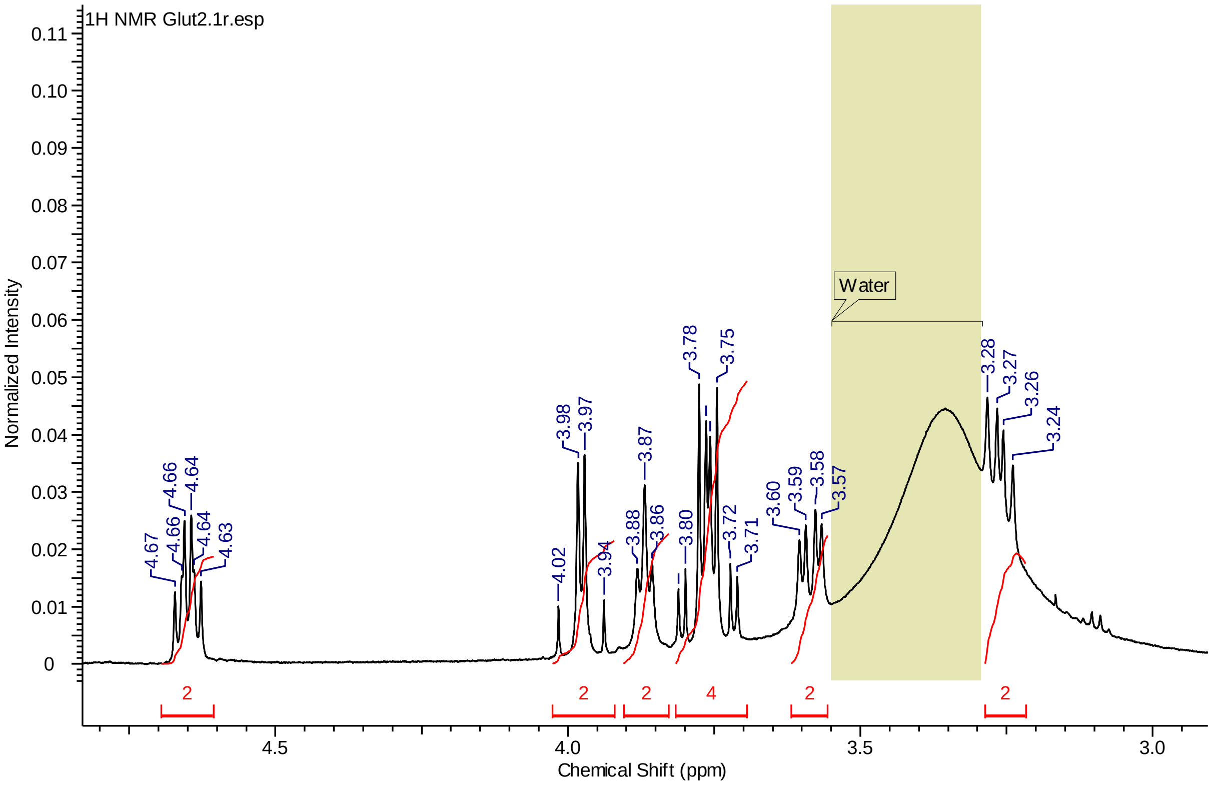

Figure S114. Shift range for aliphatic protons on 1H NMR spectrum of TMT(AcOH)(glutathione)$_2$ in DMSO-d$_6$

13C{H} NMR (DMSO-d6, 125 MHz, 25°C) δ (ppm) = 178.6 (C11), 178.3 (C12/C13), 171.2 (C5), 170.8 (C1/C9), 169.9 (C7), 169.4 (C17/C17), 52.0 (C2), 51.2 (C6), 40.8 (C8), 32.7 (C14/C16), 31.5 (C10), 30.76 (C4), 25.9 (C3)

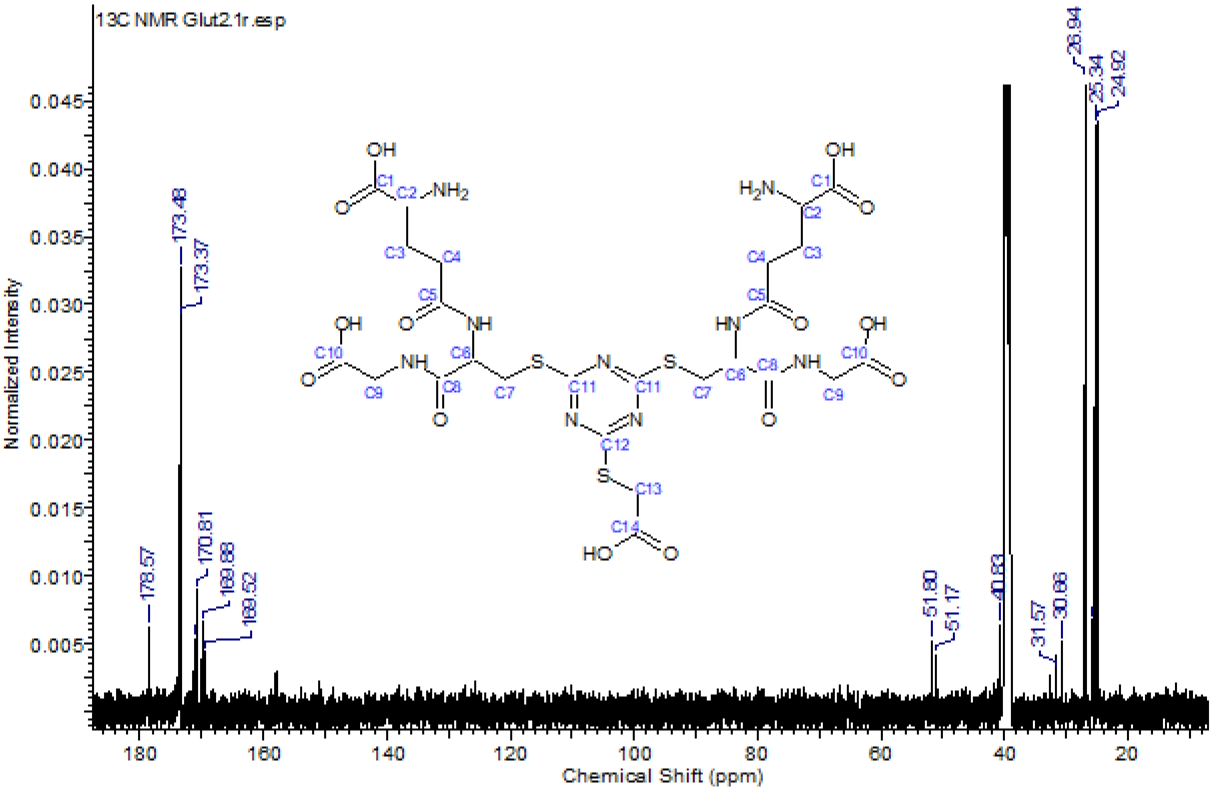

Figure S115. 13C{H} NMR spectrum of TMT(AcOH)(glutathione)$_2$ in DMSO-d$_6$

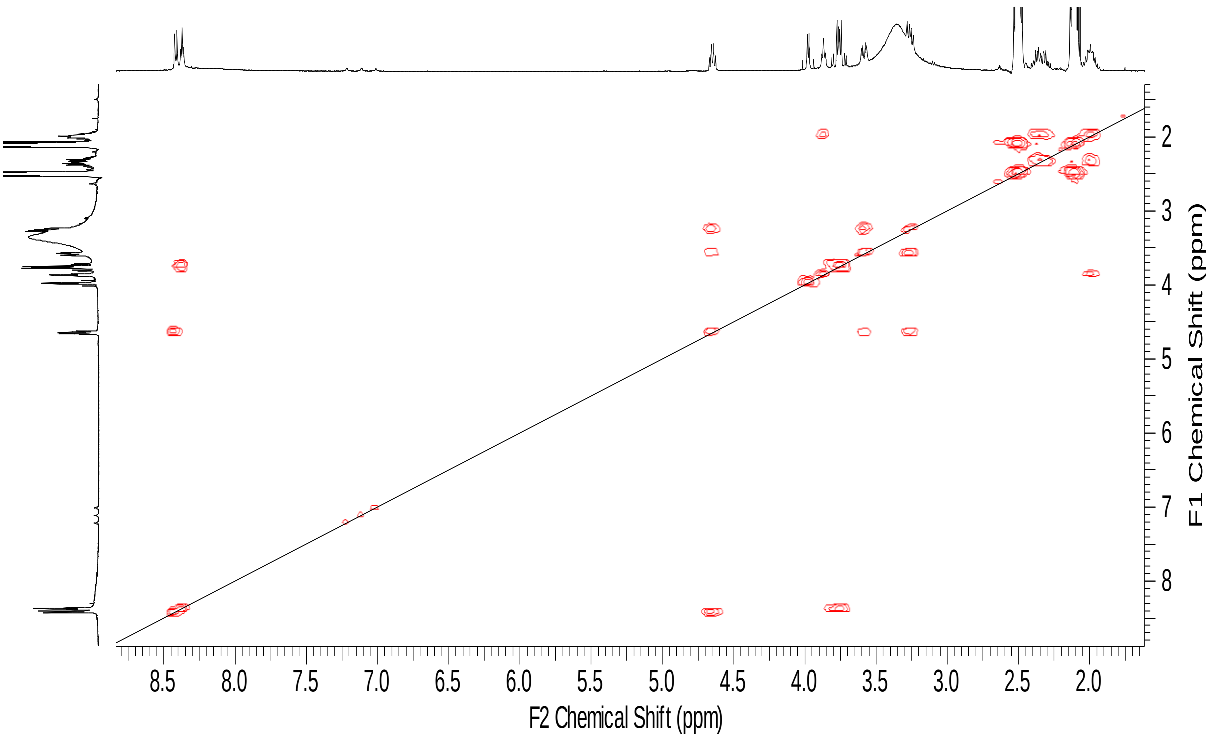

Figure S116. COSY spectrum of TMT(AcOH)(glutathione)$_2$

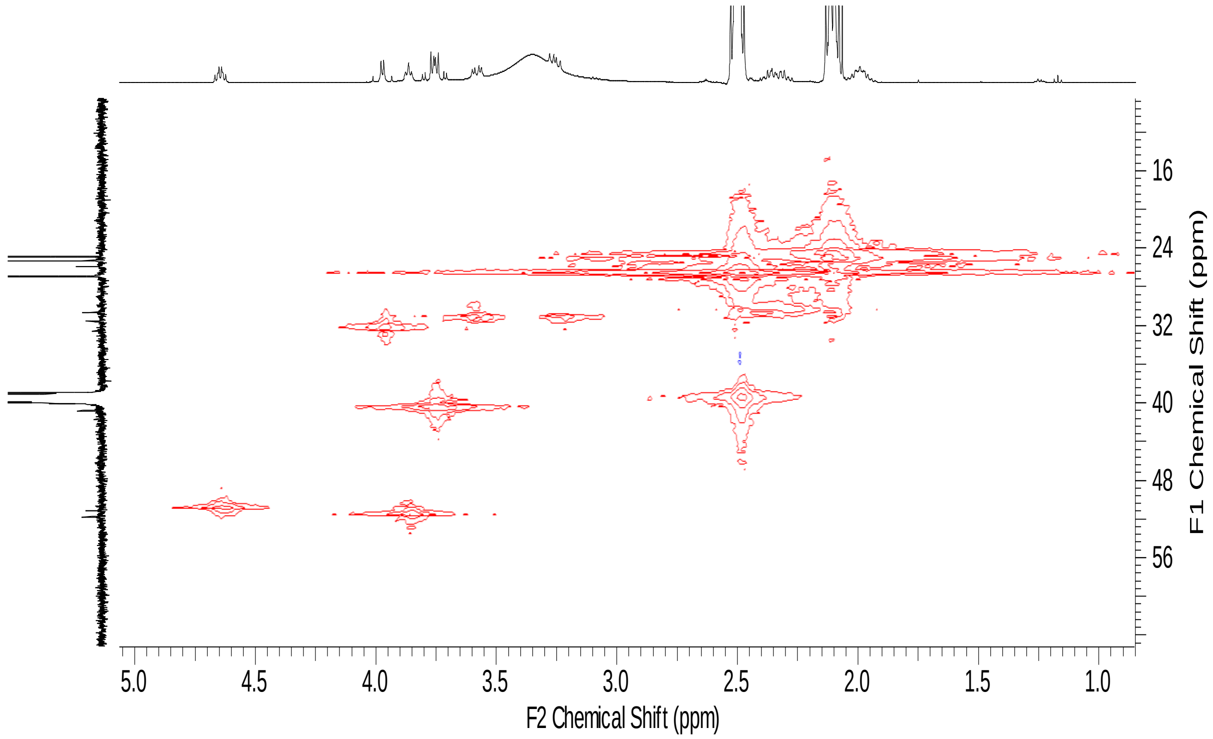

Figure S117. HSQC spectrum of TMT(AcOH)(glutathione)$_2$

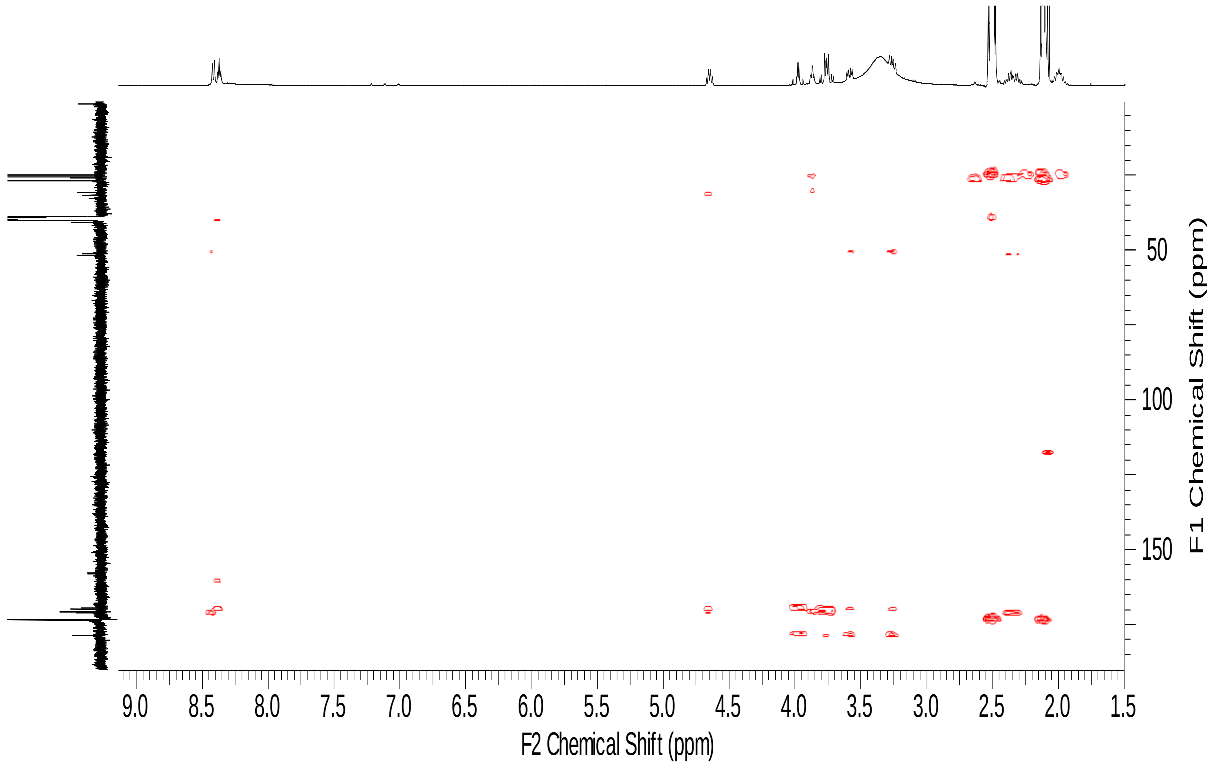

Figure S118. HMBC spectrum of TMT(AcOH)(glutathione)$_2$

TMT(glutathione)$_3$ – *S,S',S''*-(1,3,5-triazine-2,4,6-triyl}trisulfanyl)tris(glutathione)

HPLC: 10.4 min (column – Aeris 3.6 µm PEPTIDE XB-C18 50 x 2.1 mm; detection -D, 240 nm; gradient - 1%B for 5 min, 10-30%B 6-20 min, 30-100%B 21-25 min; flowrate 0.2 mL/min; eluent A – 0.1% formic acid in water, eluent B – 0.1% formic acid in acetonitrile)

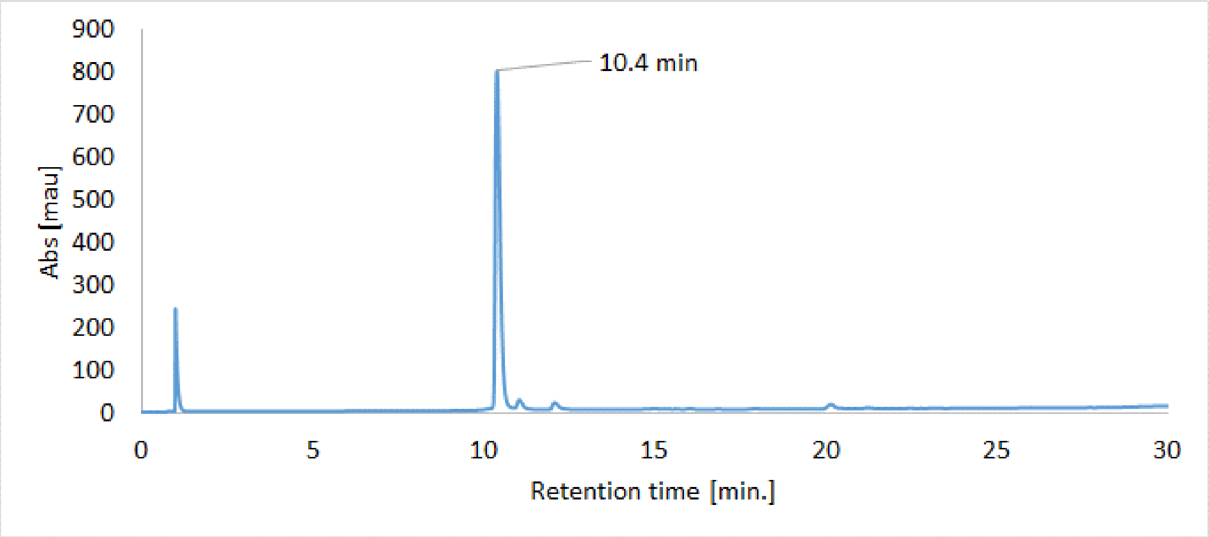

Figure S119. RP-HPLC chromatogram of purified TMT(glutathione)$_3$

HRMS (ESI-MS) m/z: $[M+H]^+$ Calcd for $C_{33}H_{49}N_{12}O_{18}S_3$ 997.2444; Found 997.2457, $[M-H_2O+H]^+$ Calcd for $C_{33}H_{47}N_{12}O_{17}S_3$ 979.2344; Found 979.2457, $[M+2H]^{2+}$ Calcd for $C_{33}H_{50}N_{12}O_{18}S_3$ 499.1259; Found 499.1292

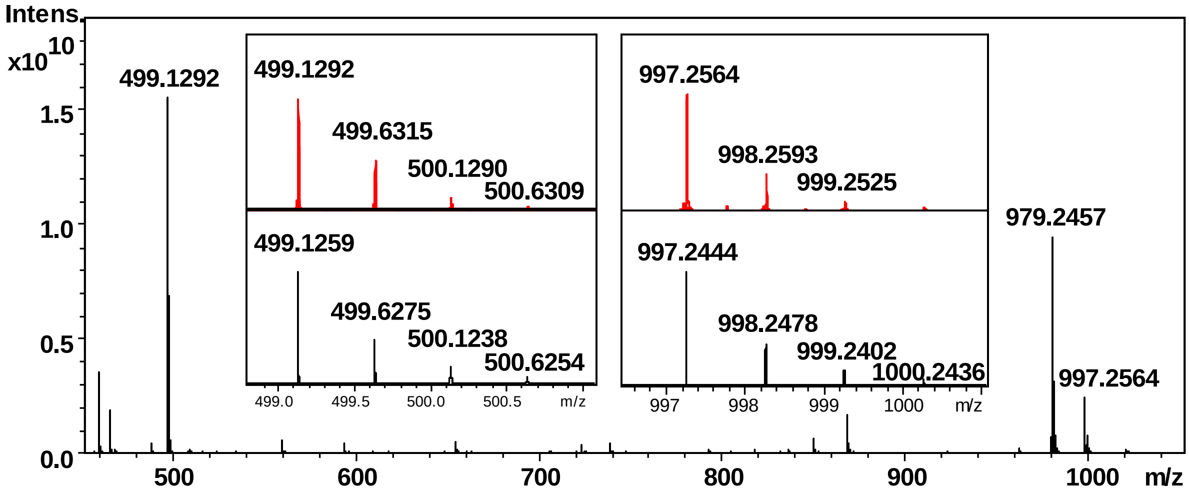

Figure S120.ESI-MS spectrum in positive ion mode of TMT(glutathione)$_3$, comparison of measured (red) and simulated (black) isotopic patterns for $[M+2H]^{2+}$ ion (left) and $[M+H]^+$ ion (right).

ESI-MS(+)/MS, m/z: 721.1550 ([$b_2/b_2/b_2$-NH_3-H_2S]$^+$, Calcd 721.1341); 706.1442 ([b_2/M/M-ΔGSH]$^+$, Calcd 706.1383); 664.1332 ($b_2/y_2/y_2$, Calcd 664.1278Da); 610.1223 ($y_2/y_2/y_2$, Calcd 610.1167); 595.1108 ([y_2/M/M-ΔGSH]$^+$, Calcd 595.1058); 577.0998 ([y_2/b_3/M-ΔGSH]$^+$, Calcd 577.0957); 561.1227 ([y_2-H_2S/b_3/M-ΔGSH]$^+$, Calcd 561.1180); 543.1126 ([$b_2(y_2)$-H_2S/M/M-ΔGSH]$^+$, Calcd 543.1080); 520.0752 ([$b_1(y_2)$-H_2S/M/M-ΔGHS]$^+$, Calcd 520.0743); 466.0671 ([y_2/y_2/M-ΔGHS]$^+$, Calcd 466.0632); 432.0790 ([y_2/y_2-H_2S/M-ΔGHS]$^+$, Calcd 432.0760); 390.0569 (unknown); 370.0864 ([y_2/y_2/M+H]$^{2+}$, Calcd 370.0833)

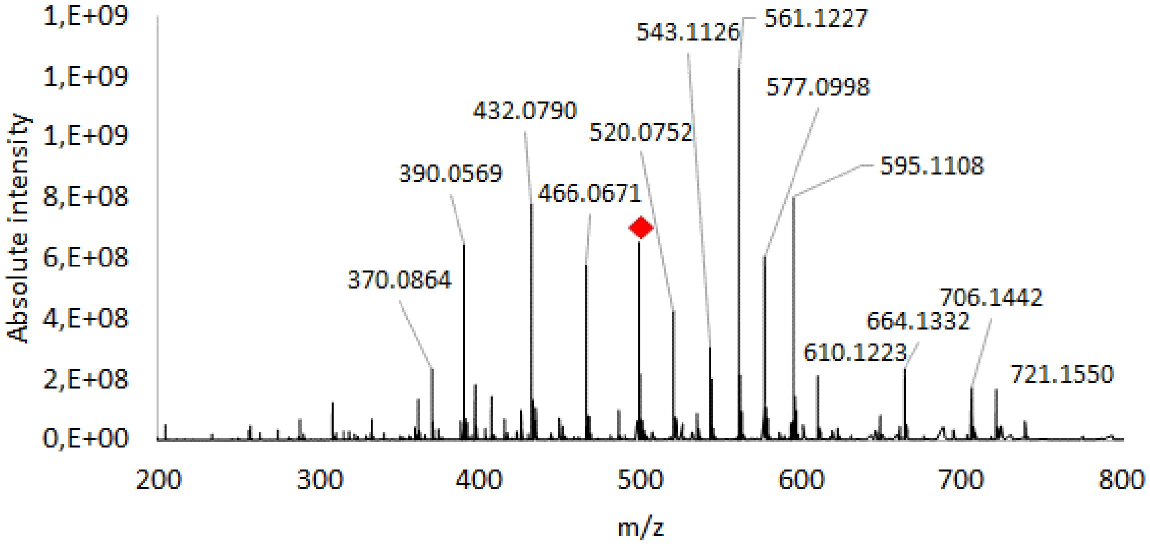

Figure S121. CID fragmentation MS[2] spectrum of TMT(glutathione)$_3$, [M+2H]$^{2+}$ parent ion.

1H NMR (DMSO-d6, 500 MHz, 25°C) δ (ppm) = 8.43 (t, 1H, NH, Gly), 8.40 (d, 1H, NH, Cys), 4.67 (m, 1H, HA, Cys), 3.76 (ddd, 2H, HB#, Gly), 3.72 (m, 1H, HA, Glu), 3.58 (dd, 1H, HB2, Cys), 3.30 (dd, 1H, HB1, Cys), 2.34 (m, 2H, HG#, Glu), 1.97 (m, 2H, HB#, Glu)

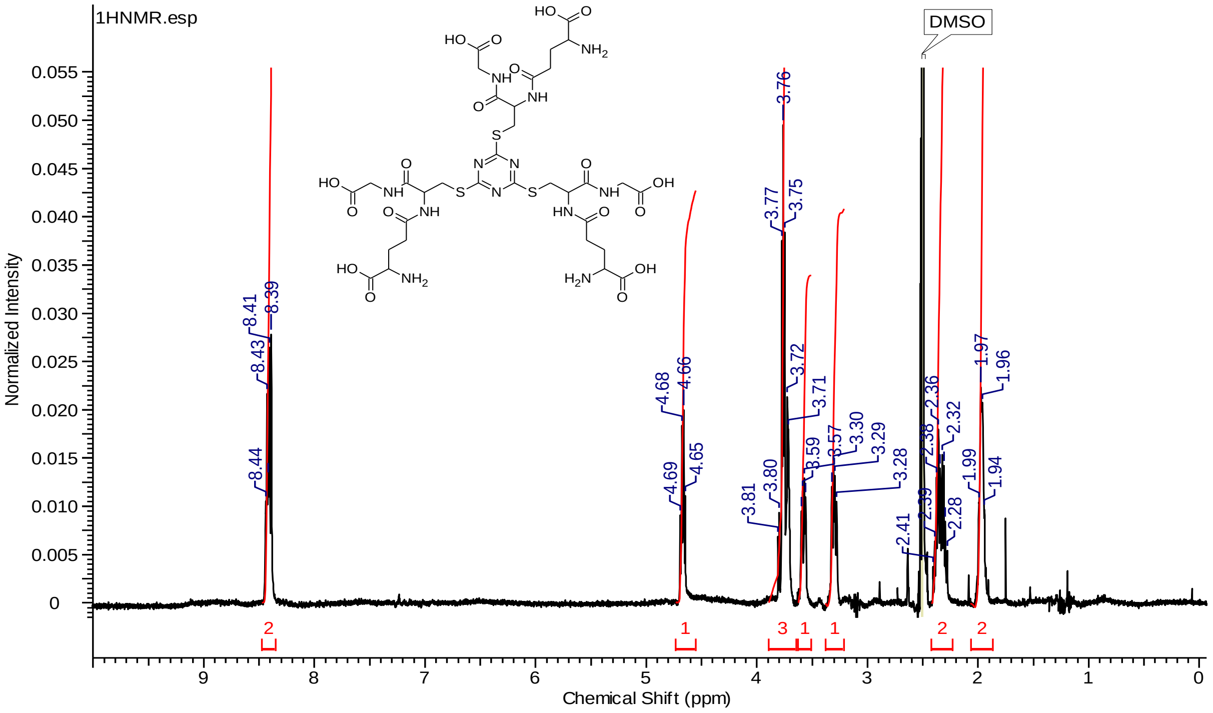

Figure S122. 1H NMR spectrum of TMT(glutathione)$_3$ in DMSO-d_6

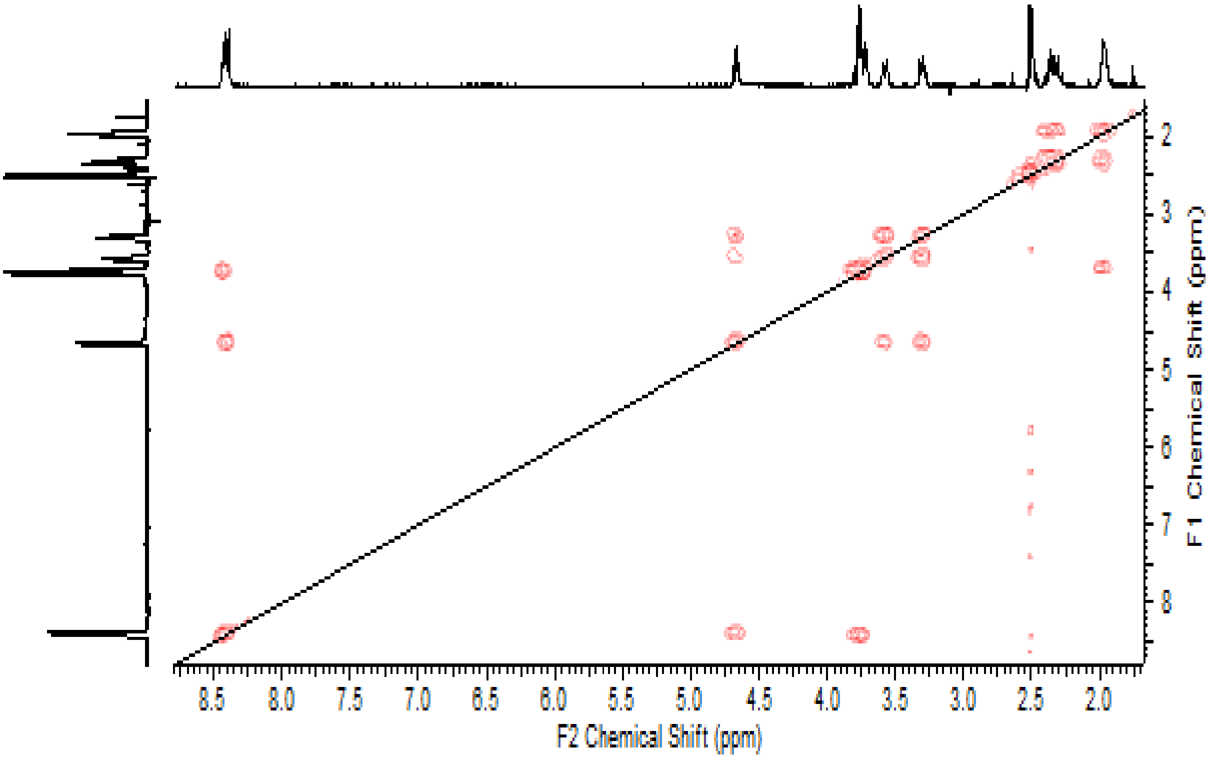

Figure S123. COSY spectrum of TMT(glutathione)$_3$

13C{H} NMR (DMSO-d6, 125 MHz, 25°C) δ (ppm) = 178.5 (C2/C4/C6), 171.2 (C11/C17/C23), 170.9 (C47/C53/C59), 170.6 (C45/C51/C57), 169.9 (C25/C31/C37), 52.1 (CA, Glu), 50.9 (CA, Cys), 40.7 (CA, Gly), 31.2 (CB, Cys), 30.9 (CG, Glu), 26.1 (CB, Glu)

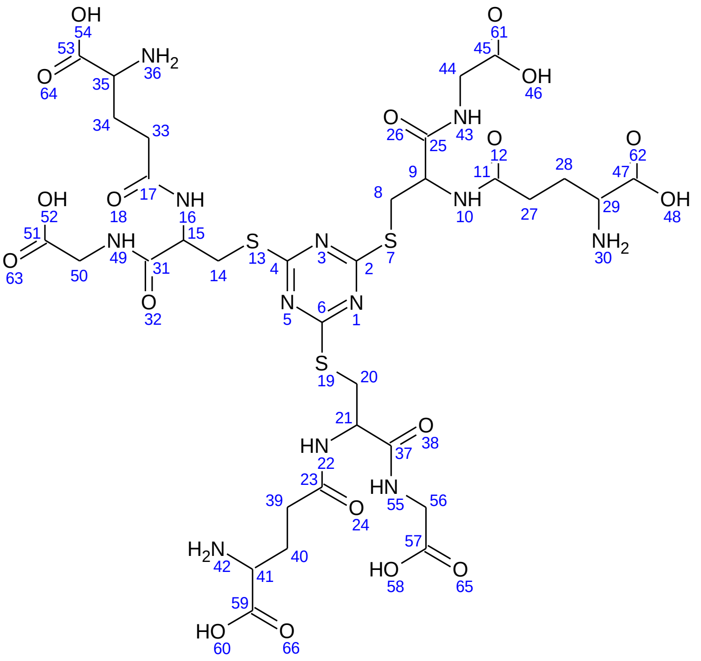

Figure S124. Numeration of atoms in TMT(glutathione)$_3$ for 13C{H} NMR assignments (based on HSQC and HMBC experiments)

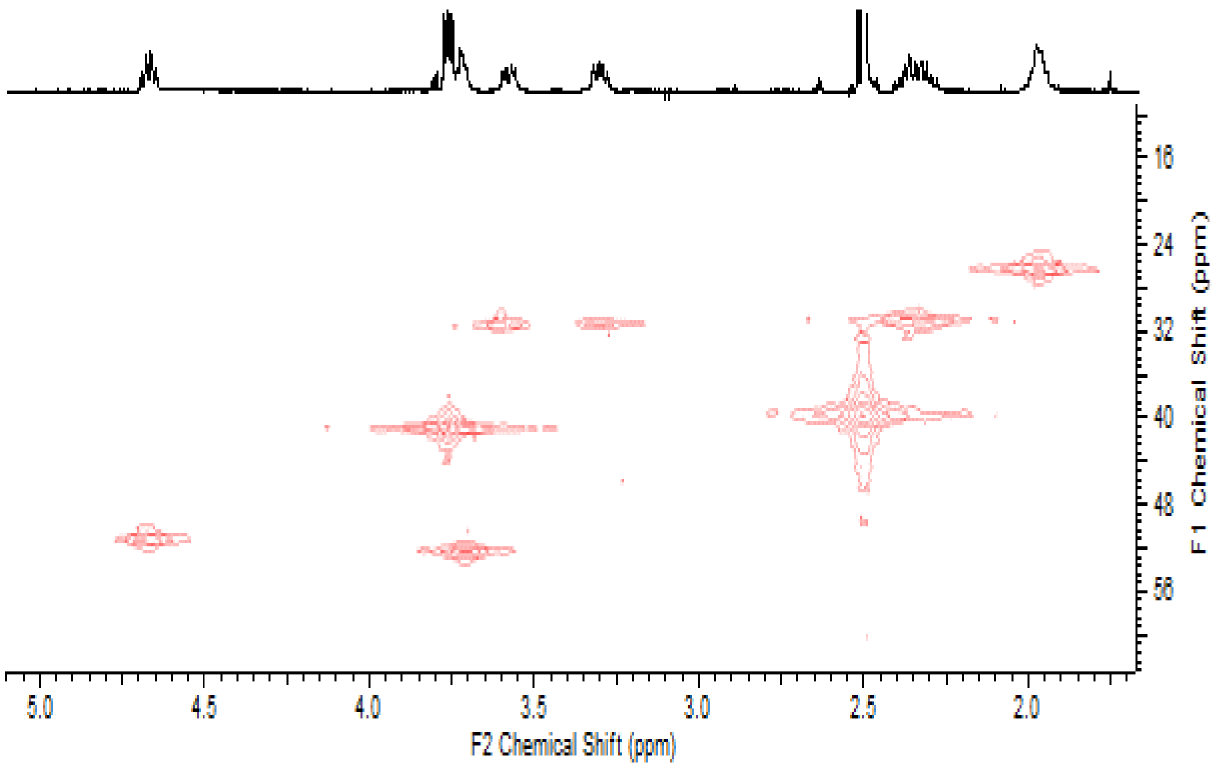

Figure S125. HSQC spectrum of TMT(glutathione)$_3$

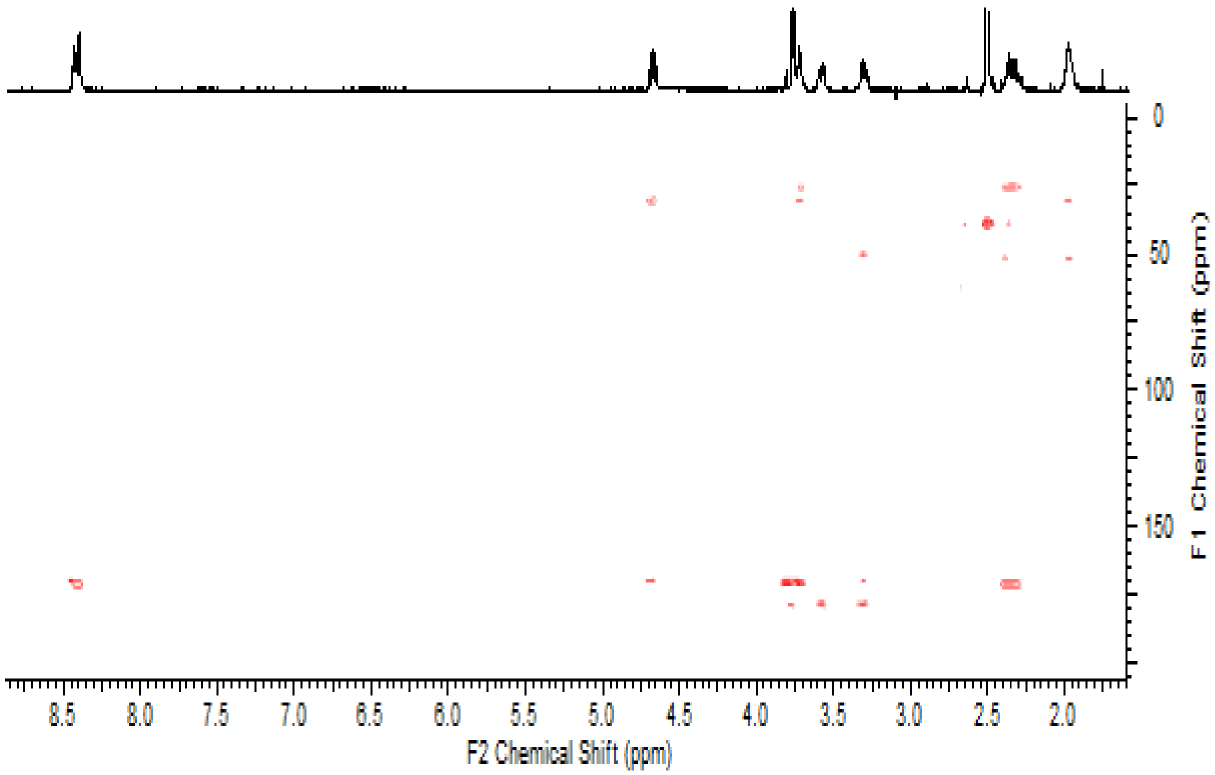

Figure S126. HMBC spectrum of TMT(glutathione)$_3$

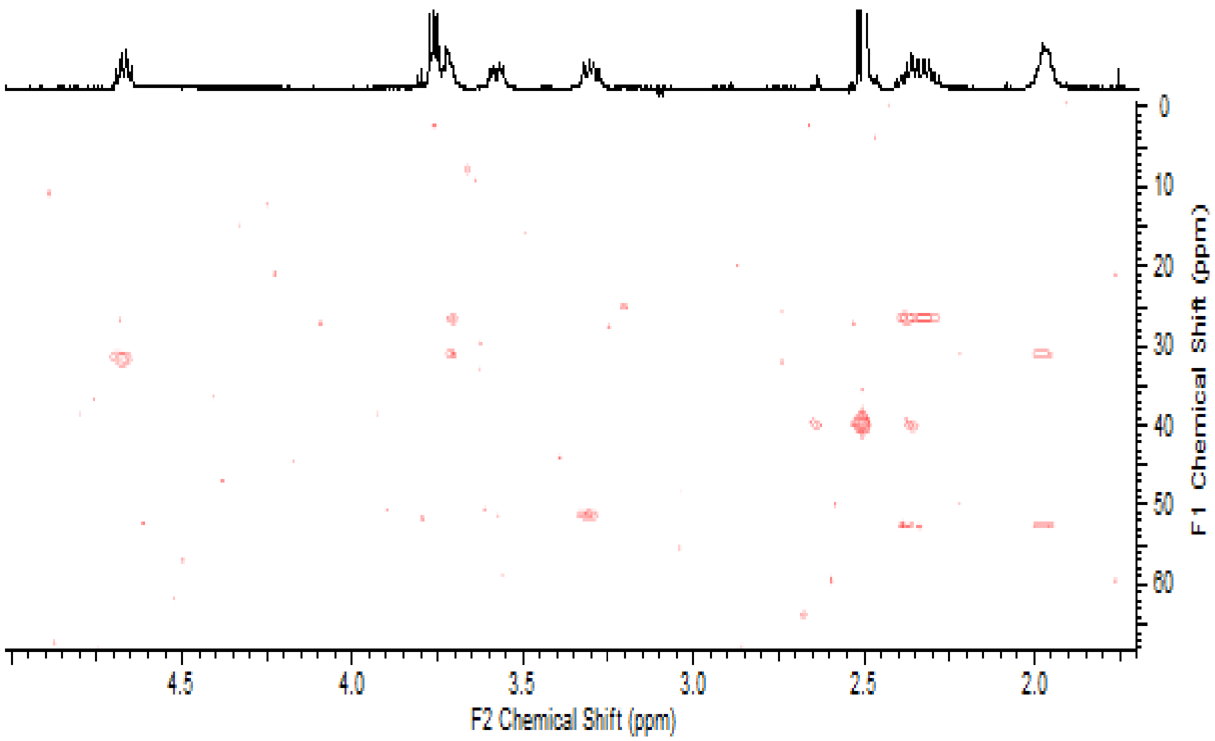

Figure S127. Correlation between aliphatic protons and aliphatic carbon atoms on HMBC spectrum of TMT(glutathione)$_3$

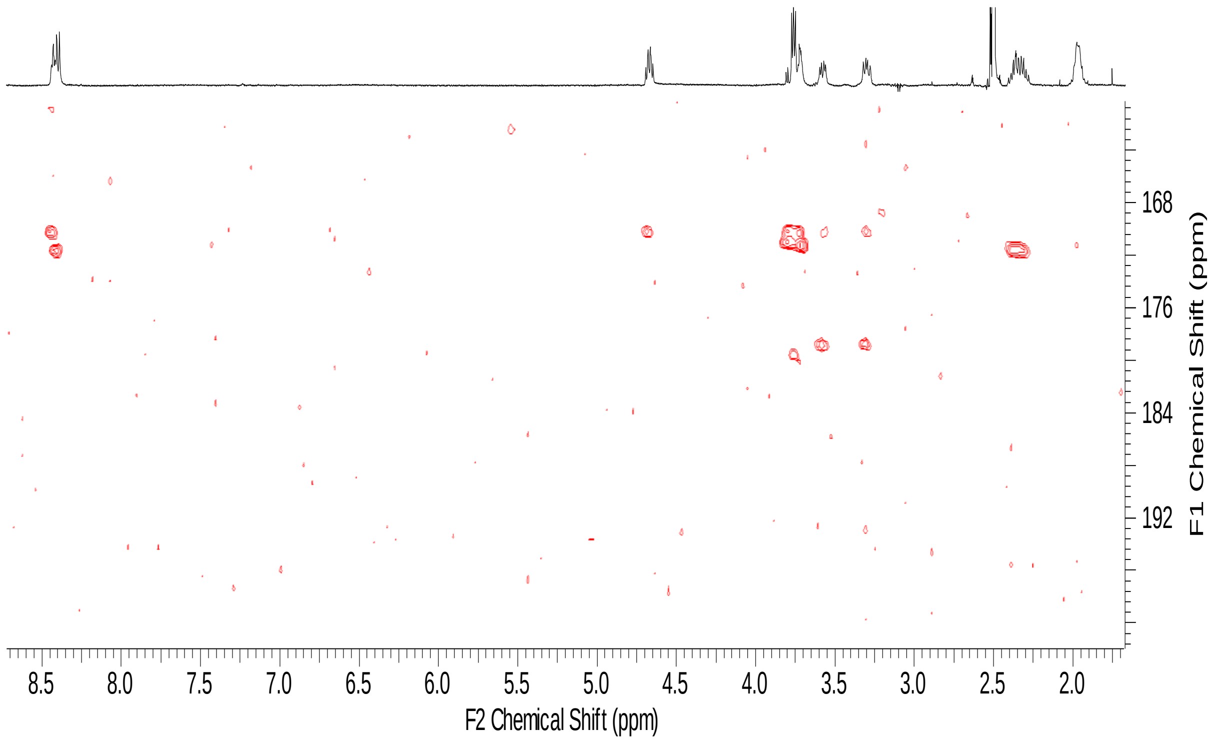

Figure S128. Correlations between aliphatic protons and carbonyl and aromatic carbon atoms on HMBC spectrum of TMT(glutathione)$_3$

5. Self-assembly of TMT(AcOH)$_X$(Glutathione)$_Y$

TMT(AcOH)2(glutathione), TMT(AcOH)(glutathione)2, and TMT(glutathione)3 were weighed separately into screw-cap glass vials and dissolved in deionized water to obtain 10mM concentration of all TMT compounds. These solutions were then incubated in a refrigerator at 5°C for 48 hours. Resulted samples were subjected to TEM imaging and circular dichroism in near ultraviolet measurements.

5.1. TEM images

TMT(AcOH)$_2$(glutathione)

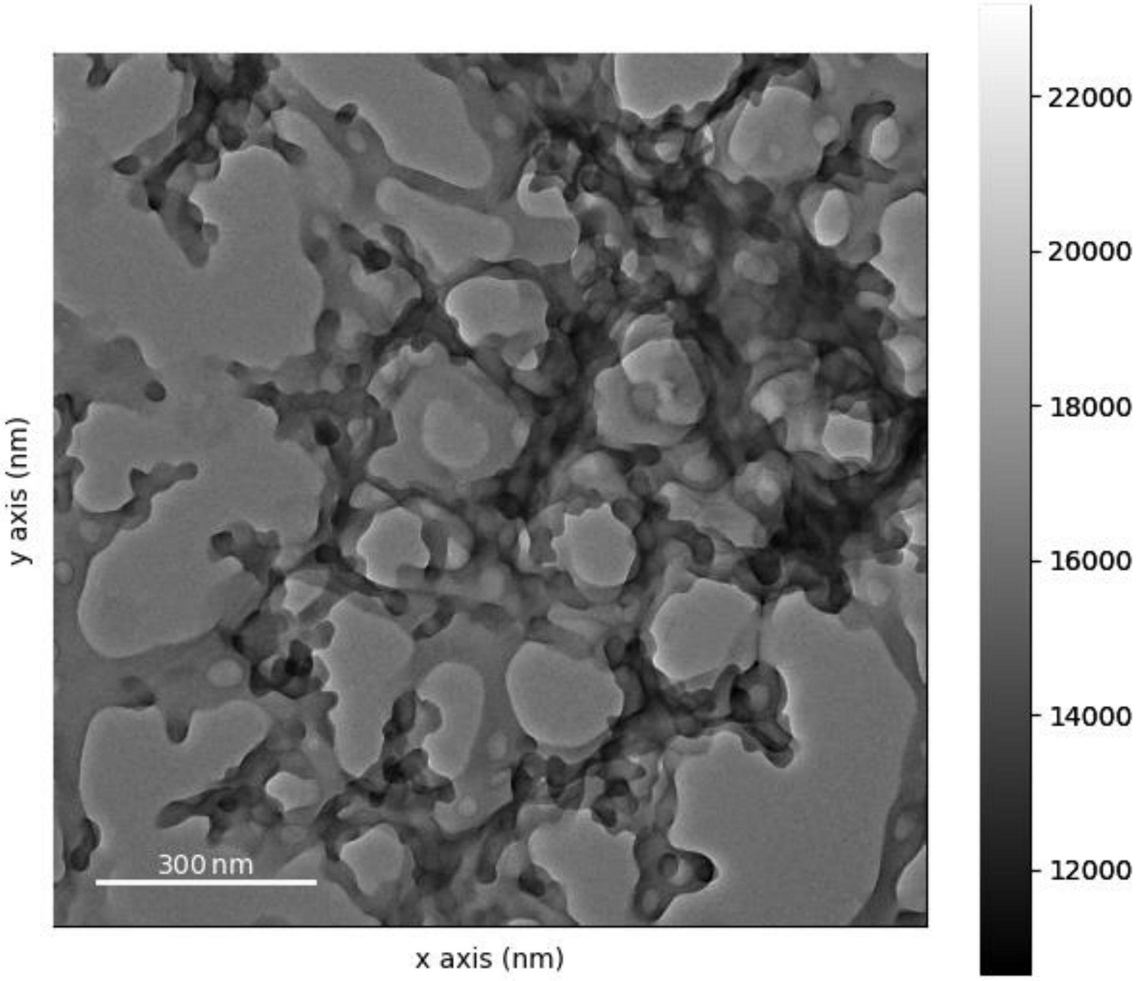

Figure S129. TEM image of assemblies formed by TMT(AcOH)$_2$(glutathione) in 300 nm scale.

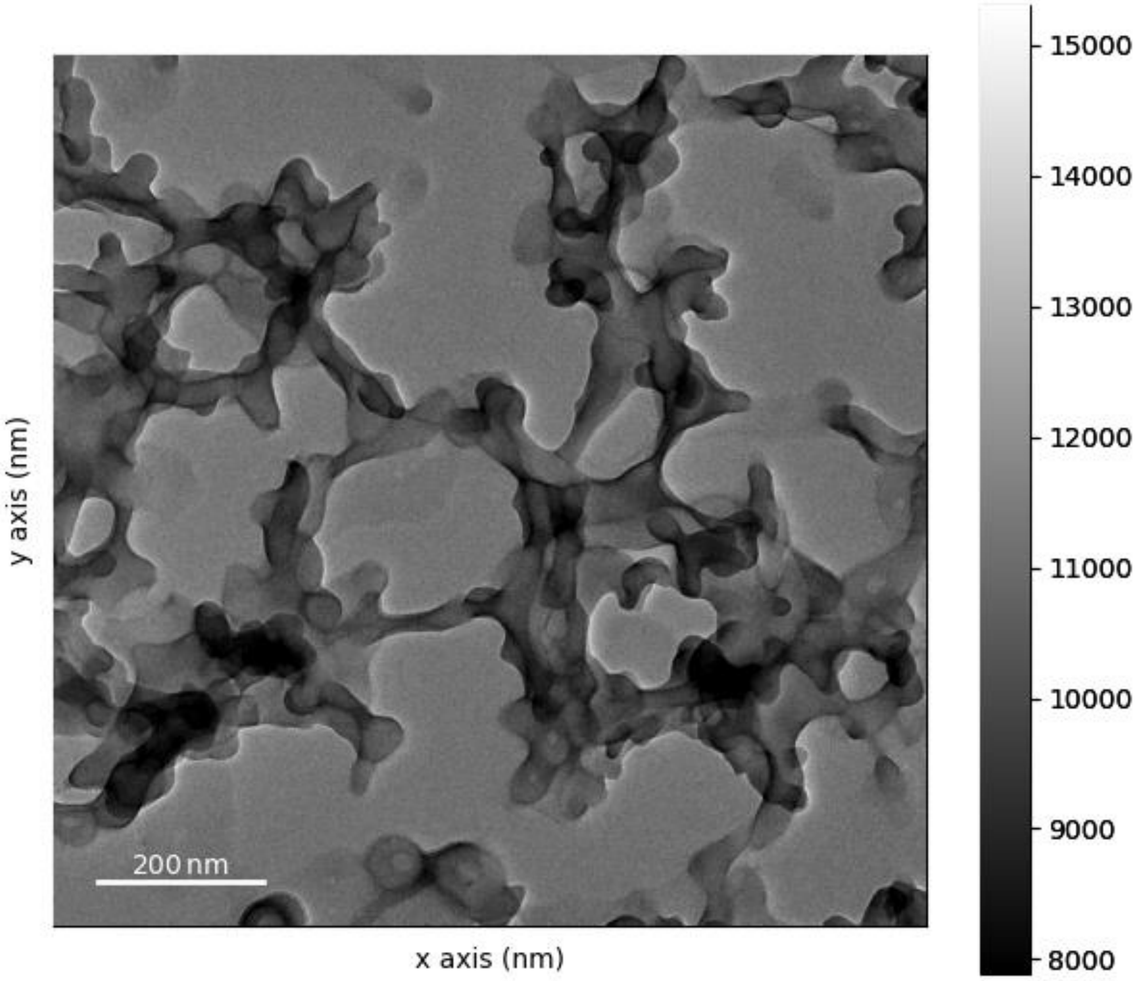

Figure S130. TEM image of assemblies formed by TMT(AcOH)$_2$(glutathione) in 200 nm scale.

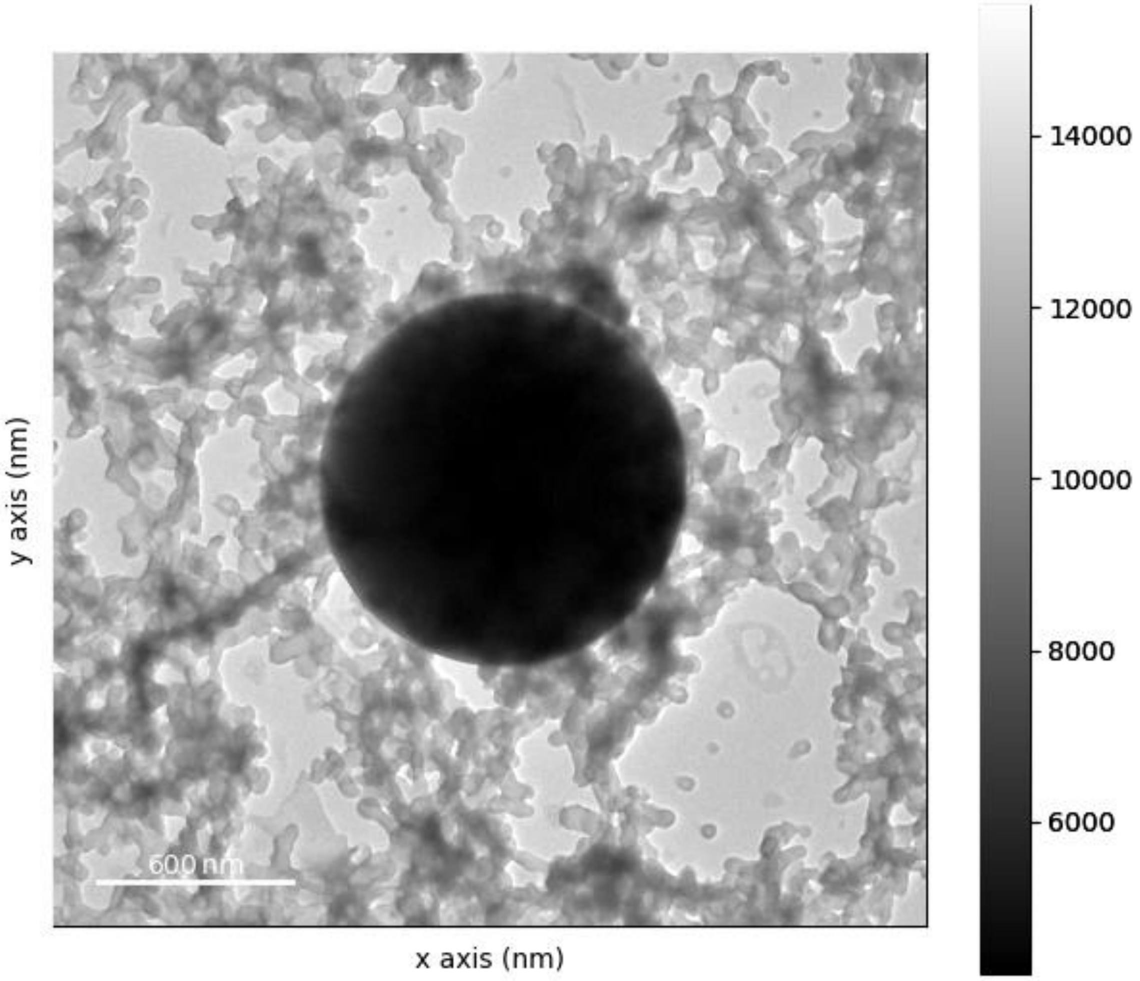

Figure S131. TEM image of assemblies formed by TMT(AcOH)$_2$(glutathione) in 600 nm scale.

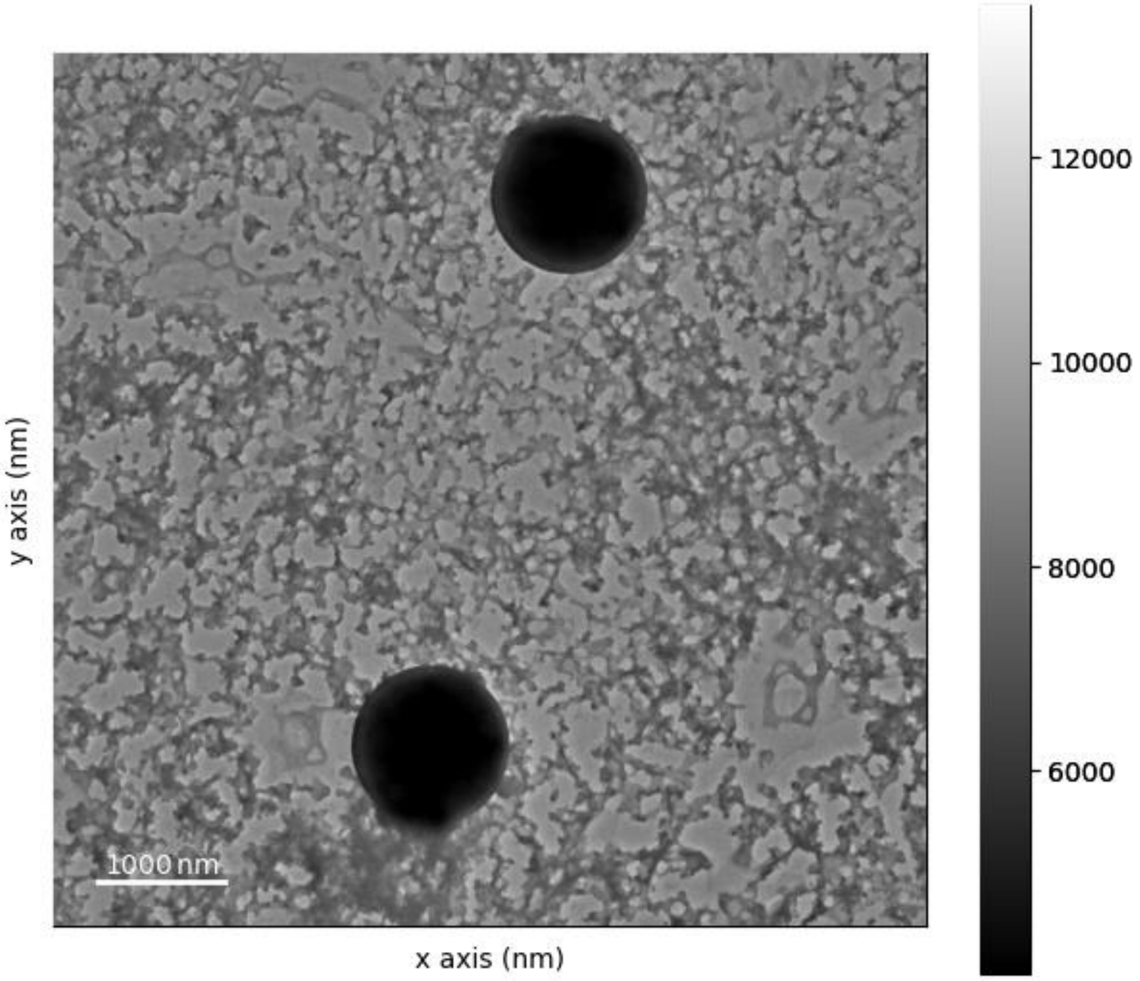

Figure S132. TEM image of assemblies formed by $TMT(AcOH)_2$(glutathione) in 1000 nm scale.

TMT(AcOH)(glutathione)$_2$

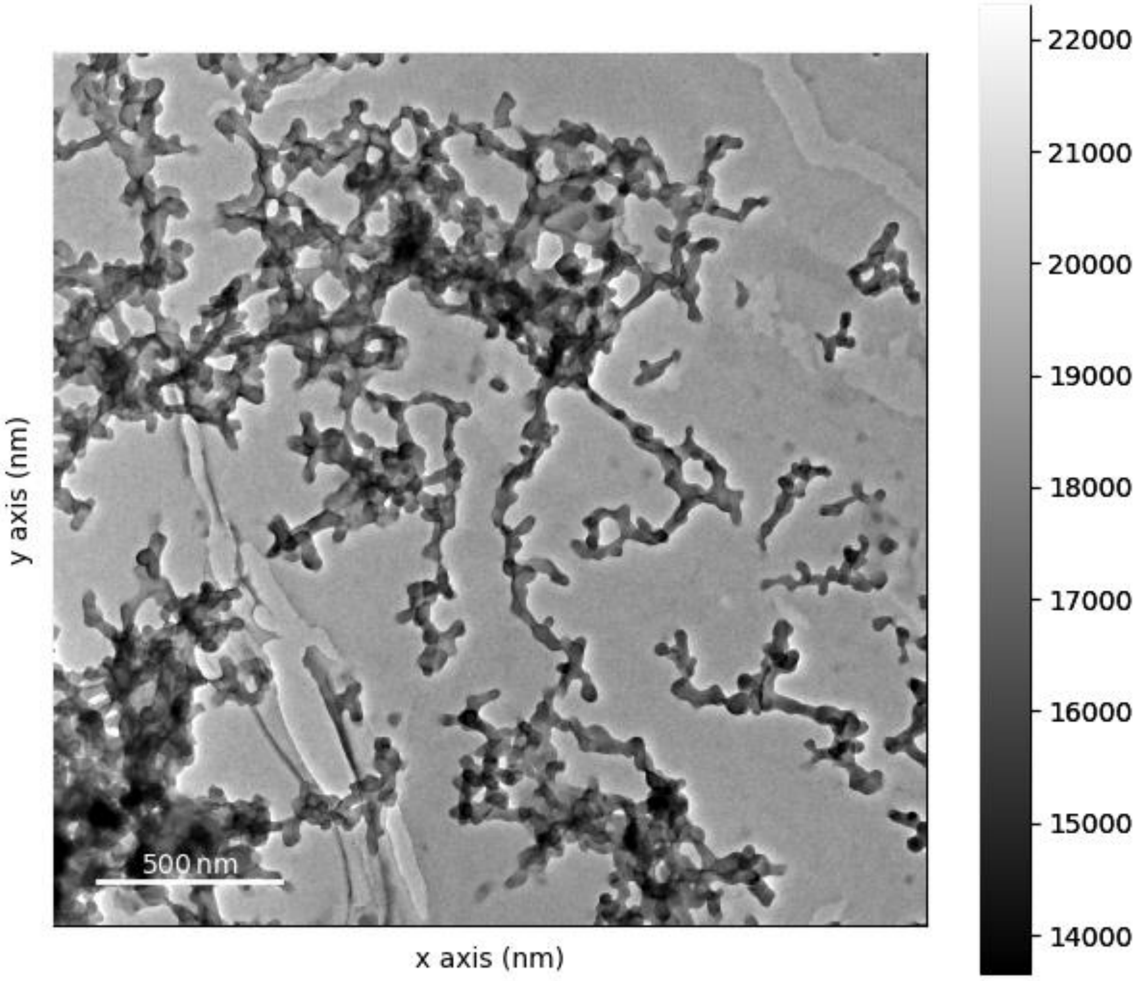

Figure S133. TEM image of assemblies formed by TMT(AcOH)(glutathione)$_2$ in 500 nm scale.

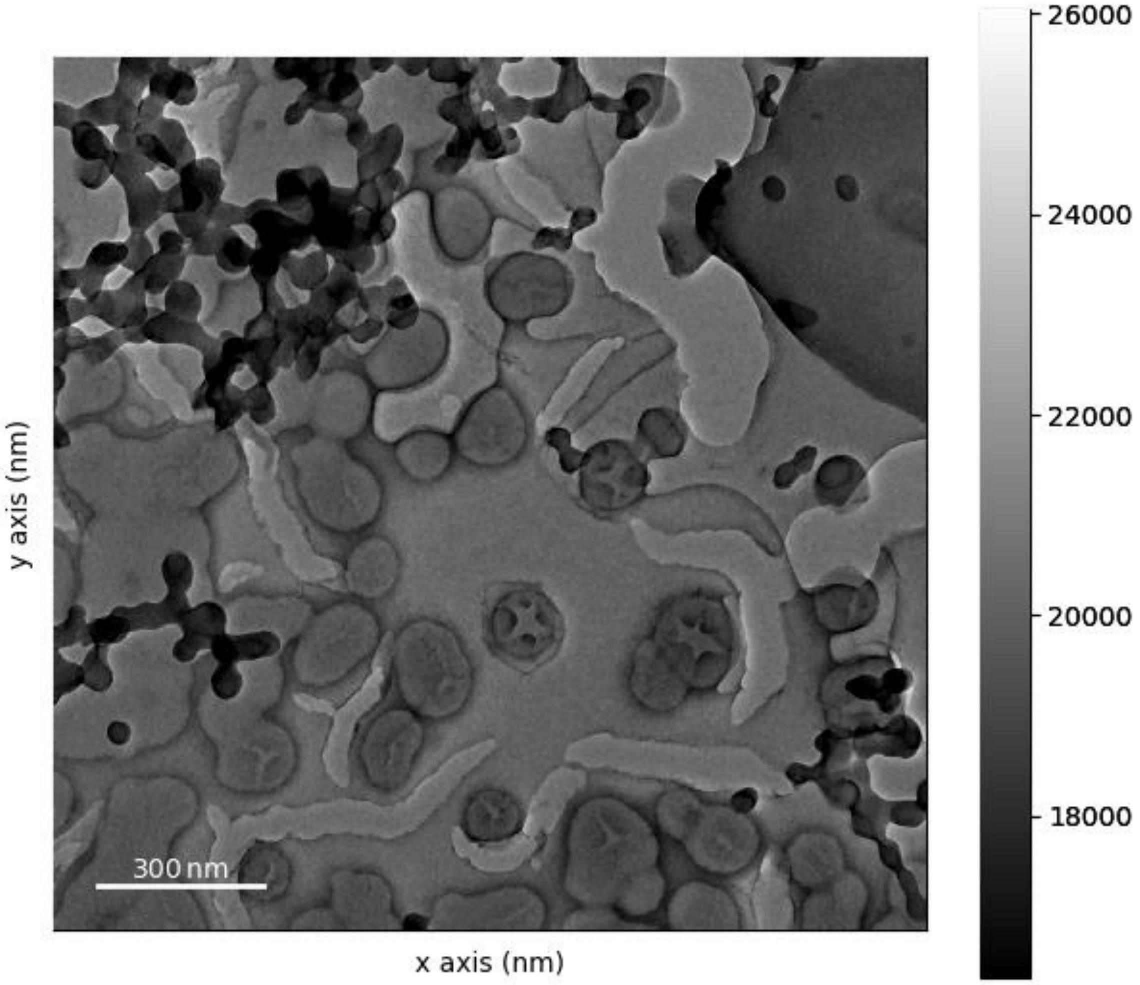

Figure S134. TEM image of assemblies formed by TMT(AcOH)(glutathione)$_2$ in 300 nm scale.

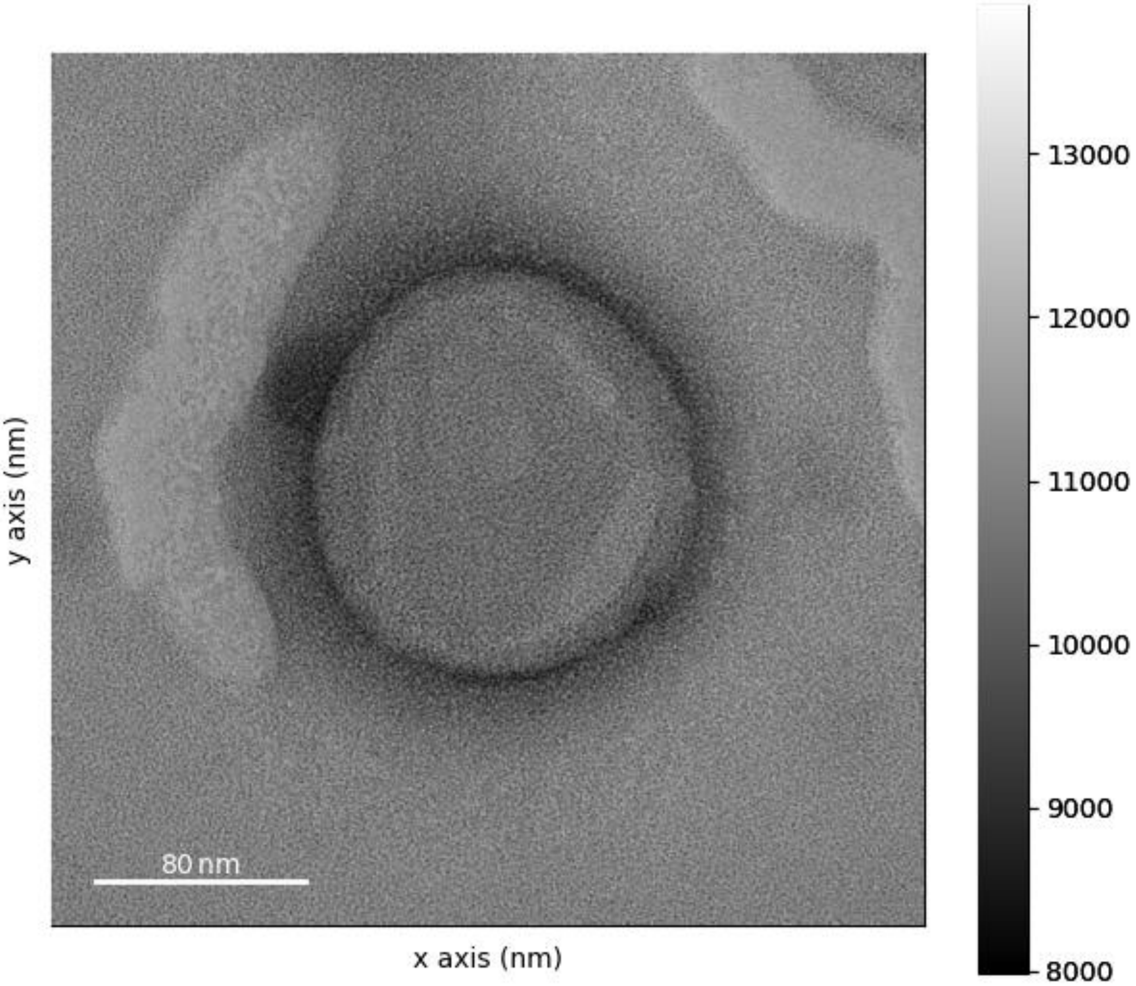

Figure S135. TEM image of assemblies formed by TMT(AcOH)(glutathione)$_2$ in 80 nm scale.

TMT(glutathione)$_3$

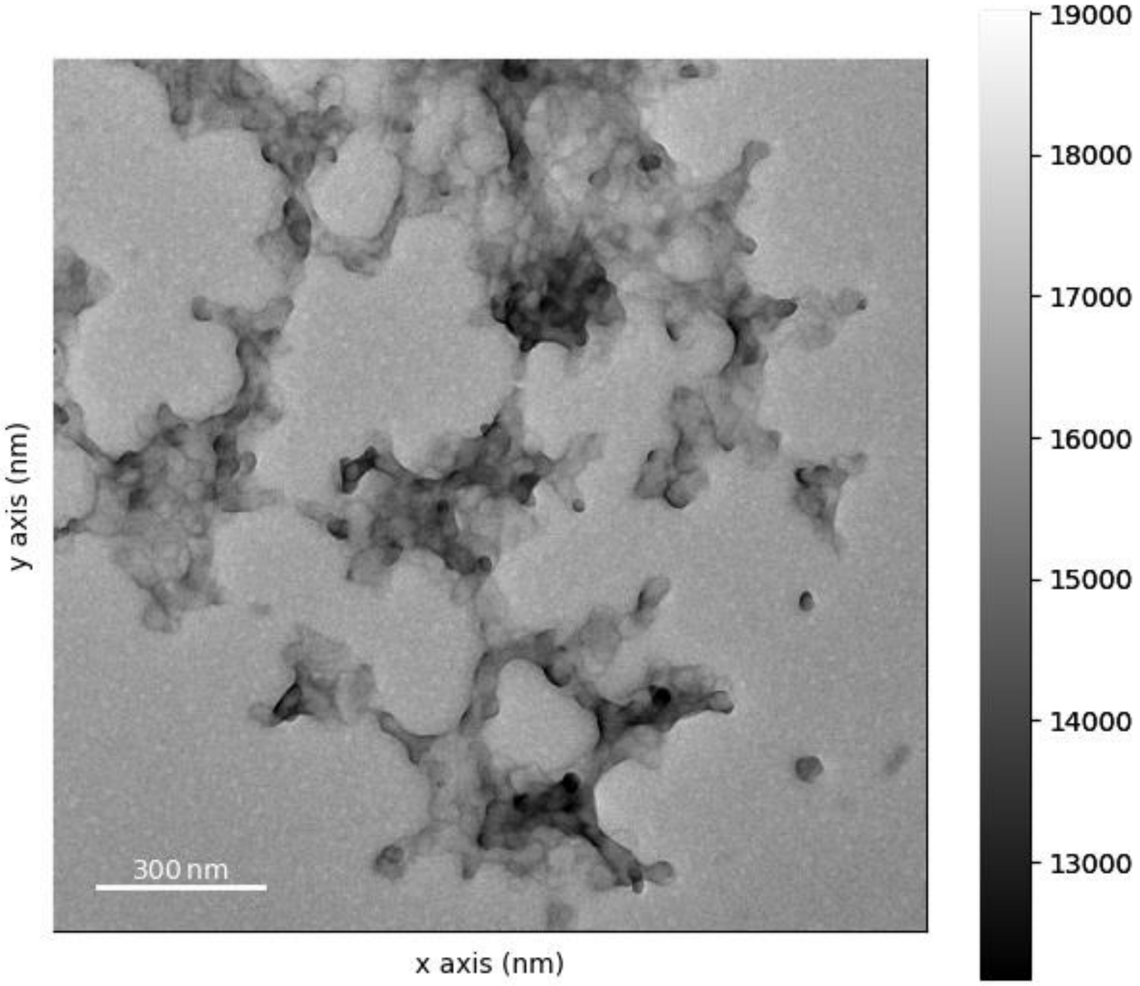

Figure S136. TEM image of assemblies formed by TMT(glutathione)$_3$ in 300 nm scale.

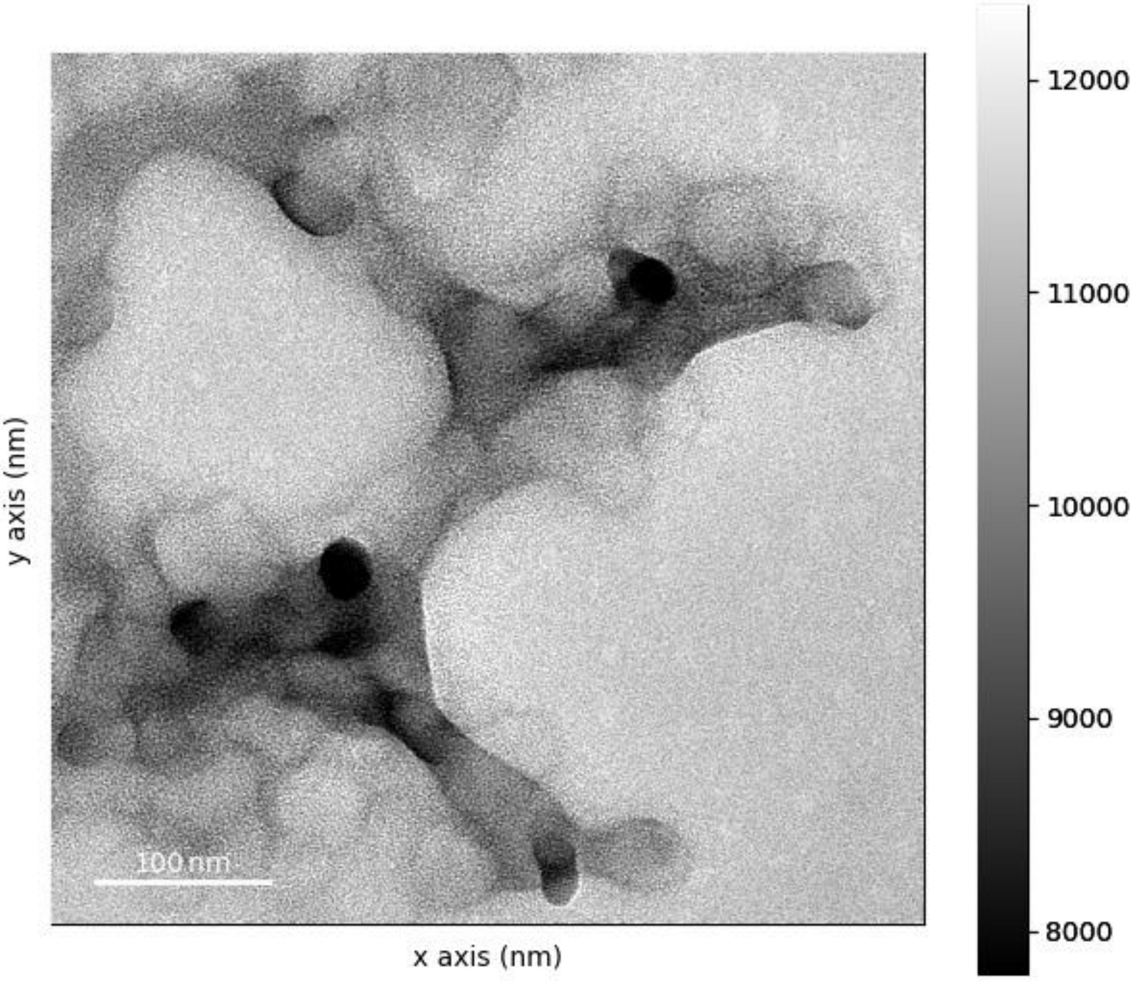

Figure S137. TEM image of assemblies formed by TMT(glutathione)$_3$ in 100 nm scale.

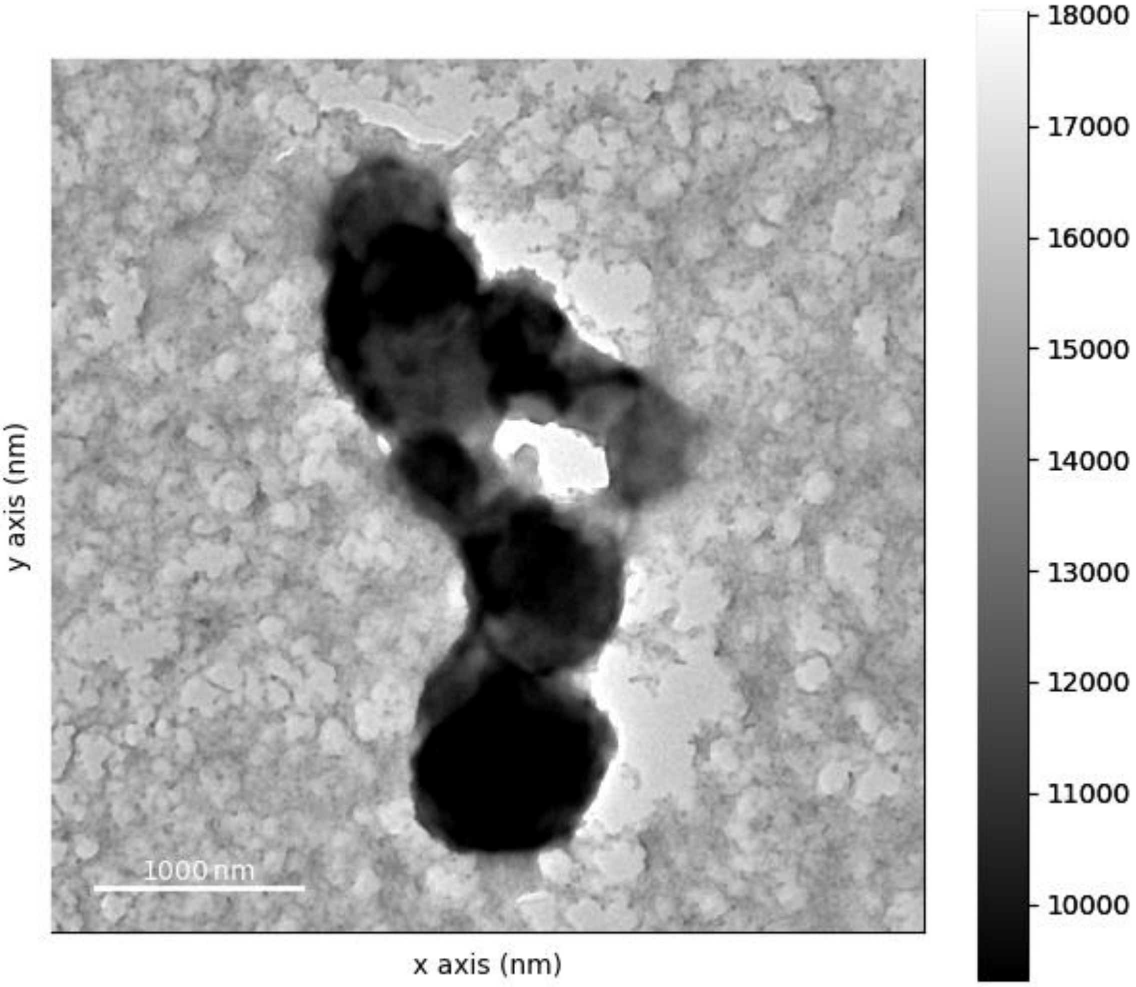

Figure S138. TEM image of assemblies formed by TMT(glutathione)$_3$ in 1000 nm scale.

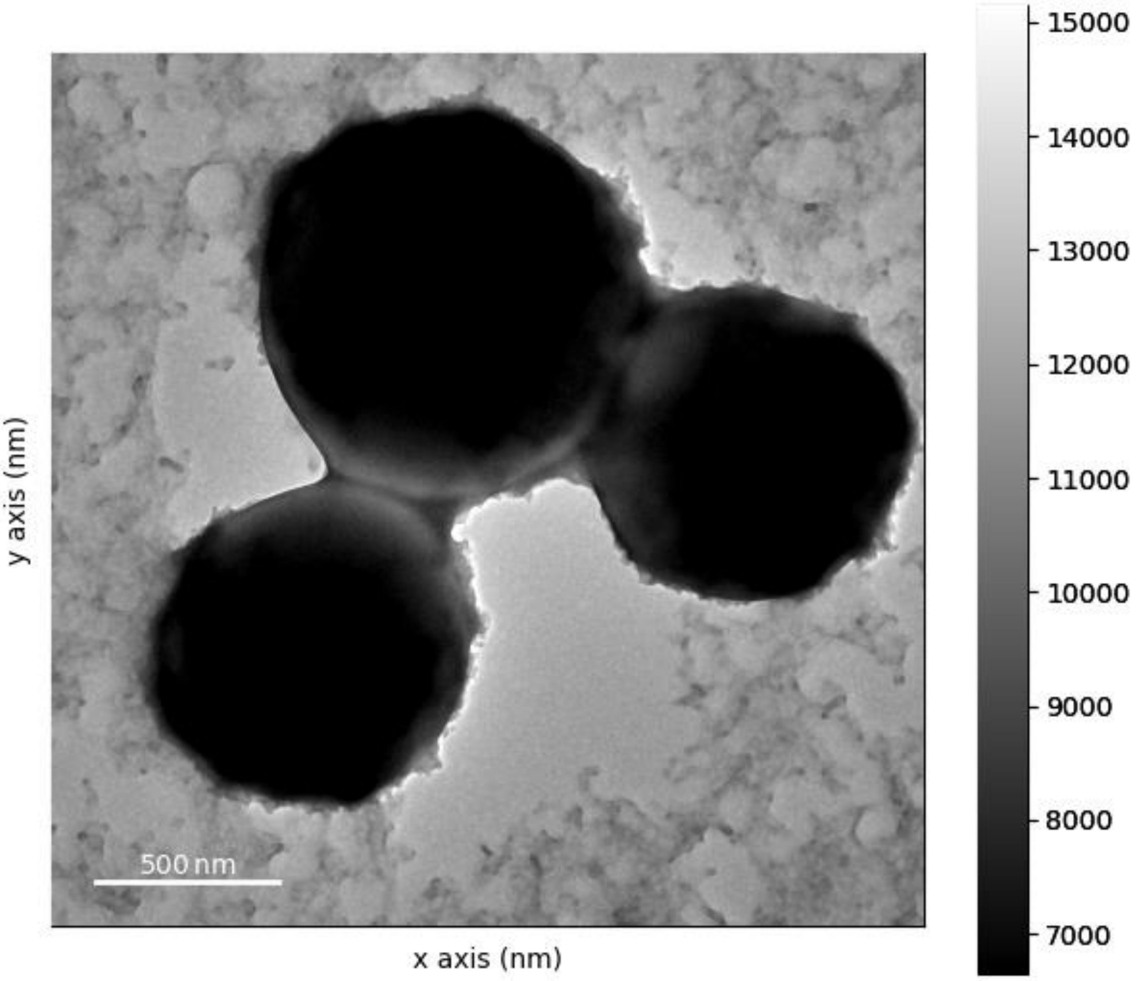

Figure S139. TEM image of assemblies formed by TMT(glutathione)$_3$ in 500 nm scale.

5.2. DLS measurements

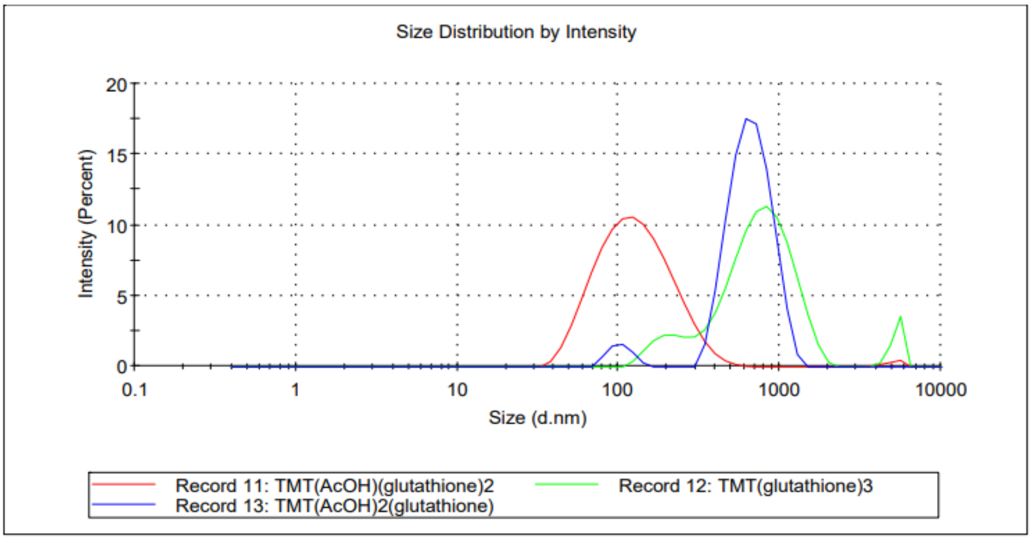

Figure S140. Size distribution of assemblies in solution by intensity for TMT(AcOH)$_X$(glutahione)$_Y$

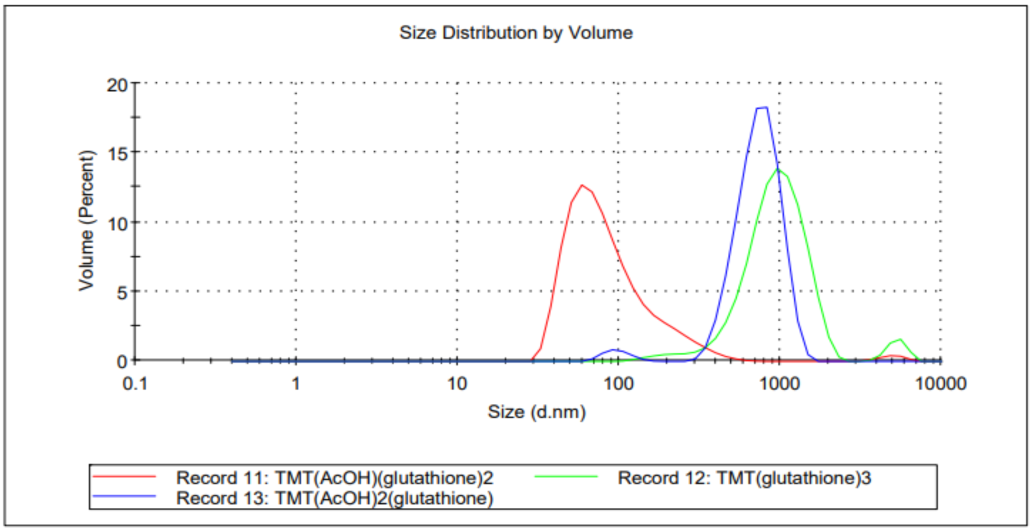

Figure S141.Size distribution in solution by volume of assemblies for TMT(AcOH)$_X$(glutahione)$_Y$

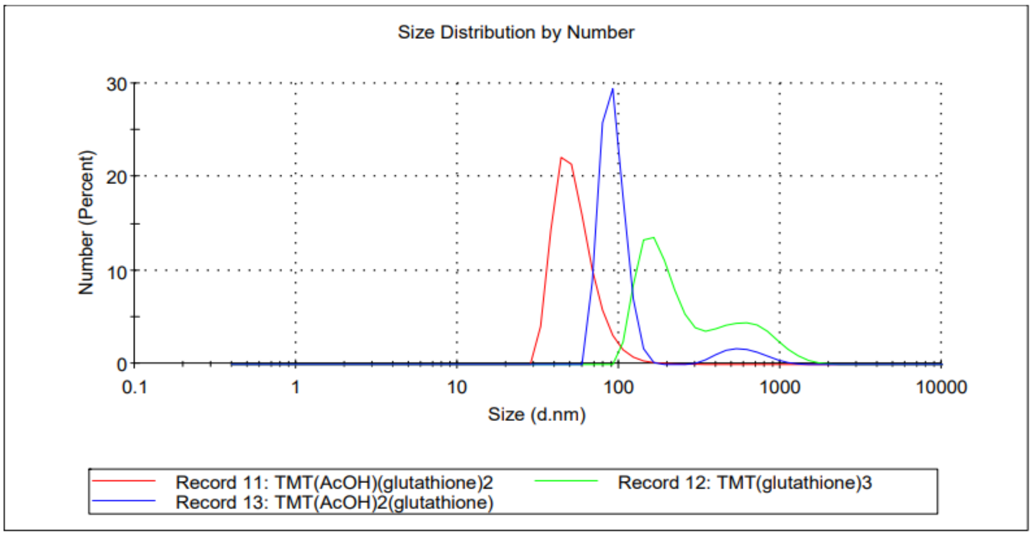

Figure S142. Size distribution in solution by number of assemblies intensity for TMT(AcOH)$_X$(glutahione)$_Y$

5.3. CD spectra

TMT(AcOH)$_2$(glutathione)

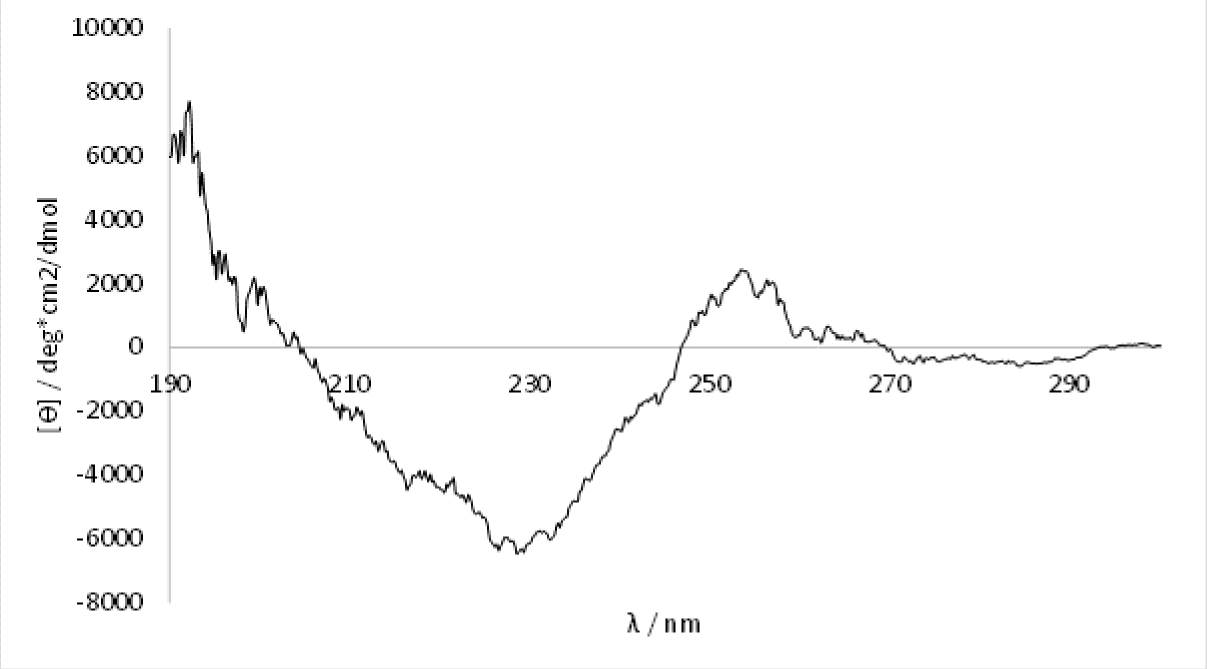

Figure S143.Circular dichroism in near UV spectrum of the aged solution of TMT(AcOH)$_2$(glutathione) in water

TMT(AcOH)(glutathione)$_2$

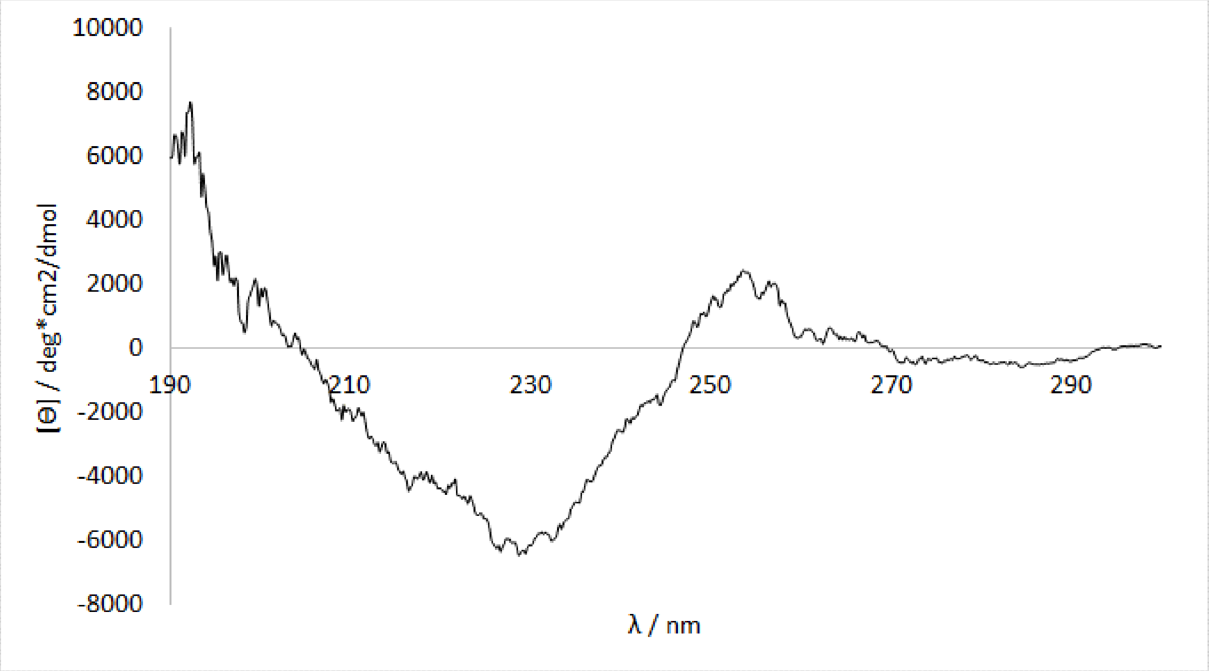

Figure S144. Circular dichroism in near UV spectrum of the aged solution of TMT(AcOH)(glutathione)$_2$ in water

TMT(glutathione)$_3$

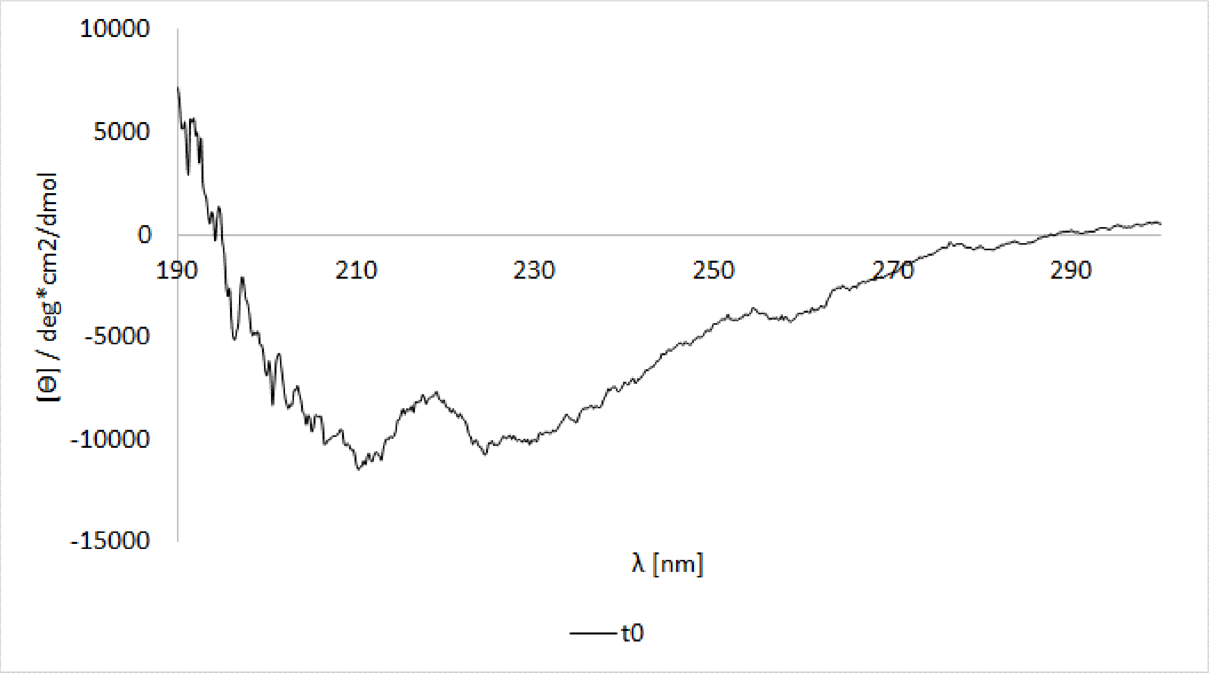

Figure S145. Circular dichroism in near UV spectrum of the aged solution of TMT(glutathione)$_3$ in water

6. The metathesis reaction of tris(carboxymethyl)thiocyanurate and tris(carboxamidomethyl)thiocyanurate

General procedure of DCL preparation: 18 mg of TMT(AcOH)$_3$ (0.05 mmol) and 18 mg of TMT(AcNH$_2$)$_3$ (0.05 mmol) were mixed and dissolved in 10 mL of 10% acetic acid, obtaining the stock solution with both components in 5mM concentration. The stock solution was divided into 1mL aliquots into glass vials and freeze-dried. Then, the samples were dissolved in 1 mL of an appropriate 0.5M phosphate buffer at pH 3, 7, or 9, containing 1.5 mM MESNa or MPAA and 20mM TCEP. The samples were incubated for 24 hours stirring on a rotary stirrer at room temperature or in a laboratory oven at 40°C. Samples for the HPLC analysis were prepared by 10-fold dilution with water.

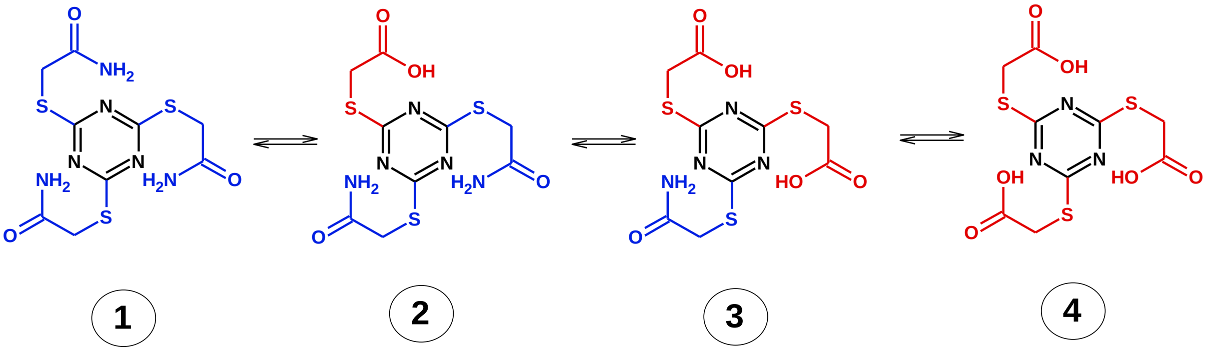

Scheme S6. Possible products in metathetic dynamic library formed by mixing of TMT(AcOH)$_3$ and TMT(AcNH$_2$)$_3$ in the presence o catalytic thiols

6.1. Influence of different pH and temperatures

HPLC/LC-MS method: column – ReproSil-XR 120 C18-MS, 3 µm, 100 x 2 mm; detection – UV 254 nm and ESI-MS(+) IT-TOF Shimadzu; gradient 1%B 0-5 min, 1-10% 5-6min, 10-30% 6-20 min; flowrate – 0.2 mL/min.; eluent A – 0.1% formic acid in the water, eluent B – 0.1% formic acid in acetonitrile

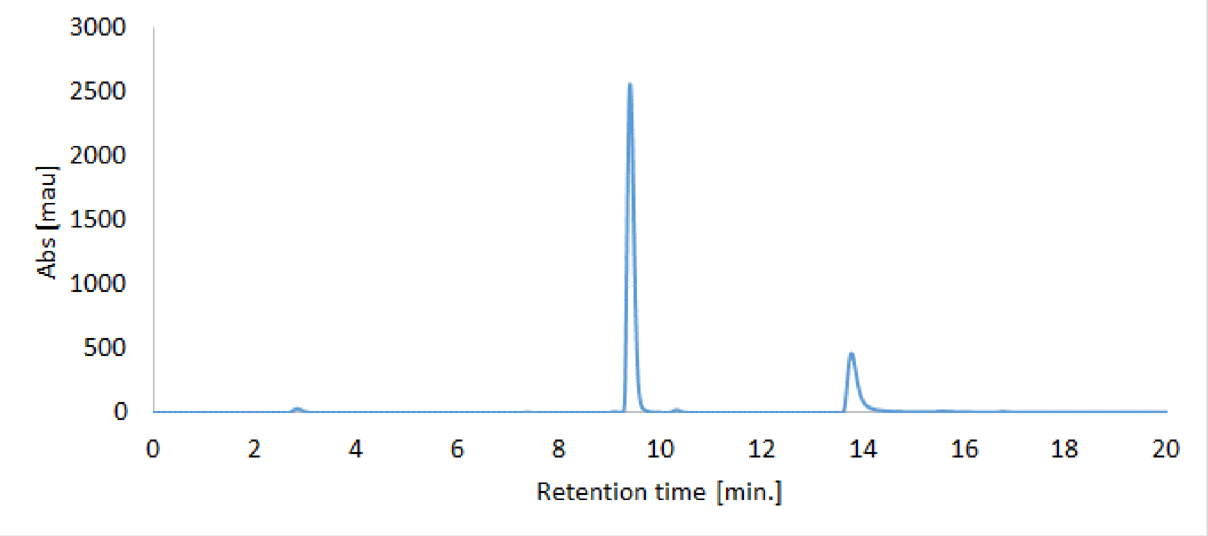

*Figure S146. RP-HPLC chromatogram of DCL at pH 3 and 40°C, **MESNa** as catalyst, detection at 254 nm*

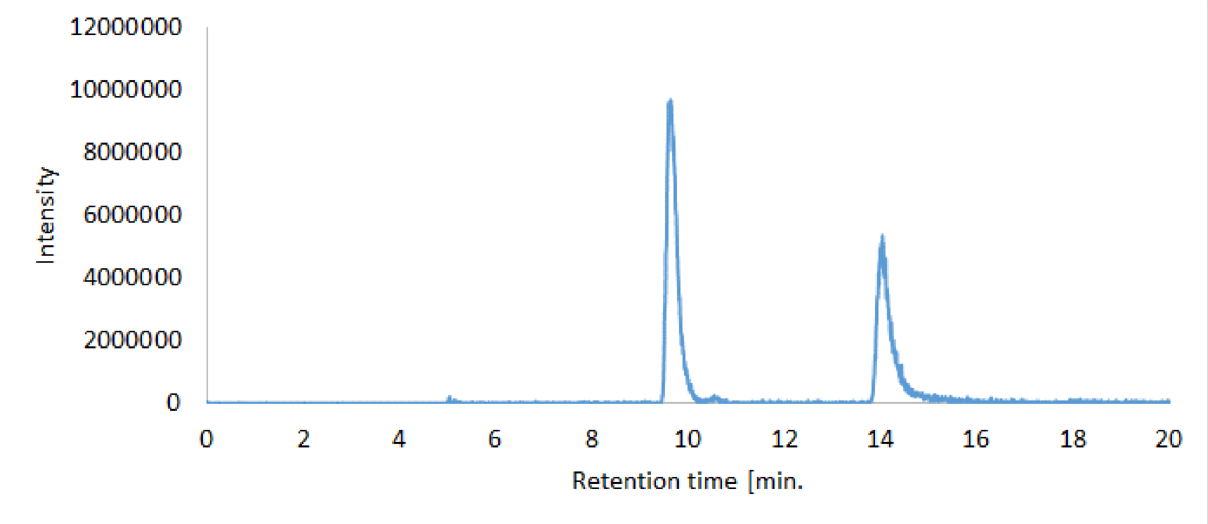

*Figure S147. Extracted ion chromatogram of DCL at pH 3 and 40°C, **MESNa** as catalyst, m/z range <348,355>*

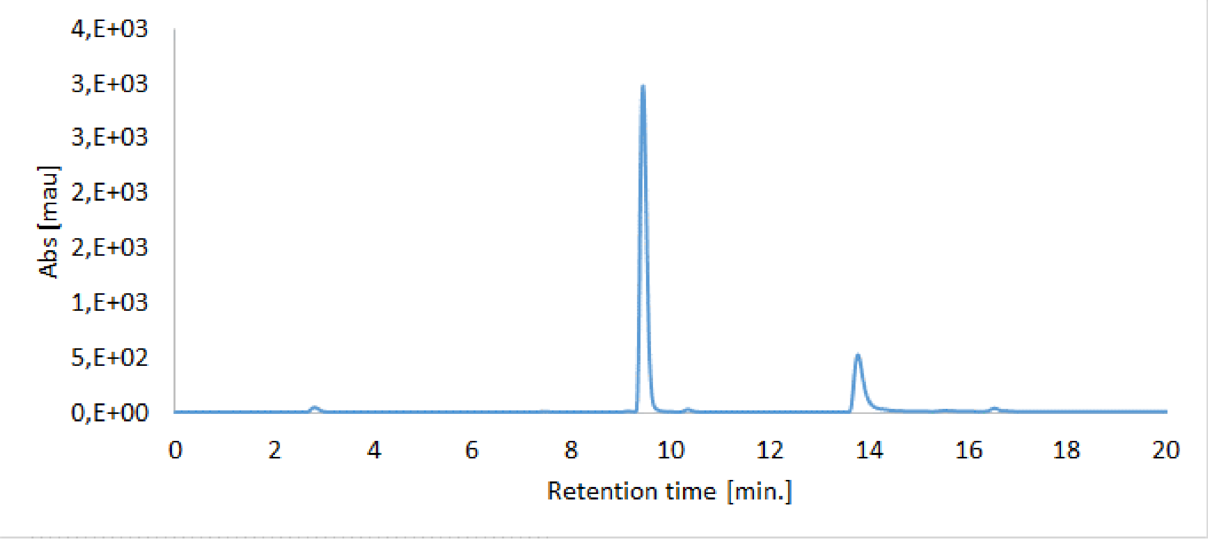

Figure S148. RP-HPLC chromatogram of DCL at pH 3 and 40°C, ***MPAA*** *as catalyst, detection at 254 nm*

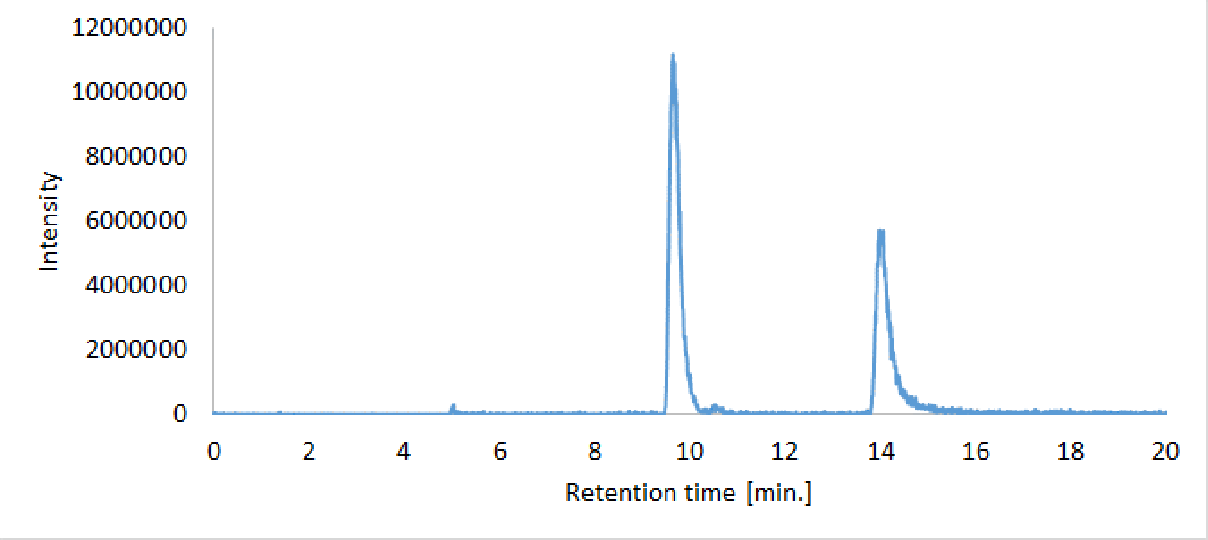

Figure S149. Extracted ion chromatogram of DCL at pH 3 and 40°C, ***MPAA*** *as catalyst, m/z range <348,355>*

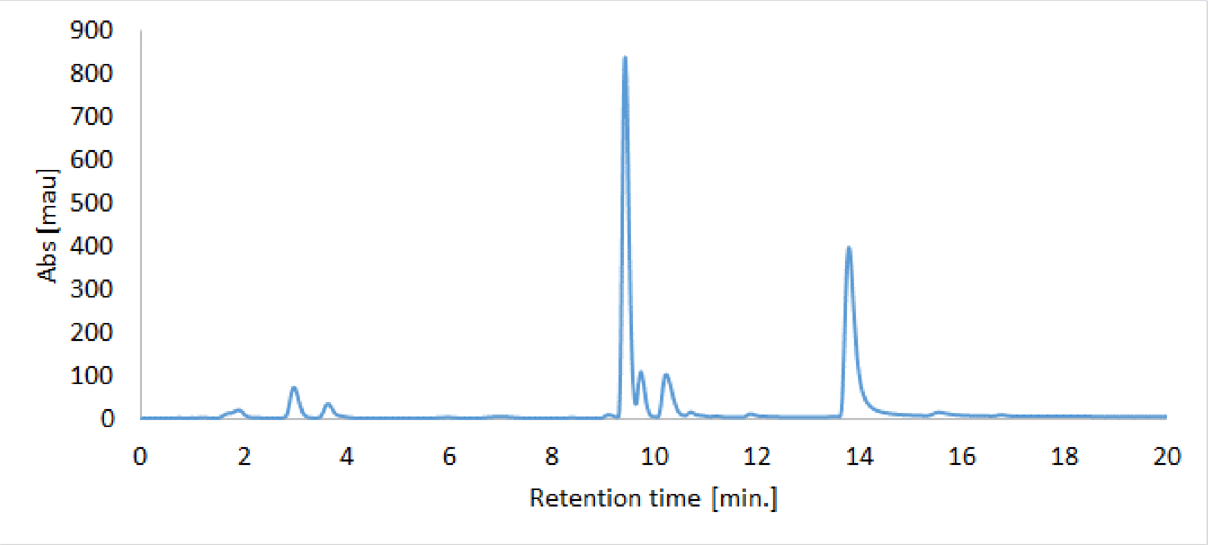

Figure S150. RP-HPLC chromatogram of DCL at pH 7 and room temperature, ***MESNa*** *as catalyst, detection at 254 nm*

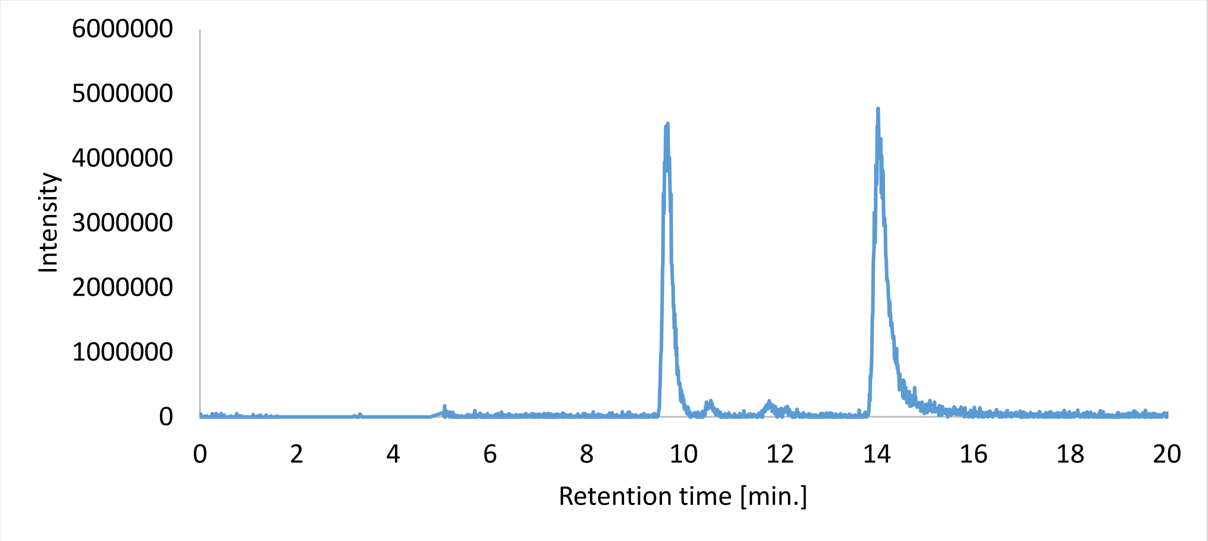

*Figure S151. Extracted ion chromatogram of DCL at pH 7 and room temperature, **MESNa** as catalyst, m/z range <348,355>*

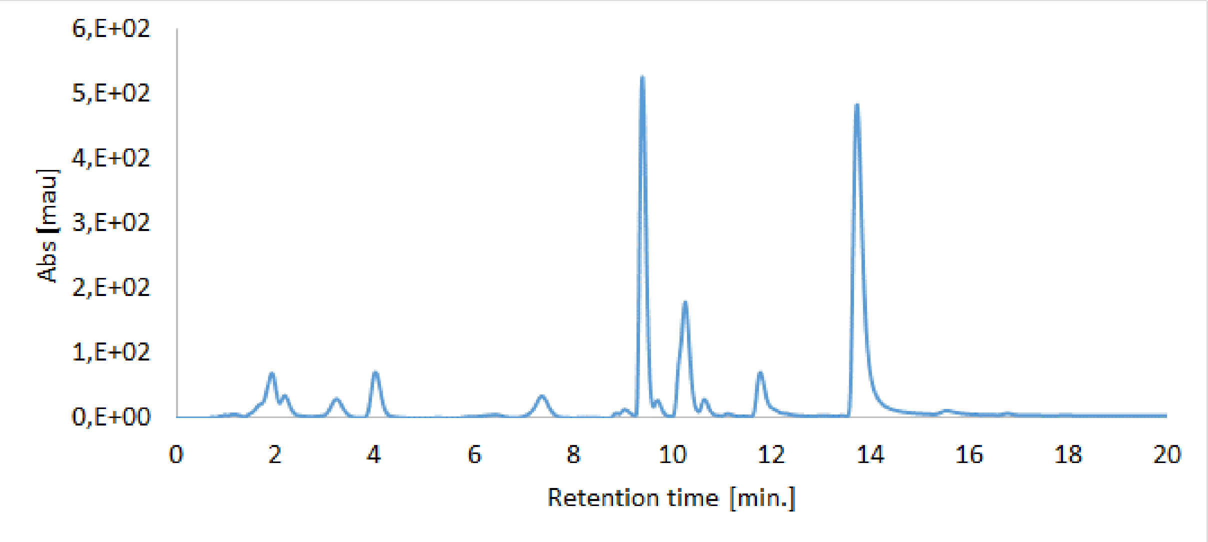

*Figure S152. RP-HPLC chromatogram of DCL at pH 7 and 40°C, **MESNa** as catalyst, detection at 254 nm*

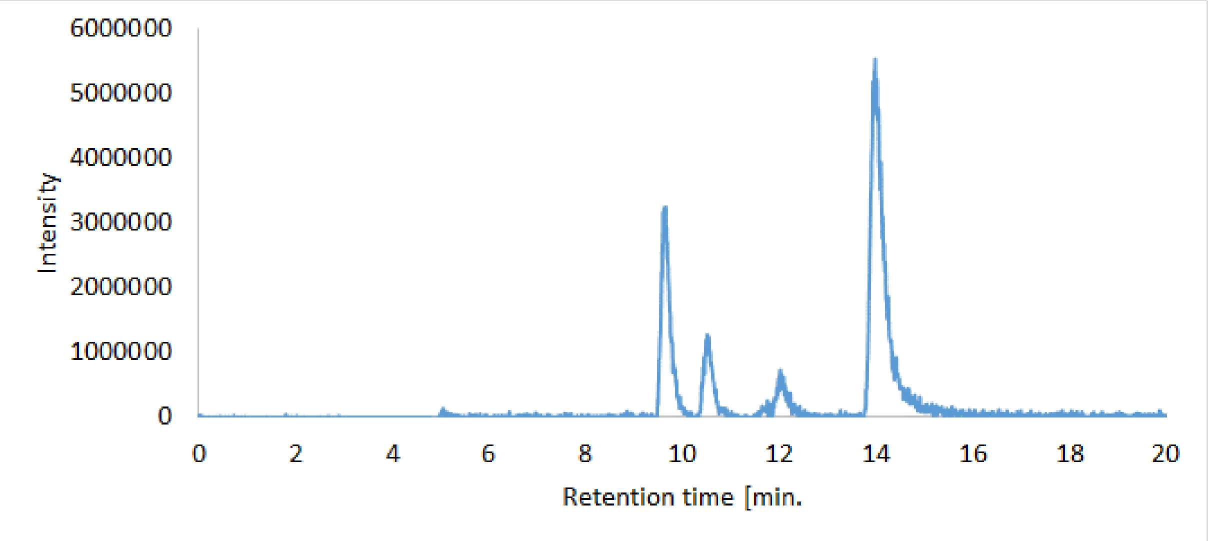

*Figure S153. Extracted ion chromatogram of DCL at pH 7 and 40°C, **MESNa** as catalyst, m/z range <348,355>*

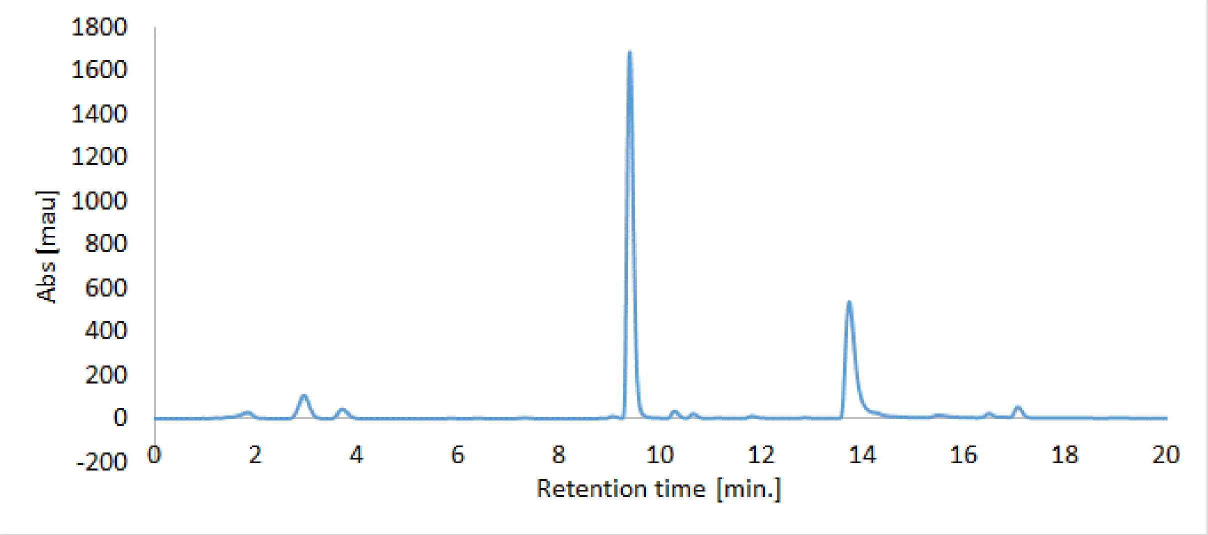

*Figure S154. RP-HPLC chromatogram of DCL at pH 7 and room temperature, **MPAA** as catalyst, detection at 254 nm*

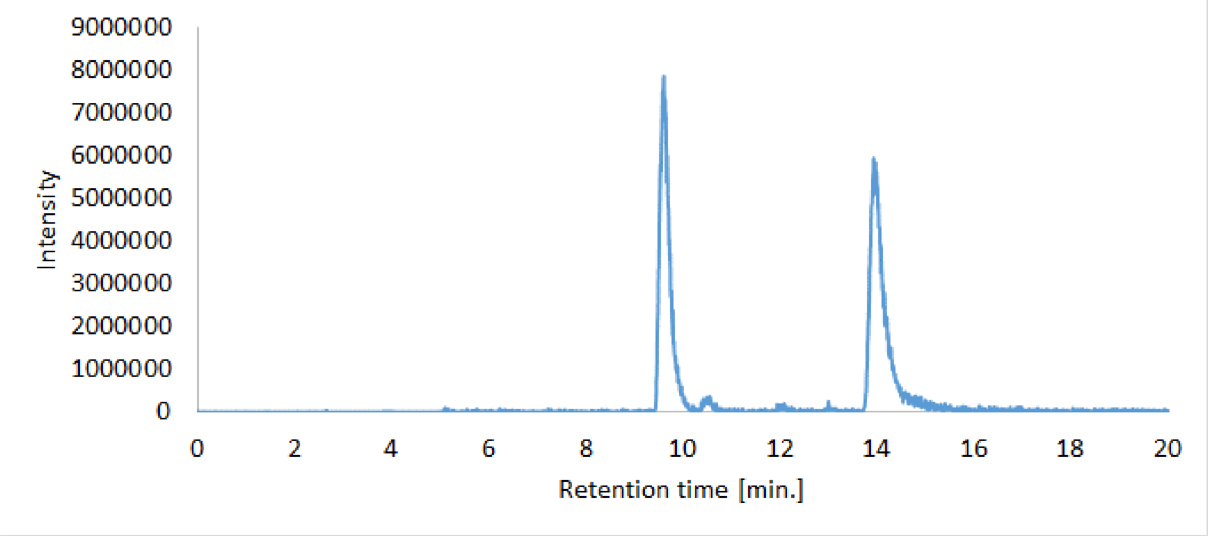

*Figure S155. Extracted ion chromatogram of DCL at pH 7 and room temperature, **MPAA** as catalyst, m/z range <348,355>*

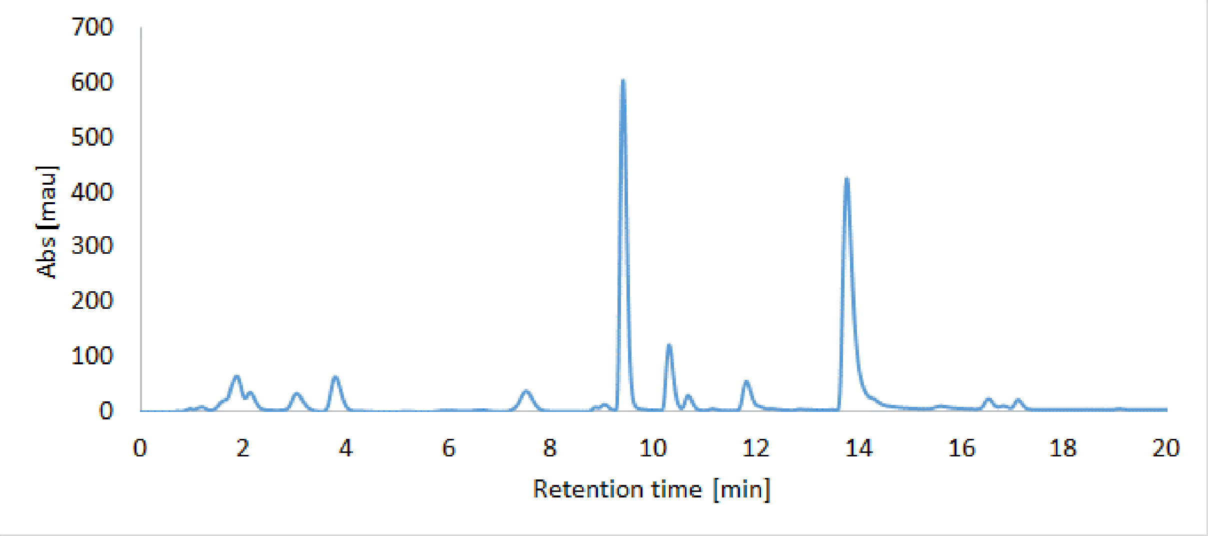

*Figure S156. RP-HPLC chromatogram of DCL at pH 7 and 40°C, **MPAA** as catalyst, detection at 254 nm*

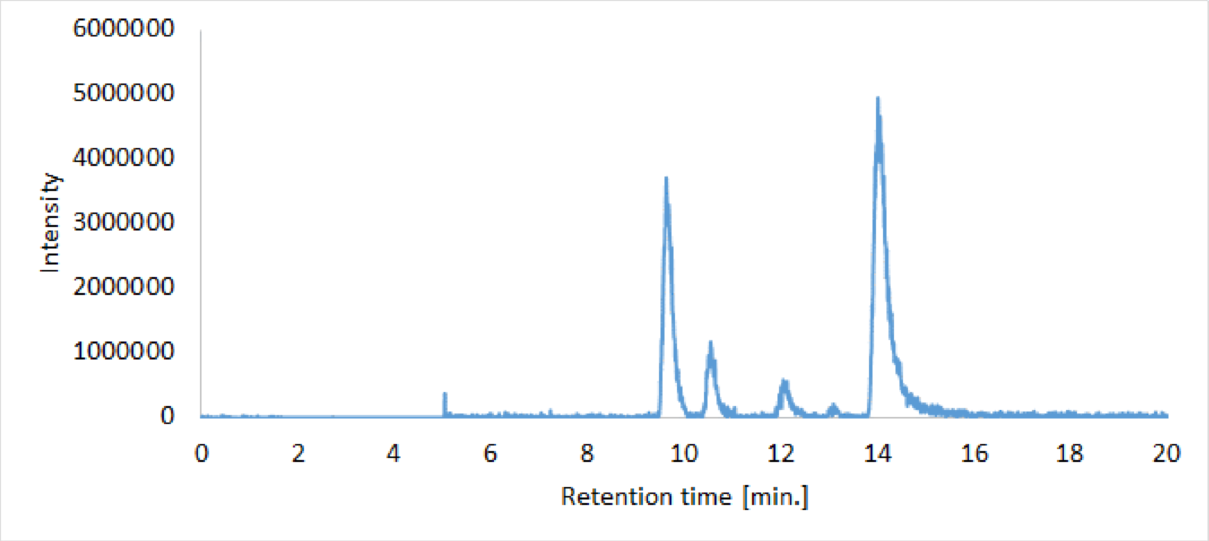

*Figure S157. Extracted ion chromatogram of DCL at pH 7 and 40°C, **MPAA** as catalyst, m/z range <348,355>*

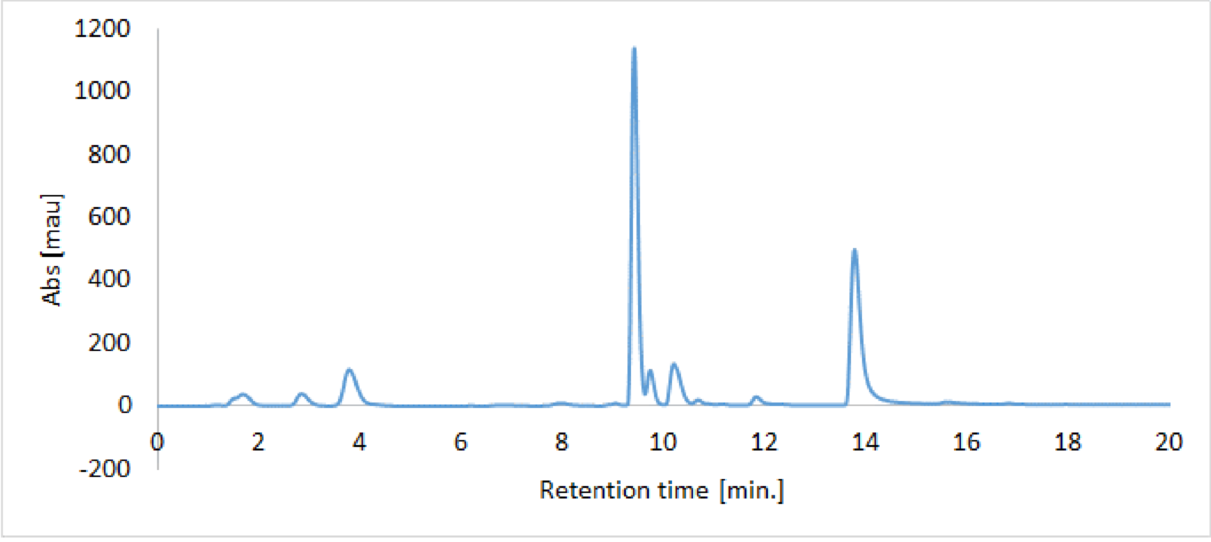

*Figure S158. RP-HPLC chromatogram of DCL at pH 9 and room temperature, **MESNa** as catalyst, detection at 254 nm*

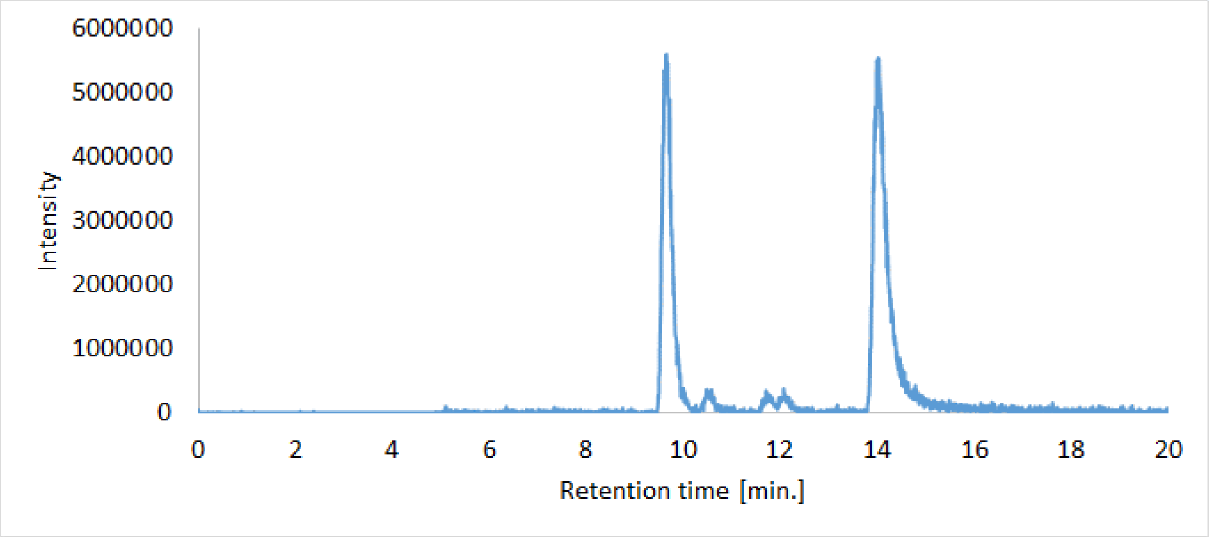

*Figure S159. Extracted ion chromatogram of DCL at pH 9 and room temperature, **MESNa** as catalyst, m/z range <348,355>*

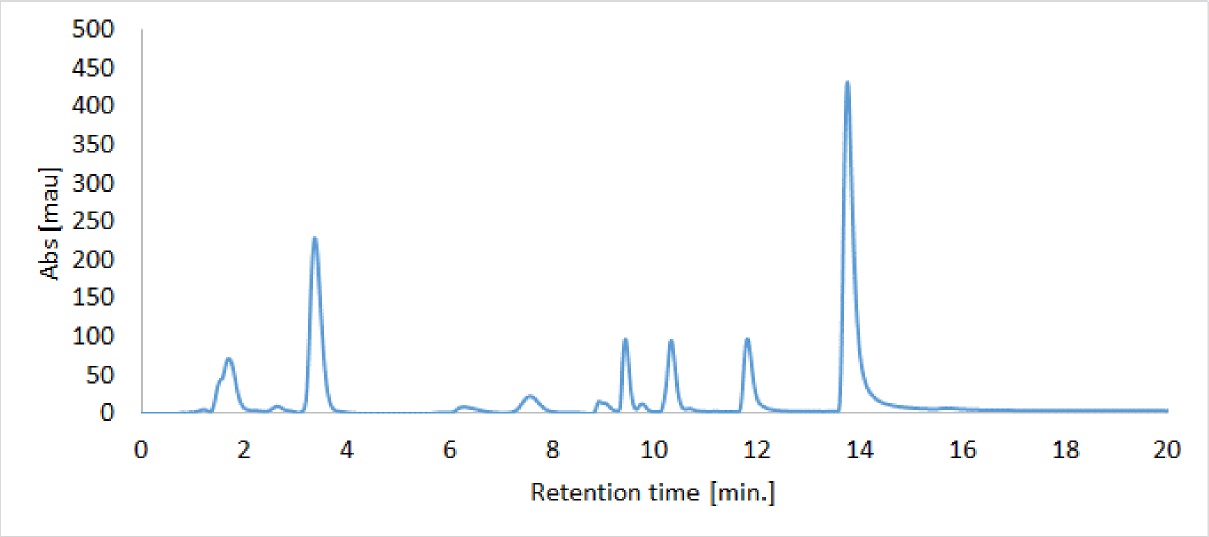

*Figure S160. RP-HPLC chromatogram of DCL at pH 9 and 40°C, **MESNa** as catalyst, detection at 254 nm*

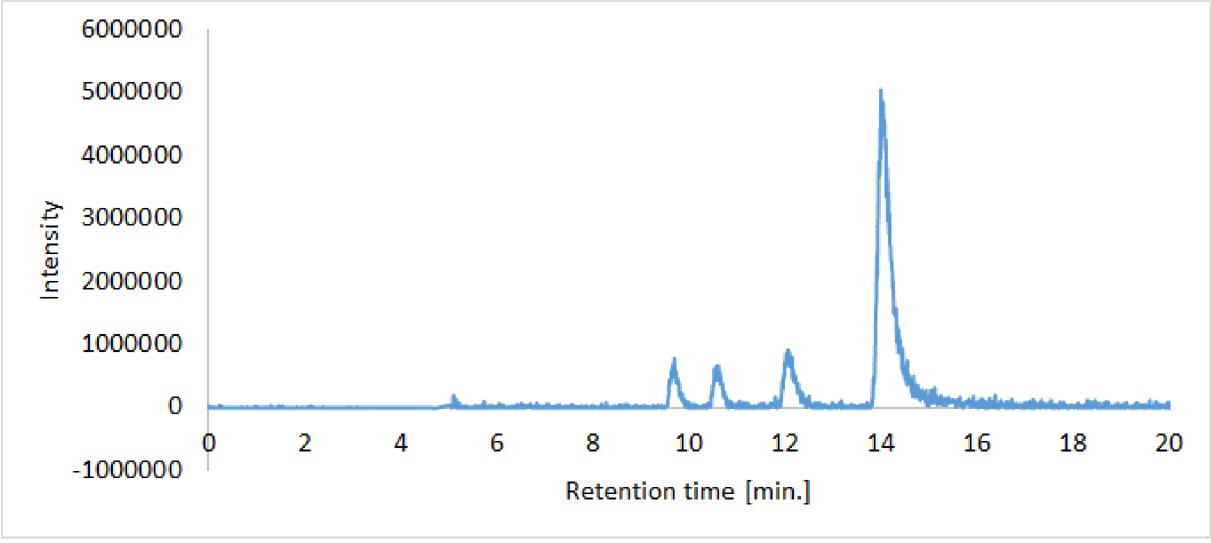

*Figure S161. Extracted ion chromatogram of DCL at pH 9 and 40°C, **MESNa** as catalyst, m/z range <348,355>*

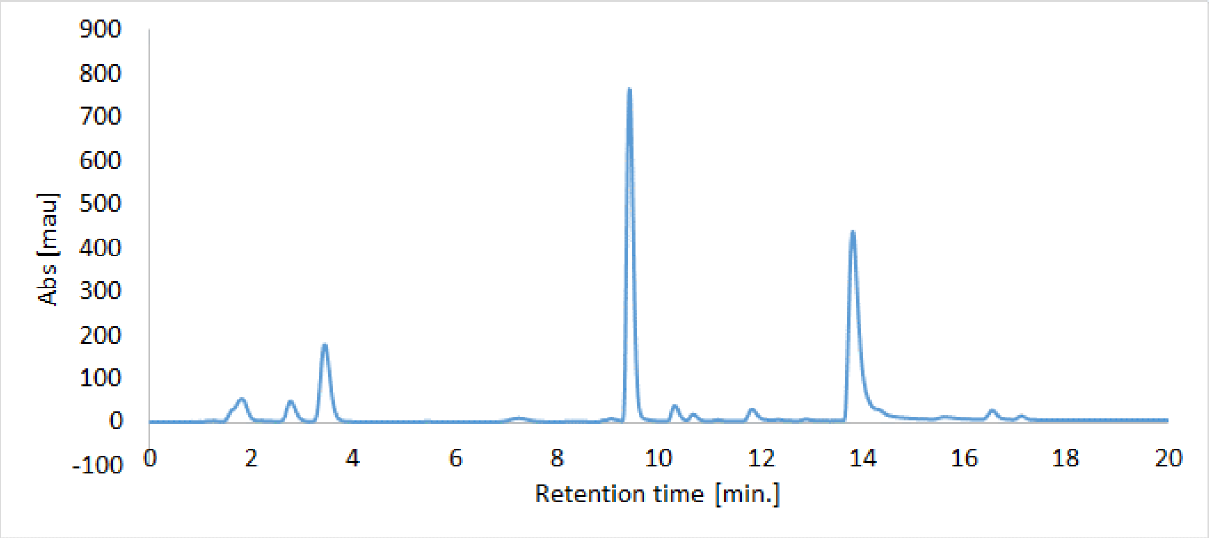

*Figure S162. RP-HPLC chromatogram of DCL at pH 9 and room temperature, **MPAA** as catalyst, detection at 254 nm*

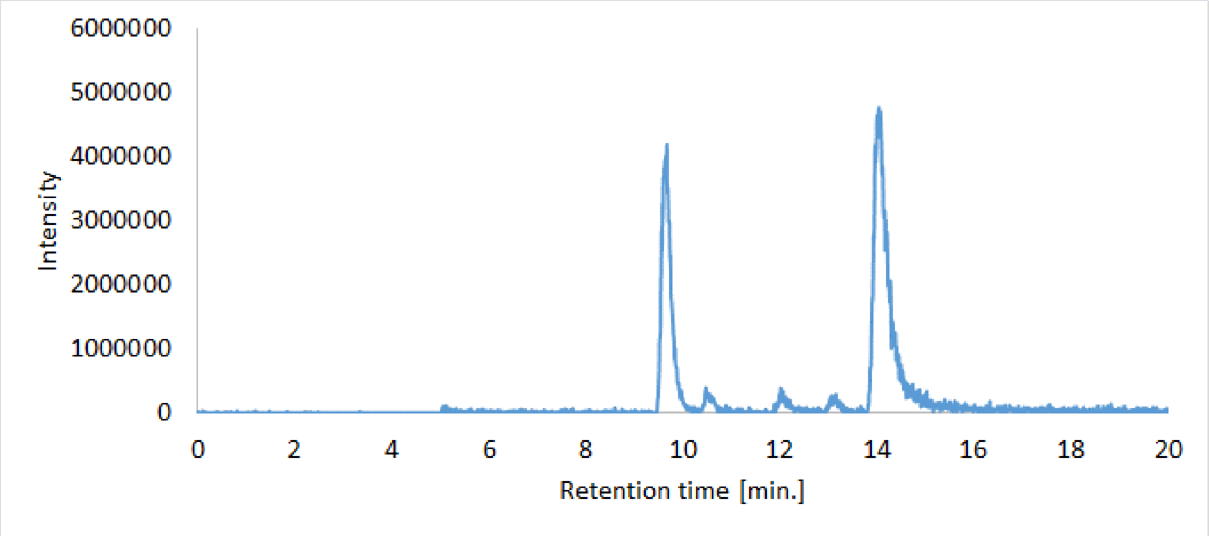

*Figure S163. Extracted ion chromatogram of DCL at pH 9 and room temperature, **MPAA** as catalyst, m/z range <348,355>*

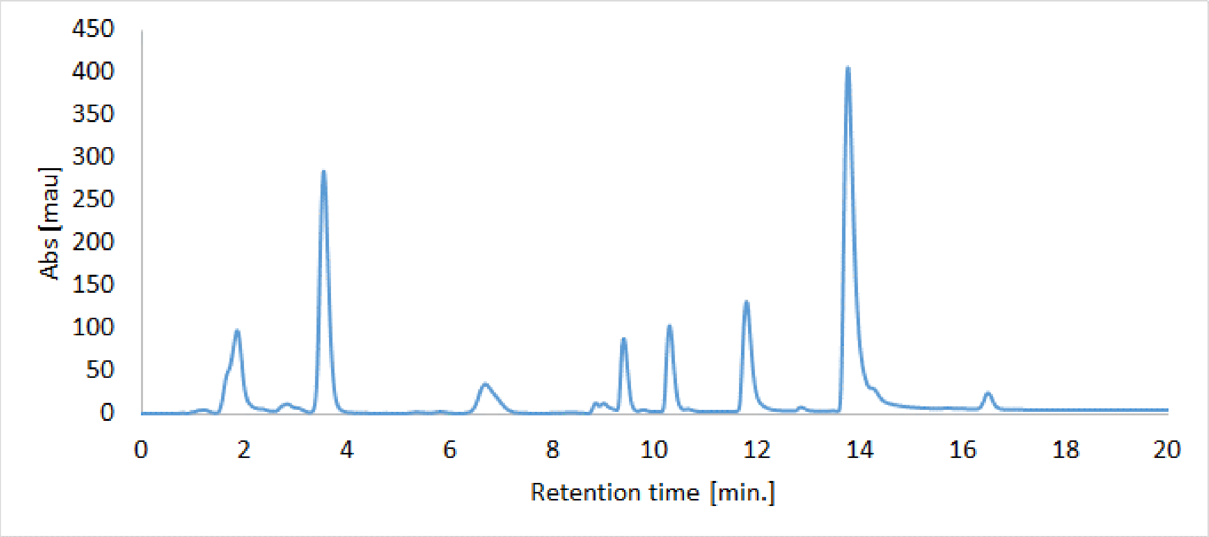

*Figure S164. RP-HPLC chromatogram of DCL at pH 9 and 40°C, **MPAA** as catalyst, detection at 254 nm*

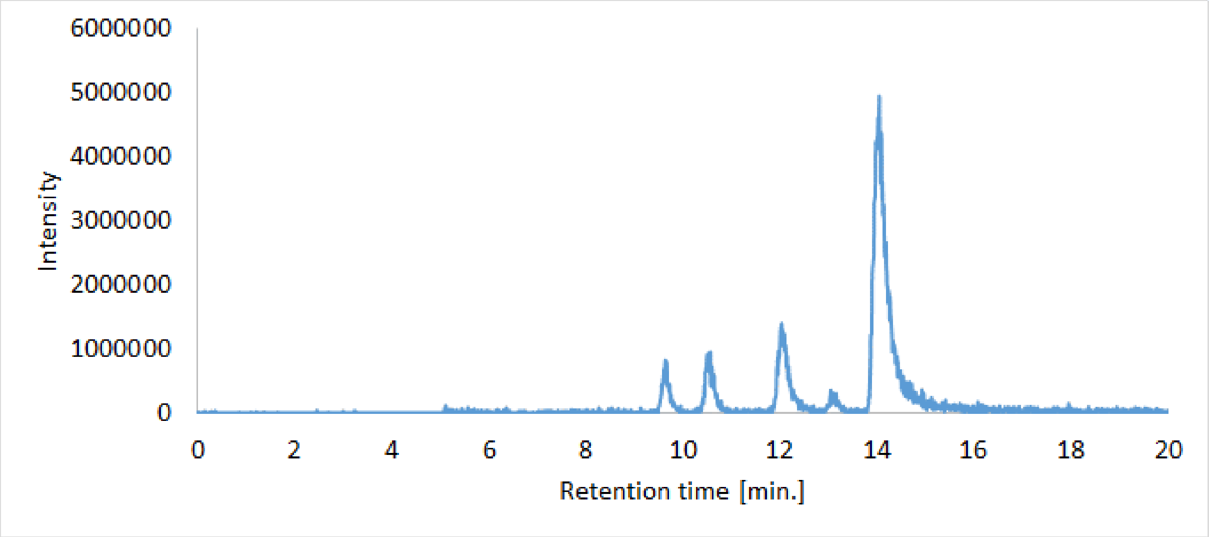

*Figure S165. Extracted ion chromatogram of DCL at pH 9 and 40°C, **MPAA** as catalyst, m/z range <348,355>*

Extracted ESI-MS(+) spectra of the main components:

All MS spectra presented in this section have been extracted from LC-MS chromatograms.

Compound 1: TMT(AcNH$_2$)$_3$

Retention time: 9.4 min

HRMS (ESI-MS) m/z: [M-NH$_3$+H]$^+$ Calcd for $C_9H_{10}N_5O_3S_3$ 331.9946; Found 331.9997, [M+H]$^+$ Calcd for $C_9H_{13}N_6O_3S_3$ 349.0206; Found 349.0118, [M+Na]$^+$ Calcd for $C_9H_{13}N_6O_3S_3Na$ 371.0025; Found 371.0005, [M+K]$^+$ Calcd for $C_9H_{13}N_6O_3S_3K$ 386.9765; Found 386.9683, [2M+Na]$^+$ Calcd for $C_{18}H_{24}N_{12}O_6S_6Na$ 719.0158; Found 718.9998

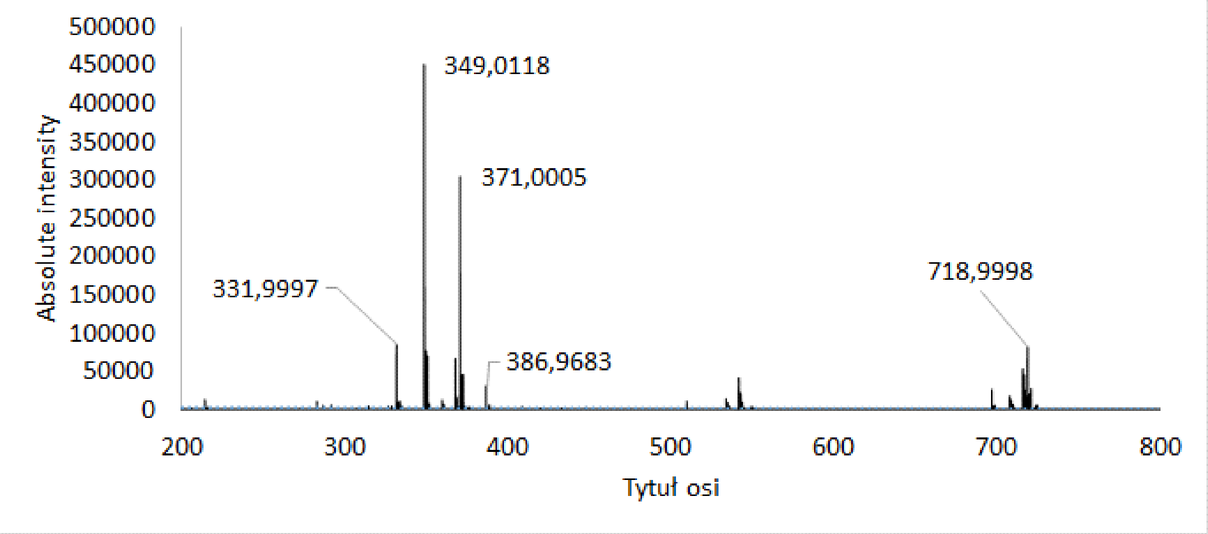

Figure S166. Extracted ESI-MS spectrum in positive ion mode of TMT(AcNH$_2$)$_3$

Compound 2: TMT(AcNH$_2$)$_2$(AcOH)

Retention time: 10.3 min

HRMS (ESI-MS) mz/: [M-NH$_3$+H]$^+$ Calcd for $C_9H_9N_4O_4S_3$ 332.9786; Found 332.9696, [M+H]$^+$ Calcd for $C_9H_{12}N_5O_4S_3$ 350.0046; Found 350.0062, [M+Na]$^+$ Calcd for $C_9H_{12}N_5O_4S_3Na$ 371.9865; Found 371.9897, [M+K]$^+$ Calcd for $C_9H_{12}N_5O_4S_3K$ 387.9605; Found 387.9603, [2M+Na]$^+$ Calcd for $C_{18}H_{24}N_{10}O_8S_6Na$ 720.9838; Found 720.9780

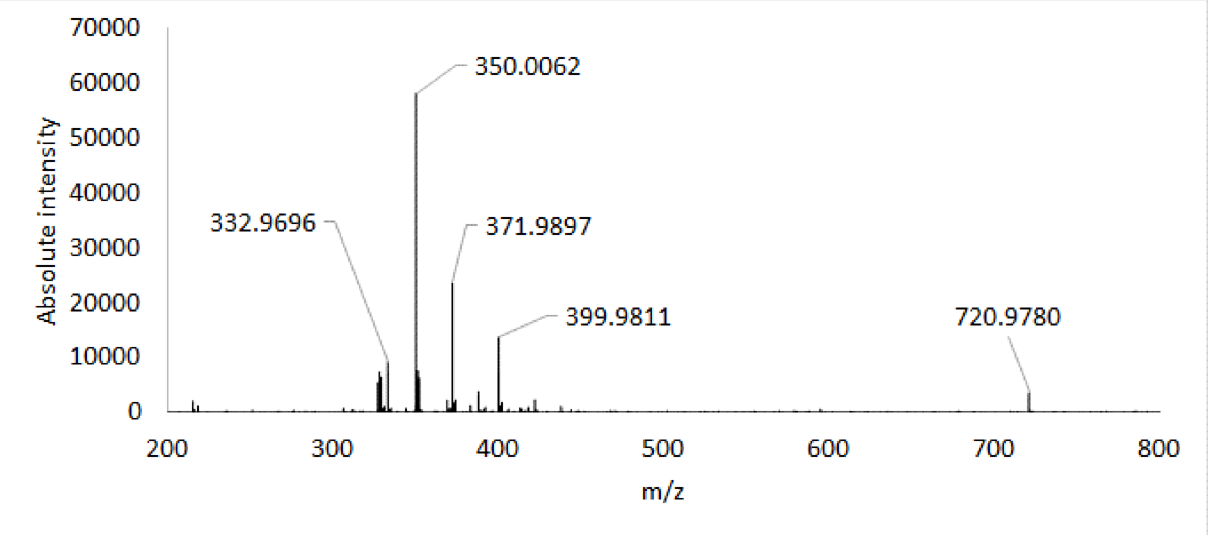

Figure S167. Extracted ESI-MS specrum in positive ion mode of TMT(AcNH$_2$)$_2$(AcOH)

Compound 3: $TMT(AcNH_2)(AcOH)_2$

Retention time: 11.8 min

HRMS (ESI-MS) m/z: $[M\text{-}NH_2\text{+}H]^+$ Calcd for $C_9H_8N_3O_5S_3$ 333.9626; Found 333.9580, $[M+H]^+$ Calcd for $C_9H_{11}N_4O_5S_3$ 350.9886; Found 350.9846, $[M+Na]^+$ Calcd for $C_9H_{10}N_4O_5S_3Na$ 372.9705; Found 372.9623, $[M+K]^+$ Calcd for $C_9H_{10}N_4O_5S_3K$ 388.9445; Found 388.9351, $[2M+Na]^+$ Calcd for $C_{18}H_{20}N_8O_{10}S_6Na$ 722.9519; Found 722.9338

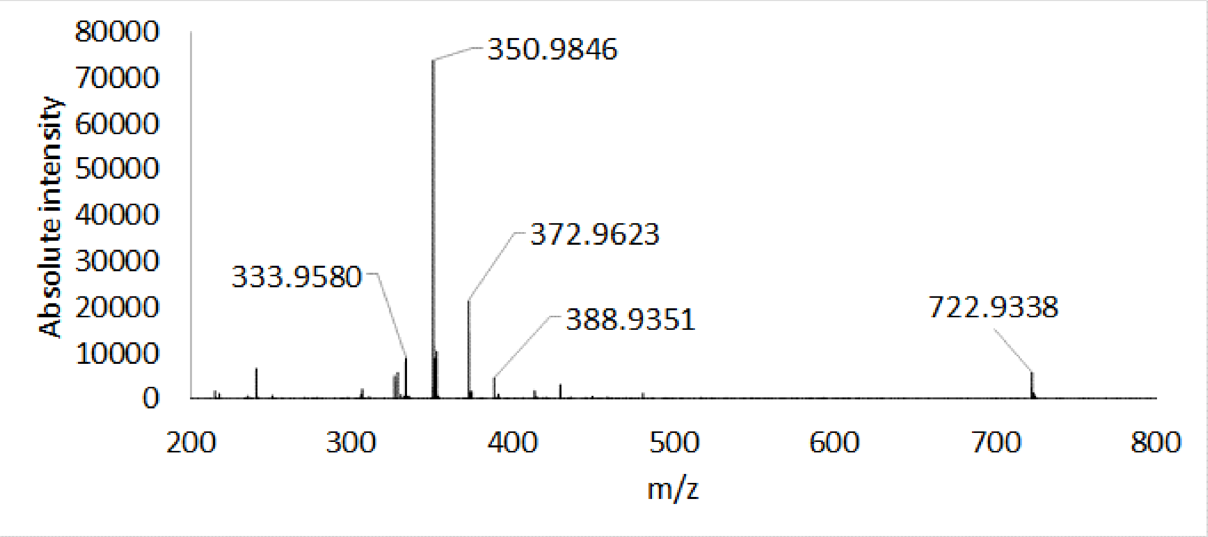

Figure S168. Extracted ESI-MS spectrum in positive ion mode of $TMT(AcNH_2)(AcOH)_2$

Compound 2: $TMT(AcOH)_3$

Retention time: 13.8 min

HRMS (ESI-MS) m/z: $[M\text{-}H_2O\text{+}H]^+$ Calcd for $C_9H_8N_3O_5S_3$ 333.9626; Found 333.9580, $[M+H]^+$ Calcd for $C_9H_{10}N_3O_6S_3$ 351.9726; Found 351.9643, $[M+Na]^+$ Calcd for $C_9H_9N_3O_6S_3Na$ 373.9546; Found 373.9541, $[M+K]^+$ Calcd for $C_9H_9N_3O_6S_3K$ 389.9285; Found 389.9295, $[2M+Na]^+$ Calcd for $C_{18}H_{18}N_6O_{12}S_6Na$ 724.9199; Found 724.9174

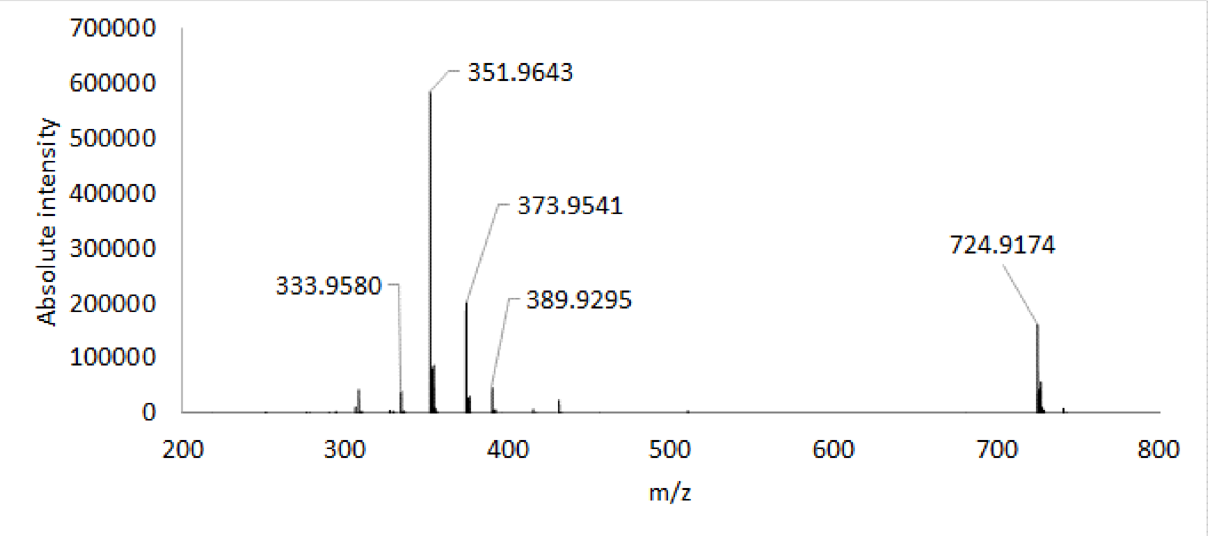

Figure S169. Extracted ESI-MS spectrum of $TMT(AcOH)_3$

6.2. Influence of the excess of a catalyst

General procedure: 18 mg of TMT(AcOH)$_3$ (0.05 mmol) and 18 mg of TMT(AcNH$_2$)$_3$ (0.05 mmol) were mixed and dissolved in 10 mL of 10% acetic acid, obtaining the stock solution with both components in 5mM concentration. The stock solution was divided into 1 mL aliquots in glass vials and freeze-dried samples were dissolved in 1 mL of an appropriate 0.5 M phosphate buffer at pH 7 or 9, containing 50 mM MESNa and 20 mM TCEP. The samples were incubated for 24 hours stirring on a rotary stirrer at room temperature or in a laboratory oven at 40°C. Samples for the HPLC analysis were prepared by 10-fold dilution with water.

RP-HPLC with UV detection:

Before incubation, detection at 254 nm:

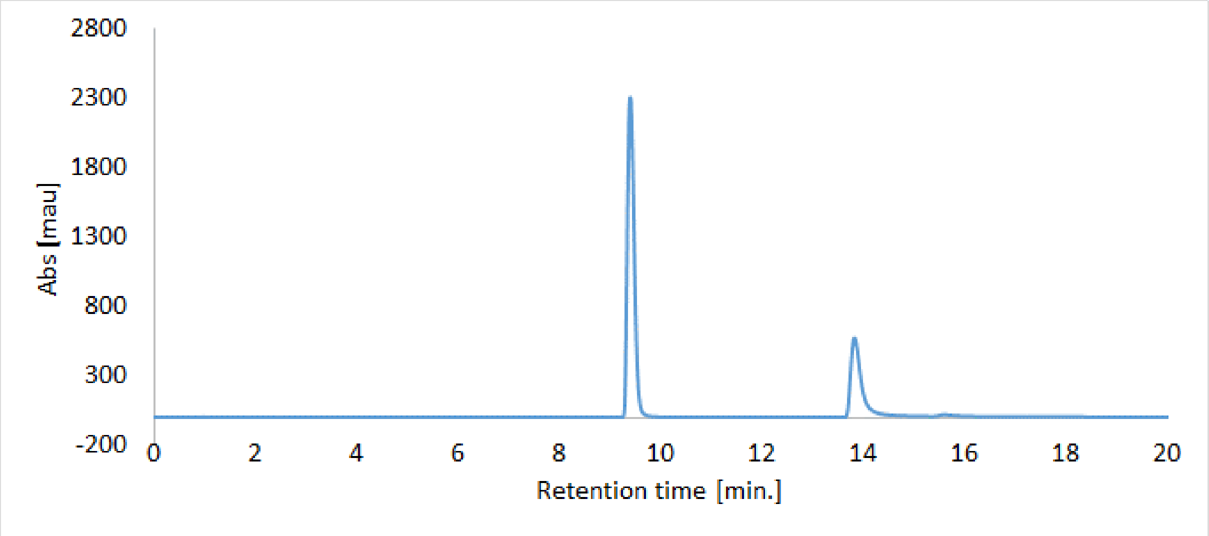

Figure S170. RP-HPLC chromatogram of starting composition of the dynamic combinatorial library

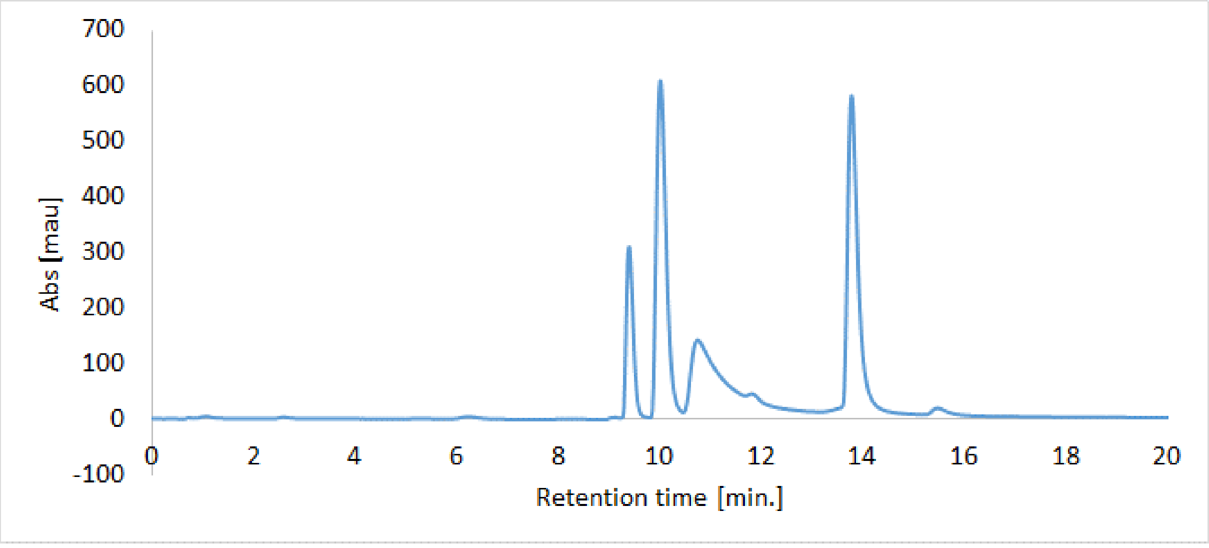

Figure S171. RP-HPLC chromatogram of DCL at pH 7 and room temperature, catalyzed by 10-fold excess of MESNa, detection at 254 nm

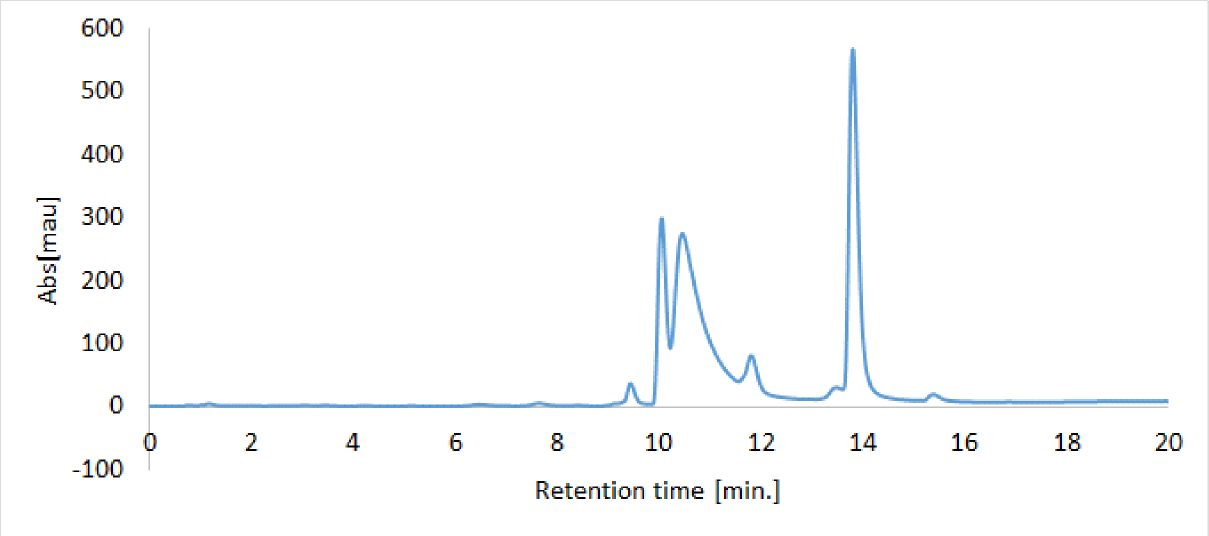

Figure S172. RP-HPLC chromatogram of DCL at pH 7 and 40°C, catalyzed by 10-fold excess of MESNa, detection at 254 nm

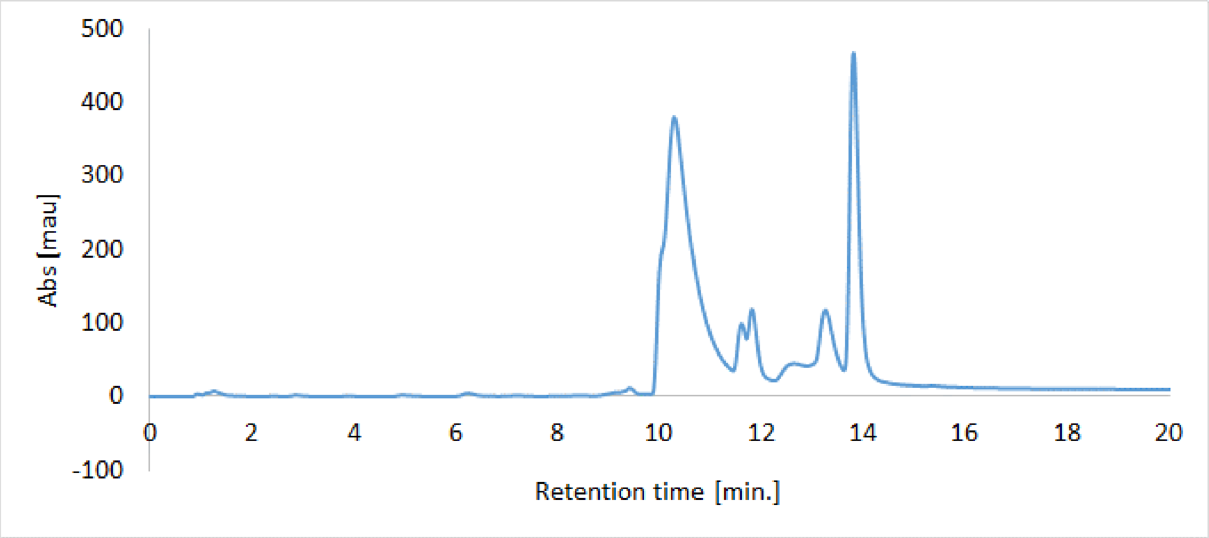

Figure S173. RP-HPLC chromatogram of DCL at pH 9 and room temperature, catalyzed by 10-fold excess of MESNa, detection at 254 nm

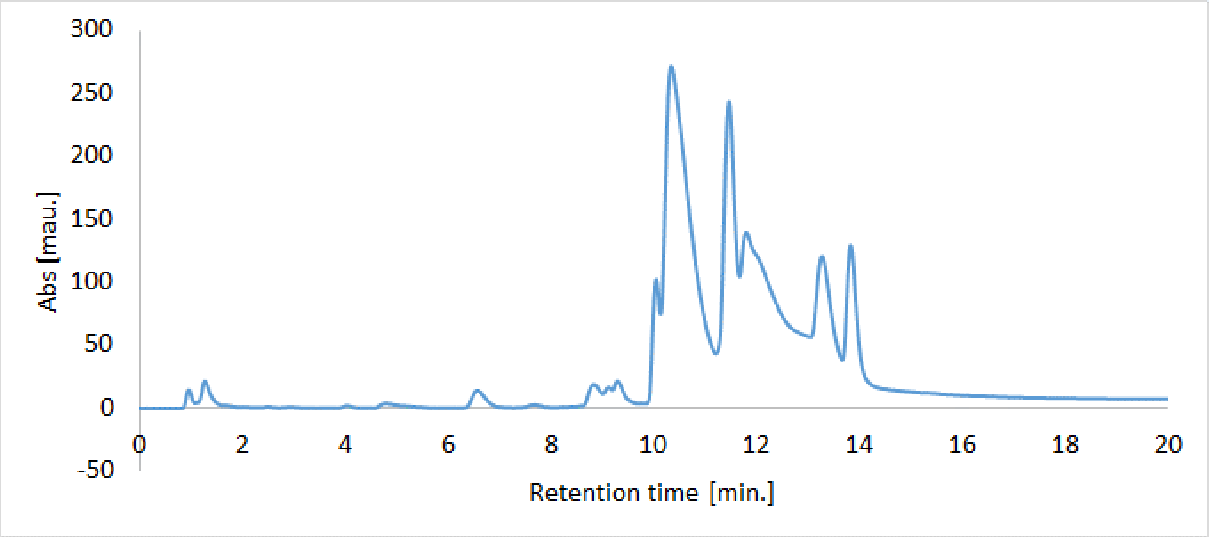

Figure S174. RP-HPLC chromatogram of DCL at pH 9 and 40°C, catalyzed by 10-fold excess of MESNa, detection at 254 nm

X = OH or NH 2

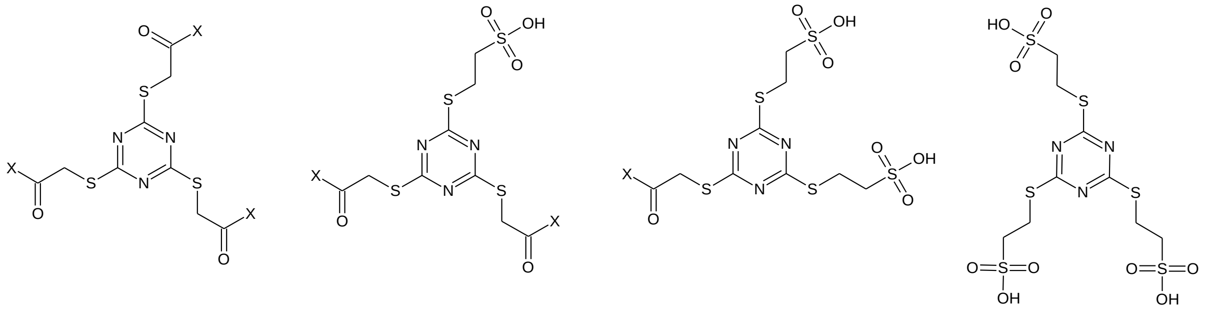

TMT(AcX)$_3$ XIC, m/z <348,355>

TMT(AcX)$_2$(MES) XIC, m/z <399,405>

TMT(AcX)(MES)$_2$ XIC, m/z <449,455>

TMT(MES)$_3$ XIC, m/z <501,505>

Figure S175. Extraction m/z ranges for [M+H]⁺ ions of analyzed DCLs components (us

pH 7, room temperature, detection at 254 nm:

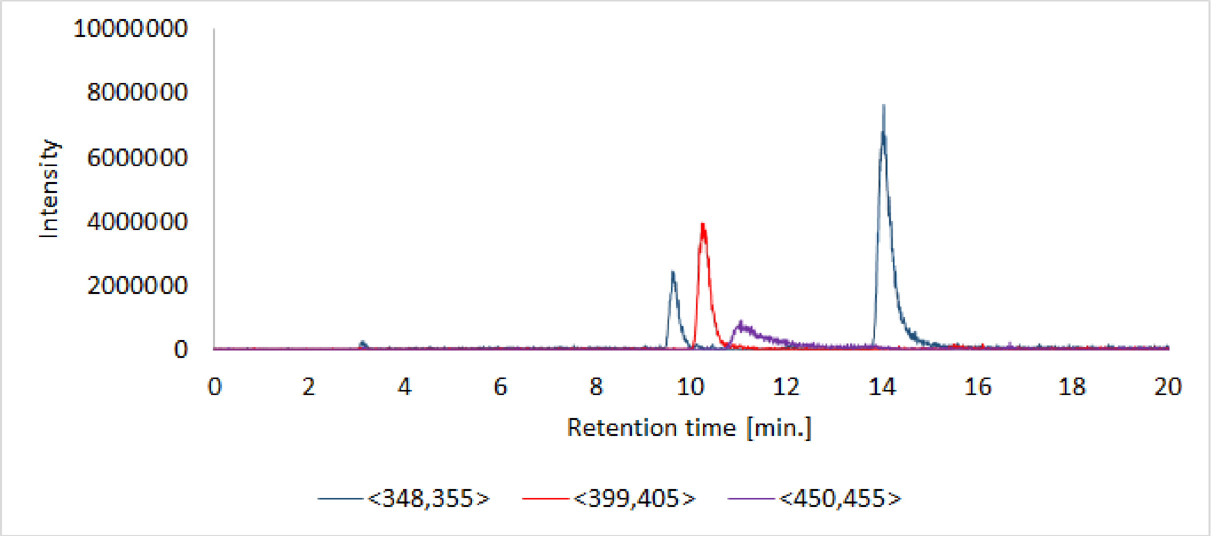

Figure S176. Extracted ion chromatograms of the DCL including MESNa containing products at pH 7 and room temperature

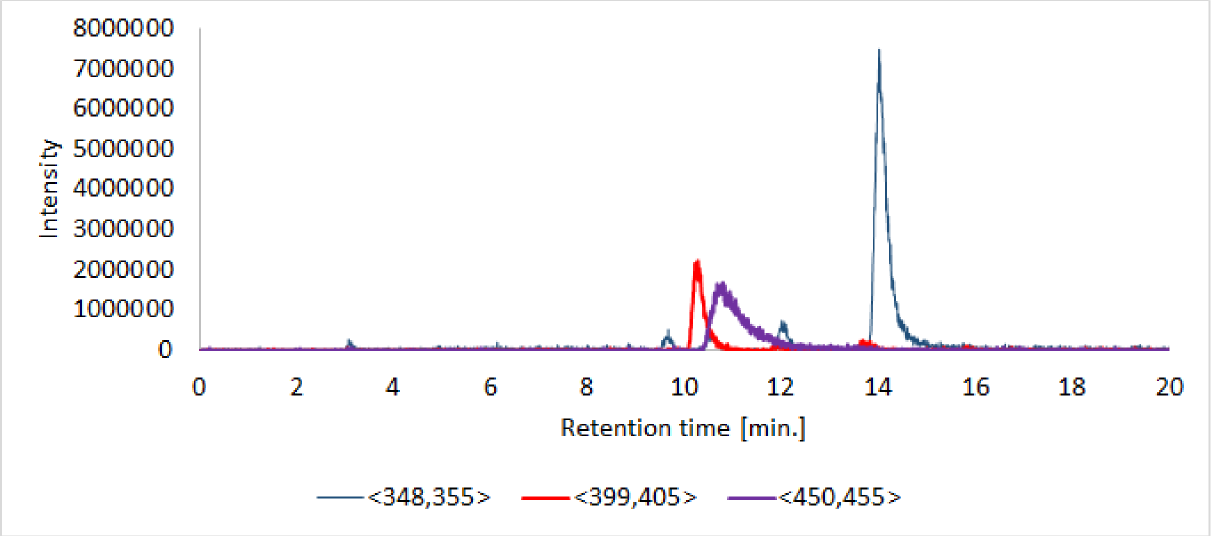

Figure S177. Extracted ion chromatograms of the DCL including MESNa containing products at pH 7 and 40°C

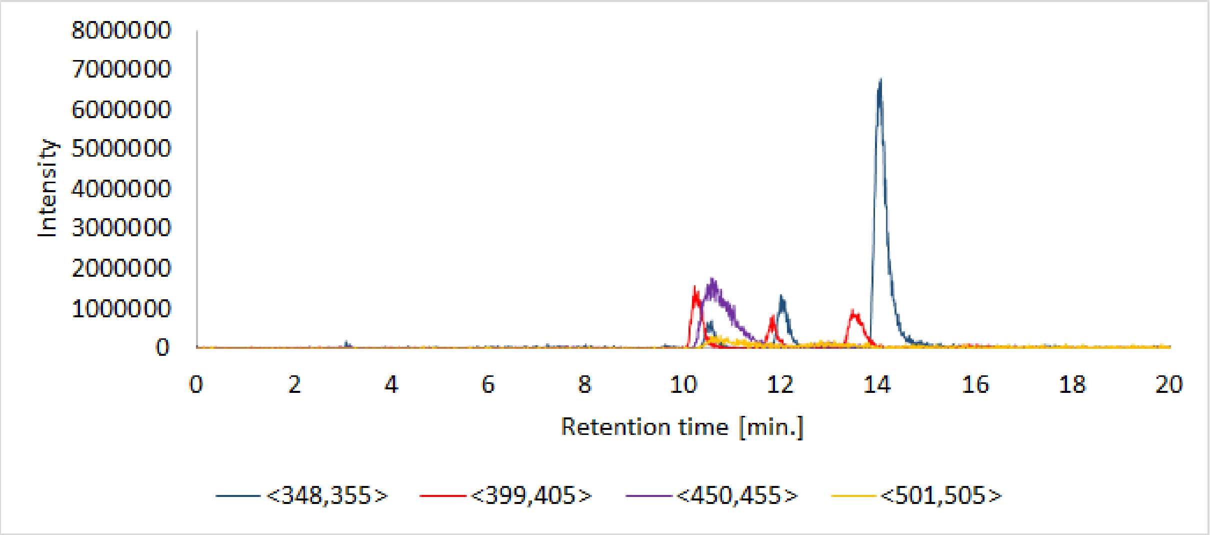

Figure S178. Extracted ion chromatograms of the DCL including MESNa containing products at pH 9 and room temperature

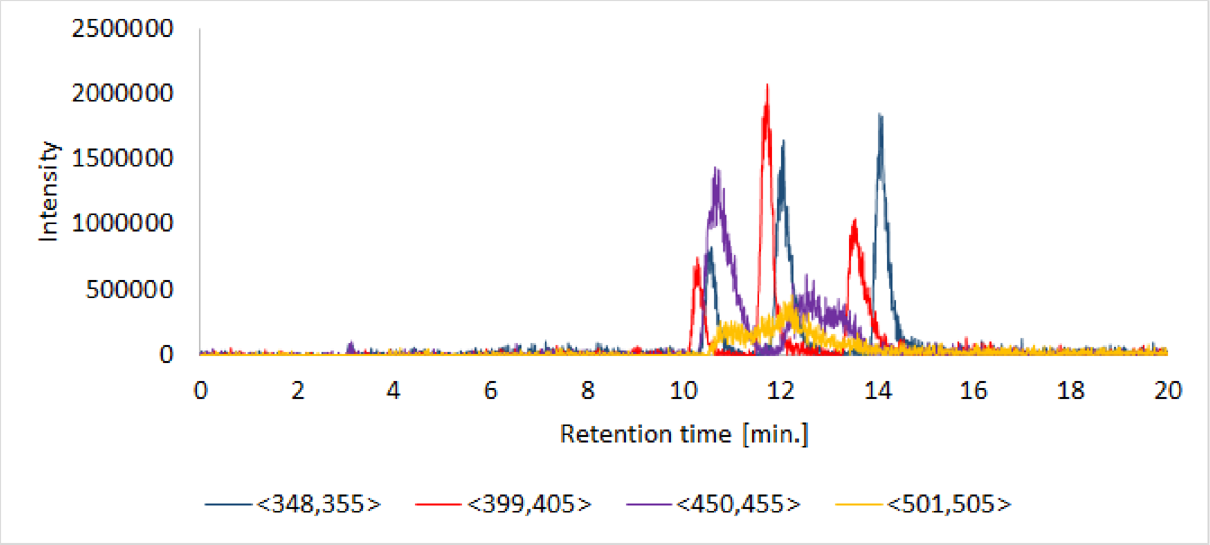

Figure S179. Extracted ion chromatograms of the DCL including MESNa containing products at pH 9 and 40°C.

Extracted ESI-MS(+) spectra of MES-containing compounds:

TMT(AcNH$_2$)$_2$(MES):

Retention time: 10.3 min

HRMS (ESI-MS) m/z: $[M-NH_2+H]^+$ Calcd for $C_9H_{11}N_4O_5S_4$ 382.9612; Found 382.9585, $[M+H]^+$ Calcd for $C_9H_{14}N_5O_5S_4$ 399.9872; Found 399.9811, $[M+Na]^+$ Calcd for $C_9H_{13}N_5O_5S_4Na$ 421.9692; Found 421.9618

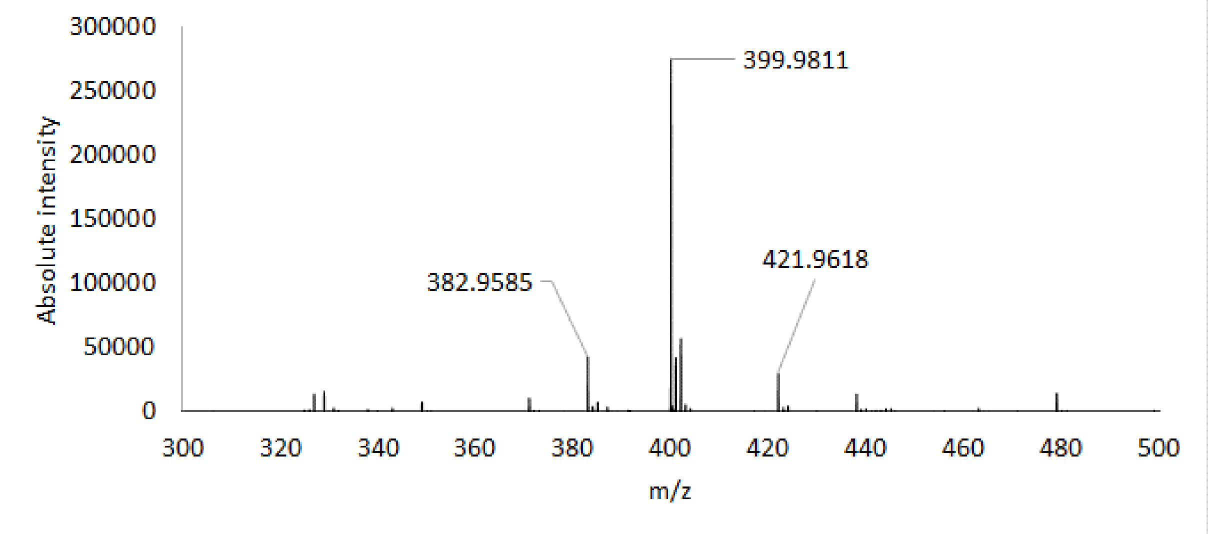

Figure S180. Extracted ESI-MS spectrum in positive ion mode of TMT(AcNH$_2$)$_2$(MES)

TMT(AcNH$_2$)(AcOH)(MES):

Retention time: 11.7 min

MS(+): $[M-NH_2+H]^+$ Calcd for $C_9H_{10}N_3O_6S_4$ 383.9452; Found 383.9453, $[M+H]^+$ Calcd for $C_9H_{13}N_4O_6S_4$ 400.9712; Found 400.9709, $[M+Na]^+$ Calcd for $C_9H_{12}N_4O_6S_4Na$ 422.9532; Found 422.9593

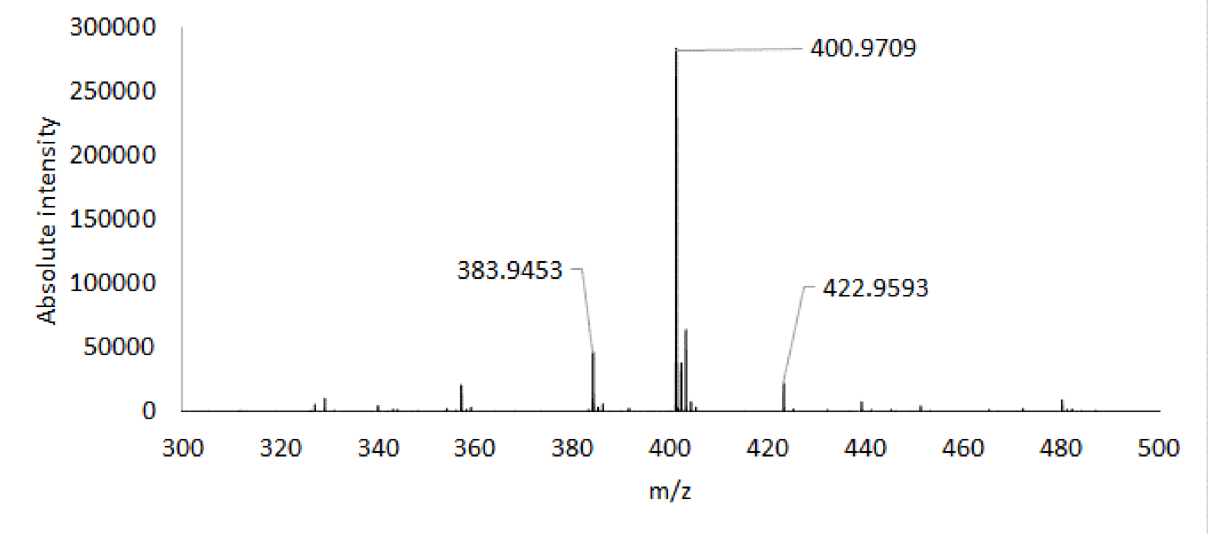

Figure S181. Extracted ESI-MS spectrum in positive ion mode of TMT(AcNH$_2$)(AcOH)(MES)

TMT(AcOH)2(MES):

Retention time: 13.5 min

HRMS (ESI-MS) m/z: $[M+H]^+$ Calcd for $C_9H_{12}N_3O_7S_4$ 401.9553; Found 401.9619, $[M+Na]^+$ Calcd for $C_9H_{12}N_3O_7S_4Na$ 423.9372; Found 423.9386

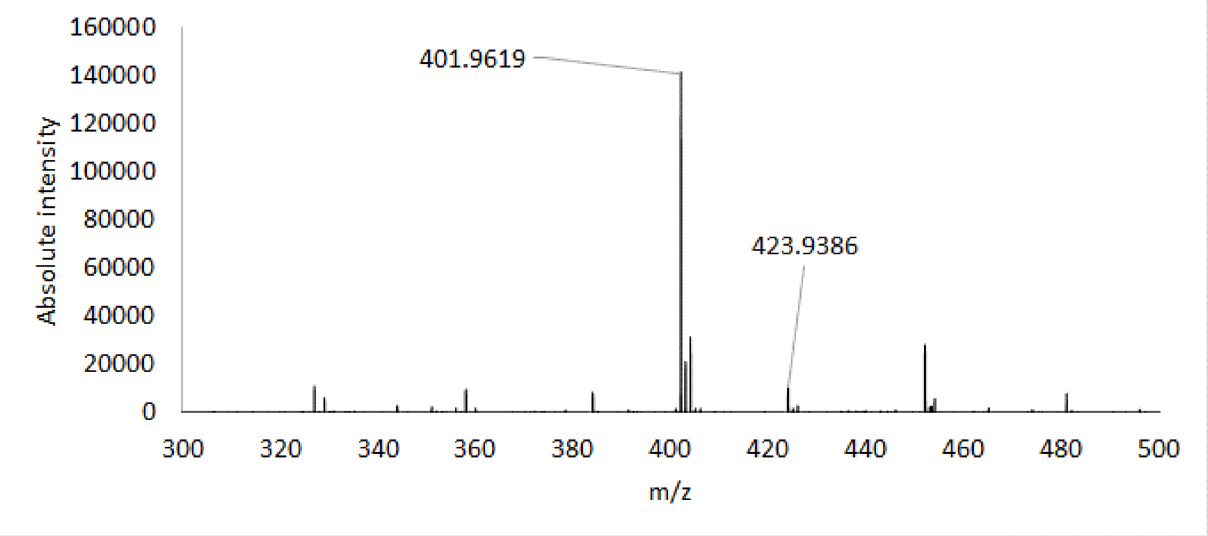

Figure S182.Extracted ESI-MS spectrum in positive ion mode of TMT(AcOH)$_2$(MES)

TMT(AcNH2)(MES)2:

Retention time: 10.6 min

HRMS (ESI-MS) m/z: $[M-NH_2+H]^+$ Calcd for $C_9H_{12}N_3O_7S_5$ 433.9279; Found 433.4305, $[M+H]^+$ Calcd for $C_9H_{15}N_4O_7S_5$ 450.9539; Found 450.9476, $[M+Na]^+$ Calcd for $C_9H_{14}N_4O_7S_5Na$ 472.9358; Found 472.9280

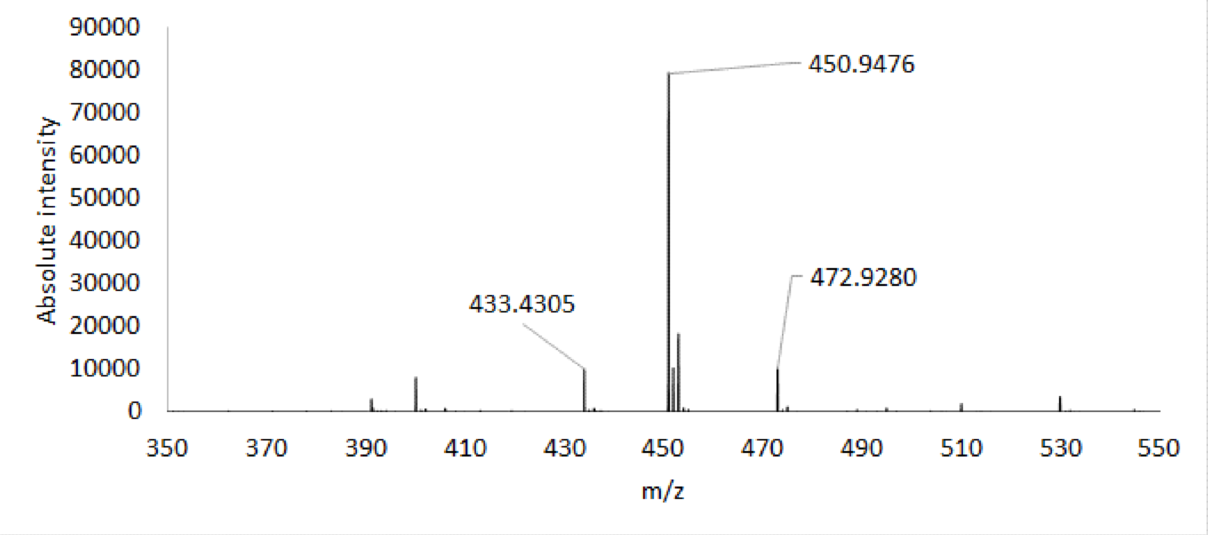

Figure S183. Extracted ESI-MS spectrum in positive ion mode of TMT(AcNH$_2$)(MES)$_2$

TMT(AcOH)(MES)$_2$:

Retention time: 12.7 min

MS(+): $[M+H]^+$ Calcd for $C_9H_{14}N_3O_8S_5$ 451.9379; Found 451.9390, $[M+Na]^+$ Calcd for $C_9H_{13}N_3O_8S_5Na$ 474.9271; Found 473.9229

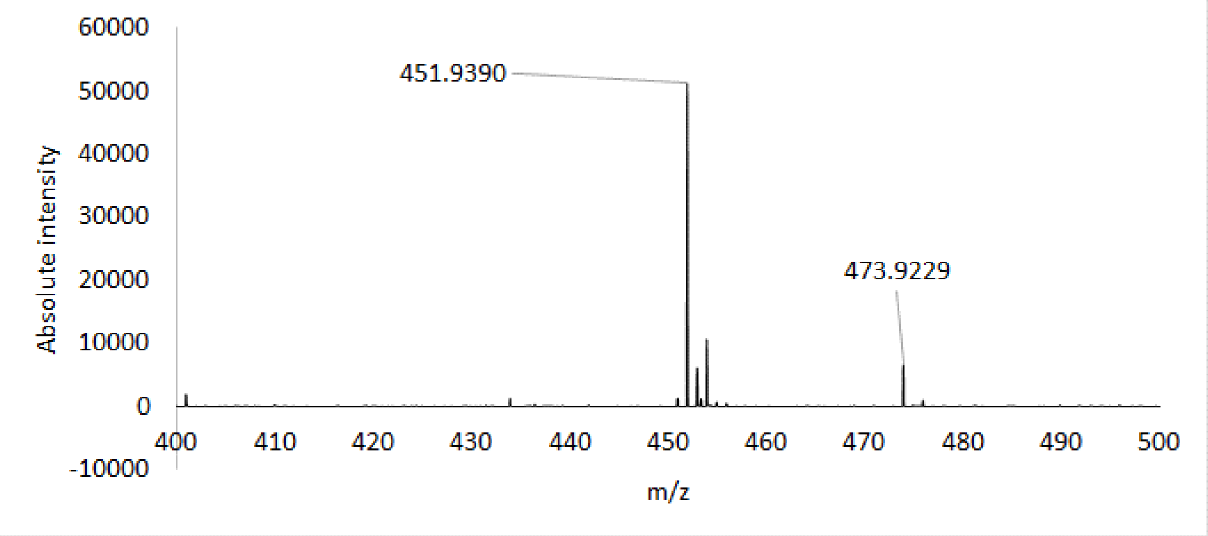

Figure S184. Extracted ESI-MS spectrum in positive ion mode of TMT(AcOH)(MES)$_2$

TMT(MES)$_3$:

Retention time: 12.0 min

MS(+): $[M+H]^+$ Calcd for $C_9H_{16}N_3O_9S_6$ 501.9205; Found 501.9123, $[M+Na]^+$ Calcd for $C_9H_{15}N_3O_9S_6Na$ 523.9097; Found 523.8905

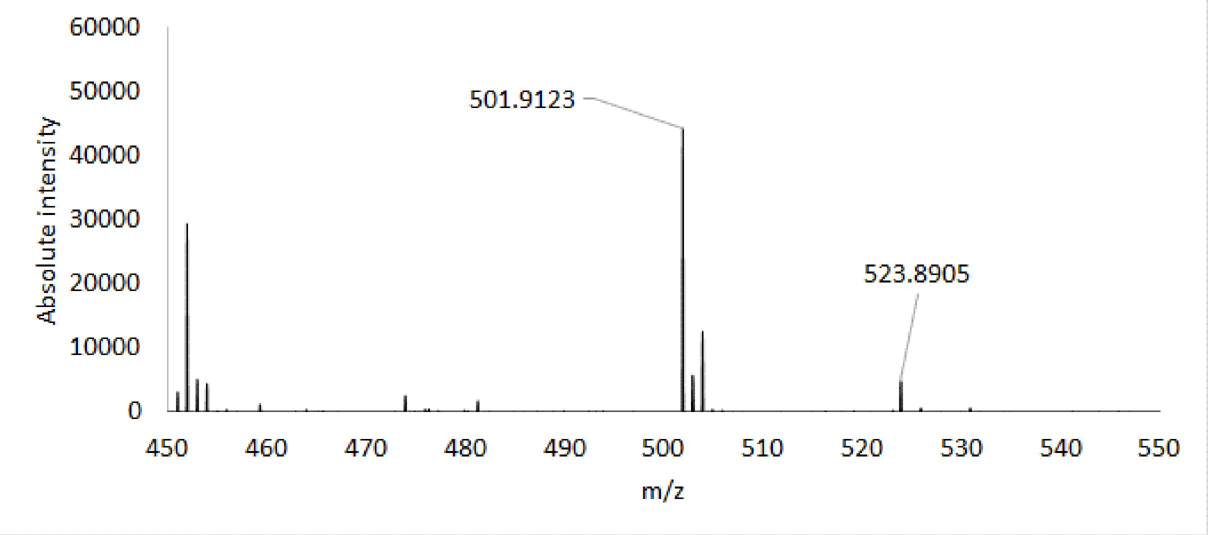

Figure S185. Extracted ESI-MS spectrum in positive ion mode of TMT(MES)$_3$

7. DFT calculations

For a better understanding of the reactivity of thiocyanurates and thiocyanuramides, we conducted a theoretical investigation using DFT calculations for model compounds:

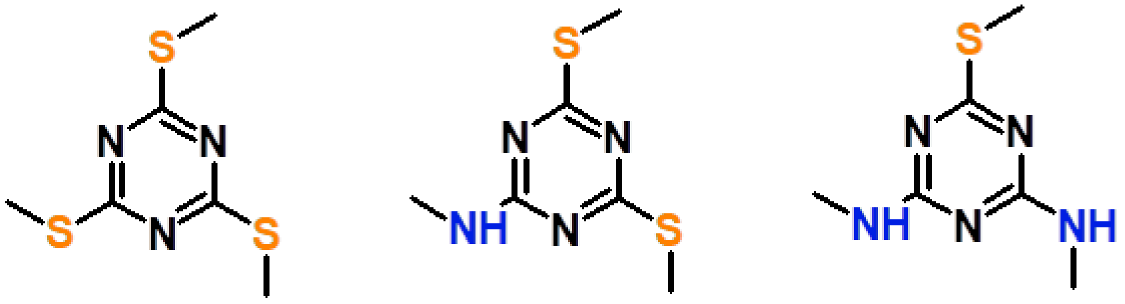

Figure S186. Models of substituted 1,3,5-triazines for DMT calculations, from left to right: TMT(Me)$_3$, DMT(Me)$_2$(methylamine), and MMT(Me)(methylamine)$_2$.

7.1. Conceptual DFT in Multiwfn

S/N	Property	TMT(Me)$_3$	DMT(Me)$_3$	MMT(Me)$_3$
1	Total Energy (N) [kcal/mol]	-999,236.1217	-784,169.2487	-569,101.0660
2	Total Energy (N+1) [kcal/mol]	-999,271.4299	-784,198.1334	-569,117.8077
3	Total Energy (N-1) [kcal/mol]	-999,076.3280	-784,015.7865	-568,950.6042
4	HOMO Energy [eV]	-8.056353	-7.930786	-7.816287
5	LUMO Energy [eV]	0.112164	0.390204	0.951324
6	Energy Gap [eV]	8.168517	8.320991	8.767610
7	Vertical IP [eV]	6.9293	6.6548	6.5246
8	2nd Vertical IP [eV]	7.1239	7.7357	7.6280
9	Electron Affinity [eV]	1.5311	1.2526	0.7260
10	Mulliken Electronegativity [eV]	4.2302	3.9537	3.6253
11	Chemical Potential [eV]	-4.2302	-3.9537	-3.6253
12	Hardness [eV]	5.3982	5.4022	5.7987
13	Softness [eV^-1]	0.1852	0.1851	0.1725
14	Electrophilicity Index [eV]	1.6575	1.4468	1.1333
15	Nucleophilicity Index [eV]	1.0648	1.1904	1.3049
16	Cubic Electrophilicity Index [eV]	1.6765	1.5554	1.3316
17	HOMA aromaticity index	0.991112	0.980966	0.978494
18	Birds aromaticity index	90.677956	86.713907	86.943085

Table S1. Energies, electronic properties, and descriptors of calculated structures obtained by analysis of electron densities in Multiwfn, including conceptual DFT analysis.

7.2. Visualization of final geometries

Original files generated by ORCA after calculations are separately attached as additional supplementary information. Final geometries are available in *name_trj.xyz* files. All calculated DFT and MP2 energies and properties for singlet structures are available in *name_property.txt* files. N+1 electron states are described in *name+1_property*.txt and *name+1.xyz* files. N-1 electron states are described in *name-1_property*.txt and *name+1.xyz* files. Resulted electron densities are attached as *.cube files.

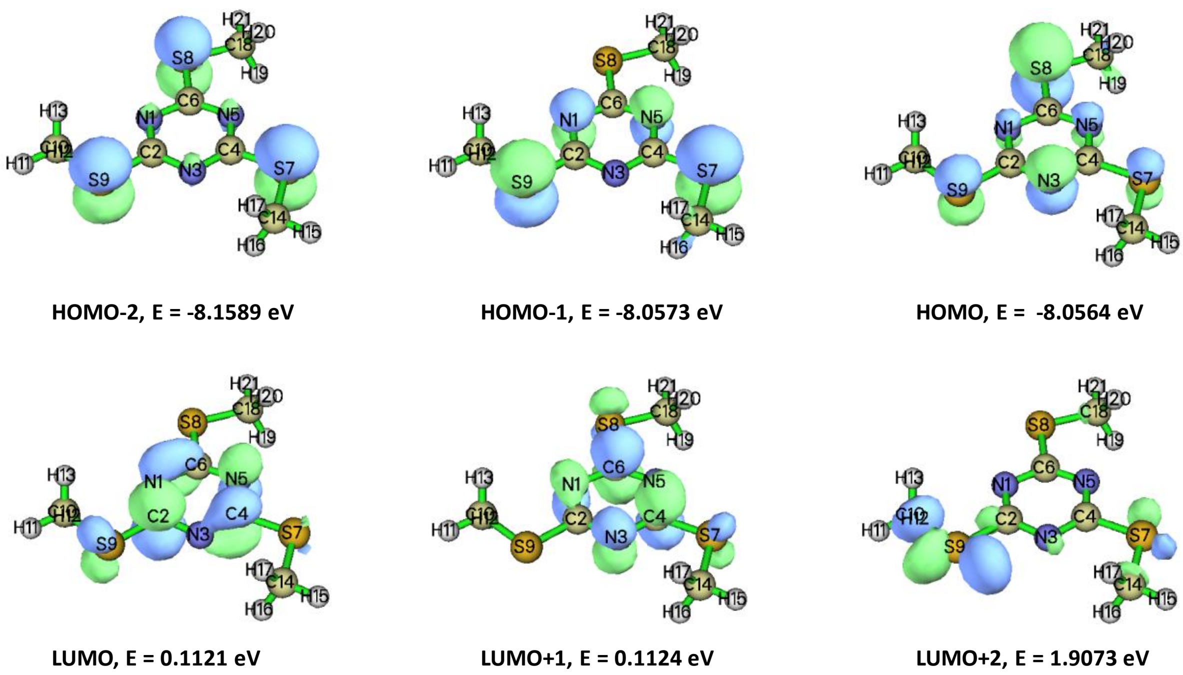

Figure S187. HOMO and LUMO orbitals of TMT(Me)$_3$. Isovalue 0.05.

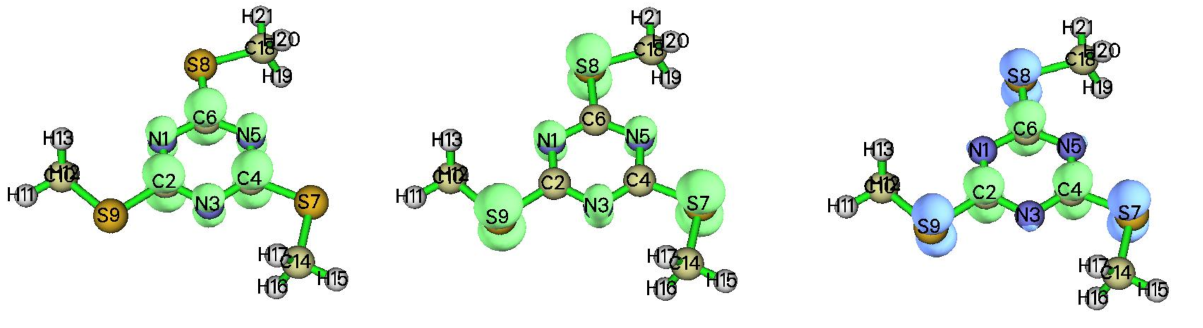

Figure S188. Gridta of orbital weighted Fukui functions and dual descriptor (F+, F-, and DD from left to right) calculated for TMT(Me)$_3$. Isovalue 0.005

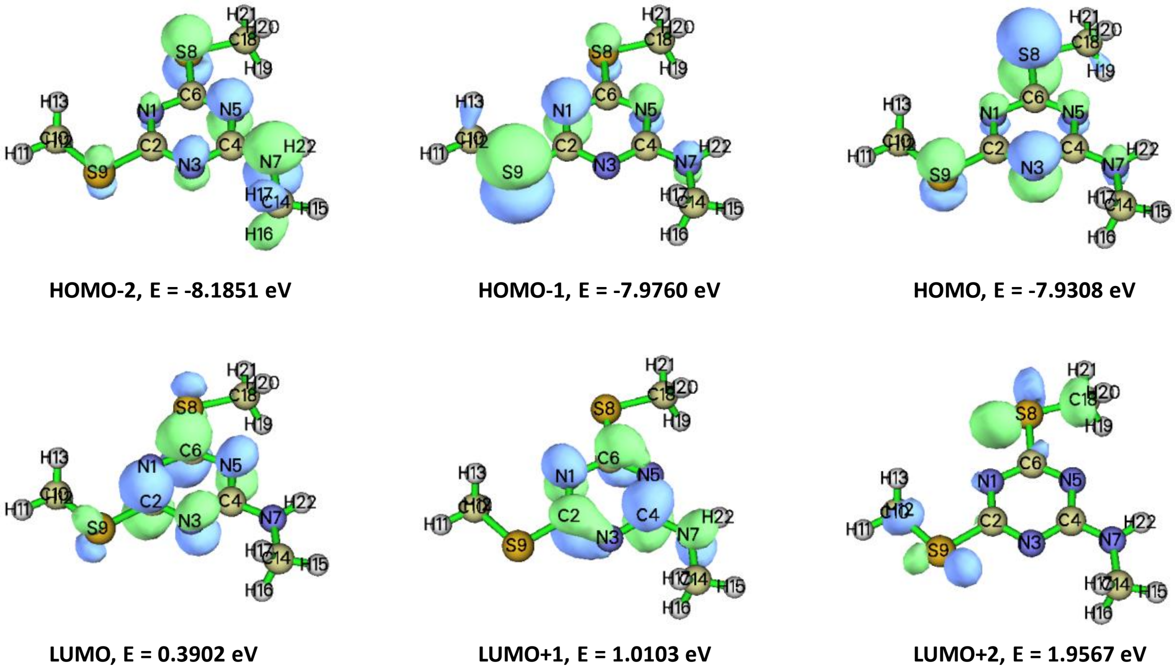

Figure S189. HOMO and LUMO orbitals of DMT(Me)$_2$(methylamine). Isovalue 0.05

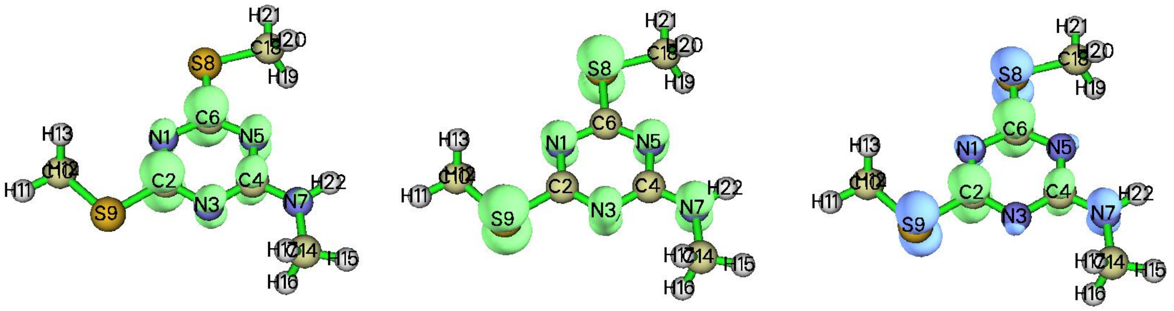

Figure S190. Gridta of orbital weighted Fukui functions and dual descriptor (F+, F-, and DD from left to right) calculated for DMT(Me)$_2$(methylamine). Isovalue 0.005

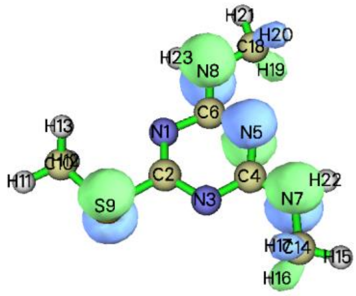

HOMO-2, E = -8.1251 eV

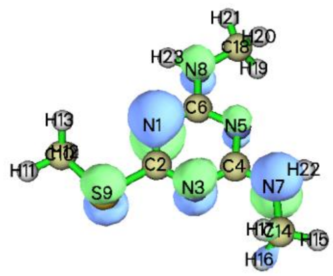

HOMO-1, E = -7.9019 eV

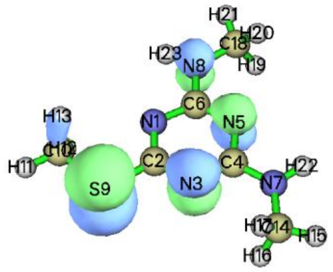

HOMO, E = -7.8163 eV

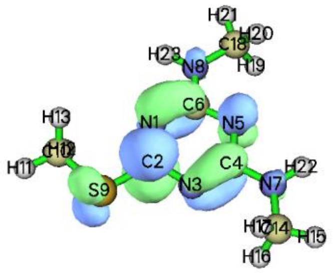

LUMO, E = 0.9513 eV

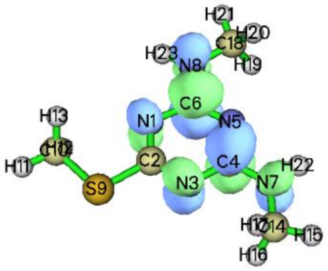

LUMO+1, E = 1.6468 eV

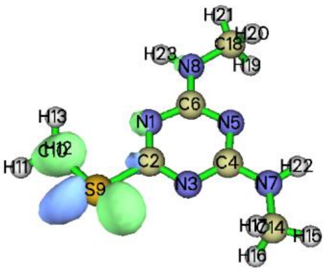

LUMO+2, E = 2.0394 eV

Figure S191. HOMO and LUMO orbitals of MMT(Me)(methylamine)$_2$. Isovalue 0.05

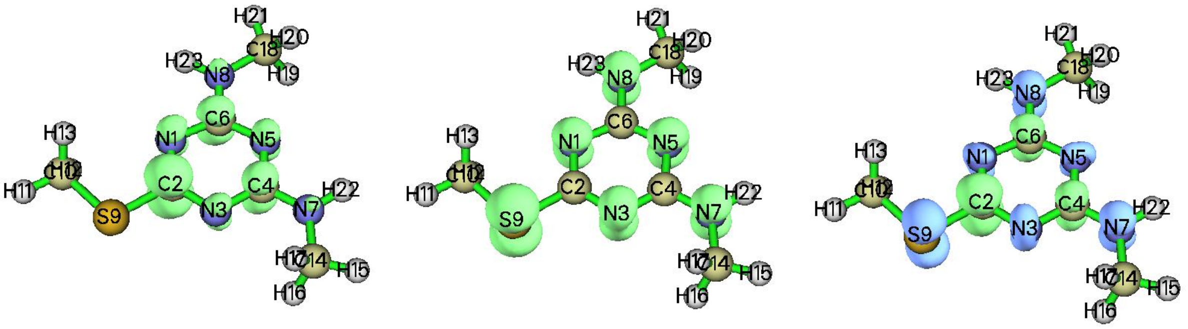

Figure S192. Gridta of orbital weighted Fukui functions and dual descriptor (F+, F-, and DD from left to right) calculated for MMT(Me)(methylamine)$_2$. Isovalue 0.005

7.3. Orbital weighted condensed Fukui indexes and dual descriptors

Atom	f+(TMT)	f+(DMT)	f+(MMT)	f-(TMT)	f-(DMT)	f-(MMT)	CDD(TMT)	CDD(DMT	CDD(MMT)
1 N	0,09289	0,07738	0,08067	0,07558	0,09529	0,08634	0,0173	-0,01791	-0,02811
2 C	0,1356	0,149	0,16184	0,02867	0,03399	0,0915	0,10693	0,11501	0,12355
3 N	0,09281	0,08609	0,07159	0,07564	0,09637	0,09123	0,01716	-0,01028	-0,04549
4 C	0,13557	0,08845	0,0932	0,02862	0,03096	0,0597	0,10695	0,05748	0,05638
5 N	0,09286	0,09446	0,07118	0,07558	0,08826	0,09136	0,01728	0,0062	-0,04085
6 C	0,13557	0,14806	0,09169	0,02862	0,03278	0,09042	0,10695	0,11528	0,05578
7 S(N)	0,07486	0,03369	0,03754	0,1962	0,09364	0,06367	-0,12135	-0,05994	-0,06586
8 S(N)	0,0749	0,09104	0,038	0,19631	0,20689	0,14897	-0,12141	-0,11585	-0,06441
9 S	0,07484	0,08947	0,11377	0,19632	0,21036	0,14992	-0,12148	-0,12089	-0,10361
10 C	0,01224	0,01757	0,0283	0,01674	0,01792	0,01775	-0,00451	-0,00035	0,00995
11 H	0,0045	0,00749	0,01354	0,00139	0,00153	0,00451	0,00311	0,00596	0,012
12 H	0,00665	0,01027	0,01823	0,00735	0,00786	0,00907	-0,0007	0,00242	0,01012
13 H	0,00663	0,0103	0,01822	0,00735	0,00787	0,00908	-0,00072	0,00244	0,01011
14 C	0,01223	0,01213	0,01835	0,01671	0,01609	0,01411	-0,00448	-0,00396	0,0007
15 H	0,0045	0,00634	0,01192	0,00139	0,00114	0,00374	0,00311	0,0052	0,01066
16 H	0,00663	0,00865	0,01392	0,00733	0,00852	0,00858	-0,0007	0,00013	0,00479
17 H	0,00663	0,0086	0,01442	0,00733	0,00837	0,00849	-0,0007	0,00023	0,00498
18 C	0,01222	0,01804	0,01829	0,01675	0,01753	0,01779	-0,00453	0,00051	0,00089
19 H	0,00665	0,01074	0,01449	0,00734	0,0077	0,00922	-0,00069	0,00303	0,00505
20 H	0,00663	0,01071	0,01412	0,00735	0,00771	0,00921	-0,00072	0,00301	0,00518
21 H	0,0045	0,0076	0,01199	0,00139	0,00145	0,00453	0,0031	0,00615	0,01076
22 H	0	0,01388	0,02196	0	0,00777	0,01082	0	0,00611	0,01325
23 H	0	0	0,02275	0	0	0	0	0	0,01418

Table S2. Orbital weighted condensed Fukui indexes and dual descriptors for TMT(Me)$_3$, DMT(Me)$_2$(methylamine), and MMT(Me)(methylamine)$_2$

-
